# *In vitro* and *in vivo* comparative and competitive activity-based protein profiling of GH29 α-l-fucosidases†
†Electronic supplementary information (ESI) available: Experimental part including synthesis procedures, characterization data, copies of NMR spectra of new compounds, supporting biological figures and crystallographic information. See DOI: 10.1039/c4sc03739a



**DOI:** 10.1039/c4sc03739a

**Published:** 2015-02-09

**Authors:** Jianbing Jiang, Wouter W. Kallemeijn, Daniel W. Wright, Adrianus M. C. H. van den Nieuwendijk, Veronica Coco Rohde, Elisa Colomina Folch, Hans van den Elst, Bogdan I. Florea, Saskia Scheij, Wilma E. Donker-Koopman, Marri Verhoek, Nan Li, Martin Schürmann, Daniel Mink, Rolf G. Boot, Jeroen D. C. Codée, Gijsbert A. van der Marel, Gideon J. Davies, Johannes M. F. G. Aerts, Herman S. Overkleeft

**Affiliations:** a Leiden Institute of Chemistry , Leiden University , P. O. Box 9502 , 2300 RA Leiden , The Netherlands . Email: h.s.overkleeft@chem.leidenuniv.nl ; Email: j.m.aerts@amc.uva.nl; b Department of Medical Biochemistry , Academic Medical Center , Meibergdreef 15 , 1105 AZ Amsterdam , The Netherlands; c Department of Chemistry , University of York , Heslington , York , YO10 5DD , UK; d DSM Innovative Synthesis , Urmonderbaan 22 , NL-6167 RD Geleen , The Netherlands

## Abstract

GH29 α-l-fucosidases catalyze the hydrolysis of α-l-fucosidic linkages. Deficiency in human lysosomal α-l-fucosidase (FUCA1) leads to the recessively inherited disorder, fucosidosis. Herein we describe the development of fucopyranose-configured cyclophellitol aziridines as activity-based probes (ABPs) for selective *in vitro* and *in vivo* labeling of GH29 α-l-fucosidases from bacteria, mice and man. Crystallographic analysis on bacterial α-l-fucosidase confirms that the ABPs act by covalent modification of the active site nucleophile. Competitive activity-based protein profiling identified l-fuconojirimycin as the single GH29 α-l-fucosidase inhibitor from eight configurational isomers.

## Abstract 2

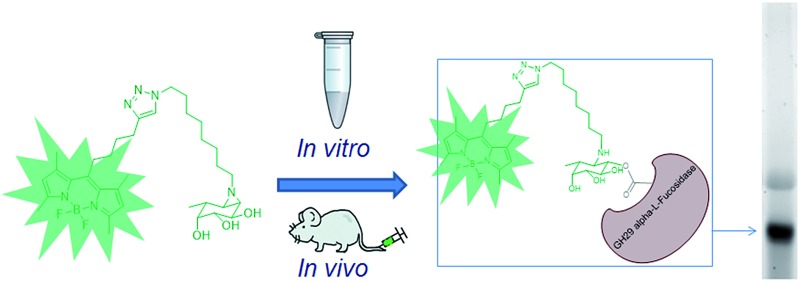
Development of probes for active GH29 α-l-fucosidases.

## Introduction

**Fig. 1:**
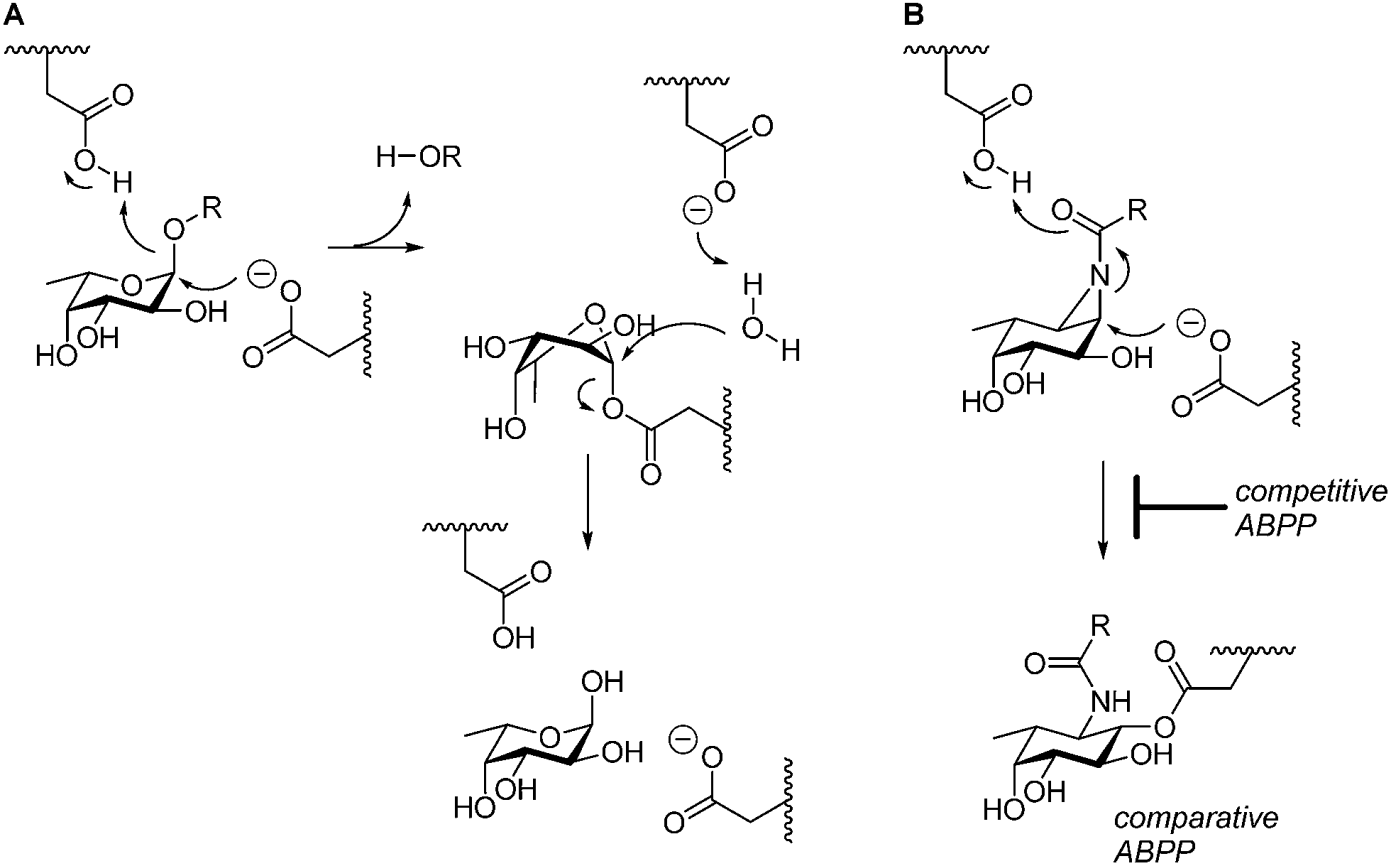
(A) Double-displacement mechanism of retaining α-l-fucosidases. (B) Comparative and competitive activity-based profiling of GH29 α-l-fucosidases presented here.

GH29 α-l-fucosidases catalyze the hydrolysis of terminal α-l-fucosidic linkages.^1^ The GH29 (ref. 2) glycoside hydrolase family of retaining α-l-fucosidases contains members from various kingdoms of life including the eukaryota^3^ and bacteria (for example, *Bacteroides thetaiotaomicron*,^4^ *Sulfolobus solfataricus*,^5^ and *Thermotoga maritima*^6^). GH29 α-l-fucosidases process their substrate with overall retention of configuration at the anomeric center of the cleaved fucopyranose and do so through a double-displacement mechanism. In this mechanism, first proposed by Koshland,^7^ (Fig. 1A), S_N_2 displacement of the aglycon (activated through protonation by the general acid/base residue) by nucleophilic attack of the catalytic nucleophile yields a fucosyl-enzyme intermediate, which is subsequently hydrolyzed to yield α-l-fucopyranose together with the released aglycon.

Two GH29 α-l-fucosidases are expressed in man. Of these, FUCA1 is found in lysosomes whereas FUCA2 is secreted into the plasma.^8^ Deficiency of FUCA1 α-l-fucosidase activity causes fucosidosis,^9^ a rare autosomal recessive lysosomal storage disorder. Next to its role in lysosomal turnover of fucosylated substrates, FUCA1 is also involved in sperm transport and sperm–egg interactions.^10^ FUCA1 activity levels are considered to be a biomarker for cellular senescence^11^ as well as for the diagnosis of hepatocellular cancers.^12^ Deficiency of human FUCA2 has been shown to protect against *Helicobacter pylori* adhesion to gastric cancer cells.^8^

**Fig. 2:**
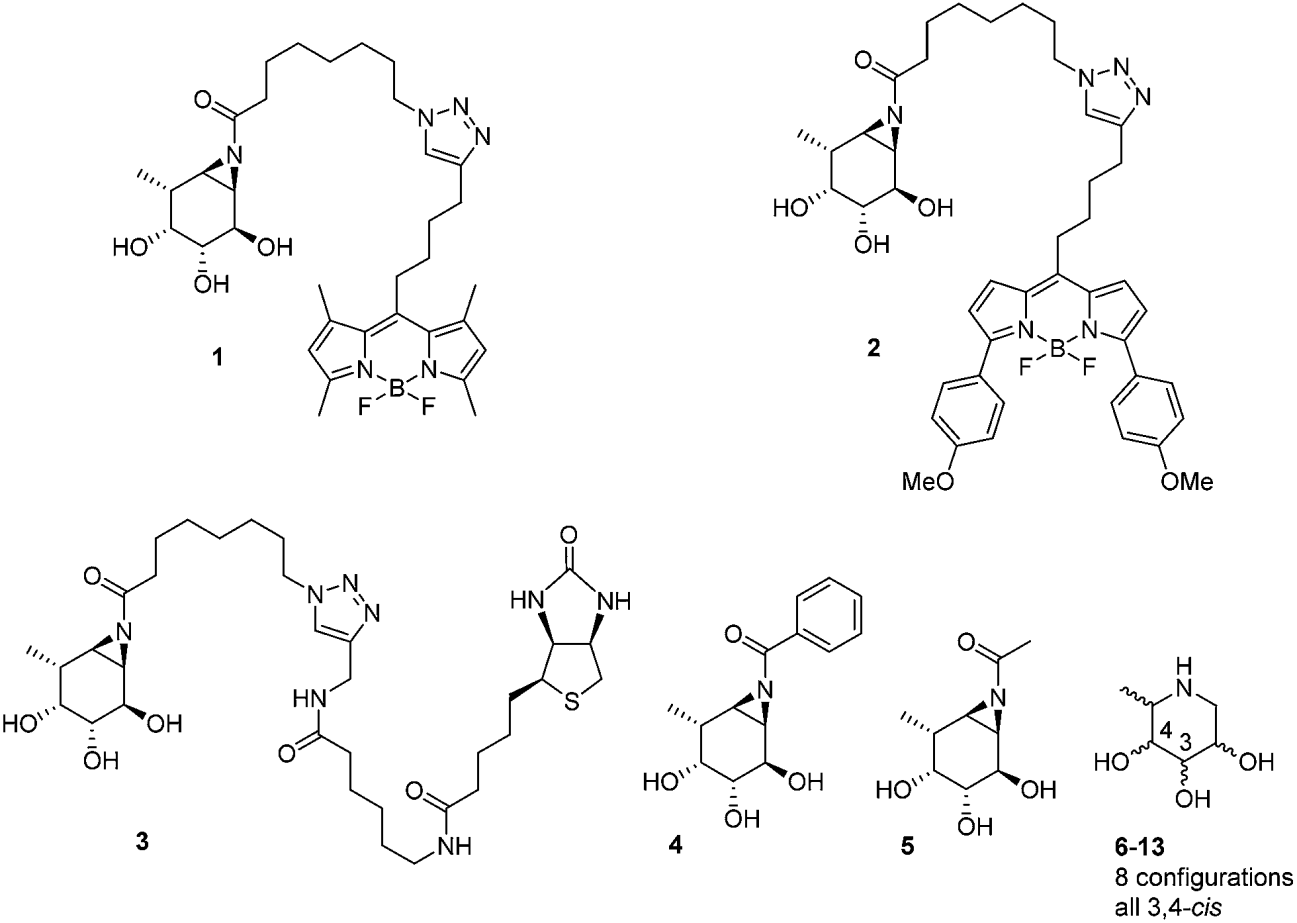
Structures of inhibitors and ABPs presented in this study.

The biological and biomedical relevance of GH29 retaining α-l-fucosidases warrant the development of efficient methods to monitor their functional state and activity *in vitro*, *in situ* and *in vivo*. In this respect, activity-based probes (ABPs) have shown their merit as tools to detect active enzyme molecules in their native environment (Fig. 1B).^13^ We have previously reported the use of cyclophellitol aziridines as scaffolds for the design of *in situ* and *in vivo* active ABPs directed at GH1 retaining β-glucosidases^14^ and GH27 retaining α-galactosidases.^15^ The specificity of these probes appeared due to their configuration, with the β-glucopyranose configured cyclitol aziridine being highly selective towards retaining β-glucosidases and their α-galacto-configured counterparts selective towards α-galactosidases. Here we describe the development of retaining GH29 α-l-fucosidase ABPs. The ABPs are based on the cyclophellitol aziridine structure having α-l-fucoside configuration and are equipped with a green (**1**, JJB256) or red (**2**, JJB244) BODIPY fluorophore and biotin tag (**3**, JJB243) (Fig. 2). We reveal that these probes are highly sensitive and selective and can be used for *in situ* and *in vivo* monitoring of mammalian and bacterial GH29 retaining α-l-fucosidases.

We also demonstrate that ABPs **1** and **2** can be used in a competitive activity-based protein profiling (ABPP) assay^16^ to identify rapidly retaining α-l-fucosidase inhibitors from a library of eight configurational isomers of deoxy-l-fuconojirimycin (**6–13**); a library we prepared specifically for this purpose. Finally we unambiguously establish the validity of the cyclophellitol aziridine design platform for ABP development of retaining glycosidases by solving the crystal structure of retaining α-l-fucosidase from *Bacteroides thetaiotaomicron* 2970, covalently bound to *N*-acyl cyclophellitol aziridines **4** and **5**.

## Results and discussion

**Scheme 1:**
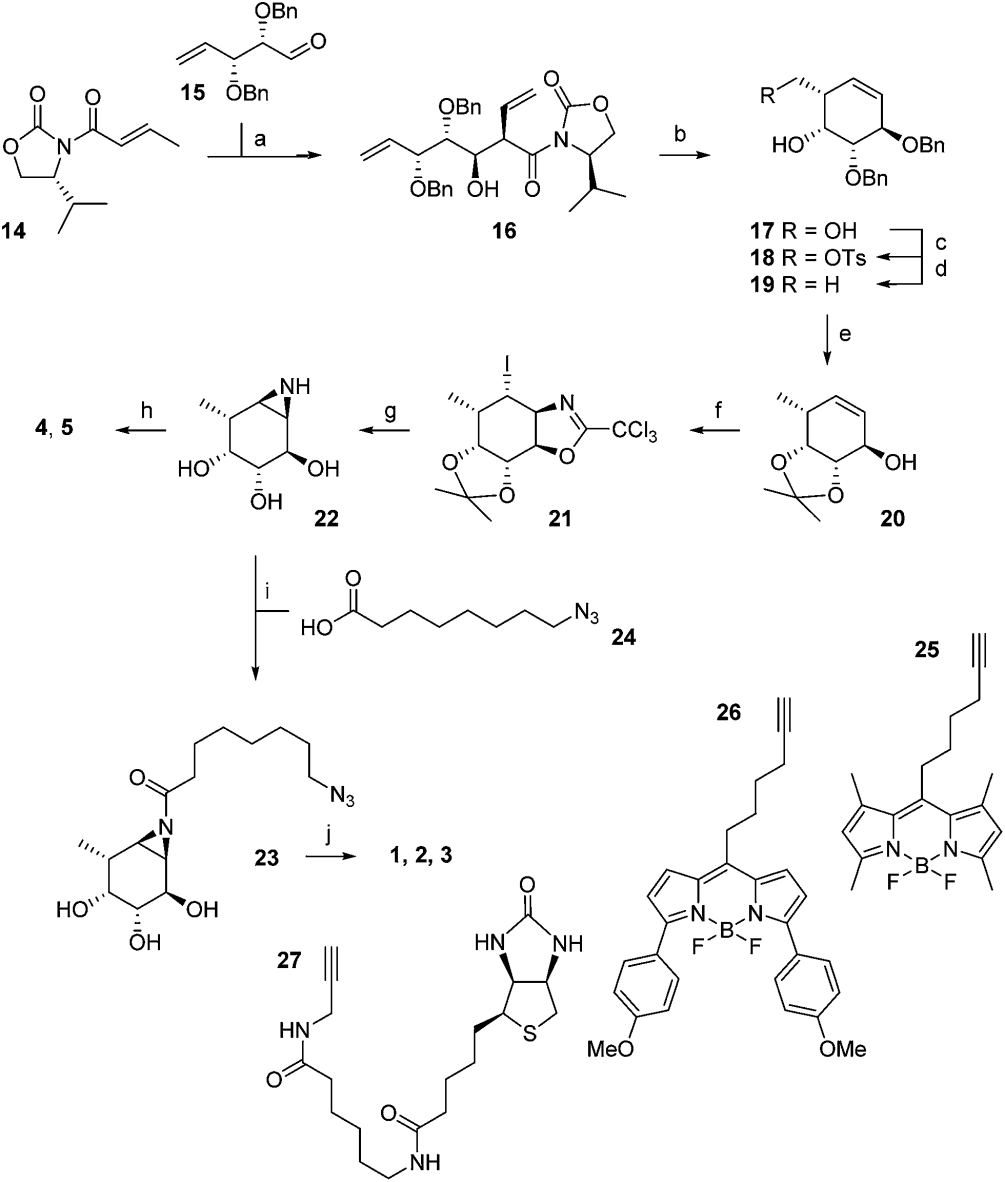
Synthesis of aziridine ABPs **1**, **2**, **3** and inhibitors **4**, **5**. Reagents and conditions: (a) DBBT, Et_3_N, CH_2_Cl_2_, –78 °C, 71%; (b) (i) LiBH_4_, THF, 83%; (ii) Grubbs 2^nd^ generation, CH_2_Cl_2_, 95%; (c) *p*-TsCl, Et_3_N, CH_2_Cl_2_, 87%; (d) LiAlH_4_, THF, 0 °C to rt, 87%; (e) (i) Li, NH_3_ (l), THF, –60 °C, 73%, (ii) 2,2-dimethoxypropane, CSA, rt, 60%; (f) (i) CCl_3_CN, DBU, CH_2_Cl_2_, rt; (ii) NaHCO_3_, I_2_, H_2_O, rt, 46%; (g) (i) 37% HCl (aq.), MeOH, 60 °C; (ii) NaHCO_3_, MeOH, rt, 65%; (h) EEDQ, benzoic acid or acetic acid, DMF, 0 °C, 7% **4**, 28% **5**; (i) EEDQ, **24**, DMF, 0 °C 25%; (j) CuSO_4_, sodium ascorbate, **25**, **26** or **27**, rt, 19% **1**, 12% **2**, 13% **3**.

The synthesis of α-l-fucopyranose-configured cyclophellitol aziridine-based target compounds started with aldol condensation of aldehyde **15** and chiral acrylamide **14**, following the procedure reported^17^ by Llebaria and co-workers for the enantiomer of **16** (Scheme 1). Reductive removal of the Evans template in **16** followed by ring-closing metathesis yielded according to the Llebaria procedure^17^ partially protected l-galactopyranose-configured cyclohexene **17** in good yield. Tosylation of the primary alcohol in **17** was followed by hydride displacement of the tosylate to afford l-fucopyranose-configured cyclohexene **19**. The benzyl groups in **19** were reduced under Birch conditions, after which the *cis*-diol was protected as the isopropylidene acetal to give **20**. The secondary alcohol in **20** was transformed into the corresponding trichloroacetimidate after which iodocyclisation yielded in a stereospecific fashion intermediate **21** analogous to the procedure we reported for the synthesis of retaining β-glucosidase ABPs.^18^ Acidic hydrolysis of both acetal and iminal in **21** was followed by base-induced intramolecular nucleophilic substitution of the iodine to yield aziridine **22**. Acetylation or benzoylation of the aziridine in **22** under the agency of 2-ethoxy-1-ethoxycarbonyl-1,2-dihydroquinoline (EEDQ) following conditions we had developed previously^18^ yielded compounds **4** and **5**, respectively. EEDQ-induced acylation of **22** with 8-azidooctanoic acid^19^ **24** provided azide **23**, which was transformed into target ABPs **1**, **2** and **3** by conjugation to BODIPY-alkynes **25**, **26** and biotin-alkyne **27**, respectively, *via* copper(i)-catalyzed Huisgen [2 + 3] cycloaddition. The final compounds were purified by reverse phase HPLC.

**Fig. 3:**
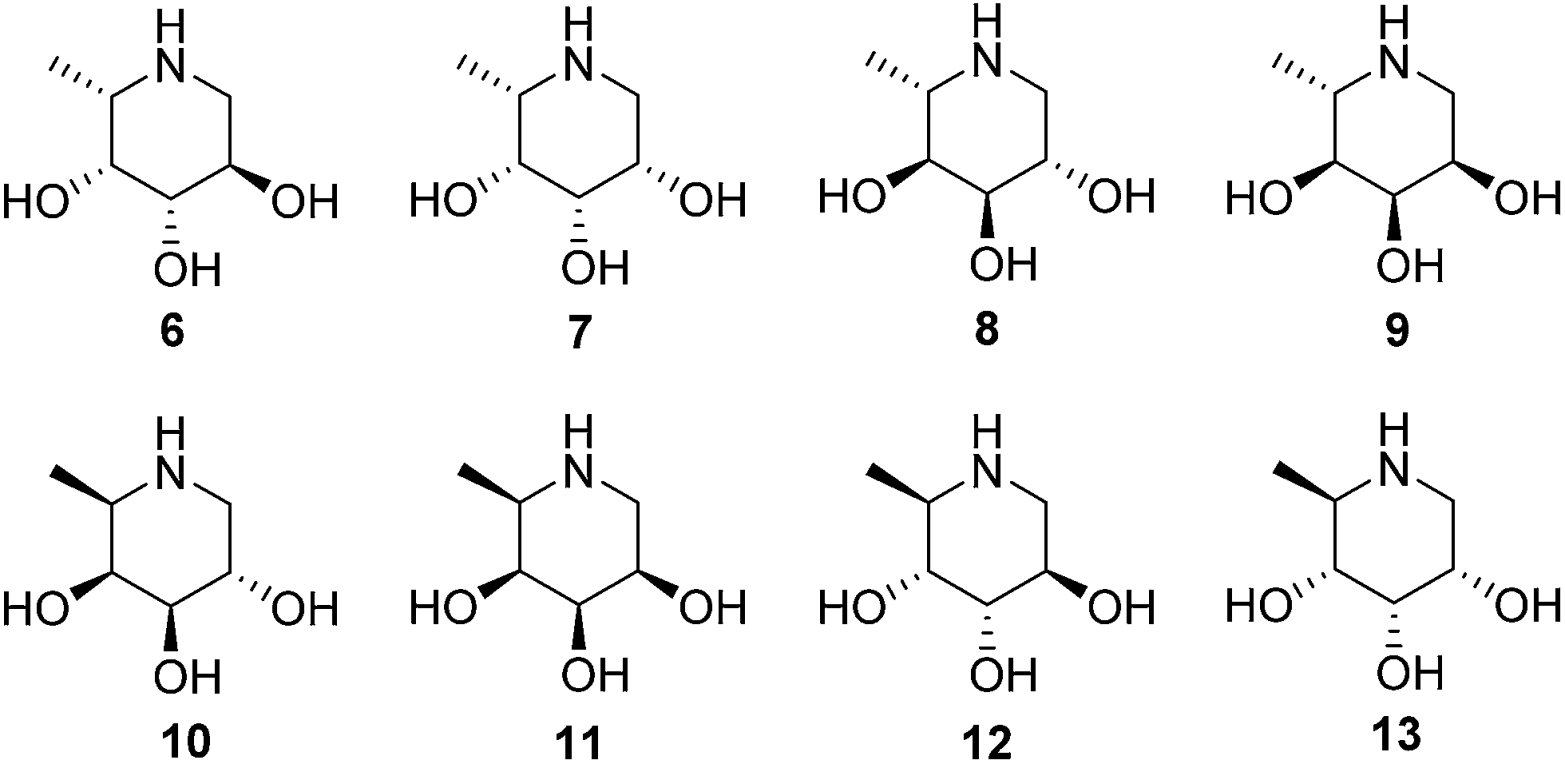
Structures of 1-deoxy-l-fuconojirimycin **6** and configurational isomers **7–13**.

**Scheme 2:**
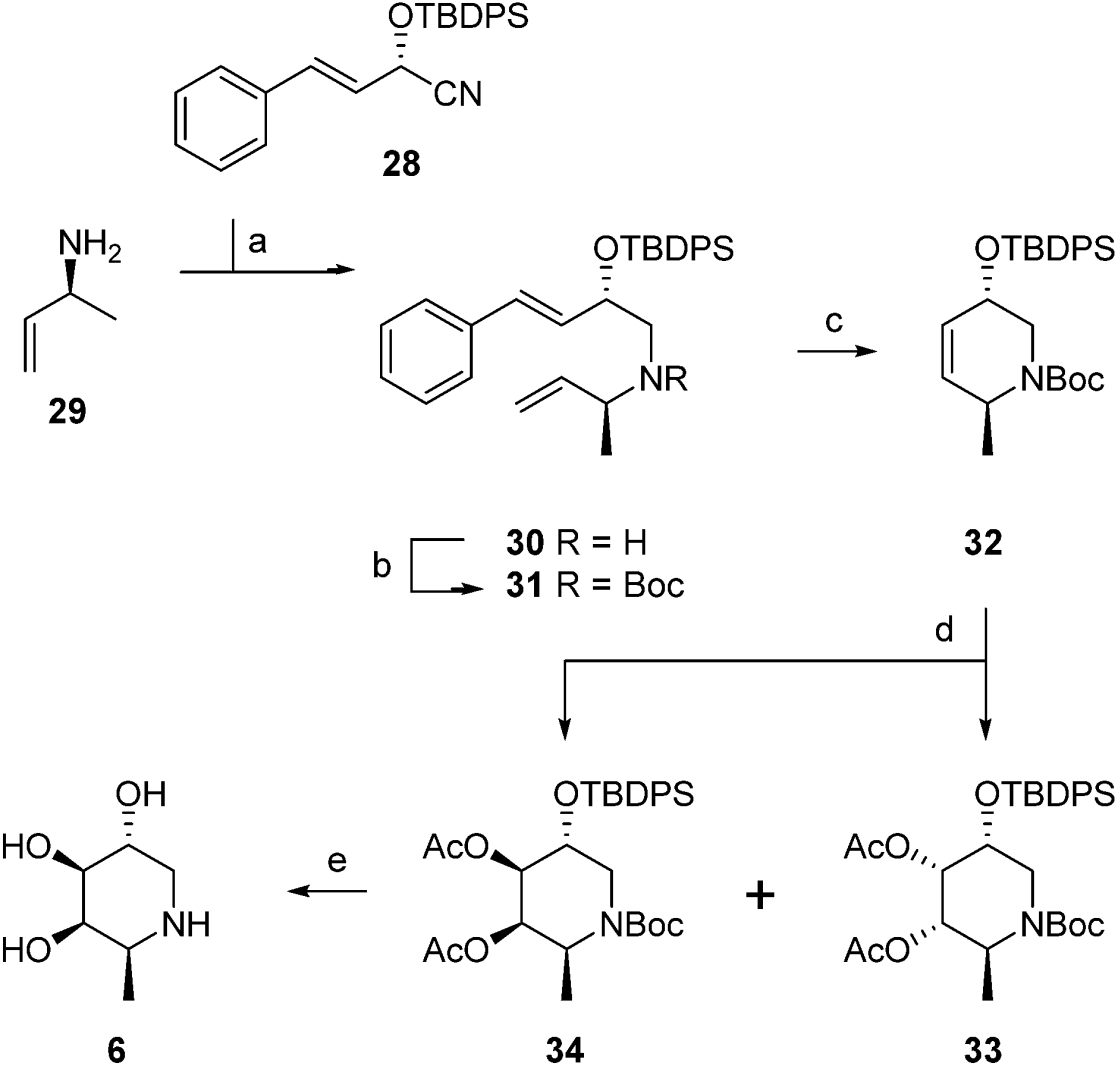
Synthesis of 1-deoxy-l-fuconojirimycin **6**. Reagents and conditions: (a) (i) Et_2_O, DIBAL-H, –80 °C, (ii) MeOH, –90 °C, (iii) amine **29**, NaOMe, (iv) NaBH_4_, –15 °C to rt, 88%; (b) Boc_2_O, 50 °C, 100%; (c) Grubbs 1^st^ generation, CH_2_Cl_2_, 88%; (d) (i) K_2_OSO_4_·2H_2_O, NMO, acetone–H_2_O (1 : 1), –10 °C, (ii) Ac_2_O, pyridine, DMAP, 0 °C, 88% (**33** : **34** = 1 : 3); (e) (i) K_2_CO_3_, MeOH, (ii) TBAF, THF, (iii) 6M HCl, MeOH, 71%.

The configurational fuconojirimycin isomers **6–13** (Fig. 3) were synthesized following the strategy exemplified for 1-deoxy-l-fuconojirimycin **6** (Scheme 2). Key steps in the synthetic scheme include a DIBAL-H reduction-transimination-sodium borohydride reduction cascade of reactions involving enantiomerically pure cyanohydrin **28**, prepared employing (*S*)-hydroxynitrile lyase (*S-Hb*HNL), from *Hevea brasiliensis* rubber tree,^20^ and the allylic amine **29** prepared by reported strategy,^21^ to give secondary amine **30**. *N*-Boc protection (**30** to **31**), ring-closing metathesis (**31** to **32**) and Upjohn dihydroxylation afforded a mixture of *syn*-diols, which were acetylated and separated by silica gel purification to yield diastereomers **33** and **34** in a 1 : 3 ratio.

Global deprotection of **34** afforded 1-deoxy-l-fuconoijirimycin **6**, the analytical and spectroscopical data of which were in full agreement with those reported in the literature.^22^ The seven configurational isomers **7–13** were prepared by alteration of the building blocks and/or the chemical transformations. It should be noted that four out of the eight isomers of fuconojirimycin are derived from **28**. To enable the synthesis of the four enantiomers we needed access to the enantiomer of cyanohydrin **28**, which we prepared using almond (*R*)-hydroxynitrile lyase (*R-Pa*HNL).^23^

### *In vitro* GH29 α-l-fucosidase activity assays

Having the cyclophellitol aziridine inhibitors and probes in hand, we determined their inhibitory potency towards the human lysosomal α-l-fucosidase, FUCA1. Inhibition potency was determined by measuring the residual enzyme activity using the fluorogenic substrate 4-methylumbelliferyl-α-l-fucopyranoside after pre-incubation of lysates of COS-7 cells over-expressing recombinant human FUCA1, with varying concentrations of the non-fluorescent, irreversible cyclophellitol aziridine inhibitors **4**, **5** and **23**; the ABPs **1** (JJB256), **2** (JJB244), **3** (JJB243), 1-deoxy-l-fuconojirimycin **6**, and the seven fuconojirimycin isomers **7–13**. All *N*-acyl aziridines inhibited human FUCA1 activity with nanomolar IC_50_ activities (Table 1).

**Table 1:** *In vitro* and *in situ* inhibition of recombinant human GH29 α-l-fucosidase, given as half-maximal inhibitory concentration (IC_50_)^*a*^

	*In vitro* IC_50_	*In situ* IC_50_
**Aziridine**		
**1** (JJB256)	6.9 ± 0.8	28.9 ± 4.4
**2** (JJB244)	8.7 ± 1.2	157.8 ± 22
**3** (JJB243)	7.2 ± 1.1	25 512 ± 2278
**4**	371.6 ± 21.2	515.4 ± 72.9
**5**	46.8 ± 3.4	77.9 ± 9.1
**23**	8.7 ± 1.1	65.4 ± 7.5

**Iminosugars**		
**6**	3979 ± 257	ND
**7–13**	>100 000	ND

^*a*^Values are given in nanomolar. ND: not determined. It should be noted that IC_50_ values on competitive inhibitors do not compare well to those obtained for mechanism-based inhibitors. The values given above allow for a comparison of inhibitory potency within the two classes of compounds studied.

Perusal of the inhibitory data does reveal some trends that allow us to draw some tentative conclusions. *N*-Benzoyl aziridine **4**, bearing a bulky aromatic *N*-benzoate group, is about eight-fold less active compared to its *N*-acetylated counterpart, **5**. In contrast, comparing **5** with **23** reveals that a bulky aliphatic *N*-acyl substituent is in fact favored for enzyme inhibition (5-fold increase, Table 1). ABPs **1** and **2**, bearing a BODIPY fluorophore attached to the alkyl chain, inhibit FUCA1 in the same order as their azide precursor **23**. *In situ* inhibition of FUCA1 in living fibroblasts by ABP **1** and **2** occurs with similar efficacy as **23** and ABP **3**, the latter carrying a biotin attached to the alkyl chain. Exposure of cells to ABP **3** revealed in a 25-fold decreased inhibitory potency, suggesting a reduced ability to penetrate into cells to reach the lysosomal FUCA1. The known competitive fucosidase inhibitor, 1-deoxy-l-fuconojirimycin **6** inhibits FUCA1 with an IC_50_ of 3.9 μM, in accordance with the literature values.^24^ The seven configurational isomers **7–13** do not significantly inhibit FUCA1 activity up to 100 μM, a result that corroborates previous findings on some of the configurational analogues, which were reported as poor fucosidase inhibitors.^25^

**Fig. 4:**
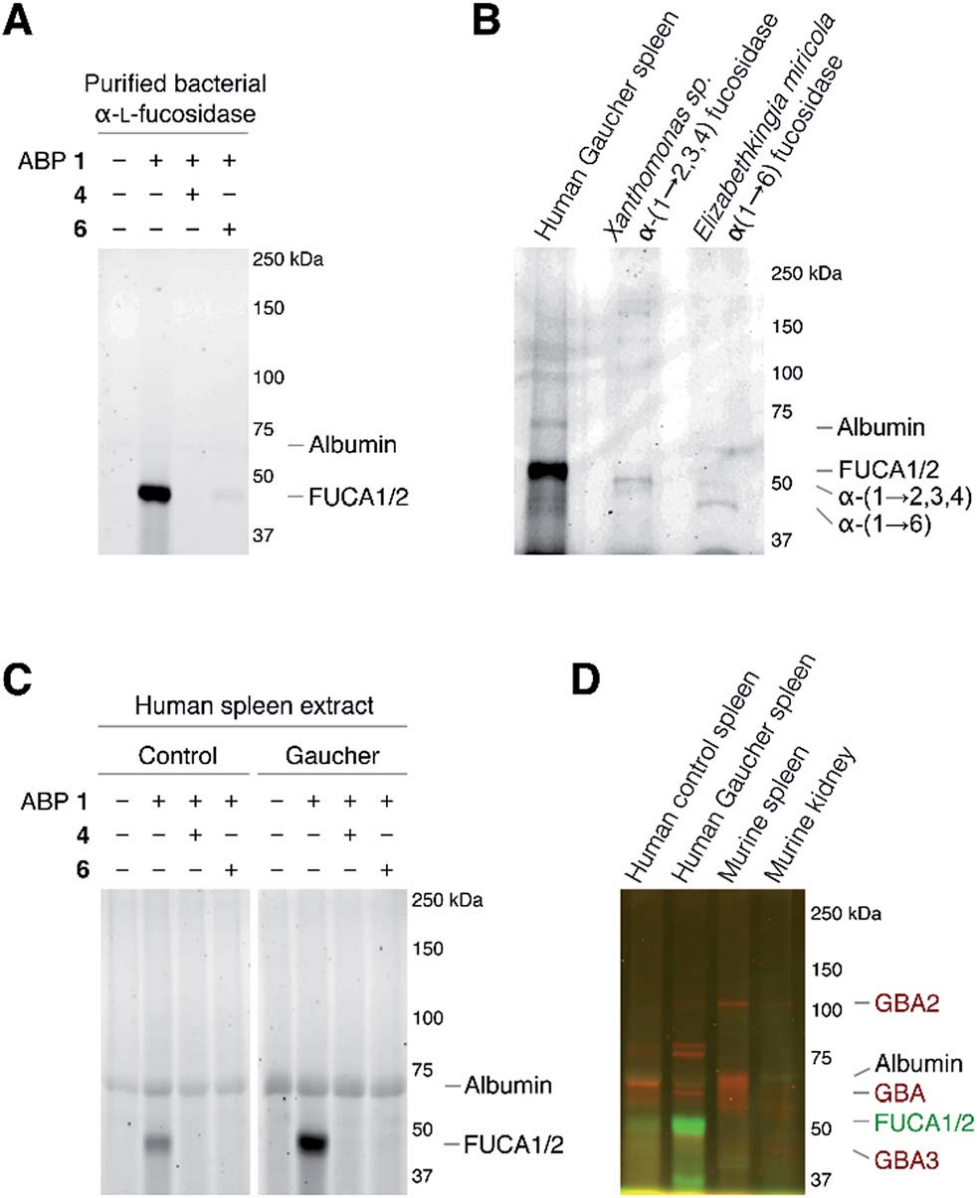
*In vitro* activity-based protein profiling of GH29 α-l-fucosidases. (A) Labeling with ABP **1** of recombinant α-l-fucosidase from *Bacteroides thetaiotaomicron* 2970. (B) *In vitro* labeling of lysate of spleen from a Gaucher disease patient, α-(1-2,3,4)-fucosidase from *Xanthomonas* sp. and α-(1-6)-fucosidase from *Elizabethkingia miricola* with ABP **2**. (C) *In vitro* labeling of human healthy and Gaucher disease spleen. (D) Direct labeling of GH29 α-l-fucosidases with green-fluorescent ABP **1** and retaining β-glucosidases GBA, GBA2 and GBA3 with red-fluorescent JJB75.^18^ The location of albumin autofluorescence is designated on each gel.

As the next research objective, we examined activity-based profiling of GH29 α-l-fucosidases from varying sources with green-fluorescent aziridine ABP **1**, in the presence or absence of excess concentrations of, either the mechanism-based inhibitor **4** or the competitive inhibitor **6**. As is shown in Fig. 4A, ABP **1** efficiently labels purified α-l-fucosidase from *Bacteroides thetaiotaomicron*. In lysates from an *E. coli* culture overexpressing recombinant α-l-fucosidase from *Bacteroides thetaiotaomicron* gene 2970, several fluorescent protein bands are visible upon labeling with **1** (ESI Fig. S1†), with the most prominent band at around 50 kDa, corresponding to the predicted molecular weight of the enzyme. Labeling of the major band at 50 kD could moreover be blocked following pre-incubation with either 100 μM **4** or with 5 mM **6** (ESI Fig. S1†). Red fluorescent ABP **2** labels α-(1-2,3,4) and α-(1-6)-fucosidases from various bacterial sources in a similar fashion (Fig. 4B).

Subsequently, we exposed lysates of spleens from a healthy human individual and a patient suffering from Gaucher disease to ABP **1**. As can be seen in Fig. 4C, a single fluorescent protein migrating slightly below 50 kDa was fluorescently labeled. The labeled band from human Gaucher spleen lysate is considerably more intense than the corresponding band in healthy human spleen, reflecting elevated α-l-fucosidase activity in the former. This result corroborates earlier observations that mRNA encoding lysosomal glycosidases are upregulated in Gaucher tissue.^26^ As before, labeling with ABP **1** was suppressed after pre-treatment with either *N*-benzoyl aziridine **4** or l-fuconojirimycin **6**. To further ascertain that ABP **2** labels mammalian GH29 retaining α-l-fucosidases we performed a number of control experiments (ESI Fig. S2a†). Compound **1** labeled both FUCA1 and FUCA2 that were overexpressed in COS cells. Treatment of murine spleen lysates with **1** yielded a result essentially as observed for healthy human tissue (ESI Fig. S2b†).

Finally, we evaluated the simultaneous labeling of retaining β-glucosidases and retaining α-l-fucosidase. To this end we incubated tissue lysates with **1** and our previously^18^ reported broad-spectrum activity-based retaining β-glucosidase probe, JJB75 (see for its structure, ESI Fig. S3†). As can be seen (Fig. 4D) the applied ABPs label a distinct set of proteins. Since they bear complementary fluorophores they can be used jointly to profile both retaining glycosidase families in a single experiment.

The FUCA1 enzymatic activity is maximal at around pH 4.5 (see ESI Fig. S4†), consistent with the acidic pH of its natural lysosomal environment. Labeling efficiency with **1** largely reflects the pH dependence of FUCA1 at pH below 7. Of note, the pH dependence of labeling of FUCA1 does not follow that of its enzymatic activity. At alkaline conditions, where enzymatic activity is low, labeling still proceeds. We made similar observations in the past for ABPP of retaining β-exoglucosidases using cyclophellitol β-aziridine ABPs.^14^ This result reveals the high reactivity of the aziridine probes in the initial displacement step employed by retaining glycosidases, a feature which, in conjunction with their selectivity in binding and relative stability in physiological environment, explains their efficacy as activity-based glycosidase probes.^27^

Assessment of fluorescent labeling kinetics by employing 10 nM **1** labeling at stoichiometric concentrations of rhFUCA1 at 4 °C and 37 °C revealed that, during time-course experiments, examined on SDS-PAGE gels, labeling is near complete within the first minute at 4 °C (ESI Fig. S5a†), impairing accurate determination of kinetic constants. This rapid reaction with both **1** and **2** also precluded a classic determination of kinetic constants following a time-dependent decrease in detectable 4-methylumbelliferone (ESI Fig. S5b†).

### *In vivo* GH29 α-l-fucosidase assays

The ability of **1** to label α-l-fucosidase in living mice was investigated next. Four wild-type C57Bl/6J male mice were injected with 100 μL vehicle (PBS) or PBS containing 10, 100 or 1000 pmol **1**. After two hours, the mice were anesthetized, perfused with PBS and then brain, spleen, liver and kidney tissues were isolated. Tissue homogenates were prepared and each lysate was labeled prior to gel electrophoresis with red-fluorescent cyclophellitol β-aziridine JJB75, which labels β-exoglucosidases as loading control (ESI Fig. S6†). Furthermore, tissue homogenates of vehicle-treated animals were labeled with excess **1** to visualize the maximal α-l-fucosidase labeling achievable in each tissue.

**Fig. 5:**
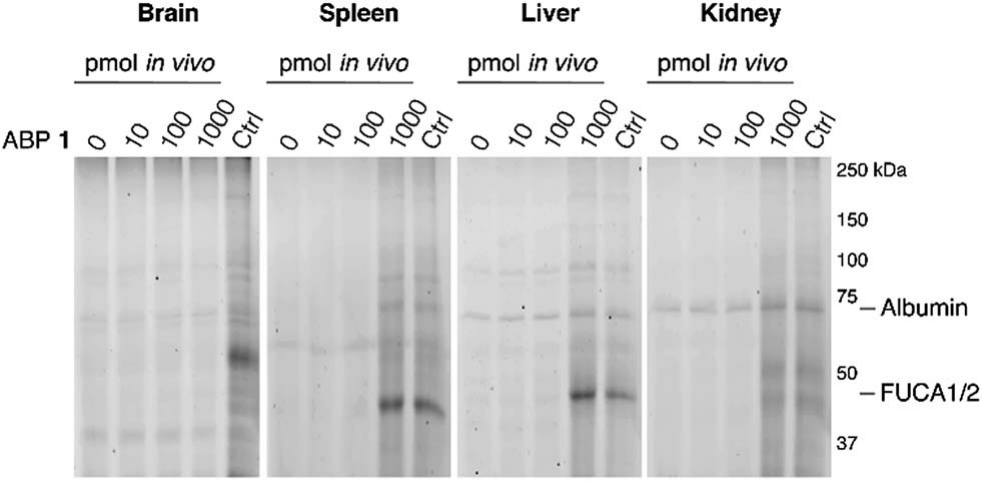
*In vivo* labeling of α-l-fucosidases in mice with various concentrations of ABP **1** during 2 hours. *In vivo* labeling compared to maximal labeling with excess ABP **1** of matched homogenates (Ctrl). The location of albumin autofluorescence is designated on each gel.

After treatment of mice with ABP **1**, a dose-dependent labeling of retaining α-l-fucosidases is observed in spleen, liver and kidney (Fig. 5). Injection of 1000 pmol **1** results in substantial labeling of α-fucosidase in spleen, liver and kidney. Detected fluorescence levels are comparable to that in matching samples from vehicle-treated mice incubated *in vitro* with excess 1. In contrast, no *in vivo* brain FUCA1 labeling was observed after any of the administered doses of ABP **1**, and we conclude that ABP **1**, just as its β-glucose and α-galactose congeners,^14,15^ does not penetrate the brain.

### Competitive activity-based GH29 α-l-fucosidase profiling

**Fig. 6:**
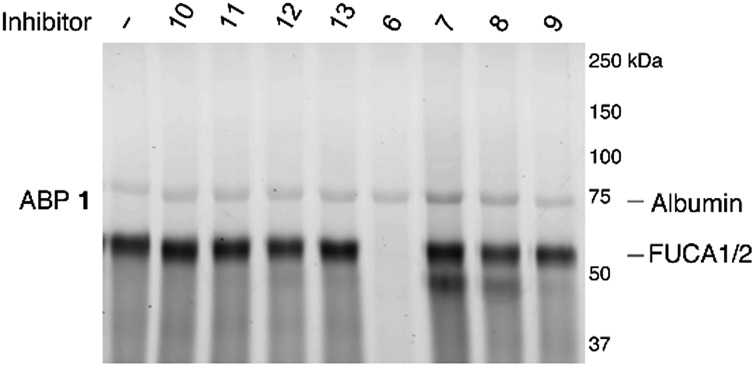
Competitive ABPP on recombinant FUCA1 with deoxyfuconojirimycin **6** and configurational analogues **7–13** towards α-l-fucosidases, with ABP **1** labeling as readout.

Having established the efficacy of ABP **2** to selectively label GH29 retaining α-l-fucosidases from various sources in an activity-based manner, we determined the inhibition potential of deoxyfuconojirimycin **6** and its **7** stereoisomers **7–13** in a competitive ABPP format (Fig. 6). In contrast to **6**, none of the seven configurational isomers **7–13** were capable of blocking ABP **1** labeling of α-l-fucosidase, a result that matches with the data on inhibition of recombinant FUCA1 in the fluorogenic activity assay (Table 1).

### Identification of biotin labeled GH29 α-l-fucosidases

To further determine the specificity of the developed ABPs, we have analyzed the ABP-labeled proteins in a complex tissue homogenate. For this purpose, we incubated Gaucher spleen lysate with biotinylated ABP **3** or with DMSO only (control) or competitive ABP **3**-labeling by first incubating with fluorescent ABP **1**. Glycosylated proteins were then enriched *via* ConA, followed by affinity purification with streptavidin coated paramagnetic beads. The identity of biotinylated and ABP **3**-labeled proteins was determined by on-bead digestion with trypsin, peptide analysis by LC-MS/MS and matching against the human UniProt database, using the Mascot search engine as previously reported.^14^ FUCA1 was identified after ABP **3** pull-down as one of the top identified proteins (Table S1A) in the ESI data, but was not found in the competitive (Table S1B) or untreated control (Table S1C†). Proteins with higher scores were background proteins such as abundant endogenously biotinylated PCCA and PC, and keratin contaminations. FUCA1 was selectively found in the ABP **3**-pull down experiment only. ESI Table S2† shows the analysis parameters of the identified peptides from FUCA1, with accuracy below 5 ppm, and Mascot ion scores above 40 (indicating reliable MS/MS fragment annotation and match) and manually curated fragmentation patterns. These results show FUCA1 can be undisputedly affinity purified and identified *via* biotinylated ABP **3**. Moreover the binding of ABP **3** can be completely blocked by pre-incubation with ABP **1**, which may indicate that both ABPs bind at the same site of the enzyme.

### 3-D crystal structure analysis of bacterial FUCA1 complexed to **4** and **5**

In order to obtain experimental evidence for the formation of a covalent adduct between α-l-fucosidase and ABPs **1**, **2** and **3**, a crystal structure of *Bt*Fuc2970 (often used as a surrogate for the mammalian enzyme) with mechanism-based inhibitor **4** was obtained (PDB code ; 4WSK). While the resulting crystal structure clearly demonstrates the formation of an enzyme–**4** complex, electron density around the aryl group of the inhibitor “aglycon” moiety was close to the side-chain of the catalytic acid/base of the enzyme, residue E288 (ESI Fig. S7†) resulting in disorder. This likely reflects steric clashing and considerable conformational flexibility in the **4** aglycon when bound to *Bt*Fuc2970. “Aglycon” here reflects the aziridine *N*-acyl moiety to which the BODIPY tags are grafted in ABPs **1** and **2** and which is designed to occupy the space normally occupied by the substrate fucoside.

**Fig. 7:**
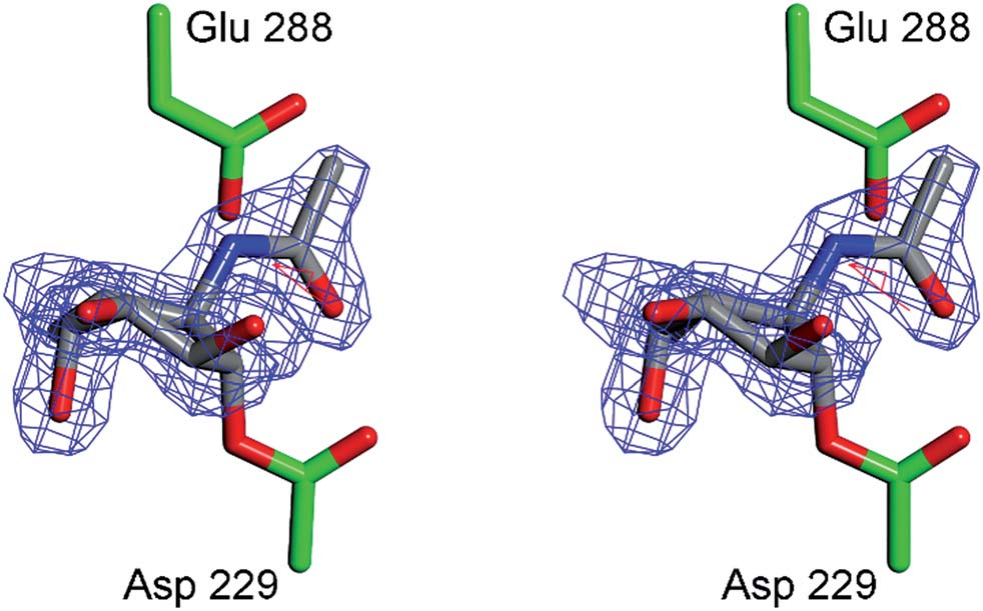
Crystal structure of α-l-fucosidase from *Bacteroides thetaiotaomicron* in complex with **5**. Catalytic residues are annotated: Asp 229 (nucleophile) and Glu 288 (acid/base). Electron density displayed is *F*_o_–*F*_c_ density from phases calculated prior to the inclusion of **5** in refinement, contoured at 3*σ*. Figure was prepared using CCP4MG.^29^

In our design of aziridine-based retaining *exo*-glycosidase ABPs we made the assumption that such aglycon-like moieties would not interfere with enzyme binding (this also based on the numerous fluorogenic substrates that are in use to study exoglycosidases and in which the aglycon moiety can take on made shapes and sizes). The 3-D fold of *Bt*Fuc2970, however, appears not to provide sufficient space to accommodate an extended aryl aglycon pendant to the atom, which would equate to the ring oxygen in fucose. In order to minimize steric clashes, *Bt*Fuc2970 was subsequently incubated with **5**. The crystal structure of the resulting complex (PDB code ; 4WSJ) revealed unambiguous electron density for the presence of a covalent enzyme–**5** complex (Fig. 7). The complex has C–O bond lengths between the *Bt*Fuc2970 catalytic nucleophile and **5** of *ca.* 1.43 Å as would be expected for a C–O ester bond. As expected from the reduction in aglycon size, **5** is better ordered than **4** when bound to the bacterial enzyme and provides a clearer definition of the resulting conformation and interactions. Upon *trans*-diaxial opening of the acylaziridine the covalently bound and substituted cyclohexane adopts a slightly distorted ^3^H_4_ conformation (between ^3^H_4_ and ^3^S_1_); consistent with the expected catalytic itinerary.^28^

## Conclusions

We have developed the potent and selective aziridine-based ABPs **1**, **2** and **3** for selective profiling of active GH29 α-l-fucosidases in cell extracts from bacteria, mice and man as well as *in vivo* in mice. Labeling of GH29 retaining α-l-fucosidases with our l-fucopyranose-configured, cyclophellitol aziridine-based ABPs proceeds with good efficiency and high selectively both *in vitro* and *in vivo* with the single caveat that the probes do not penetrate brain tissue in mice. The covalent irreversible aziridine inhibitors proved much more potent than their iminosugar counterparts, of which l-fuconojirimycin **6** appeared to be the single compound from this set of configurational isomers that is able to inhibit FUCA1 in a competitive ABPP setting. The crystal structures of α-l-fucosidase from *Bacteroides thetaiotaomicron* in complex with compound **4** and **5** provide strong evidence for the covalent binding of cyclophellitol aziridine to active α-l-fucosidases and by this virtue the validity of the cyclophellitol aziridine design for activity-based profiling of retaining glycosidases that employ the Koshland double displacement mechanism. Whereas aziridines and epoxides that are annulated to cyclohexane rings preferably open in a *trans*-diaxial fashion through a chair-like transition state, reaction with the α-l-fucosidase nucleophile takes place at the aziridine carbon corresponding to the anomeric center of a substrate α-l-fucoside. This corresponds to ring opening to yield a skew boat, as is observed in the trapped enzyme active site in the co-crystal.

In conclusion, we offer ABPs **1**, **2** and **3** as reagents for the discovery and annotation of new members of the GH29 family of α-l-fucosidase in comparative ABPP experiments, for monitoring retaining α-l-fucosidase activities in health and disease, and for the discovery of inhibitors able to interfere with specific α-l-fucosidases in competitive ABPP experiments. ABPs **1**, **2** and **3** add to our growing series of *in situ* and *in vivo* active retaining glycosidase ABPs and moreover their design hold promise for the design of ABPs targeting retaining glycosidases recognizing and processing differently configured and substituted carbohydrates.

## Experimental section

### Synthesis

The synthesis, analytical and spectroscopical analysis of l-fuconojirimycin configured cyclophellitol aziridines **1–5** and l-fuconojirimycin isomers **6–13** is described in the ESI.†

### Biological assays

#### Materials

Cyclophellitol β-aziridine ABP JJB75 was synthesized as described earlier.^18^ Gaucher patients were diagnosed on the basis of reduced GBA activity and demonstration of an abnormal genotype.^30^ Cell lines were cultured in HAMF12-DMEM medium (Invitrogen) supplied with 10% (v/v). Spleens from a normal subject and a patient suffering from type 1 Gaucher were collected after splenectomy and immediately stored frozen.

#### Molecular cloning and recombinant expression

The coding sequences of *H. sapiens* FUCA1 (NCBI reference sequence XM_005245821.1, using forward primer 5′-GGGGACAAGTTTGTACAAAAAAGCAGGCTCCACCACCATGCGGGCTCCGGGGATG-3′ and reverse primer 5′-GGGGACCACTTTGTACAAGAAAGCTGGGTCTTACTTCACTCCTGTCAGCTTTAT-3′), and of *H. sapiens* FUCA2 (NCBI reference sequence NM_032020.4, using forward primer 5′-GGGGACAAGTTTGTACAAAAAAGCAGGCTC CACCACCATGCGGCCCCAGGAGCTC-3′ and reverse primer 5′-GGGGACCACTTTGTACAAGAAAGCTGGGTCT TAGATCACATTAGTCAGGGCTA-3′) were amplified *via* PCR and cloned into pDNOR-221 and thereafter sub-cloned in pcDNA™-DEST40 vector using the Gateway system (Invitrogen). Correctness of all constructs was verified by sequencing. Confluent COS-7 cells were transfected with pcDNA3.1 empty vector (Mock) or the vector with described insert in conjunction with FuGENE (Roche). After 72 hours, medium isolated and frozen at –80 °C and cells were harvested by scraping in 25 mM potassium phosphate buffer (pH 6.5, supplemented with 0.1% (v/v) Triton X-100 and protease inhibitor cocktail (Roche)). After determination of the protein concentration (BCA kit, Pierce), lysates were aliquoted and frozen at –80 °C.

#### Enzyme activity assays

The enzymatic activity of α-l-fucosidase was assayed at 37 °C by incubating with 1.5 mM 4-methylumbelliferyl-α-l-fucopyranoside as substrate in 150 mM McIlvaine buffer, pH 4.5, supplemented with 0.1% (w/v) BSA. To determine the apparent IC_50_ value, COS-7 cell lysate containing over-expressed human recombinant FUCA1 was pre-incubated with a range of inhibitor dilutions for 30 min at 37 °C. The reaction was quenched by adding excess NaOH–glycine (pH 10.6), after which fluorescence was measured with a fluorimeter LS55 (Perkin Elmer) using *λ*_EX_ 366 nm and *λ*_EM_ 445 nm.

#### *In vitro* labeling and SDS-PAGE analysis

All pre-incubations and ABP labeling-reactions occurred for 30 min at 37 °C. Total lysates (50 μg), medium (500 μg) or purified protein preparations (5 μg) were labeled with 1 μM JJB256 **1** or JJB244 **2**, dissolved in 150 mM McIlvaine buffer, pH 4.5, incubating for 30 min at 37 °C. For ABPP, protein preparations were pre-incubated with compounds **4** (100 μM), **6** (100 μM), **7–13** (1 or 5 mM, specified in the main text) prior to the addition of 100 nM fluorescent ABPs. Influence of pH on ABP labeling involved pre-incubation at pH 3–10 prior to addition of 100 nM ABP **1**. Direct labeling of retaining β-glucosidases occurred at pH 5.0 in conjunction with 100 nM ABP JJB75. Samples were then denatured with 5× Laemmli buffer (50% (v/v) 1 M Tris–HCl, pH 6.8, 50% (v/v) 100% glycerol, 10% (w/v) DTT, 10% (w/v) SDS, 0.01% (w/v) bromophenol blue), boiled for 4 min at 100 °C, and separated by gel electrophoresis on 10% (w/v) SDS-PAGE gels running continuously at 90 V.^13,14^ Wet slab-gels were scanned on fluorescence using a Typhoon Variable Mode Imager (Amersham Biosciences) using *λ*_EX_ 488 nm and *λ*_EM_ 520 nm (band pass filter 40 nm) for green fluorescent JJB256 **1** and *λ*_EX_ 532 nm and *λ*_EM_ 610 nM (band pass filter 30 nm) for red fluorescent JJB244 **2** and JJB75.

#### *In vivo* labeling

The appropriate ethics committee for animal experiments approved all experimental procedures. Wild-type C57Bl/6J male mice were obtained from Harlan and fed a commercially available lab diet (RMH-B; Hope Farms). Four C57BL/6J mice were injected intraperitoneally with 100 μL sterile PBS (vehicle) or PBS containing 10, 100, or 1000 pmol of ABP **1** (about 0.2 μg kg^–1^, 2 μg kg^–1^, and 20 μg kg^–1^, respectively). At 2 h post-administration, urine was collected and the mice were anesthetized with FFM mix (25/25/50 fentanylcitrate/midazalam/H_2_O), blood was collected and perfused with PBS at 3.0 mL min^–1^. Then urine and organs were collected and directly frozen in liquid nitrogen. Homogenates were made in 25 mM potassium phosphate buffer, pH 6.5, supplemented with 0.1% (v/v) Triton X-100 and protease inhibitor cocktail (Roche). After determination of protein concentration (BCA kit, Pierce), 50 μg total protein was incubated with 100 nM red fluorescent JJB75 and analyzed by SDS-PAGE. As controls, matching tissue homogenates of vehicle-treated animals were concomitantly labeled with 1 μM ABP **1** and 100 nM JJB75 prior to SDS-PAGE.

#### ABP pulldown and LC-MS/MS analysis

Gaucher spleen lysate (1 g) was cut with a sterile scalpel, mixed with 4 mL 25 mM potassium phosphate buffer (pH 6.5, supplemented with 0.1% (v/v) Triton X-100 and protease inhibitor cocktail (Roche)) and sonicated three times for 10 seconds at 100% strength and kept on ice. After 2 min, procedure repeated twice. Lysate was then centrifuged for 10 min at 10 000 *g*, supernatant collected carefully, protein concentration determined and 200 mg total protein was then incubated with either 0.1% (v/v) DMSO, ABP **1** or ABP **3**, or firstly with 10 μM ABP **1** followed by 10 μM of ABP **3**, each step taking 30 min at 37 °C, in a total volume of 1 mL McIlvaine buffer, pH 4.5. Glycosylated biomolecules were enriched by using 1 mL ConA-Sepharose per sample, according to manufacturer's instructions (Amersham Pharmacia Biotech AB, Sweden) and subsequently denatured by the presence of 2% (w/v) SDS and boiling for 5 min at 100 °C. From here on, samples were prepared for MS as published earlier.^14^ After desalting on StageTips, peptides were analyzed with a 2 h gradient of 5–25% ACN on nano-LC, hyphenated to an LTQ-Orbitrap and identified *via* the Mascot protein search engine.^14^

#### X-ray crystallography

Recombinant *Bacteroides thetaiotaomicron* 2970 α-l-fucosidase (*Bt*Fuc2970) was prepared as described previously.^28^ Protein crystals were obtained through hanging (Crystals soaked with compound **5**) or sitting (crystals soaked with compound **4**) drop vapor diffusion (for further details see PDB file headers). Compounds **4** and **5** were dissolved in crystallization mother liquor at a concentration of **5** or 20 mM respectively and added to crystallization drops containing crystals of *Bt*Fuc2970 in a 1 : 1 ratio. After *ca.* 1 h soaking with ligands, crystals were fished into cryo-protectant solutions (mother liquor supplemented with 20% v/v glycerol) and cryo-cooled in liquid nitrogen. Diffraction data were collected at Diamond Light Source. Diffraction images were indexed and integrated using MOSFLM^29^ (**4**) or XDS^31^ (**5**) and scaled and merged using AIMLESS.^32^ Crystals grew in an almost isomorphous space group to PDB entry ; 4JFV, and coordinates from this entry were used directly to obtain a starting model for refinement. Iterative stages of model-building (COOT^33^) and maximum-likelihood refinement (REFMAC5 (ref. 34)) were conducted to yield final models. Maximum-likelihood restraints for compounds **4** and **5** were created using the PRODRG online server^35^ and link restraints generated using JLIGAND.^36^ X-ray crystallographic data statistics are available in ESI Table S3.†

## Supplementary Material

Supplementary information
