# Supplementary material for: In vitro and in vivo comparative and competitive activity-based protein profiling of GH29 α-l-fucosidases
Source: Chem Sci. 2015 Feb 9;6(5):2782–9. doi: 10.1039/c4sc03739a (PMC5654414; doi:10.1039/c4sc03739a)

Electronic Supplementary Material (ESI) for Chemical Science.
This journal is © The Royal Society of Chemistry 2015

Supporting Information

In vitro and *In vivo* Comparative and Competitive Activity-based Protein Profiling of GH29 α-L-Fucosidases

Jianbing Jiang,[a,†] Wouter W. Kallemeijn,[b,†] Daniel W. Wright,[c] Adrianus M. C. H. van den Nieuwendijk,[a] Veronica Coco Rohde, [a] Elisa Colomina Folch, [a] Hans van den Elst, [a] Bogdan I. Florea, [a] Saskia Scheij, [b] Wilma E. Donker-Koopman, [b] Marri Verhoek, [b] Nan Li, [a] Martin Schürmann,[d] Daniel Mink, [d] Rolf G. Boot, [b] Jeroen D. C. Codée, [a] Gijsbert A. van der Marel, [a] Gideon J. Davies, [c] Johannes M. F. G. Aerts*[,a,b] and Herman S. Overkleeft.*[,a]

[a] Leiden Institute of Chemistry, Leiden University, P.O. Box 9502, 2300 RA Leiden, The Netherlands.

[b] Department of Medical Biochemistry, Academic Medical Center, Meibergdreef 15, 1105 AZ Amsterdam, The Netherlands.

[c] Department of Chemistry, University of York, Heslington, York, YO10 5DD, United Kingdom.

[d] DSM Innovative Synthesis, Urmonderbaan 22, NL-6167 RD Geleen, The Netherlands.

Table of contents

1. General synthetic methods.......S2
2. Synthesis of compounds 1 – 5.......S2
3. Synthesis of compounds 28 and 29.......S7
4. Preparation of fuconojirimycin 6 and the configurational isomers 7 – 13.......S10
5. Biological assays.......S21
5.1 Fig. S1. Bacterial lysate with ABP **1**.......S21
5.2 Fig. S2. FUCA1, FUCA2, COS cell, Murine tissues lysate with ABP **1**.......S21
5.3 Fig. S3. Structure of JJB75.......S21
5.4 Fig. S4. pH optimum.......S22
5.5 Fig. S5. Kinetics detection.......S22
5.6 Fig. S6. *In vivo* labeling of full gel with JJB75 and ABP **1**.......S23
5.7 Table S1.Identification of biotinylated proteins by LC-MS/MS analysis.......S23
5.8 Table S2.Identification of peptide fractions from FUCA1.......S25
5.9 Fig. S7. Crystal structure of complex of **4**-enzyme.......S25
5.10 Table S3. X-ray crystallographic data statistics.......S26
6. References.......S27
^{1}H and ^{13}C NMR spectra.......S28

1. General synthetic methods

All reagents were of a commercial grade and were used as received unless stated otherwise. Dichloromethane (DCM), tetra-hydrofuran (THF) and *N,N*-dimethylformamide (DMF) were stored over 4 Å molecular sieves, which were dried *in vacuo* before use. All reactions were performed under an argon atmosphere unless stated otherwise. Solvents used for flash column chromatography were of pro analysis quality. Reactions were monitored by TLC analysis using Merck aluminium sheets pre-coated with silica gel 60 with detection by UV absorbtion (254 nm) and by spraying with a solution of $(NH_4)_6Mo_7O_{24}$•H_2O (25 g/L) and $(NH_4)_4Ce(SO_4)_4$•H_2O (10 g/L) in 10% sulfuric acid followed by charring at ~150˚C or by spraying with 20% sulfuric acid in ethanol followed by charring at ~150 ℃. Column chromatography was performed using either Baker - or Screening Device silica gel 60 (0.04-0.063mm) in the indicated solvents. ^{1}H NMR and ^{13}C NMR spectra were recorded on a Bruker DMX-600 (600/150 MHz) and a Bruker AV-400 (400/100 MHz) spectrometer in the given solvent. Chemical shifts are given in ppm relative to the chloroform residual solvent peak or tetramethylsilane (TMS) as internal standard. Coupling constants are given in Hz. All given ^{13}C spectra are proton decoupled. High-resolution mass spectra were recorded with a LTQ Orbitrap (Thermo Finnigan). FT-IR spectra were recorded on a Shimadzu FT-IR 83000 spectrometer. LC/MS analysis was performed on a Jasco HPLC-system (detection simultaneously at 214nm and 254nm) equipped with buffers A: H_2O, B: acetonitrile (MeCN) and C: 10% 0.5M NH_4OAc, and coupled to a Perkin Elmer Sciex API 165 mass instrument. For reversed-phase HPLC purifications an Agilent Technologies 1200 series instrument equipped with a semi preparative Gemini C18 column (10 x 250 mm) was used. The applied buffers were A: H_2O, B: MeCN.

2. Synthesis of compounds 1 – 5. (*Scheme 1, main paper*)

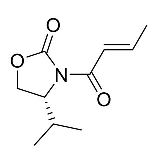

(*R,E*)-3-(But-2-enoyl)-4-isopropyloxazolidin-2-one (14): Compound **14** was prepared from Boc-D-Valine via the strategy reported by Evans[1] *et. al.* for its enantiomer, giving compound **14** (3.56 g, 18.0 mmol, 38% over three steps) as a yellow oil. TLC: R_f = 0.59 (Pentane/EtOAc, 1/1, v/v); $[\alpha]_D^{20}$ = -104 (*c* = 1, $CHCl_3$); lit[1] for enantiomer: $[\alpha]_D^{20}$ = 105 (*c* = 1.97, $CHCl_3$); HRMS: Calculated for $[C_{10}H_{15}NO_3 + H]^+$ 198.11247, found: 198.11224; ^{1}H NMR (400 MHz, $CDCl_3$) δ 7.28 (d, *J* = 15.8 Hz, 1H), 7.20-7.11 (m, 1H), 4.51-4.47 (m, 1H), 4.29 (dd, *J* = 9.2, 8.4 Hz, 1H), 4.22 (dd, *J* = 9.2, 3.2 Hz,, 1H), 2.48 – 2.27 (m, 1H), 1.96 (d, *J* = 6.7 Hz, 4H), 0.93 (d, *J* = 7.2 Hz, 3H); 0.89 (d, *J* = 7.2 Hz, 3H); ^{13}C NMR (101 MHz, $CDCl_3$) δ 165.01, 154.13, 146.71, 121.92, 63.39, 58.55, 28.51, 18.56, 18.06, 14.72. IR(neat, cm^{-1}): 2965, 1773, 1684, 1638, 1389, 1364, 1233, 1202, 1119, 1061, 1036, 970, 926, 754, 714.

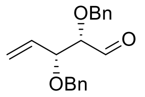

(2*R*,3*S*)-2,3-Bis(benzyloxy)pent-4-enal (14): Building block **15** was prepared from L-(-)-xylose by the reported strategy[2a] of Hansen *et. al.* for its enantiomer. Compound **15** was obtained as a clear oil (6.14 g, 20.7 mmol, overall yield 47%). TLC: R_f = 0.45 (Pentane/EtOAc, 5/1, v/v); $[\alpha]_D^{20}$ = -88 (*c* = 1, $CHCl_3$); lit[2] for enantiomer: $[\alpha]_D^{20}$ = 68.7 (*c* = 1, $CHCl_3$); HRMS: Calculated for $[C_{19}H_{20}O_3 + H]^+$ 297.14852, Found 297.14922; ^{1}H NMR (400 MHz, $CDCl_3$) δ 9.66 (d, *J* = 7.6 Hz, 1H), 7.41 – 7.20 (m, 10H), 5.92 (ddd, *J* = 17.6, 10.5, 7.6 Hz, 1H), 5.44 – 5.22 (m, 2H), 4.73 (d, *J* = 12.1 Hz, 1H), 4.60 (apparent t, *J* = 12.0 Hz, 2H), 4.33 (d, *J* = 12.1 Hz, 1H), 4.15 (dd, *J* = 7.7, 4.1 Hz, 1H), 3.81 (d, *J* = 4.1 Hz, 1H); ^{13}C NMR (101 MHz, $CDCl_3$) δ 202.38, 137.53, 137.06, 133.79, 128.46, 128.35, 128.12, 128.08, 127.90, 127.75, 119.84, 85.11, 79.86, 73.39, 70.61. IR(neat, cm^{-1}): 2862, 1732, 1454, 1207, 1069, 1028, 934, 737, 696.

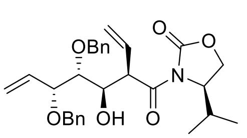

(*R*)-3-((2*R*,3*R*,4*R*,5*R*)-4,5-Bis(benzyloxy)-3-hydroxy-2-vinylhept-6-enoyl)-4-isopropyloxazolidin-2-one (16): The oxazolidinone **14** (2.50 g, 12.6 mmol) was dissolved in anhydrous CH_2Cl_2 (20.0 mL). After addition of a solution of 1.0 M dibutylboryltrifluoromethanesulfonate (DBBT) in anhydrous CH_2Cl_2 (12.6 mL, 12.6 mmol) at -78 ℃, the resulting dark green mixture was removed from the cold bath to dissolve any frozen triflate and cooled again to -78 °C. Triethylamine (TEA) (2.07 mL, 14.5 mmol) was added subsequently, causing the dark green color to fade. The solution was stirred for 50 minutes at -78 ℃ and then at 0 ℃ for 15 minutes (the solution turned yellow). While the reaction mixture was being cooled back down to -78 ℃, a solution of aldehyde **15** (3.35 g, 11.4 mmol) in anhydrous CH_2Cl_2 (20.0 mL) was added to the reaction mixture via a syringe. The temperature was slowly raised to -20 ℃ over one hour and then maintained at this temperature for an additional hour. The resulting yellow solution was then stirred at -15 ℃ for another hour and then warmed to -5 ℃ and quenched with a phosphate buffer (pH 7) solution (25 mL). A 30% H_2O_2 solution was then added dropwise while maintaining the internal temperature below 5 ℃. Addition of the peroxide was continued until the internal temperature remained constant. The mixture was stirred

for an additional 45 minutes while slowly warming to room temperature. The reaction was then poured into aqueous saturated $NaHCO_3$ (100 mL) and the aqueous layer extracted with CH_2Cl_2 (3 x 50 mL). The combined organic layers were dried over $MgSO_4$, filtered, and concentrated under reduced pressure. The crude product was purified by silica gel column chromatography (2-20% EtOAc in pentane) giving product **16** as colorless oil (4.41 g, 8.90mmol, 71%). TLC: R_f = 0.47 (Pentane /EtOAc, 3/1, v/v); $[\alpha]_D^{20}$ = +24 (c = 1, $CHCl_3$); HRMS: Calculated for $[C_{29}H_{35}NO_6 + H]^+$; 494.25371. Found: 494.25344; ^{1}H NMR (400 MHz, CDCl3) δ 7.37 – 7.20 (m, 10H), 6.08-6.00 (m, 1H), 5.96 – 5.82 (m, 1H), 5.42 (m, 2H), 5.37 (m, 1H), 5.28 (d, J = 9.9 Hz, 1H), 4.99 (dd, J = 8.4, 7.6 Hz, 1H), 4.68 (d, J = 11.5 Hz, 2H), 4.55 – 4.33 (m, 3H), 4.28 (dd, J = 7.2, 3.9 Hz, 1H), 4.07 – 3.95 (m, 1H), 3.83 (dd, J = 8.9, 3.1 Hz, 1H), 3.57 (dd, J = 8.3, 3.9 Hz, 1H), 3.35 (d, J = 2.1 Hz, 1H), 3.24 (t, J = 8.8 Hz, 1H), 2.21 (m, 1H), 0.77 (d, J = 7.0 Hz, 3H), 0.72 (d, J = 6.9 Hz, 3H); ^{13}C NMR (101 MHz, $CDCl_3$) δ 172.58, 153.62, 138.06, 137.76, 134.48, 133.61, 128.53, 128.34, 128.00, 127.91, 127.66, 127.42, 120.58, 119.37, 81.75, 79.88, 73.04, 71.23, 70.73, 62.45, 58.04, 50.21, 28.02, 17.88, 14.49. IR (neat, cm^{-1}): 3503, 2963, 1776, 1697, 1385, 1371, 1300, 1202, 1099, 1061, 928, 739, 698.

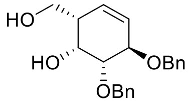

(1*R*,2*S*,5*R*,6*R*)-5,6-Bis(benzyloxy)-2-(hydroxymethyl)cyclohex-3-en-1-ol (17): The product **16** (4.41 g, 8.90 mmol) was dissolved in a mixture of THF (65 mL) and H_2O (3.30 mL). Next, $LiBH_4$ (2 M solution in THF, 26 mL, 52 mmol) was added at 0 ℃. After stirring at 0 ℃ for one hour, the reaction mixture was warmed to room temperature and stirring was continued for one hour. The reaction was quenched with aqueous 2M NaOH (50 mL) and diluted with Et_2O (50 mL). After stirring for five minutes the reaction mixture was extracted with Et_2O (100 mL), and the separated organic phase was washed with aqueous saturated $NaHCO_3$ (20 mL) and brine (100 mL), dried over $MgSO_4$, filtered, and concentrated under reduced pressure to give the crude alcohol that was purified by silica gel column chromatography (10-50% EtOAc in pentane) giving the intermediate primary alcohol as a white solid (2.72 g, 7.38 mmol) that was dissolved in CH_2Cl_2 (260 mL). After addition of the second generation Grubbs catalyst (313 mg, 0.37 mmol, 0.05 eq.), the mixture was stirred at 40 ℃ in the dark for 24 h. DMSO (0.50 mL) was next added, and the solution was stirred at room temperature for another 3 h. The solvent was evaporated under reduced pressure to give a crude mixture, which was purified by silica gel column chromatography (20-50% EtOAc in pentane) giving product **17** as a black solid (2.36g, 6.90mmol, 78%). TLC: R_f = 0.41 (Pentane /EtOAc, 3/2, v/v); $[\alpha]_D^{20}$ = -147 (c = 1, $CHCl_3$); HRMS: Calculated for $[C_{21}H_{24}O_4 + H]^+$ 341.17474, Found: 341.17486; ^{1}H NMR (400 MHz, $CDCl_3$) δ 7.41 – 7.25 (m, 10H), 5.86 (dt, J = 10.2, 2.8 Hz, 1H), 5.58 (d, J = 9.7 Hz, 1H), 4.80 – 4.66 (m, 4H), 4.38 (s, 1H), 4.35 – 4.27 (m, 1H), 3.92 – 3.76 (m, 2H), 3.68 (dd, J = 7.8, 2.2 Hz, 1H), 2.65 (m, 1H), 2,60 (s, 1H), 2.50 (m, 1H); ^{13}C NMR (101 MHz, $CDCl_3$) δ 138.64, 138.14, 128.66, 128.53, 128.07, 128.01, 127.94, 127.78, 127.64, 126.97, 81.88, 76.69, 72.40, 72.36, 70.47, 63.88, 41.97. IR(neat, cm^{-1}) 3422, 2872, 1454, 1206, 1090, 1051, 1026, 860, 801, 733, 696.

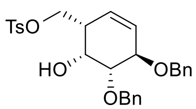

((1*S*,4*R*,5*R*,6*R*)-4,5-Bis(benzyloxy)-6-hydroxycyclohex-2-en-1-yl)methyl 4-methylbenzenesulfonate (18): A solution of **17** (1.73 g, 5.09 mmol, 1.00 eq.) in anhydrous CH_2Cl_2 (40 mL), containing Et_3N (1.75 mL, 12.7 mmol, 2.50 eq.) was cooled to 0 ℃ and treated with p-TsCl (2.18 g, 11.2 mmol, 2.2 eq.). The reaction mixture was stirred at room temperature for 3 h, followed by extra addition of Et_3N (0.80 mL, 5.70 mmol, 1.1 eq.) and p-TsCl (1.00 g, 5.00 mmol, 1.00 eq.). After TLC confirmed full conversion of the starting material, the reaction was diluted with water and extracted with CH_2Cl_2. The organic layer was washed with brine and dried over $MgSO_4$. The solvent was removed under reduced pressure and the residue was purified by silica gel column chromatography (5-35% EtOAc in pentane) giving product **18** as a pale yellow solid (2.18 g, 4.42 mmol, 87%). TLC: R_f = 0.30 (Pentane /EtOAc, 3/1, v/v); $[\alpha]_D^{20}$ = -133 (c = 1, $CHCl_3$); HRMS: Calculated for $[C_{28}H_{30}O_6S + H]^+$ 495.18359, Found: 495.18300; ^{1}H NMR (400 MHz, $CDCl_3$) δ 7.77 (d, J = 8.0 Hz, 2H), 7.39 - 7.23 (m, 12H), 5.78 (dt, J = 10.4, 2.4 Hz, 1H), 5.34 (d, J = 9.5 Hz, 1H), 4.69 - 4.62 (m, 4H), 4.24 (m, 1H), 4.16 (m, 2H), 4.00 (dd, J = 7.2, 6.8 Hz, 1H), 3.57 (dd, J = 7.6, 2.0 Hz, 1H), 2.67 (m, 1H), 2.51 (broad s, 1H), 2.39 (s, 3H); ^{13}C NMR (101 MHz, $CDCl_3$) δ 144.89, 138.43, 137.93, 132.65, 129.88, 128.47, 128.45, 128.36, 127.92, 127.86, 127.80, 127.62, 123.64, 81.72, 76.57, 72.31, 72.17, 69.84, 66.83, 40.25, 21.64. IR(neat, cm^{-1}) : 3032, 2872, 1597, 1497, 1454, 1358, 1175, 1096, 964, 787, 698, 664.

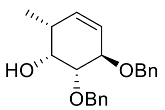

(1*R*,2*R*,5*R*,6*R*)-5,6-Bis(benzyloxy)-2-methylcyclohex-3-en-1-ol (19): Compound **18** (2.19 g, 4.42 mmol, 1.00 eq.) was dissolved in dry THF (18 mL) at 0 ℃. A solution of $LiAlH_4$ (2M in THF)(3.32 mL, 6.63 mmol, 1.50 eq.) was added dropwise. The reaction mixture was stirred at room temperature for 3h, diluted with Et_2O and quenched with dropwise addition of saturated aqueous NaCl. The solid material was removed by filtration and the residue washed thoroughly 3 times with hot EtOAc. The filtrate was dried over $MgSO_4$, filtered again and the solvents removed under reduced pressure. The crude product was purified by silica gel column chromatography (10-20% EtOAc in pentane) giving product **19** as yellow oil (1.24 g, 3.83 mmol, 87%). TLC: R_f = 0.53 (Pentane/EtOAc, 3/1, v/v); $[\alpha]_D^{20}$ = -121 (c = 1, $CHCl_3$);

HRMS: Calculated for $[C_{21}H_{24}O_3 + H]^+$ 325.17982, Found: 325.17995; ^{1}H NMR (400 MHz, $CDCl_3$) δ 7.41 – 7.20 (m, 10H), 5.71 (dt, *J* = 10.1, 2.7 Hz, 1H), 5.48 – 5.40 (m, 1H), 4.76 – 4.62 (m, 4H), 4.31 (dq, *J* = 7.6, 2.6 Hz, 1H), 4.06 (broad s, 1H), 3.67 (dd, *J* = 7.7, 2.2 Hz, 1H), 2.45-2.39 (m, 1H), 2.25 (broad s, 1H), 1.12 (d, *J* = 7.5 Hz, 3H); ^{13}C NMR (101 MHz, $CDCl_3$) δ 138.77, 138.37, 131.27, 128.54, 128.44, 127.91, 127.90, 127.86, 127.64, 125.26, 82.73, 76.99, 72.28, 72.09, 70.90, 35.00, 16.36. IR(neat, cm^{-1}) : 3456, 2872, 1497, 1454, 1207, 1090,1057, 980, 785, 735, 696.

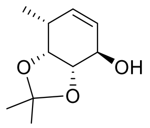

(3a*S*,4*R*,7*R*,7a*R*)-2,2,7-trimethyl-3a,4,7,7a-tetrahydrobenzo[d][1,3]dioxol-4-ol (20): Ammonia (50 mL) was condensed at -60 ℃. Lithium (525 mg, 75.6 mmol, 10.0 eq.) was added and the mixture was stirred until the lithium was completely dissolved. To this solution was added a solution of cyclohexene **19** (2.45 g, 7.56 mmol, 1.00 eq.) in dry THF(60 mL). The reaction mixture was stirred for 30 minutes at -60 ℃ and subsequently quenched with water (10 mL). The resulting solution was allowed to come to room temperature and stirred until all ammonia had evolved. Then the solution was concentrated under reduced pressure, re-dissolved in water and neutralized with Amberlite H^+. The resin was removed by filtration, and the filtrate was concentrated under reduced pressure. The crude product was purified by silica gel column chromatography (5-20% MeOH in CH_2Cl_2) giving a white crystaline product (1.01 g, 7.00 mmol) which was dissolved in 2,2-dimethoxypropane (70 mL) and cooled to 0 ℃. A catalytic amount of D-(+)-10-camphorsulfonic acid (162 mg, 0.70 mmol, 0.10 eq.) was added and the mixture was stirred at 0 ℃ for 2h. TLC analysis showed complete conversion and the mixture was diluted by MeOH/H_2O (v/v = 9/1, 50 mL) and stirred at room temperature for 30 minutes. The reaction mixture was neutralized with Et_3N, concentrated under reduced pressure, extracted with CH_2Cl_2, washed with brine, dried over $MgSO_4$, filtered and concentrated. After purification by silica gel column chromatography (0-8% MeOH in CH_2Cl_2) product **20** was obtained as a pale yellow oil (870 mg, 4.73 mmol, 63%). TLC: R_f = 0.40 (DCM/MeOH, 10/1, v/v); $[\alpha]^{20}_{D}$ = -126 (c = 1, MeOH); HRMS: Calculated for $[C_{10}H_{16}O_3 + H]^+$ 185.11722, Found: 185.11714; ^{1}H NMR (400 MHz, $CDCl_3$): δ 5.93-5.89 (m, 1H), 5.76 (dd, *J* = 10.0, 3.6 Hz, 1H), 4.42 - 4.39 (m, 1H), 4.28-4.24 (m, 2H), 2.69-2.66 (m, 1H), 1.79 (broad s, 1H), 1.40 (s, 3H), 1.35 (s, 3H), 1.15 (d, *J* = 7.4 Hz, 3H); ^{13}C NMR (101 MHz, $CDCl_3$) δ 135.50, 128.09, 108.20, 80.17, 76.46, 67.80, 31.11, 26.85, 24.91, 16.12. IR(neat, cm^{-1}): 3345, 2913, 2699, 1161, 1084, 1053, 986, 858, 783.

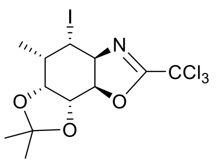

(3a*R*,4*S*,5*S*,5a*S*,8a*R*,8b*R*)-5-iodo-2,2,4-trimethyl-7-(trichloromethyl)-3a,4,5,5a,8a,8b-hexahydro-[1,3]dioxolo[4',5':3,4]benzo[1,2-d]oxazole (21): Compound **20** (870 mg, 4.73 mmol, 1.00 eq.) was dissolved in anhydrous CH_2Cl_2 (70 mL). The solution was cooled to 0 ℃ and treated with trichloroacetonitrile (946 μL, 9.46 mmol, 2.00 eq.) and 1,8-diazobicyclo[5.4.0]undec-7-ene (68.0 μL, 0.47 mmol, 0.1 eq.). After 2h stirring at 0 ℃, TLC analysis revealed complete conversion to a higher running product. To the resulting solution was added water (18 mL), $NaHCO_3$ (3.96 g, 47.0 mmol, 10.0 eq.) and iodine (4.32 g, 17.0 mmol, 3.50 eq.). The reaction mixture was stirred overnight at room temperature before being quenched with aqueous 10% $Na_2S_2O_3$ solution and extracted three times with EtOAc. The organic layer was dried over $MgSO_4$, filtered, concentrated under reduced pressure and the residue purified by silica gel column chromatography (0-8% EtOAc in pentane) giving product **21** as brown oil (980 mg, 2.16 mmol, 46%). TLC: R_f = 0.49 (Pentane /EtOAc, 10/1, v/v); $[\alpha]^{20}_{D}$ = +34 (*c* = 1, $CHCl_3$). HRMS: Calculated for $[C_{12}H_{15}Cl_3INO_3 + H]^+$ 453.92350, Found: 453.92347; ^{1}H NMR (400 MHz, $CDCl_3$) δ 5.12 (dd, *J* = 10.2, 4.1 Hz, 1H), 4.89 (dd, *J* = 10.2, 7.0 Hz, 1H), 4.45 (dd, *J* = 8.1, 5.7 Hz, 1H), 4.35 (dd, *J* = 8.1, 4.0 Hz, 1H), 4.12 (dd, *J* = 7.0, 3.0 Hz, 1H), 2.11-2.07 (m, 1H), 1.58 (s, 3H), 1.32 (s, 3H), 1.17 (d, *J* = 7.0 Hz, 3H); ^{13}C NMR (101 MHz, $CDCl_3$) δ 162.43, 109.70, 84.10, 74.88, 73.75, 73.30, 35.47, 26.11, 25.09, 23.93, 14.98. IR(neat, cm^{-1}) : 2982, 1661, 1381, 1207, 1067, 1045, 988, 953, 837, 791, 665, 652.

(1*R*,2*R*,3*R*,4*R*,5*R*,6*R*)-5-methyl-7-azabicyclo[4.1.0]heptane-2,3,4-triol (22): Compound **21** (980 mg, 2.16 mmol, 1.00 eq.) was dissolved in MeOH (32 mL). The solution was treated with concentrated HCl (8.00 mL) at 60 ℃ overnight. LC/MS analysis showed complete conversion. The solution was concentrated under reduced pressure and re-dissolved in MeOH (30 mL), $NaHCO_3$ (3.96 g, 47 mmol, 22 eq.) was added. After stirring at room temperature for 4 days, the reaction mixture was filtered and concentrated under reduced pressure. After purification by silica gel column chromatography (5-20% MeOH in CH_2Cl_2) product **22** was obtained as a colorless oil (225 mg, 1.41 mmol, 65%).TLC: R_f = 0.26 (DCM/MeOH, 5/1, v/v); $[\alpha]^{20}_{D}$ = -97 (*c* = 1, MeOH); HRMS: Calculated for $[C_7H_{13}NO_3 + H]^+$ 160.09682, Found: 160.09711; ^{1}H NMR (400 MHz, CD_3OD) δ 4.07 (dd, J = 8.8, 4.2 Hz, 1H), 3.57-3.56 (m, 1H), 3.35 (d, *J* = 2.3 Hz, 1H), 3.33 (d, *J* = 1.9 Hz, 1H), 2.51 (dd, *J* = 6.3, 4.2 Hz, 1H), 1.95 (d, *J* = 6.3 Hz, 1H), 1.88 (qd, *J* = 7.5, 3.2 Hz, 1H), 1.17 (d, *J* = 7.5 Hz, 3H); ^{13}C NMR (101 MHz, CD_3OD) δ 75.90, 74.39, 70.23, 37.32, 36.92, 36.38, 16.82. IR (neat, cm^{-1}) : 3283, 1456, 1090, 1065, 995, 914, 874, 752.

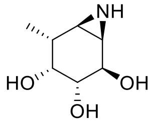

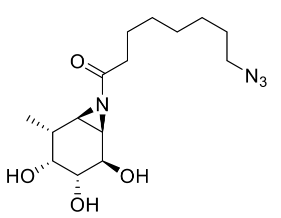

8-Azido-1-((1*R*,2*R*,3*R*,4*R*,5*R*,6*R*)-2,3,4-trihydroxy-5-methyl-7-azabicyclo[4.1.0]heptan-7-yl)-octan-1-one (23): 8-azido-octanoic acid **24**[4] (207 mg, 1.12 mmol, 1.3 eq.) and EEDQ (277 mg, 1.12 mmol, 1.3 eq.) were dissolved in anhydrous DMF (1.10 mL) and stirred at room temperature for 2h. This pre-activated mixed anhydride solution (600 µL, 0.61 eq.) was added to a solution of aziridine **22** (137 mg, 0.86 mmol, 1.0 eq.) in DMF (5 mL) at 0 ℃ and stirred for 30 minutes after which the remaining portion of the pre-activated mixed anhydride solution (500 µL, 0.51 eq.) was added. The resulting mixture was stirred at 0 ℃ for 3h. The reaction was quenched by 2 mL MeOH and the mixture was concentrated *in vacuo*. Then the crude product was purified by silica gel column chromatography (1-10% MeOH in CH_2Cl_2) giving **23** as a colorless oil (162 mg, 0.50 mmol, 58% yield). TLC: R_f = 0.31 (DCM/MeOH, 10/1, v/v); $[\alpha]_D^{20}$ = -29 (*c* = 1, MeOH); LC/MS: R_t 5.35 min, linear gradient 10-90% B in 15 min; ESI-MS: m/z=327.4 $(M+H)^+$; HRMS: Calculated for $[C_{15}H_{26}N_4O_4 + H]^+$ 327.20268, Found: 327.20266; ^1H NMR (400 MHz, CD_3OD) δ 4.06 (dd, *J* = 8.7, 3.9 Hz, 1H), 3.67 – 3.55 (m, 1H), 3.38 (dd, *J* = 8.0, 1.6 Hz, 1H), 3.28 (t, *J* = 6.9 Hz, 2H), 2.96 (dd, *J* = 6.0, 3.6 Hz, 1H), 2.54 (dt, *J* = 15.3, 7.5 Hz, 1H), 2.45 (t, *J* = 7.6 Hz, 1H), 2.42 – 2.36 (m, 1H), 1.99 (qd, *J* = 7.4, 3.3 Hz, 1H), 1.64 – 1.55 (m, 4H), 1.41-1.33 (m, 6H), 1.20 (d, *J* = 7.5 Hz, 3H); ^{13}C NMR (101 MHz, CD_3OD) δ 188.56, 75.71, 74.42, 69.20, 52.36, 43.48, 42.60, 36.94, 36.57, 30.10, 29.90, 29.79, 27.60, 25.83, 16.17. IR (neat, cm^{-1}) : 3402, 2932, 2959, 2093, 1674, 1425, 1258, 1167, 1063, 997, 816.

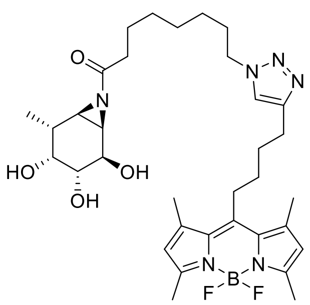

8-(4-(4-(5,5-Difluoro-1,3,7,9-tetramethyl-5H-4l4,5l4-dipyrrolo[1,2-c:2',1'-f][1,3,2]diaza-borinin-10-yl)butyl)-1H-1,2,3-triazol-1-yl)-1-((1*R*,2*R*,3*R*,4*R*,5*R*,6*R*)-2,3,4-trihydroxy-5-methyl-7-azabicyclo[4.1.0]heptan-7-yl)octan-1-one (1): Azide **23** (40 mg, 0.12 mmol) was dissolved in DMF (3 mL), Bodipy compound **25**[4] (44 mg, 0.13 mmol, 1.1 eq.) and aqueous solutions of $CuSO_4$ (1M, 24 µL, 0.024 mmol, 0.2 eq.) and sodium ascorbate (1M, 25 µL, 0.025 mmol, 0.2 eq) were added to the solution under argon atmosphere. The mixture was stirred at room temperature for 2h. The volatiles were removed under reduced pressure and the crude product was purified by semi-preparative reversed HPLC (linear gradient: 44-46% B in A, 12 min, solutions used A: H_2O, B: acetonitrile) and the pure product **1** was obtained as orange powder after lyophilization (9.5 mg, 0.0145 mmol, 12% yield). LC/MS: R_t: 8.58 min; linear gradient 10-90% B in 15 min; ESI-MS: m/z = 655.5 $(M+H)^+$; HRMS: Calculated for $[C_{34}H_{49}BF_2N_6O_4 + H]^+$ 655.39552, Found: 655.39549. ^1H NMR (400 MHz, CD_3OD) δ 7.73 (s, 1H), 6.11 (s, 2H), 4.34 (t, *J* = 6.9 Hz, 2H), 4.05 (dd, *J* = 8.7, 3.9 Hz, 1H), 3.65 – 3.55 (m, 1H), 3.37 (dd, *J* = 8.7, 1.8 Hz, 1H), 3.02 – 2.94 (m, 2H), 2.92 (dd, *J* = 6.0, 3.9 Hz, 1H), 2.77 (t, *J* = 7.3 Hz, 2H), 2.54 – 2.47 (m, 1H), 2.43 (s, 6H), 2.39 – 2.33 (m, 1H), 2.36 (s, 6H), 2.22 (t, *J* = 7.5 Hz, 1H), 2.00-1.93 (m, 1H), 1.92-1.82 (m, 4H), 1.69 – 1.50 (m, 4H), 1.33-1.26 (m, 6H), 1.18 (d, *J* = 7.2 Hz, 3H); ^{13}C NMR (101 MHz, CD_3OD) δ 188.59, 154.91, 148.51, 147.89, 142.20, 132.58, 123.38, 122.61, 75.83, 74.49, 69.27, 51.17, 43.53, 42.66, 36.89, 36.76, 36.67, 32.22, 32.18, 31.16, 30.83, 29.98, 29.70, 29.65, 29.04, 27.28, 27.18, 26.84, 25.86, 25.75, 16.48, 16.18, 14.45.

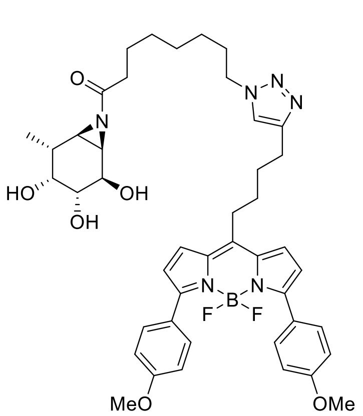

8-(4-(4-(5,5-Difluoro-3,7-bis(4-methoxyphenyl)-5H-4l4,5l4-dipyrrolo[1,2-c:2',1'-f]-[1,3,2]diazaborinin-10-yl)butyl)-1H-1,2,3-triazol-1-yl)-1-((1R,2R,3R,4R,5R,6R)-2,3,4-trihydroxy-5-methyl-7-azabicyclo[4.1.0]heptan-7-yl)octan-1-one (2): Azide **23** (31 mg, 0.099 mmol, 1 eq) was dissolved in DMF (3 mL), Bodipy compound **26**[4] (52.7 mg, 0.11 mmol, 1.1 eq), and aqueous solutions of $CuSO_4$ (1M, 20 µL, 0.019 mmol, 0.2 eq.) and sodium ascorbate (1M, 21 µL, 0.021 mmol, 0.2 eq) were added to the solution under argon atmosphere and the mixture was stirred at room temperature for 2h. The reaction was checked with LC/MS within the elution system of 10% NH_4OAc. The volatiles were removed under reduced pressure and the crude product was purified by semi-preparative reversed HPLC (linear gradient: 52-58% B in A, 12min, solutions used A: H_2O, B: acetonitrile) resulting a dark blue powder as the product **2** after lyophilization (15.32 mg, 0.019 mmol, 19%). LC/MS: R_t : 9.15 min; linear gradient 0-90% B in 15 min; ESI-MS: m/z = 811.8 $(M+H)^+$; HRMS: Calculated for $[C_{44}H_{53}BF_2N_6O_6 + H]^+$ 811.41681, Found: 811.41690. ^1H NMR (400 MHz, CD_3CN) δ 7.83 – 7.74 (m, 4H), 7.51 – 7.49 (s, 1H), 7.46 (d, *J* = 4.4 Hz, 2H), 7.03 – 6.94 (m, 4H), 6.69 (d, *J* = 4.4 Hz, 2H), 4.27 (t, *J* = 7.0 Hz, 2H), 3.91 (dd, *J* = 8.5, 3.9 Hz, 1H), 3.84 (s, 6H), 3.55 (dt, *J* = 3.4, 1.5 Hz, 1H), 3.25 (dd, *J* = 8.6, 1.8 Hz, 1H), 3.09 – 3.00 (m, 2H), 2.82 (dd, *J* = 6.1, 3.9 Hz, 1H), 2.78 – 2.69 (m, 2H), 2.44 – 2.23 (m, 3H), 1.88 – 1.74 (m, 6H), 1.50 (p, *J* = 7.3 Hz, 2H), 1.31-1.20 (m, 7H), 1.11 (d, *J* = 7.5 Hz, 3H); ^{13}C NMR (101 MHz, CD_3CN) δ 161.85, 158.30, 148.12, 147.71, 137.18,

132.13, 132.09, 132.05, 128.79, 126.09, 122.37, 121.27, 118.40, 114.62, 101.03, 74.97, 74.36, 69.23, 56.15, 50.66, 42.70, 41.99, 36.77, 36.05, 34.07, 31.01, 30.89, 30.34, 29.64, 29.28, 26.88, 25.72, 25.52, 16.24.

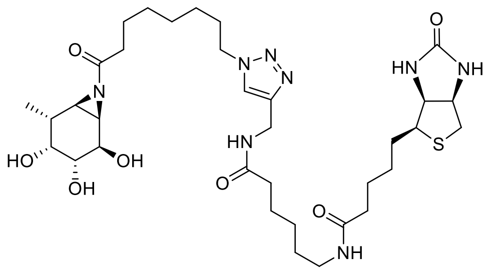

N-((1-(8-oxo-8-((1R,2R,3R,4R,5R,6R)-2,3,4-trihydroxy-5-methyl-7-azabicyclo[4.1.0]heptan-7-yl)octyl)-1H-1,2,3-triazol-4-yl)methyl)-6-(5-((3aS,4S,6aR)-2-oxohexahydro-1H-thieno[3,4-d]imidazol-4-yl)pentanamido)hexanamide (3) : Azide compound **23** (31mg,0.099mmol,1eq) was dissolved in DMF(3mL), biotin-ahx-alkyne **27** (37.5mg,0.099mmol,1eq), $CuSO_4$(1M) (20μL,0.019mmol,0.2eq) and sodium ascorbate (1M) (21μL,0.021mmol, 0.22eq) was added to the solution under argon atmosphere, and the mixture was stirred at 80 ℃ overnight, the reaction was followed by LC/MS. Then the crude product was purified by semi-preparative reversed HPLC (linear gradient: 18%→24% B in A, 12min, solutions used A: H_2O, B: actonitrile) and the fraction were freeze-dried without concentration resulting white powder product **3** (9.631mg, 0.013mmol, 13%). LC/MS: R_t : 4.31min; linear gradient 10→90% B in 15 min; ESI-MS: *m/z*=721.7$(M+H)^+$. HRMS: Calculated for $C_{34}H_{56}N_8O_7S$ $(M+H^+)$ 721.40655. Found: 721.40661. ^1H NMR (400 MHz, CD_3OD) δ 7.85 (s, 1H), 4.51 (dd, J = 7.6, 4.8 Hz, 1H), 4.42 (s, 2H), 4.38 (t, J= 3.6 Hz, 2H), 4.32 (dd, J= 8.0, 4.4 Hz, 1H), 4.08 (dd, J=8.8, 4.0 Hz, 1H), 3.61 (t, J = 1.6 Hz, 1H), 3.39 (dd, J = 8.8, 1.6 Hz, 1H), 3.22-3.14 (m, 3H), 2.95-2.90 (m, 2H), 2.72 (d, J = 12.8 Hz, 1H), 2.58-2.50 (m, 1H), 2.47-2.38 (m, 2H), 2.25-2.17 (m, 4H), 2.01- 1.96 (m, 1H), 1.95- 1.87 (m, 2H), 1.79- 1.58 (m, 8H), 1.53 - 1.41 (m, 4H), 1.37 - 1.30 (m, 8H), 1.21 (d, J =2.4Hz, 3H); ^{13}C NMR (100 MHz, CD_3OD) δ 188.7, 177.0, 176.0, 166.1, 146.3, 124.2, 75.8, 74.5, 69.3, 63.55, 63.4, 61.6, 57.0, 51.3, 43.6, 42.7, 41.1, 40.2, 36.9, 36.8, 36.7, 36.7, 35.6, 31.2, 30.1, 30.0, 29.9, 29.8, 29.7, 29.5, 27.5, 27.3, 27.2, 26.9, 26.8, 26.5, 25.8, 16.2.

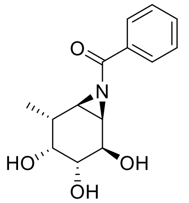

Phenyl((1*R*,2*R*,3*R*,4*R*,5*R*,6*R*)-2,3,4-trihydroxy-5-methyl-7-azabicyclo[4.1.0]heptan-7-yl)methanone (4): Benzoic acid (49 mg, 0.40 mmol, 2.0 eq.) and EEDQ (99 mg, 0.40 mmol, 2.0 eq.) were dissolved in anhydrous DMF (0.40 mL) and stirred at room temperature for 2h. This pre-activated mixed anhydride solution (200 µL, 1.0 eq) was added to aziridine **22** (31.8 mg, 0.2 mmol, 1.0 eq.) in dry DMF (1.0 mL) at 0 ℃ and stirred for 30 minutes. The remaining half of the pre-activated mixed anhydride solution (200 µL, 1.0 eq) was added and the resulting mixture was stirred at 0 ℃ for 2h. The reaction was quenched with MeOH (1.0 mL) and the mixture was concentrated *in vacuo*. The crude product was purified by HPLC (linear gradient: 15-21% B in A, 12 min, solutions used A: H_2O, B: actonitrile) giving compound **4** as white powder (3.8 mg, 14.6 µmol, 7% yield). TLC: R_f = 0.52 (DCM /MeOH, 5/1, v/v); LC/MS: R_t: 5.35min; linear gradient 0-90% B in 15 min; ESI-MS: m/z = 264.3 $(M+H)^+$; HRMS: Calculated for $[C_{14}H_{17}NO_4 + H]^+$ 264.12304, Found: 264.12308. ^1H NMR (400 MHz, CD_3OD) δ 8.21 – 8.11 (m, 2H), 7.60 (m, 1H), 7.48 (dd, *J* = 7.6 Hz, 2H), 4.18 (dd, *J* = 8.8, 4.0 Hz, 1H), 3.73-3.71(m, 1H) , 3.55 (dd, *J* = 8.8, 1.8 Hz, 1H), 3.14 (dd, *J* = 6.2, 4.0 Hz, 1H), 2.43 (d, *J* = 6.2 Hz, 1H), 2.27 (qd, *J* = 7.6, 3.3 Hz, 1H), 1.90 (s, 1H), 1.21 (d, *J* = 7.5 Hz, 3H); ^{13}C NMR (101 MHz, CD_3OD) δ 181.40, 134.11, 134.06, 130.50, 129.50, 75.78, 74.65, 69.40, 44.43, 44.34, 36.94, 16.30.

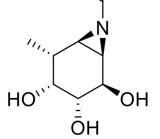

1-((1*R*,2*R*,3*R*,4*R*,5*R*,6*R*)-2,3,4-trihydroxy-5-methyl-7-azabicyclo[4.1.0]heptan-7-yl)ethan-1-one (5): Acetic acid (15.8 µL, 0.28 mmol, 2.0 eq.) and EEDQ (68.0 mg, 0.28 mmol, 2.0 eq.) were dissolved in anhydrous DMF (0.30 mL) and stirred at room temperature for 2h. This pre-activated mixed anhydride solution (150 µL, 1.0 eq.) was added to aziridine **22** (22.0 mg, 0.14 mmol, 1.0 eq.) in dry DMF (0.70 mL) at 0 ℃ and stirred for 30 min. The remaining half of the pre-activated mixed anhydride solution (150 µL, 1.0 eq.) was added and the resulting mixture was stirred at 0 ℃ for 2h. The reaction was quenched by adding MeOH (0.50 mL) and the mixture was concentrated *in vacuo*. The crude product was purified by semi-preparative reversed HPLC (linear gradient: 0%-10% B in A, 12 min, solutions used A: H_2O, B: acetonitrile) giving compound **5** as white powder after lyophilization (6.9 mg, 34 µmol, 25% yield). TLC: R_f = 0.38 (DCM /MeOH, 10/3, v/v); LC/MS: R_t : min; linear gradient 0-90% B in 2.13 min; ESI-MS: m/z = 202.2 $(M+H)^+$. HRMS: Calculated for $[C_8H_{15}NO_4 + H]^+$ 202.10738, Found: 202.10740. ^1H NMR (400 MHz, D_2O) δ 4.06 (dd, *J* = 8.9, 4.0 Hz, 1H), 3.76 – 3.63 (m, 1H), 3.46 (dd, *J* = 8.9, 1.8 Hz, 1H), 3.12 (dd, *J* = 6.0, 4.0 Hz, 1H), 2.56 (d, *J* = 6.1 Hz, 1H), 2.16 (s, 3H), 2.13-2.07 (m, 1H) 1.16 (d, *J* = 7.5 Hz, 3H); ^{13}C NMR (101 MHz, D_2O) δ 187.12, 74.33, 72.46, 67.67, 42.08, 42.03, 34.32, 22.58, 14.83.

3. Synthesis of compounds 28 and 29.

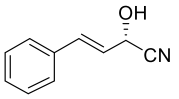

(*S*,*E*)-2-Hydroxy-4-phenylbut-3-enenitrile:[5] (**Caution!!!** Toxic gas (HCN) may evolve! Work in a well ventilated hood!) In an erlenmeyer flask KCN (26.4 g, 406 mmol) was dissolved in water (90 mL). On top a layer of MTBE (80 mL) was placed and the mixture was magnetically stirred at such a rate that the two layer system remains. Under slight ice-cooling an aqueous 20% ($^{w}/_{w}$) citric acid solution was added in portions until a pH of 5.45 was reached (pH meter control). At that time the mixture was transferred into a separation funnel, shaken firmly and separated. The water layer was extracted once more with MTBE (80 mL) and the combined MTBE layers were combined and kept on ice. In the mean time a 500 mL three necked flask, equipped with a magnetic stirrer and a thermometer, was charged with a citrate buffer (75 mL, 0.1 M, pH 5.45), MTBE (20 mL) and cinnamon aldehyde (14.7 g, 111 mmol). The mixture was cooled on an ice bath and *Hb*HNL extract (4.5 g) was added. Under argon and vigorous stirring the ice cold HCN buffer was added drop wise in 15 minutes at 8 °C. The reaction was stirred at this temperature for one hour and for 24 hours at room temperature. At this time TLC showed almost complete conversion and the reaction was stopped. The layers were separated, the water layer extracted once more with MTBE (50 mL). The combined MTBE layers were dried ($MgSO_4$), filtered and evaporated to afford the crude product as a yellow oil (20.6 g, 93% e.e. as determined by chiral HPLC). After two crystallizations from DCM/pentane the target cyanohydrin was obtained (9.71 g, 55 %, e.e. = 99%) as colorless crystals. $[\alpha]_D^{23}$ = -30 (*c* = 1, $CHCl_3$), lit[6] $[\alpha]_D^{20}$ = -21.8 (*c* = 0.97, $CHCl_3$), lit[7] $[\alpha]_D^{28}$ = -24.1 (*c* = 0.8, $CHCl_3$). ^{1}H NMR (400 MHz, $CDCl_3$) δ 7.41 - 7.37 (m, 2H), 7.37 - 7.27 (m, 3H), 6.88 (d, *J* = 16.1 Hz, 1H), 6.23 (dd, *J* = 16.1, 6.2 Hz, 1H), 5.14 (dd, *J* = 6.2, 6.2, Hz, 1H), 3.28 (d, *J* = 7.2 Hz, 1H). Chiral HPLC: (Daicel Chiralcel OD, UV 254 nm, Hexane/2-propanol/acetic acid = 85/15/0.1; 1.0 mL/min: R_t = 14.8 min.).

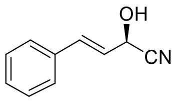

(*R*,*E*)-2-Hydroxy-4-phenylbut-3-enenitrile:[8] (**Caution!!!** Toxic gas (HCN) may evolve! Work in a well ventilated hood!) In an erlenmeyer flask KCN (28.3 g, 435 mmol) was dissolved in water (100 mL). On top a layer of MTBE (100 mL) was placed and mixture was magnetically stirred at such a rate that the two layer system remains. Under slight ice-cooling an aqueous 20% ($^{w}/_{w}$) citric acid solution was added in portions until a pH of 5.45 was reached (pH meter control). At that time the mixture was transferred into a separation funnel, shaken firmly and separated. The water layer is extracted once more with MTBE (100 mL) and the combined MTBE layers were combined and kept on ice. In the mean time, a 500 mL three necked flask, equipped with a magnetic stirrer and a thermometer, was charged with a citrate buffer (50 mL, 0.1 M, pH 5.45), MTBE (60 mL) and cinnamon aldehyde (21.5 g, 163 mmol). The mixture was cooled on an ice bath and *pa*HNL (142 mg) was added. Under vigorous stirring the ice cold HCN buffer was added drop wise in 15 minutes. After 64 hours the reaction was stopped, the layers separated and the water layer extracted once more with MTBE (50 mL). The combined MTBE layers were dried ($MgSO_4$), filtered and evaporated to afford a yellow oil (28.8 g) as the crude product. The oil was dissolved in DCM (150 mL) and pentane (200 mL) was added. After standing at room temperature for 2 hours and 2 hours at 4 °C the formed crystals were collected by filtration and washed with cold pentane twice. Drying afforded the title compound as colorless crystals (11.8 g, 46 %, e.e. = 99%). $[\alpha]_D^{23}$ = +30 (*c* = 1, $CHCl_3$), lit[9] $[\alpha]_D^{20}$ = +28.8 (92% e.e.; *c* = 1.02, $CHCl_3$), lit[8] $[\alpha]_D^{28}$ = +30.5 (*c* = 1.0, $CHCl_3$). ^{1}H NMR (400 MHz, $CDCl_3$) δ 7.45 - 7.40 (m, 2H), 7.40 - 7.30 (m, 3H), 6.92 (d, *J* = 15.8 Hz, 1H), 6.26 (dd, *J* = 15.8, 6.0 Hz, 1H), 5.17 (dd, *J* = 6.2, 6.2 Hz, 1H), 2.86 (d, *J* = 7.1 Hz).

Determination of enantiomeric excess: Chiral HPLC: (Daicel Chiralcel OD, UV 254 nm, Hexane/2-propanol /acetic acid = 85/15/0.1, 1.0 mL/min.). Chromatograms shown below: left side, *S*-enantiomer e.e. > 99% (R_t = 14.7 min.); right side, *R*-enantiomer e.e. = 99% (R_t = 12.8 min.).

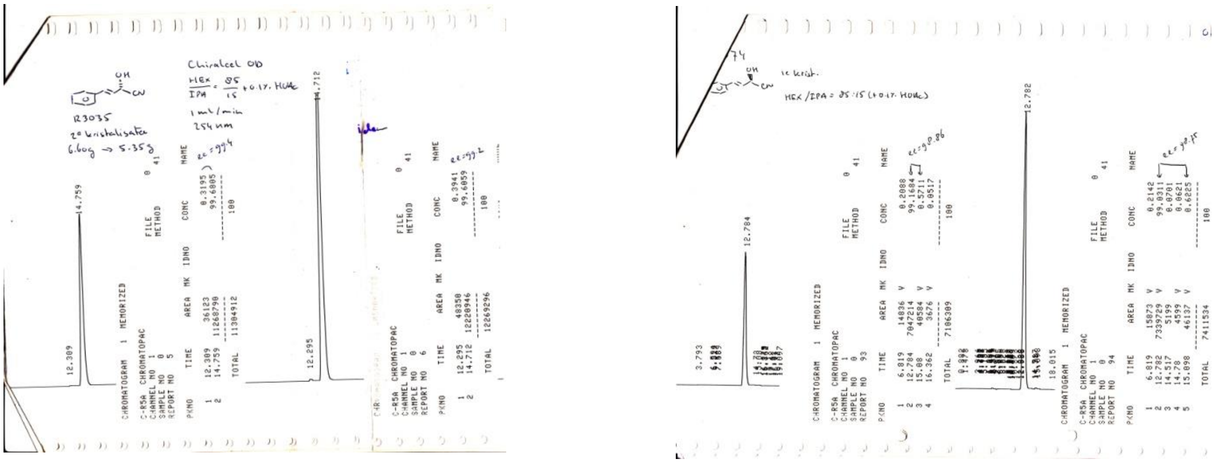

Figure 3.1: Chiral HPLC traces for (*S*)- and (*R*)-2-Hydroxy-4-phenylbut-3-enenitrile.

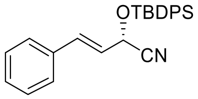

(*S,E*)-2-((*tert*-Butyldiphenylsilyl)oxy)-4-phenylbut-3-enenitrile((*S*)-28): *tert*-Butylchlorodiphenylsilane (7.20 g, 26.2 mmol) was dissolved in DMF (80 mL) and imidazole (2.7 g, 40.0 mmol) was added. The mixture was stirred at room temperature for 15 min. Then it was cooled on ice and (*S,E*)-2-hydroxy-4-phenylbut-3-enenitrile (3.18 g, 20.0 mmol) was added and the reaction stirred for 24 h. TLC analysis revealed complete conversion and the reaction was quenched with H_2O (250 ml), extracted with Et_2O (3 x 100 mL). The combined organic layers were washed with H_2O (50 mL) and brine (50 mL), dried with $MgSO_4$ and evaporated. The mixture was purified by silicagel column chromatography (pentane/EtOAc = 99/1 → 98/2) to afford the title compound as a colorless oil (7.80 g, 98 %). $[\alpha]_D^{23}$ = +75 (*c* = 1, $CHCl_3$); ^{1}H NMR (400 MHz, $CDCl_3$) δ 7.75 (d, *J* = 7.6, 2H), 7.67 (d, *J* = 7.6, 2H), 7.52 - 7.23 (m, 11H), 6.56 (d, *J* = 15.8 Hz, 1H), 6.13 (d, *J* = 15.8, 6.4 Hz, 1H), 4.97 (d, *J* = 6.4 Hz, 1H), 1.12 (s, 9H). ^{13}C NMR (101 MHz, $CDCl_3$) δ 135.92, 135.87, 135.13, 131.97, 131.59, 130.57, 130.47, 128.85, 128.78, 128.13, 128.05, 127.05, 134.47, 123.38, 118.28, 63.61, 26.77, 19.40. IR (cm^{-1}) 3024, 2933, 2860, 1472, 1428, 1116, 1112, 1075, 1060, 965, 753, 741, 700, 613, 504.

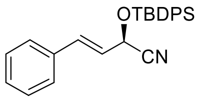

(*R,E*)-2-((*tert*-Butyldiphenylsilyl)oxy)-4-phenylbut-3-enenitrile((*R*)-28): Prepared as described for compound **28**, obtained as a pail yellow oil (54.0 mmol scale, yield 21.2 g, 99%). $[\alpha]_D^{24}$ = -75 (*c* = 1, $CHCl_3$); ^{1}H NMR (400 MHz, $CDCl_3$) δ 7.75 (d, *J* = 7.7, 2H), 7.67 (d, *J* = 7.7, 2H), 7.51 - 7.22 (m, 11H), 6.56 (d, *J* = 15.8 Hz, 1H), 6.13 (dd, *J* = 15.8, 6.4 Hz, 1H), 4.97 (d, *J* = 6.4 Hz, 1H), 1.18 (s, 9H). ^{13}C NMR (101 MHz, $CDCl_3$) δ 135.93, 135.88, 135.75, 135.14, 134.89, 131.99, 131.60, 130.67, 130.58, 130.47, 129.68, 128.86, 128.79, 128.39, 128.14, 128.05, 127.77, 127.06, 126.66, 134.49, 123.40, 118.28, 63.62, 26.78, 19.41.

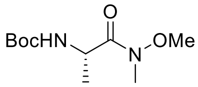

***tert*-Butyl (*S*)-(1-(methoxy(methyl)amino)-1-oxopropan-2-yl)carbamate:**[10] A solution of Boc-L-Alanine (19.2 g, 102 mmol) in CH_2Cl_2 (400 mL) was cooled to -15 ºC followed by addition of *N,O*-dimethylhydroxylamine hydrochloride (10.1 g, 103 mmol) and then NMM (11.3 ml, 103 mmol). *N*-(3-Dimethylaminopropyl)-*N*-ethylcarbodiimide hydrochloride (19.8 g, 103 mmol) was added portion wise as a solid over 30 minutes. The reaction was stirred at room temperature for 24 hours. After cooling on an ice-bath, 1M HCI was added (30 mL).The aqueous layer was extracted twice with CH_2Cl_2 (150 mL) and the combined organic layers were washed with a saturated aqueous $NaHCO_3$ solution (60 mL), dried ($MgSO_4$), filtered and the solvent was evaporated under vacuum to give the Weinreb amide as a white solid (22.1 g, 94%) that was used without purification. ^{1}H NMR (400 MHz, $CDCl_3$) δ 5.28 (d, *J* = 7.0 Hz), 4.75 - 4.60 (m, 1H), 3.77 (s, 3H), 3.21 (s, 3H), 1.44 (s, 9H), 1.31 (d, *J* = 6.9 Hz, 3H). ^{13}C NMR (101 MHz, $CDCl_3$) δ 155.36, 79.58, 61.71, 46.62, 32.25, 28.47, 18.78.

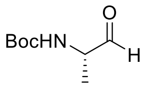

***tert-B*utyl (*S*)-(1-oxopropan-2-yl)carbamate:**[10] Weinreb amide from above (22.1 g, 95.2 mmol) was dissolved in anhydrous THF (300 mL) and cooled to 0 ℃. A solution of 2.0 M $LiAlH_4$ in THF (47.7 ml, 95.4 mmol) was added dropwise and the mixture was stirred for another 30 minutes. The reaction was cooled to -15 ℃ and a saturated aqueous $KHSO_4$ solution (250 mL) was added carefully. The solution was diluted with Et_2O (500 mL) and stirred vigorously for 30 min. The organic layer was separated, dried with $MgSO_4$, filtered and the solvent was evaporated under vacuum to give the aldehyde as a white solid (16.4 g, quant.) that was used crude. ^{1}H NMR (400 MHz, $CDCl_3$) δ 9.58

(s, 1H), 5.36 - 4.91 (m, 1H), 4.30 - 4.07 (m, 1H), 1.46 (s, 9H), 1.33 (d, *J* = 7.3 Hz, 3H). ^{13}C NMR (101 MHz, $CDCl_3$) δ 199.93, 155.41, 80.06, 55.61, 28.39, 14.92.

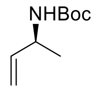

***tert*-Butyl (*S*)-but-3-en-2-ylcarbamate:**[10] Methyltriphenylphosphonium bromide (56.8 g, 159 mmol) was suspended in THF (750 mL) at room temperature and KHMDS (30.3 g, 152 mmol) was added. The resultant yellow suspension was stirred at room temperature for 1 hour and then cooled to -78 ℃ and a solution of the aldehyde from above (16.4 g, 95.2 mmol) dissolved in THF (150 mL) was added dropwise. The cooling bath was removed and the mixture was stirred for another 2 hours. The reaction was quenched with MeOH (100 mL) and the resulting mixture was poured into saturated ammonium chloride solution (500 mL). Extraction with Et_2O (3 x 200 mL), drying ($MgSO_4$) and evaporation of the solvent in vacuo afforded an orange semi-solid that was treated several times with pentane. The combined pentane fractions were filtered and the solvent was evaporated to give the target compound as a white solid that was purified by silica gel column chromatography (Pentane/EtOAc = 98/2 → 97/3 → 95/5) to afford the title compound as a white solid (14.9 g, 91 %). $[\alpha]_D^{23}$ = -4.2 (*c* = 1, $CHCl_3$), lit[11] $[\alpha]_D^{26}$ = -6.33 (*c* = 1.2, $CHCl_3$); HRMS calculated for $[C_9H_{17}NO_2 + H]^+$: 173.13321; found: 173.13307. ^1H NMR (400 MHz, $CDCl_3$) δ 5.82 (ddd, *J* = 17.2, 10.4, 5.0 Hz), 5.24 (d, *J* = 7.7 Hz, 1H), 5.12 (d, *J* = 17.2 Hz, 1H), 5.01 (d, *J* = 10.4 Hz, 1H), 4.31 - 4.14 (m, 1H), 1.43 (s, 1H), 1.20 (d, *J* = 7.0 Hz, 3H). ^{13}C NMR (101 MHz, $CDCl_3$) δ 154.70, 139.88, 112.84, 78.14, 47.57, 27.93, 20.04. IR (cm^{-1}) 3459, 3020, 1703, 1498, 1215, 1170, 748.

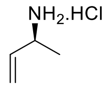

(*S*)-But-3-en-2-amine hydrochloride ((*S*)-29): The Boc-protected amine (14.2 g, 82.7 mmol) from above was dissolved in MeOH (110 mL), aqueous 6M HCl (100 mL) was added and the mixture stirred overnight at room temperature. The solvents were evaporated using a water aspirator affording the title salt as an off white solid (10.3 g, quant, 92% overall, e.e. > 99%). $[\alpha]_D^{23}$ = +2.4 (*c* = 1, MeOH), lit[12] $[\alpha]_D^{20}$ = -3.5 (*c* = 1, EtOH); ^1H NMR (400 MHz, D_2O) δ 5.90 (ddd, *J* = 17.2, 10.6, 6.7 Hz, 1H), 5.37 (d, *J* = 17.2 Hz, 1H), 5.33 (d, *J* = 10.6 Hz, 1H), 3.92 (c, *J* = 6.7 Hz, 1H), 2.20 (s, 2H), 1.37 (d, *J* = 6.8 Hz, 3H). ^{13}C NMR (101 MHz, D_2O) δ 134.76, 118.62, 49.34, 17.90. IR (cm^{-1}) 3400 - 3200, 1649, 1610, 1483, 1425, 1385, 1019, 995, 927, 659.

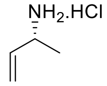

(*R*)-But-3-en-2-amine hydrochloride ((*R*)-29): Prepared from Boc-D-Alanine in the same manner as described above for the (*S*)-enantiomer, e.e. = 95%. $[\alpha]_D^{23}$ = -3.6 (*c* = 1, MeOH). All spectral data were identical.

Determination of enantiomeric excess: Analytical samples of both obtained amine hydrochlorides were treated with benzoyl chloride in DCM in the presence of triethyl amine. After work up and purification these samples were subjected to Chiral HPLC analysis on a Daicel Chiralcel OD column (250 x 4.5 mm) using Hexane / 2-propanol = 90 /10, 1.0 ml/min, UV detection (254 nm). (*R*)-isomer, e.e. = 95% (left chromatogram); (*S*)-isomer, e.e. > 99% (left chromatogram); See chromatograms below.

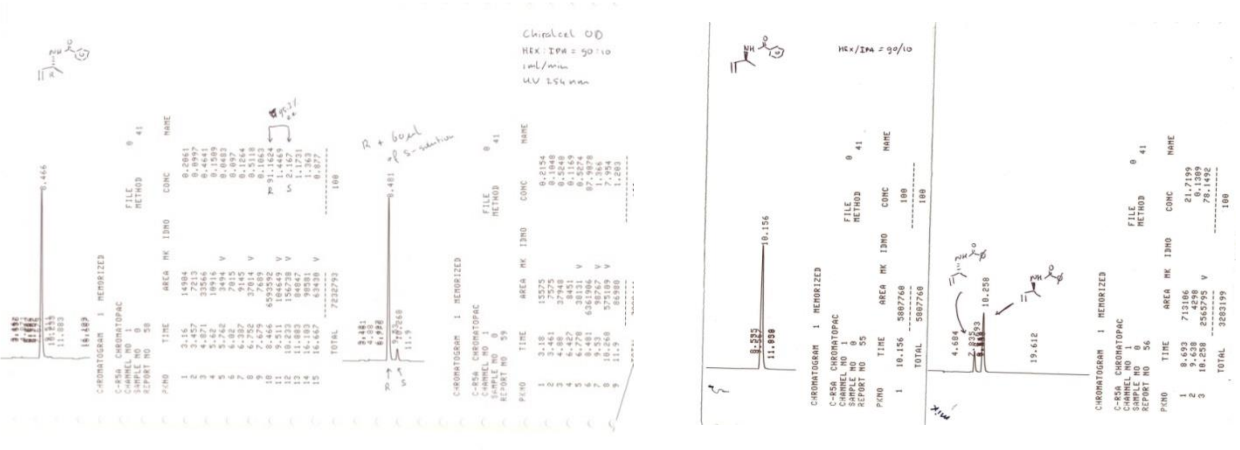

Figure 3.2: Chiral HPLC traces for (R)- and (S)-N-(but-3-en-2-yl)benzamide.

4. Preparation of fuconojirimycin (6) and the configurational isomers 7 – 13.

*Scheme S1: Preparation of fuconojirimycin **(6)** from cyanohydrin **(S)-28**.*

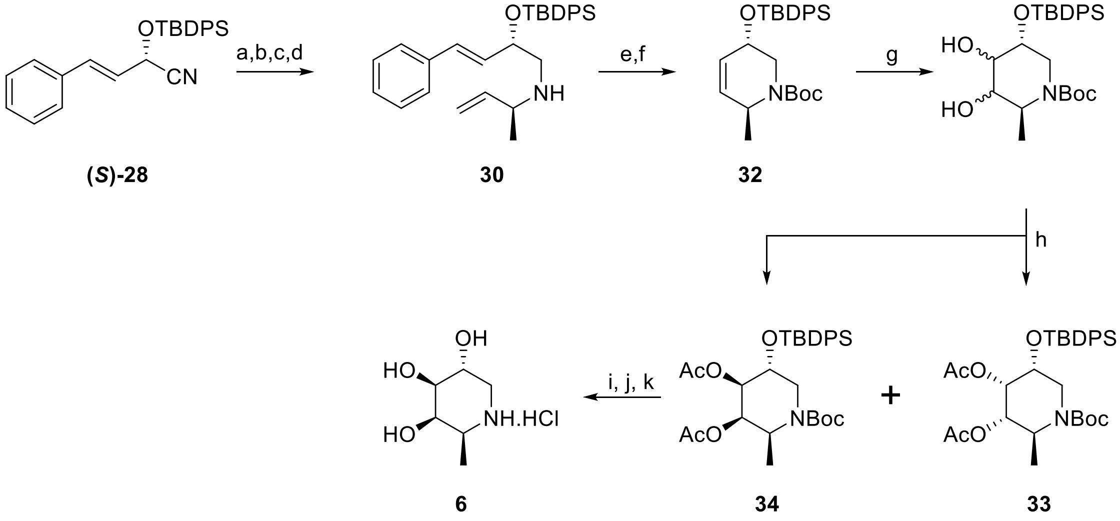

Reagents and conditions: a) Dibal-H, -78 → 5 °C; b) MeOH, -90 °C; c) amine **(*S*)-29**, NaOMe, RT, 18 h; d) $NaBH_4$, 5 °C → RT, 4 h; e) Boc_2O, 50 °C; f) Grubbs I, DCM, reflux; g) $K_2OsO_4.2H_2O$, NMO, acetone/water; h) Ac_2O, pyridine, DMAP, 0 °C → RT; i) K_2CO_3, MeOH; j) TBAF, THF; k) 6M HCl, MeOH.

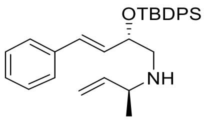

(*S*,*E*)-*N*-((*S*)-But-3-en-2-yl)-2-((*tert*-butyldiphenylsilyl)oxy)-4-phenylbut-3-en-1-amine (30): In a flame dried flask and under argon atmosphere, a solution of ((*S*,*E*)-2-((tert-butyldiphenylsilyl)oxy)-4-phenylbut-3-enenitrile (3.57 g, 9.00 mmol) in dry Et_2O (80 mL) was cooled to -78 °C. A 1M solution of Dibal-H in toluene (13.5 mL, 13.5 mmol) was added dropwise and the reaction was allowed to warm up slowly to 10 °C. After cooling to -90 °C, absolute MeOH (13.5 mL) was added at once, followed by a solution (*S*)-but-3-en-2-amine hydrochloride (3.17 g, 29.5 mmol, 3.3 eq.) in MeOH (20 mL). Subsequently dry sodium methoxide (2.41 g, 44.6 mmol) was added to deprotonate the (*S*)-but-3-en-2-amine hydrochloride *in situ*. The cooling bath was removed and the mixture stirred overnight at room temperature under a light flow of argon to reduce the volume of the reaction by half. The mixture was cooled on an ice bath and $NaBH_4$ (1.24 g, 32.7 mmol) was added in three portions. After stirring for 30 min on the ice bath and two hours at room temperature, the reaction was poured in to an aqueous 0.5 M NaOH (90 mL) solution and extracted with diethyl ether (3 x 80 mL). The combined organic layers were washed with a cold aqueous 1M HCl solution (100 mL). Evaporation of this acidic aqueous layer afforded recovered (*S*)-but-3-en-2-amine hydrochloride (2.07 g, 19.2 mmol). The organic layer was washed subsequently with aqueous 0.5 M NaOH (60 mL) solution and brine (30 mL). Drying on $MgSO_4$, filtering and evaporation of the solvent afforded the crude product that was purified by silicagel column chromatography (pentane/EtOAc = 97/3 → 95/5 → 9/1) to afford the target compound as a yellow oil (3.60 g, 88%). $[\alpha]_D^{23}$ = +128 (*c* = 1, $CHCl_3$) HRMS calculated for $[C_{30}H_{37}NOSi + H]^+$: 456.27172; found: 456.27122. ^1H NMR (400MHz, $CDCl_3$) δ 7.72 - 7.62 (m, 4H), 7.46 - 7.13 (m, 11H), 6.19 (d, *J* = 16.0 Hz, 1H), 6.10 (dd, *J* = 16.0, 7 Hz, 1H), 5.60 (ddd, *J* = 17.4, 10.0, 7.7 Hz, 1H), 5.01 (d, *J* = 17.4 Hz, 1H), 4.98 (d, *J* = 10.0 Hz, 1H), 4.45 (dt, *J* = 12.5, 6.3 Hz, 1H), 3.11 (m, 1H), 2.72 (dd, *J* = 11.8, 6.3 Hz, 1H), 2.68 (dd, *J* = 11.8, 5.8 Hz, 1H), 1.08 (s, 9H), 1.05 (d, *J* = 3.4 Hz, 3H). ^{13}C NMR (101MHz, $CDCl_3$) δ 142.24, 136.80, 136.12, 136.03, 135.68, 134.97, 134.15, 134.06, 131.46, 130.68, 129.80, 129.69, 128.50, 127.71, 127.58, 126.60, 114.88, 74.34, 56.73, 53.64, 27.22, 21.50, 19.50. IR (cm^{-1}) 3071, 2958, 2930, 2856, 1471, 1427, 1109, 740.

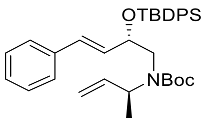

***tert*-Butyl ((*S*)-but-3-en-2-yl)((*S*,*E*)-2-((*tert*-butyldiphenylsilyl)oxy)-4-phenylbut-3-en-1-yl)carbamate (31):** To compound **30** (8.28 g, 15.6 mmol) was added Boc_2O (5.10 g, 23.4 mmol) and the mixture was stirred overnight at 50 °C. TLC analysis showed complete conversion and after evaporation of the solvent the mixture was purified by silica gel column chromatography (pentane/EtOAc = 98/2 → 97/3 → 95/5) to afford the title compound **31** as a colorless oil (8.70 g, 100%). $[\alpha]_D^{23}$ = +27 (*c* = 1, $CHCl_3$);

HRMS calculated for $[C_{35}H_{45}NO_3Si + H]^+$: 556.32415; found: 556.32387. ^{1}H NMR (400 MHz, $CDCl_3$) δ 7.78 – 7.62 (m, 4H), 7.47 – 7.12 (m, 11H), 6.15 – 6.00 (m, 2H), 5.77 (ddd, *J* = 16.3, 10.6, 5.2 Hz, 1H), 5.00 – 4.86 (m, 2H), 4.53 – 4.37 (m, 1H), 3.60 – 3.37 (m, 1H), 3.37 – 3.19 (m, 1H), 3.19 - 3.01 (m, 1H), 1.47 - 1.18 (m, 9H), 1.07 (s, 9H), 1.06 (d, *J* = 3.4 Hz, 3H). ^{13}C NMR (101 MHz, $CDCl_3$) δ 139.54, 136.15, 136.05, 135.52, 131.12, 130.82, 129.78, 129.69, 128.43, 127.93, 129.69, 128.43, 127.93, 127.69, 127.56, 126.56, 115.13, 73.55, 50.13, 45.54, 28.45, 27.20, 19.44, 17.69. IR (cm^{-1}) 3072, 2933, 2858, 1690, 1428, 1391, 1365, 1164, 1111, 736.

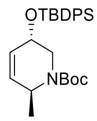

***tert*-Butyl (3*S*,6*S*)-3-((*tert*-butyldiphenylsilyl)oxy)-6-methyl-3,6-dihydropyridine-1(2*H*)-carboxylate (32):** Boc-protected diene **31** (8.70 g, 15.6 mmol) was dissolved in DCM and argon was bubbled through the solution for five minutes. Grubbs I (260 mg, 0.316 mmol, 2.0 mol %) was added and the reaction refluxed under argon for 48 hours after which TLC analysis revealed complete conversion. Evaporation of the solvent and silica gel column chromatography (pentane/EtOAc = 97/3→ 95/5) afforded the compound **32** as a colorless oil (6.85 g, 97%). $[\alpha]_D^{23}$ = +158 (*c* = 1, $CHCl_3$); HRMS calculated for $[C_{27}H_{37}NO_3Si + H]^+$: 452.26155; found: 452.26159. ^{1}H NMR (400 MHz, $CDCl_3$) δ 7.72 (d, *J* = 6.8 Hz, 2H), 7.66 (d, *J* = 6.8 Hz, 2H), 7.46 -7.32 (m, 6H), 5.74 - 5.43 (m, 1H), 5.60 - 5.43 (m, 1H), 4.67 - 4.46 (m, 1H), 4.27 - 4.08 (m, 1H), 4.08 - 3.97 (m, 1H), 2.96 - 2.75 (m, 1H), 1.50 (s, 9H), 1.08 (d, *J* = 7.0 Hz, 3H), 1.05 (s, 9H). ^{13}C NMR (101 MHz, $CDCl_3$) δ 154.80, 135.93, 134.93, 134.12, 133.58, 129.80, 127.78, 125.39, 79.46, 64.20, 49.91, 41.94, 28.63, 27.02, 19.34, 17.18. IR (cm^{-1}) 2965, 2931, 2858, 1695, 1416, 1384, 1175, 1131, 1106, 1073, 702.

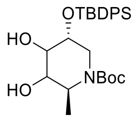

Upjohn dihydroxylation of compound 32: Compound **32** (8.26 g, 18.3 mmol) was dissolved in a mixture of acetone (70 mL) and water (70 mL) and cooled to -10 ℃. *N*-Methylmorpholine-*N*-oxide monohydrate (6.70 g, 49.8 mmol) and $K_2OsO_4.2H_2O$ (72 mg, 0.19 mmol, 1.04 mol %) were added subsequently. After 24 - 48 hours TLC analysis showed complete conversion of the starting material **32**. The reaction was quenched with an aqueous saturated Na_2SO_3 solution (100 mL) and stirred for 30 min. The mixture was diluted with water (100 mL) and extracted with EtOAc (3 x 60 mL). The combined organic layers were washed with 0.6 M HCl, saturated aqueous $NaHCO_3$ and brine. After drying (Na_2SO_4), filtering and evaporation of the solvent, afforded a 3:1 mixture of diastereoisomers (5.42 g, 61%) that could not be separated by silica gel column chromatography.

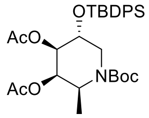

(2*S*,3*R*,4*R*,5*R*)-1-(*tert*-butoxycarbonyl)-5-((*tert*-butyldiphenylsilyl)oxy)-2-methylpiperidine-3,4-diyl diacetate (34): Mixture from above (5.42 g, 11.1 mmol) was dissolved in pyridine (25 mL) and cooled to 0 ℃. Acetic anhydride (6.0 mL, 63.6 mmol) and a few crystals of DMAP were added and the reaction was stirred for 24 – 48 hours at room temperature. TLC analysis showed complete conversion of the starting material. The reaction was diluted with toluene (100 mL) and the solvents evaporated. The resulting mixture was diluted with EtOAc (100 mL) and washed with H_2O (50 mL), 1 M HCl (50 mL), saturated aqueous $NaHCO_3$ solution (50 mL) and brine (50 mL). After drying (Na_2SO_4), filtering and evaporation of the solvent, the silica gel column chromatography (pentane/EtOAc = 97/3→ 95/5→ 93/7) afforded compound **32** as the first eluting isomer, pale yellow oil (4.02 g, 63%). $[\alpha]_D^{23}$ = +12 (*c* = 1, $CHCl_3$); HRMS calculated for $[C_{31}H_{43}NO_7Si + H]^+$: 570.28816; found: 570.28780. ^{1}H NMR (400 MHz, $CDCl_3$) δ 7.72 – 7.66 (m, 4H), 7.47 – 7.34 (m, 6H), 5.44 (dd, *J* = 6.9, 3.1 Hz, 1H), 5.12 (dd, *J* = 3.9, 3.9 Hz, 1H), 4.54 (qd, *J* = 6.9, 6.9 Hz, 1H), 3.94 (d, *J* = 14.2 Hz, 1H), 3.76 (ddd, *J* = 3.9, 1.5, 1.5 Hz, 1H), 3.07 (dd, *J* = 14.2, 1.5 Hz, 1H) 2.03 (s, 3H), 1.93 (s, 3H), 1.46 (s, 9H), 1.20 (d, *J* = 6.9 Hz, 3H**)**, 1.10 (s, 9H). ^{13}C NMR (101 MHz, $CDCl_3$) δ 169.79, 169.37, 154.89, 135.97, 135.94, 133.13, 133.05, 130.03, 129.94, 127.85, 127.80, 80.03, 70.99, 68.68, 67.41, 48.08, 40.83, 28.48, 26.94, 21.00, 20.96, 19.27, 12.50. IR (cm^{-1}) 2933, 2859, 1752, 1697, 1418, 1366, 1284, 1162, 703.

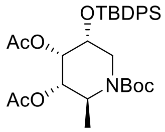

(2*S*,3*S*,4*S*,5*R*)-1-(*tert*-butoxycarbonyl)-5-((*tert*-butyldiphenylsilyl)oxy)-2-methylpiperidine-3,4-diyl diacetate (33): Obtained as the later eluting isomer, pale yellow oil (1.21 g, 19%). ^{1}H NMR (400 MHz, $CDCl_3$) δ 7.78 – 7.69 (m, 4H), 7.45 – 7.32 (m, 6H), 5.03 (m, 1H), 4.93 (app. t, *J* = 3.2 Hz, 1H), 4.60 (q, *J* = 7.2 Hz, 1H), 4.06 – 3.97 (m, 2H), 2.89 (d, *J* = 11.2 Hz, 1H), 2.15 (s, 3H), 1.83 (s, 3H), 1.41 (s, 9H), 1.19 (d, *J* = 7.4 Hz, 3H), 1.11 (s, 9H). ^{13}C NMR (101 MHz, $CDCl_3$) δ 170.89, 170.16, 154.99, 136.10, 136.01, 133.69, 133.23, 129.75, 129.60, 127.63, 127.29, 79.90, 70.90, 68.12, 67.33, 51.01, 44.19, 28.32, 26.76, 21.35, 20.77, 19.52, 14.61.

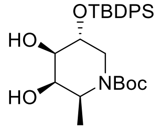

***tert*-Butyl (2*S*,3*R*,4*R*,5*R*)-5-((*tert*-butyldiphenylsilyl)oxy)-3,4-dihydroxy-2-methylpiperidine-1-carboxylate:** Compound **34** (3.98 g, 6.98 mmol) was dissolved in MeOH (100 mL) and K_2CO_3 (1.30 g, 9.42 mmol) was added. The reaction was stirred for 24 hours after which TLC analysis showed complete conversion of the material **34**. The reaction was acidified with AcOH until pH 5, subsequently diluted with EtOAc (80 mL) and washed with Brine (80 mL). Drying (Na_2SO_4), filtering, evaporation of the solvent and silica gel column

chromatography (pentane/EtOAc = 9/1→ 3/1→ 1/1) afforded the title compound as a pale yellow oil (3.39 g, quant.). $[\alpha]_D^{23}$ = -4.2 (*c* = 1, $CHCl_3$); HRMS calculated for $[C_{27}H_{39}NO_5Si + H]^+$: 486.26703; found: 486.26669. ^1H NMR (400 MHz, $CDCl_3$) δ 7.71 (dd, *J* = 7.8, 1.2 Hz, 2H), 7.68 (dd, *J* = 7.8, 1.2 Hz, 2H), 7.50 - 7.37 (m, 6H), 4.51 (qd, *J* = 6.9, 6.9 Hz, 1H), 4.14 (dd, *J* = 6.4, 3.1 Hz, 1H), 3.93 - 3.84 (m, 2H), 3.77 (dd, *J* = 3.1, 3.1 Hz, 1H), 3.16 (dd, *J* = 14.5, 1.8 Hz, 1H), 2.80 - 2.17 (m, 2H), 1.48 (s, 9H), 1.23 (d, *J* = 7.1 Hz, 3H**),** 1.10 (s, 9H). ^{13}C NMR (101 MHz, $CDCl_3$) δ 155.44, 135.98, 135.82, 133.86, 133.38, 130.00, 128.22, 128.22, 128.01, 127.86, 127.85, 127.09, 127.09, 79.83, 72.46, 70.70, 66.59, 49.83, 39.67, 28.59, 27.09, 19.34, 12.04. IR (cm^{-1}) 3412, 2832, 2858, 1662, 1426, 1365, 1158, 1092, 1017, 755, 740, 700.

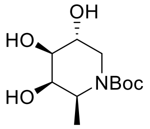

***tert*-Butyl (2*S*,3*R*,4*S*,5*R*)-3,4,5-trihydroxy-2-methylpiperidine-1-carboxylate:** The TBDPS-ether from above (1.52 g, 3.13 mmol) was dissolved in THF (30 mL) and TBAF.3H_2O (2.77 g, 8.80 mmol) was added at room temperature. The reaction was stirred at ambient temperature overnight. TLC indicated complete conversion and the mixture was concentrated. The crude compound was purified by silica gel column chromatography (pentane/EtOAc = 1/1 → 0/1 → EtOAc) to afford the title compound as a colorless oil (727 mg, 94%). $[\alpha]_D^{23}$ = +19 (*c* = 1, $CHCl_3$); HRMS calculated for $[C_{11}H_{21}NO_5 + H]^+$: 248.14925; found: 248.14920. ^1H NMR (400 MHz, CD_3OD) δ 4.28 (dq, *J* = 6.9, 6.9 Hz, 1H), 3.86 (dd, *J* = 6.9, 2.9 Hz, 1H), 3.85 – 3.75 (m, 3H), 3.27 (dd, *J* = 14.2, 1.7 Hz, 1H), 1.46 (s, 9H), 1.25 (d, *J* = 6.9 Hz, 3H). ^{13}C NMR (101 MHz, CD_3OD) δ 157.62, 81.17, 73.43, 71.01, 67.57, 52.28, 40.98, 28.90, 12.71. IR (cm^{-1}) 3400 - 3200, 2977, 2931, 1659, 1420, 1365, 1315, 1252, 1158, 1069, 1044, 1015, 732.

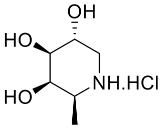

(2*S*,3*R*,4*S*,5*R*)-2-methylpiperidine-3,4,5-triol hydrochloride (6): The Boc-protected-imino sugar from above (645 mg, 2.61 mmol) was dissolved in a mixture of MeOH (20 mL) and aqueous 6M HCl (3 mL) and stirred overnight at room temperature. The mixture was concentrated to afford the title compound **6** as a white foam (366 mg, 76%). $[\alpha]_D^{23}$ = -36 (*c* = 1, MeOH); HRMS calculated for $[C_6H_{13}NO_3 + H]^+$: 148.09682; found: 148.09658. ^1H NMR (400 MHz, D_2O) δ 4.04 – 3.92 (m, 2H), 3.60 (dd, *J* = 9.9, 2.8 Hz, 1H), 3.45 – 3.37 (m, 2H), 2.81 (t, *J* = 12.0 Hz, 1H), 1.30 (d, *J* = 6.7 Hz, 3H). ^{13}C NMR (101 MHz, D_2O) δ 73.04, 69.75, 64.29, 54.92, 46.01, 14.01. IR (cm^{-1}) 3400 - 3200, 2942, 2816, 2464, 1457, 1388, 1076, 1003.

*Scheme S2: Preparation of iminosugar **7** from intermediate **34**.*

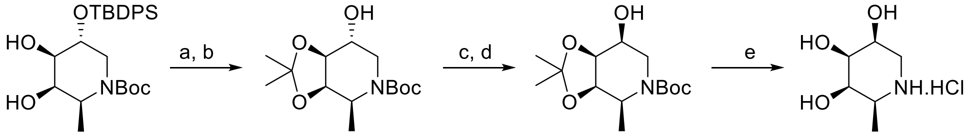

35 **36** **7**

Reagents and conditions: a) Acetone / 2,2-dimethoxypropane, $BF_3.EtO_2$, 5 ^0C; b) TBAF, THF; c) Dess-Martin, DCM; d) $NaBH_4$, EtOH, -78 ^0C → RT; e) 6M HCl, MeOH.

.

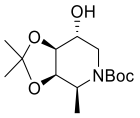

***tert*-Butyl (3a*R*,4*S*,7*R*,7a*S*)-7-hydroxy-2,2,4-trimethyltetrahydro-[1,3]dioxolo[4,5-c]pyridine-5(4*H*)-carboxylate (35):** The diol derived from diacetate **32** (2.18 g, 4.50 mmol) was dissolved in a mixture of acetone (40 mL) and 2,2-dimethoxypropane (10 ml) and cooled to 5 ℃. Boron trifluoride diethyl etherate (200 µL,) was added and the reaction stirred on an ice bath during 30 minutes and at room temperature for 18 hours. The reaction was quenched with TEA (2 mL) and diluted with EtOAc (125 mL). The mixture was washed with brine (60 mL), dried with $MgSO_4$, filtered and evaporated. The crude product was purified by silica gel column chromatography (pentane/EtOAc = 99/1→ 97/3→ 95/5) to afford the title compound as a yellow oil (2.01 g, 85%). The TBDPS-protected compound (1.84 g, 3.50 mmol) was dissolved in THF (40 mL) and TBAF.3H_2O (3.42 g, 10.5 mmol, 3.1 eq) was added and the reaction stirred at room temperature for 24 hours. TLC analysis confirmed complete conversion. The mixture was diluted with EtOAc (150 mL) and washed with water (20 mL) and Brine (20 mL), dried ($MgSO_4$), filtered and concentrated. The crude mixture was purified by silica gel column chromatography (pentane/EtOAc = 95/5→ 9/1→ 3/1) to afford compound **35** (0.78 g, 77%). $[\alpha]_D^{23}$ = +2.0 (*c* = 1, $CHCl_3$); HRMS calculated for $[C_{14}H_{25}NO_5 + H]^+$: 288.18055; found: 288.18061. ^1H NMR (400 MHz, $CDCl_3$) δ 4.33 (dd, *J* = 6.8, 5.8 Hz, 1H), 4.16 – 4.10 (m, 1H), 4.08 (dd, *J* = 6.8, 3.1 Hz, 1H), 3.90 (dd, *J* = 6.6, 3.1 Hz, 1H),

3.64 – 3.54 (m, 1H), 3.44 (dd, *J* = 13.5, 3.1 Hz,1H), 2.82 – 2.45 (m 1H), 1.48 (s, 3H), 1.46 (s, 9H), 1.34 (s , 3H), 1.28 (d, *J* = 6.8 Hz, 3H). ^{13}C NMR (101 MHz, $CDCl_3$) δ 156.58, 108.79, 80.26, 77.33, 74.00, 68.39, 47.71, 42.98, 28.52, 26.65, 24.63, 17.56. IR (cm^{-1}) 3500 – 3200, 2929, 1672, 1405, 1381, 1367, 1253, 1215, 1166, 1049, 751.

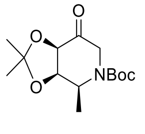

***tert*-Butyl (3a*R*,4*S*,7a*R*)-2,2,4-trimethyl-7-oxotetrahydro-[1,3]dioxolo[4,5-c]pyridine-5(4*H*)-carboxylate:** The alcohol **35** (690 mg, 2.42 mmol) was dissolved in DCM (30 mL) and at 0 ℃ Dess-Martin reagent (1.85 g, 4.35 mmol, 1.8 eq.) was added. The reaction mixture was allowed to warm up to room temperature and stirred overnight. TLC indicated complete conversion and the reaction was quenched with a mixture of saturated aqueous $NaHCO_3$ (30 mL) and saturated aqueous $Na_2S_2O_3$ (30 mL) and stirred for 5 minutes. The mixture was extracted with EtOAc (2 x 50 mL), dried with $MgSO_4$, filtered and concentrated. The crude product was purified by silica gel column chromatography (pentane/EtOAc = 95/5→ 9/1) to afford the title compound (613 mg, 89%). $[\alpha]_D^{23}$ = -13 (*c* = 1, $CHCl_3$); HRMS calculated for $[C_{14}H_{23}NO_5 + H]^+$: 286.16490; found: 286.16488. ^{1}H NMR (400 MHz, $CDCl_3$) δ 4.92 – 4.75 (m, 2H), 4.55 (d, *J* = 9.0 Hz, 1H), 4.51 – 4.29 (m, 1H), 3.74 (d, *J* = 19.2 Hz, 1H), 1.53 (s, 3H), 1.48 (s, 9H), 1.39 (s, 3H), 1.16 (d, *J* = 7.0 Hz, 3H). ^{13}C NMR (101 MHz, $CDCl_3$) δ 204.12, 154.05, 110.92, 81.12, 77.39, 74.47, 50.20, 28.20, 26.05, 24.33, 12.70. IR (cm^{-1}) 2979, 2932, 1699, 1369, 1247, 1219, 1159, 1016, 773, 745.

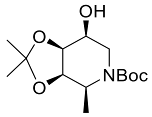

***tert*-Butyl (3a*R*,4*S*,7*S*,7a*S*)-7-hydroxy-2,2,4-trimethyltetrahydro-[1,3]dioxolo[4,5-c]pyridine-5(4*H*)-carboxylate (36):** The ketone from above (456 mg, 1.60 mmol) was dissolved in EtOH (20 mL) and at -78 ℃ $NaBH_4$ (48 mg, 1.26 mmol) was added and the reaction was allowed to warm up slowly over night. TLC indicated complete conversion and the mixture was diluted with EtOAc (50 mL), washed subsequently with water (30 mL) and Brine (20 mL), dried with $MgSO_4$, filtered and evaporated to give a crude product that was purified by silica gel column chromatography (pentane/EtOAc = 3/1→ 1/1→ 0/1) to afford compound **36** (184 mg, 40%). $[\alpha]_D^{23}$ = +2.0 (*c* = 1, $CHCl_3$); HRMS calculated for $[C_{14}H_{25}NO_5 + H]^+$: 288.18056; found: 288.18057. ^{1}H NMR (400 MHz, $CDCl_3$) δ 4.43 – 4.33 (m, 2H), 4.01 (dq, *J* = 6.6, 6.6 Hz, 1H), 3.88 (dd, *J* = 12.0, 3.8 Hz, 1H), 3.61 (ddd, *J* = 10.2, 4.3, 4.3 Hz, 1H), 2.99 (t, *J* = 12.0 Hz, 1H), 2.92 – 2.60 (m, 1H), 1.53 (s, 3H), 1.46 (s, 9H), 1.39 (s, 3H), 1.34 (d, *J* = 6.6 Hz, 3H). ^{13}C NMR (101 MHz, $CDCl_3$) δ 155.13, 108.78, 80.12, 76.03, 72.85, 66.35, 47.44, 41.89, 28.48, 26.42, 24.80, 16.77. IR (cm^{-1}) 3500 – 3200, 2980, 2934, 1688, 1393, 1366, 1251, 1212, 1159, 1023, 867, 773, 734.

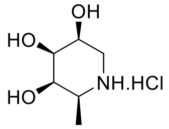

(2*S*,3*R*,4*S*,5*S*)-2-methylpiperidine-3,4,5-triol hydrochloride (7): The Boc-protected-iminosugar **36** (144 mg, 0.50 mmol) was dissolved in a mixture of MeOH (20 mL) and aqueous 6M HCl (3 mL) and stirred over night at room temperature. The mixture was concentrated to afford the title compound **7** as a white foam (92 mg, quant.). $[\alpha]_D^{23}$ = +13 (*c* = 1, MeOH); HRMS calculated for $[C_6H_{13}NO_3 + H]^+$: 148.09682; found: 148.09675. ^{1}H NMR (400 MHz, D_2O) δ 4.20 – 4.16 (m, 1H), 4.00 – 3.97 (m, 1H), 3.83 (t, *J* = 3.3 Hz, 1H), 3.40 (dd, *J* = 13.8, 2.8 Hz, 1H), 3.41 – 3.36 (m, 1H), 3.22 (dd, *J* = 13.8, 1.2 Hz, 1H), 1.36 (d, *J* = 6.8 Hz, 3H). ^{13}C NMR (101 MHz, D_2O) δ 70.46, 67.34, 66.21, 55.03, 48.21, 14.12. IR (cm^{-1}) 3676, 3400 – 3200, 2971, 2925, 1724, 1568, 1148, 1407, 1394, 1250, 1118, 1075, 1066.

*Scheme S3: Preparation of iminosugars **8** and **9**.*

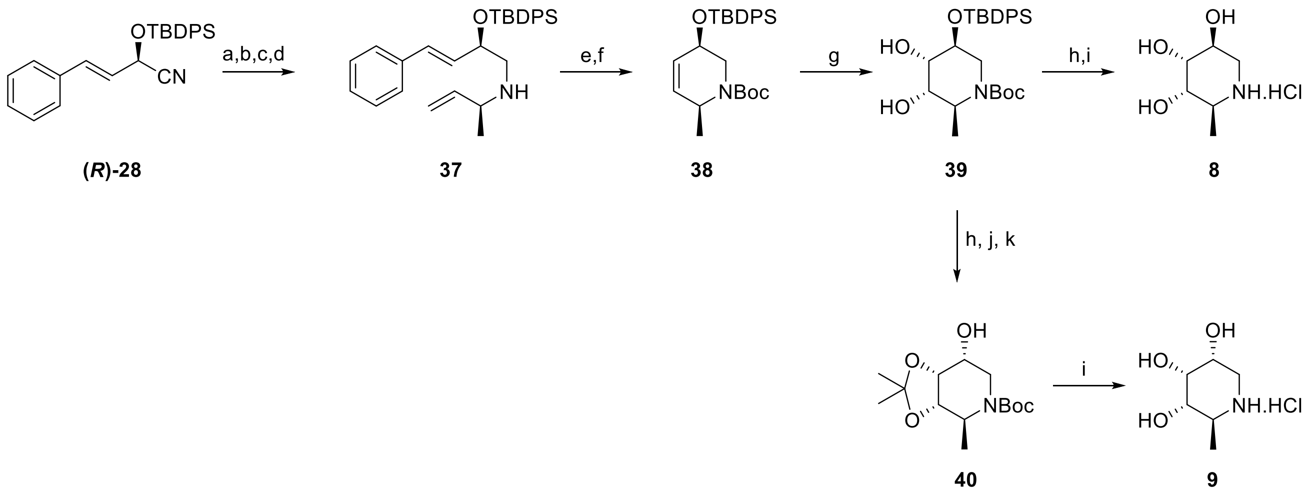

Reagents and conditions: a) Dibal-H, -78 → 5 °C; b) MeOH, -90 °C; c) amine **(*S*)-29**, NaOMe, RT, 18 h; d) $NaBH_4$, 5 °C → RT, 4 h; e) Boc_2O, 50 °C; f) Grubbs I, DCM, reflux; h) $K_2OsO_4.2H_2O$, NMO, acetone/water; h) TBAF, THF; i) 6M HCl, MeOH; j) Dess-Martin, DCM; k) $NaBH_4$, EtOH, -78 ^{0}C → RT.

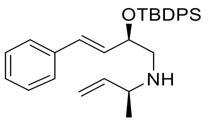

(*R*,*E*)-*N*-((*S*)-But-3-en-2-yl)-2-((*tert*-butyldiphenylsilyl)oxy)-4-phenylbut-3-en-1-amine (37): Prepared as described for compound **30** (18.0 mmol scale, yield 6.79 g, 83%). $[\alpha]_D^{23}$ = -92 (*c* = 1, $CHCl_3$); HRMS calculated for $[C_{30}H_{37}NOSi + H]^+$: 456.27172; found: 456.27144. ^{1}H NMR (400 MHz, $CDCl_3$) δ 7.71 (d, *J* = 7.1 Hz, 2H), 7.68 (d, *J* = 7.1 Hz, 2H), 7.48 - 7.09 (m, 11H), 6.21 (d, *J* = 16.0 Hz, 1H), 6.10 (dd, *J* = 16.0, 6.9 Hz, 1H), 5.57 (ddd, *J* = 17.4, 10.3, 7.8 Hz, 1H), 4.98 (d, *J* = 10.3 Hz, 1H), 4.95 (d, *J* = 7.8 Hz, 1H), 4.46 (dd, *J* = 12.0, 6.3 Hz, 1H), 3.04 (c, *J* = 6.7 Hz, 1H), 2.78 (dd, *J* = 12.0, 6.7 Hz, 1H), 2.59 (dd, *J* = 12.0, 5.2 Hz, 1H), 1.08 (s, 9H), 1.02 (d, *J* = 6.4Hz, 3H). ^{13}C NMR (101 MHz, $CDCl_3$) δ 142.45, 136.76, 136.03, 135.92, 135.36, 134.10, 134.07, 133.96, 131.20, 130.79, 129.72, 129.16, 128.40, 127.65, 127.50, 126.48, 114.51, 74.07, 56.25, 53.64, 27.16, 21.77, 19.43. IR (cm^{-1}) 2931, 2858, 1219, 1112, 772, 702.

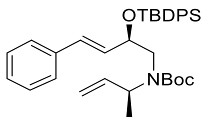

***tert*-Butyl ((*S*)-but-3-en-2-yl)((*R*,*E*)-2-((*tert*-butyldiphenylsilyl)oxy)-4-phenylbut-3-en-1-yl)carbamate:** Prepared as described for **31** (3.90 mmol scale, yield 2.19 g, quant.). $[\alpha]_D^{23}$ = -68 (*c* = 1, $CHCl_3$); HRMS calculated for $[C_{35}H_{45}NO_3Si + H]^+$: 556.32415; found: 556.32436. ^{1}H NMR (400 MHz, $CDCl_3$) δ δ 7.69 (d, *J* = 6.8 Hz, 2H), 7.65 (d, *J* = 6.8 Hz, 2H), 7.48 - 7.09 (m, 11H), 6.17 - 6.00 (m, 2H), 5.71 (ddd, *J* = 16.1, 10.3, 4.8 Hz, 1H), 5.08 - 4.78 (m, 2H), 4.47 - 4.34 (m, 1H), 3.59 - 3.40 (m, 1H), 3.37 - 3.18 (m, 1H), 3.15 - 3.00 (m, 1H), 1.42 - 1.22 (m, 9H), 1.07 (s, 9H), 1.06 - 1.04 (m, 3H). ^{13}C NMR (101 MHz, $CDCl_3$) δ 155.61, 143.50, 136.15, 136.05, 135.68, 131.05, 130.89, 129.81, 129.71, 128.02, 127.94, 127.73, 127.58, 126.58, 114.89, 79.64, 73.93, 50.39, 41.47, 28.44, 27.21, 19.44, 17.40. IR (cm^{-1}) 3072, 2932, 2858, 1693, 1266, 1167, 1113, 1070, 741, 702.

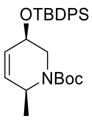

***tert*-Butyl (3*R*,6*S*)-3-((*tert*-butyldiphenylsilyl)oxy)-6-methyl-3,6-dihydropyridine-1(2*H*)-carboxy-late (38):** Prepared as described for **32**. Compound **38** was obtained as a clear oil (3.80 mmol scale, yield 1.65 g, 96%). $[\alpha]_D^{23}$ = +22 (*c* = 1, $CHCl_3$); HRMS calculated for $[C_{27}H_{37}NO_3Si + H]^+$: 452.26155; found: 452.26185. ^{1}H NMR (400 MHz, $CDCl_3$) δ 7.75 - 7.60 (m, 4H), 7.47 - 7.32 (m, 6H), 5.76 - 5.37 (m, 2H), 4.44 - 4.28 (m, 1H), 4.28 - 4.15 (m, 1H), 4.09 – 3.88 (m, 1H), 2.80 - 2.67 (m, 1H), 1.34 (s, 9H), 1.16 (d, *J* = 6.6 Hz, 3H), 1.08 (s, 9H). ^{13}C NMR (101 MHz, $CDCl_3$) δ 153.93, 135.91, 135.80, 135.46, 130.86, 130.57, 129.88, 129.78, 128.31, 127.79, 127.57, 126.52, 79.61, 65.63, 47.42, 43.99, 28.48, 27.06, 19.31, 17.85. IR (cm^{-1}) 3072, 2932, 2858, 1697, 1453, 1366, 1162, 1112, 763, 741, 702.

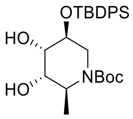

***tert*-Butyl (2*S*,3*S*,4*S*,5*S*)-5-((*tert*-butyldiphenylsilyl)oxy)-3,4-dihydroxy-2-methylpiperidine-1-carboxylate (39):** Prepared as described in the Upjohn procedure concerning compound **32**. Compound **37** was obtained as a clear oil (1.12 mmol scale, yield 430 mg, 79%). $[\alpha]_D^{23}$ = +21 (*c* = 1, $CHCl_3$); HRMS calculated for $[C_{27}H_{39}NO_5Si + H]^+$: 486.26703; found: 486.26723. ^{1}H NMR (400 MHz, $CDCl_3$) δ 7.70 (dd, *J* = 7.9, 1.5 Hz, 2H), 7.68 (dd, *J* = 7.9, 1.5 Hz, 2H), 7.48 - 7.36 (m, 6H), 4.55 - 4.29 (m, 1H), 4.29 - 3.96 (m, 1H), 3.90 (td, J = 10.3, 5.4 Hz, 1H), 3.84 - 3.74 (m, 1H), 3.71 (dd, *J* = 8.8, 2.9 Hz, 1H), 2.77 (dd, *J* = 13.2, 10.7 Hz, 1H), 2.38 - 2.00 (m, 2H), 1.34 (s, 9H), 1.13 (d, *J* = 7.3 Hz, 3H), 1.09 (s, 9H). ^{13}C NMR (101 MHz, $CDCl_3$) δ 155.32, 135.92, 135.81, 133.75, 130.16, 130.13, 128.03, 127.97, 80.03, 73.46, 72.52, 70.24, 48.03, 41.78, 28.42, 27.14, 19.46, 14.18. IR (cm^{-1}) 3020, 1215, 1111, 770, 748, 668.

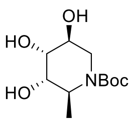

***tert*-Butyl (2*S*,3*S*,4*R*,5*S*)-3,4,5-trihydroxy-2-methylpiperidine-1-carboxylate:** The TBDPS-ether **39** (1.91 g, 3.94 mmol) was dissolved in THF (40 ml) and TBAF.$3H_2O$ (3.46 g, 11.0 mmol) was added at room temperature. The reaction was stirred at ambient temperature overnight. TLC indicated complete conversion and the mixture was concentrated. The crude compound was purified by silica gel column chromatography (pentane/EtOAc = 1/1 → 0/1 → EtOAc) to afford the title compound as a colorless oil (957 mg, 98%). $[\alpha]_D^{23}$ = +38 (*c* = 1, $CHCl_3$); HRMS calculated for $[C_{11}H_{21}NO_5 + H]^+$: 248.14925; found: 248.14924. ^{1}H NMR (400 MHz, CD_3OD) δ 4.35 (m, 1H), 4.08 (dd, *J* = 13.0, 5.4 Hz, 1H), 3.83 – 3.69 (m, 2H), 3.55 (dd, *J* = 9.5, 3.1 Hz, 1H), 2.74 – 2.61 (m, 1H), 1.46 (s, 9H), 1.15 (d, *J* = 7.3 Hz, 3H). ^{13}C NMR (101 MHz, CD_3OD) δ 157.27, 81.38, 73.99, 73.38, 68.19, 56.32, 45.39, 28.84, 14.45. IR (cm^{-1}) 3020, 1215, 770, 747.

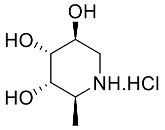

(2*S*,3*S*,4*R*,5*S*)-2-methylpiperidine-3,4,5-triol hydrochloride (8): Prepared as described for iminosugar **6**. Boc-iminosugar from above was used and **8** was obtained as a white foam (2.68 mmol scale, yield 445 mg, 90%). $[\alpha]^{23}_{D}$ = -10 (*c* = 1, MeOH); HRMS calculated for [$C_6H_{13}NO_3$ + H]$^+$: 148.09682; found: 148.09672. ^{1}H NMR[13] (400 MHz, D_2O) δ 4.14 (ddd, *J* = 4.9, 3.3, 3.3 Hz, 1H), 4.00 (dd, *J* = 4.1, 3.1 Hz, 1H), 3.85 (dd, *J* = 9.9, 3.1 Hz, 1H), 3.39 (dq, *J* = 9.9, 6.6 Hz, 1H), 3.33 (dd, *J* = 13.6, 1.9 Hz, 1H), 3.17 (dd, *J* = 13.6, 3.1 Hz, 1H), 1.39 (d, *J* = 6.6 Hz, 3H). ^{13}C NMR (101 MHz, D_2O) 68.45, 68.09, 66.32, 51.07, 43.88, 12.72. IR (cm^{-1}) 3369, 3271, 3022, 2952, 1727, 1583, 1437, 1260, 1086, 1069, 1051, 965, 701.

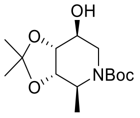

***tert*-Butyl (3a*S*,4*S*,7*S*,7a*R*)-7-hydroxy-2,2,4-trimethyltetrahydro-[1,3]dioxolo[4,5-c]pyridine-5(4*H*)-carboxylate:** Prepared as described en route towards **35** (scheme 2, steps b, c). The title compound was obtained as a clear oil (4.80 mmol scale, yield 1.25 g, 91%). $[\alpha]^{23}_{D}$ = +48 (*c* = 1, $CHCl_3$); HRMS calculated for [$C_{14}H_{25}NO_5$ + H]$^+$: 288.18055; found: 288.18053. ^{1}H NMR (400 MHz, $CDCl_3$) δ 4.68 – 4.52 (m, 1H), 4.05 (d, *J* = 5.4 Hz, 1H), 4.00 (t, *J* = 6.3 Hz, 1H), 3.93 (dd, *J* = 13.5, 4.7 Hz, 1H), 3.76 (m, 1H), 2.92 (broad s, 1H), 2.82 (dd, *J* = 13.5, 10.2 Hz, 1H), 1.48 (s, 3H), 1.47 (s, 9H), 1.35 (s, 3H), 1.20 (d, *J* = 7.3Hz, 3H). ^{13}C NMR (101 MHz, $CDCl_3$) δ 155.43, 108.88, 80.31, 78.10, 77.85, 69.50, 48.18, 41.46, 28.48, 28.22, 26.20, 16.86. IR (cm^{-1}) 3020, 1215, 748, 668.

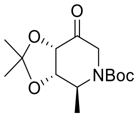

***tert*-Butyl (3a*S*,4*S*,7a*S*)-2,2,4-trimethyl-7-oxotetrahydro-[1,3]dioxolo[4,5-c]pyridine-5(4*H*)-carboxylate:** Prepared as described en route towards **36** (scheme 2, step d). The title ketone was obtained as a white solid (0.50 mmol scale, yield 108 mg, 76%). $[\alpha]^{23}_{D}$ = -2.4 (*c* = 1, $CHCl_3$); HRMS calculated for [$C_{14}H_{23}NO_5$ + H]$^+$: 286.16490; found: 286.16478. ^{1}H NMR (400 MHz, $CDCl_3$) δ 4.76 – 4.57 (m, 2H), 4.36 (d, *J* = 6.6 Hz, 1H), 4.30 (d, *J* = 6.6 Hz, 1H), 3.66 (d, *J* = 18.5 Hz, 1H), 1.48 (s, 9H), 1.46 (s, 3H), 1.35 (s , 3H), 1.16 (d, *J* = 7.5 Hz, 3H). ^{13}C NMR (101 MHz, $CDCl_3$) δ 203.20, 157.70, 111.69, 80.86, 80.25, 75.54, 50.94, 49.23, 26.67, 26.18, 24.98, 15.47. IR (cm^{-1}) 2980, 2935, 1737, 1693, 1408, 1367, 1221, 1157, 1049, 867.

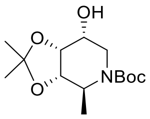

***tert*-Butyl (3a*S*,4*S*,7*R*,7a*R*)-7-hydroxy-2,2,4-trimethyltetrahydro-[1,3]dioxolo[4,5-c]pyridine-5(4*H*)-carboxylate (40):** Prepared as described for alcohol **36** from the ketone described above. Alcohol **40** was obtained as a mixture of two diastereoisomers (ratio 93:7, 0.42 mmol scale, yield 104 mg, 86%). $[\alpha]^{23}_{D}$ = +42 (*c* = 1, $CHCl_3$); HRMS calculated for [$C_{14}H_{25}NO_5$ + H]$^+$: 288.18056; found: 288.18063. ^{1}H NMR (400 MHz, $CDCl_3$) δ 4.43 (dd, *J* = 6.8, 4.3 Hz, 1H), 4.33 (dd, *J* = 7.4, 2.0 Hz, 1H), 4.27 – 4.19 (m, 1H), 3.96 (ddd, *J* = 11.3, 4.5, 4.5 Hz, 1H), 3.61 (dd, *J* = 11.7, 4.4 Hz, 1H), 3.08 (t, *J* = 11.7 Hz, 1H), 2.68 – 2.48 (m, 1H), 1.50 (s, 3H), 1.47 (s, 9H), 1.38 (s, 3H), 1.17 (d, *J* = 7.2 Hz, 3H). ^{13}C NMR (101 MHz, $CDCl_3$) δ 155.14, 108.85, 79.82, 77.35, 71.79, 65.03, 47.68, 42.46, 28.49, 26.23, 24.37, 19.10. IR (cm^{-1}) 3437, 2978, 2935, 1683, 1401, 1369, 1255, 1212, 1169, 1049, 877, 731.

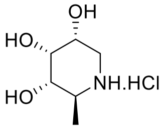

(2*S*,3*S*,4*R*,5*R*)-2-methylpiperidine-3,4,5-triol hydrochloride (9): Prepared as described for iminosugar **6** from Boc-protected-iminosugar **38**. Iminosugar **8** was obtained as a colorless foam (1.70 mmol scale, yield 311 mg, quant.) in a d.r. of 93:7. *N*-Boc-protection, column chromatography and subsequent deprotection (HCl/MeOH) afforded the diastereomerically pure compound **9**. $[\alpha]^{23}_{D}$ = -20 (*c* = 1, MeOH); HRMS calculated for [$C_6H_{13}NO_3$ + Na]$^+$: 170.07876; found: 170.07865. ^{1}H NMR (400 MHz, D_2O) δ 4.19 – 4.12 (m, 1H), 4.00 (ddd, *J* = 11.5, 4.9, 2.6 Hz, 1H) 3.62 (dd, *J* =10.4, 2.5 Hz, 1H), 3.33 (dq, *J* = 4.9, 4.9 Hz, 1H), 3.25 (dd, *J* = 12.0, 4.9 Hz, 1H), 3.09 (t, *J* = 12.0 Hz, 1H), 1.37 (d, *J* = 6.6 Hz, 3H). ^{13}C NMR (101 MHz, D_2O) δ 69.95, 69.61, 64.72, 49.92, 41.65, 14.16. IR (cm^{-1}) 3400 - 3200, 2939, 2804, 1456, 1158, 1106, 1072, 1043, 1018, 996, 962, 816, 707.

Scheme S4: Preparation of iminosugar ***10***.

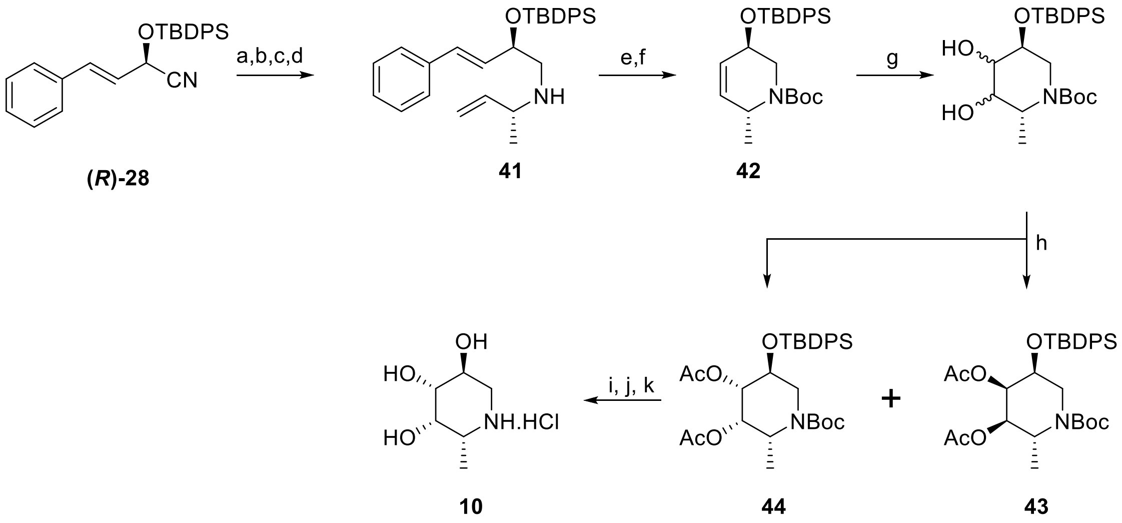

Reagents and conditions: a) Dibal-H, -78 → 5 °C; b) MeOH, -90 °C; c) amine **(*R*)-29**, NaOMe, RT, 18 h; d) $NaBH_4$, 5 °C → RT, 4 h; e) Boc_2O, 50 °C; f) Grubbs I, DCM, reflux; g) $K_2OsO_4.2H_2O$, NMO, acetone/water; h) Ac_2O, pyridine, DMAP, 0 °C → RT; i) K_2CO_3, MeOH; j) TBAF, THF; k) 6M HCl, MeOH.

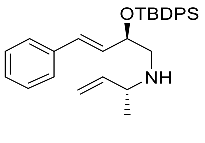

(*R*,*E*)-*N*-((*R*)-But-3-en-2-yl)-2-((*tert*-butyldiphenylsilyl)oxy)-4-phenylbut-3-en-1-amine (41): Prepared as described for compound **30**. (35.0 mmol scale, yield 13.2 g, 83%). $[\alpha]_D^{23}$ = -109 (*c* = 1, $CHCl_3$); HRMS calculated for $[C_{30}H_{37}NOSi + H]^+$: 456.27172; found: 456.27139. ^{1}H NMR (400 MHz, $CDCl_3$) δ 7.72 - 7.62 (m, 4H), 7.43 - 7.15 (m, 11H), 6.20 (d, *J* = 16.0 Hz, 1H), 6.10 (dd, *J* = 16.0, 7.0 Hz, 1H), 5.60 (ddd, *J* = 16.8, 10.2, 7.0 Hz, 1H), 5.01 (d, *J* = 16.8 Hz, 1H), 4.97 (d, *J* = 10.2 Hz, 1H), 4.44 (dt, *J* = 12.6, 6.2 Hz, 1H), 3.10 (m, 1H), 2.72 (dd, *J* = 11.8, 6.3 Hz, 1H), 2.69 (dd, *J* = 11.8, 5.6 Hz, 1H), 1.08 (s, 9H), 1.05 (t, *J* = 6.8 Hz, 3H). ^{13}C NMR (101 MHz, $CDCl_3$) δ 142.46, 136.88, 136.14, 136.06, 135.49, 134.24, 134.14, 131.40, 130.83, 129.79, 129.69, 128.51, 127.71, 127.59, 126.61, 114.69, 74.45, 56.72, 53.77, 27.25, 21.58, 19.52. IR (cm^{-1}) 3500 - 3200, 2967, 1653, 1111, 1055, 1033, 1015, 741, 700.

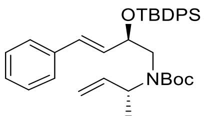

***tert*-Butyl ((*R*)-but-3-en-2-yl)((*R*,*E*)-2-((*tert*-butyldiphenylsilyl)oxy)-4-phenylbut-3-en-1-yl)carbamate:** Prepared as described for **29** (28.9 mmol scale, yield 16.0 g, 99%). $[\alpha]_D^{23}$ = -25 (*c* = 1, $CHCl_3$); HRMS calculated for $[C_{35}H_{45}NO_3Si + H]^+$: 556.32415; found: 556.32416. ^{1}H NMR (400 MHz, $CDCl_3$) δ 7.78 - 7.62 (m, 4H), 7.47 - 7.10 (m, 11H), 6.15 - 6.05 (m, 2H), 5.78 (ddd, *J* = 16.6, 10.6, 5.2 Hz, 1H), 5.00 - 4.86 (m, 2H), 4.55 - 4.38 (m, 1H), 3.59 - 3.37 (m, 1H), 3.37 - 3.20 (m, 1H), 3.20 - 3.04 (m, 1H), 1.40 - 1.20 (m, 9H), 1.08 (s, 9H), 1.08 - 1.04 (m, 3H). ^{13}C NMR (101 MHz, $CDCl_3$) δ 155.57, 146.87, 136.14, 135.12, 134.92, 131.13, 130.84, 129.78, 128.42, 127.68, 126.56, 79.64, 73.60, 50.16, 43.52, 28.45, 27.54, 19.43, 17.62. IR (cm^{-1}) 2977, 2933, 2858, 1808, 1757, 1691, 1396, 1370, 1212, 1166, 1113, 1065, 739, 701.

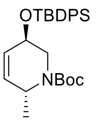

***tert*-Butyl (3*R*,6*R*)-3-((*tert*-butyldiphenylsilyl)oxy)-6-methyl-3,6-dihydropyridine-1(2*H*)-carboxylate(42):** Prepared as described for compound **32** from the Boc-protected diene mentioned above. Compound **42** was obtained as a colorless oil (8.41 mmol scale, yield 3.63 g, 95%). $[\alpha]_D^{23}$ = -153 (*c* = 1, $CHCl_3$); HRMS calculated for $[C_{27}H_{37}NO_3Si + H]^+$: 452.26155; found: 452.26155. ^{1}H NMR (400 MHz, $CDCl_3$) δ 7.72 (d, *J* = 7.7 Hz), 7.66 (d, *J* = 7.7 Hz, 2H), 7.46 - 7.32 (m, 6H), 5.71 - 5.62 (m, 1H), 5.58 - 5.47 (m, 1H), 4.64 - 4.48 (m, 1H), 4.28 - 4.09 (m, 1H), 4.07 - 4.00 (m, 1H), 2.96 - 2.76 (m, 1H), 1.50 (s, 9H), 1.08 (d, *J* = 6.9 Hz, 3H), 1.05 (s, 9H). ^{13}C NMR (101 MHz, $CDCl_3$) δ 154.80, 135.94, 134.12, 129.80, 129.65, 127.78, 127.58, 79.45, 64.21, 46.44, 28.63, 27.03, 19.35, 17.27. IR (cm^{-1}) 2978, 2933, 2858, 1808, 1757, 1691, 1470, 1212, 1166, 1113, 1065, 739, 701.

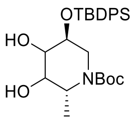

***tert*-Butyl (2*R*,5*S*)-5-((tert-butyldiphenylsilyl)oxy)-3,4-dihydroxy-2-methylpiperidine-1-carboxylate:** The procedure described for the Upjohn dihydroxylation of compound **32** afforded a 3 : 1 mixture of inseparable diastereoisomers (27.1 mmol scale, yield 10.2 g, 78%). To separate these diastereoisomers the mixture was directly converted into the diacetates.

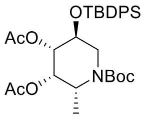

(2*R*,3*S*,4*S*,5*S*)-1-(*tert*-butoxycarbonyl)-5-((*tert*-butyldiphenylsilyl)oxy)-2-methylpiperidine-3,4-diyl diacetate (44): Prepared as described for **34**. Compound **44** was obtained as a pale yellow oil (20.9 mmol scale, yield 7.23 g, 61%). $[\alpha]_D^{23}$ = -10 (*c* = 1, $CHCl_3$); HRMS calculated for $[C_{31}H_{43}NO_7Si + H]^+$: 570.28816; found: 570.28791. ^1H NMR (400 MHz, $CDCl_3$) δ 7.73 – 7.67 (m, 4H), 7.46 – 7.35 (m, 6H), 5.44 (dd, *J* = 6.9, 3.2 Hz, 1H), 5.12 (dd, *J* = 3.2, 3.6 Hz, 1H), 4.54 (qd, *J* = 6.9, 6.9 Hz, 1H), 3.94 (d, *J* = 14.3 Hz, 1H), 3.77 (dd, *J* = 3.6, 1.5 Hz, 1H), 3.08 (dd, *J* = 14.3, 1.5 Hz, 1H) 2.03 (s, 3H), 1.92 (s, 3H), 1.47 (s, 9H), 1.20 (d, *J* = 6.9 Hz, 3H**)**, 1.09 (s, 9H). ^{13}C NMR (101 MHz, $CDCl_3$) δ 169.75, 169.33, 154.87, 135.95, 135.91, 133.11, 133.04, 130.01, 129.92, 127.83, 127.78, 80.00, 70.98, 68.67, 67.39, 48.07, 40.83, 28.46, 26.92, 20.97, 20.92, 19.25, 12.48. IR (cm^{-1}) 3073, 2933, 2859, 1750, 1694, 1417, 1365, 1237, 1218, 1160, 1026, 753, 740, 701.

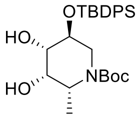

***t*ert-Butyl (2*R*,3*S*,4*S*,5*S*)-5-((*tert*-butyldiphenylsilyl)oxy)-3,4-dihydroxy-2-methylpiperidine-1-carboxylate:** Prepared as described en route towards fuconononojirimycin (**6**) and was obtained as a colorless oil (12.3 mmol scale, yield 5.52 g, 85%). $[\alpha]_D^{23}$ = +5.4 (*c* = 1, $CHCl_3$); HRMS calculated for $[C_{27}H_{39}NO_5Si + H]^+$: 486.26703; found: 486.26708. ^1H NMR (400 MHz, $CDCl_3$) δ 7.68 (dd, *J* = 7.8, 1.4 Hz, 2H), 7.64 (dd, *J* = 7.8, 1.4 Hz, 2H), 7.45 - 7.32 (m, 6H), 4.48 (qd, *J* = 6.9, 6.9 Hz, 1H), 4.11 (dd, *J* = 6.4, 3.2 Hz, 1H), 3.89 - 3.80 (m, 2H), 3.74 (t, *J* = 3.1 Hz, 1H), 3.12 (dd, *J* = 14.5, 1.7 Hz, 1H), 2.81 (broad s, 1H), 2.44 (broad s, 1H), 1.44 (s, 9H), 1.19 (d, *J* = 7.1 Hz, 3H**),** 1.07 (s, 9H). ^{13}C NMR (101 MHz, $CDCl_3$) δ 155.45, 135.95, 135.80, 133.81, 133.36, 129.97, 127.83, 79.84, 72.36, 70.61, 66.48, 49.81, 39.62, 28.57, 27.06, 19.32, 12.03. IR (cm^{-1}) 3500 – 3200, 2932, 2859, 1663, 1426, 1365, 1156, 1093, 1017, 753, 701.

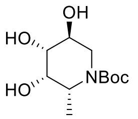

***tert*-Butyl (2*S*,3*R*,4*S*,5*R*)-3,4,5-trihydroxy-2-methylpiperidine-1-carboxylate:** Prepared as described for alcohol **35** and was obtained as a colorless oil (3.80 mmol scale, yield 860 mg, 92%). $[\alpha]_D^{23}$ = -20 (*c* = 1, $CHCl_3$); HRMS calculated for $[C_{11}H_{21}NO_5 + H]^+$: 248.14925; found: 248.14933. ^1H NMR (400 MHz, CD_3OD) δ 4.28 (dq, *J* = 6.9, 6.9 Hz, 1H), 3.86 (dd, *J* = 6.9, 2.3 Hz, 1H), 3.85 – 3.75 (m, 3H), 3.27 (dd, *J* = 14.2, 1.5 Hz, 1H), 1.46 (s, 9H), 1.24 (d, *J* = 6.9 Hz, 3H). ^{13}C NMR (101 MHz, CD_3OD) δ 157.57, 81.14, 73.37, 70.95, 67.53, 52.23, 40.96, 28.90, 12.72. IR (cm^{-1}) 3400 – 3200, 2978, 2931, 1660, 1421, 1366, 1157, 1068, 907, 729.

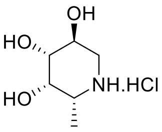

(2*R*,3*S*,4*R*,5*S*)-2-methylpiperidine-3,4,5-triol hydrochloride (10): Prepared as described for **5** and was obtained as a white foam (3.0 mmol scale, yield 549 mg, quant.). $[\alpha]_D^{23}$ = +31 (*c* = 1, MeOH); HRMS calculated for $[C_6H_{13}NO_3 + H]^+$: 148.09682; found: 148.09658. ^1H NMR (400 MHz, D_2O) δ 4.07 – 3.98 (m, 2H), 3.64 (dd, *J* = 9.9, 3.0 Hz, 1H), 3.50 – 3.40 (m, 2H), 2.85 (app. t, *J* = 12.0 Hz, 1H), 1.35 (d, *J* = 6.7 Hz, 3H). ^{13}C NMR (101 MHz, D_2O) δ 73.07, 69.79, 64.33, 54.96, 46.05, 14.05. IR (cm^{-1}) 3400 – 3200, 2939, 2805, 1583, 1444, 1386, 1159, 1074, 1022, 999, 708.

*Scheme S5: Preparation of iminosugar **11**.*

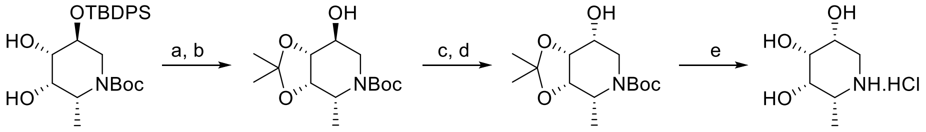

45 **46** **11**

Reagents and conditions: a) Acetone / 2,2-dimethoxypropane, $BF_3.EtO_2$, 5 ^0C; b) TBAF, THF; c) Dess-Martin, DCM; d) $NaBH_4$, EtOH, -78 ^0C → RT; e) 6M HCl, MeOH.

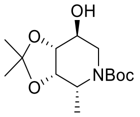

***tert*-Butyl (3a*S*,4*R*,7*S*,7a*R*)-7-hydroxy-2,2,4-trimethyltetrahydro-[1,3]dioxolo[4,5-c]pyridine-5(4*H*)-carboxylate (45):** Prepared as described for alcohol **33** from the silyl ether mentioned above (4.60 mmol scale, yield 1.20 g, 85% for two steps). $[\alpha]_D^{23}$ = -3.4 (*c* = 1, $CHCl_3$); HRMS calculated for $[C_{14}H_{25}NO_5 + H]^+$: 288.18055; found: 288.18059. ^1H NMR (400 MHz, $CDCl_3$) δ 4.34 (dd, *J* = 6.9, 5.7 Hz, 1H), 4.13 (qd, *J* = 6.6, 6.6 Hz, 1H),

4.08 (dd, *J* = 6.5, 3.3 Hz, 1H), 3.96 (ddd, *J* = 6.5, 6.5, 3.3 Hz, 1H), 3.60 (dd, *J* = 13.2, 6.5 Hz, 1H), 3.44 (dd, *J* = 13.2, 3.2 Hz, 1H), 2.55 – 2.36 (m 1H), 1.48 (s, 3H), 1.47 (s, 9H), 1.35 (s , 3H), 1.28 (d, *J* = 6.8 Hz, 3H). ^{13}C NMR (101 MHz, $CDCl_3$) δ 158.48, 108.84, 80.26, 77.38, 73.99, 68.53, 47.74, 42.97, 28.54, 26.67, 24.66, 17.56. IR (cm^{-1}) 3500 – 3200, 2980, 2934, 1670, 1403, 1367, 1253, 1212, 1166, 1056, 868, 773.

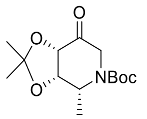

***tert*-Butyl (3a*S*,4*R*,7a*S*)-2,2,4-trimethyl-7-oxotetrahydro-[1,3]dioxolo[4,5-c]pyridine-5(4*H*)-carboxylate:** Prepared as described en route towards compound **36** (scheme 2, step d). The title compound was obtained as a white solid (3.93 mmol scale, yield 919 mg, 82 %). $[\alpha]_D^{23}$ = +13 (*c* = 1, $CHCl_3$); HRMS calculated for $[C_{14}H_{23}NO_5 + H]^+$: 286.16490; found: 286.16488. ^{1}H NMR (400 MHz, $CDCl_3$) δ 4.89 – 4.71 (m, 2H), 4.52 (d, *J* = 9.0 Hz, 1H), 4.49 – 4.27 (m, 1H), 3.75 (d, *J* = 19.2 Hz, 1H), 1.51 (s, 3H), 1.49 (s, 9H), 1.39 (s , 3H), 1.16 (d, *J* = 7.0 Hz, 3H). ^{13}C NMR (101 MHz, $CDCl_3$) δ 204.28, 154.21, 111.07, 81.28, 80.25, 75.55, 74.62, 50.35, 48.12, 28.35, 26.20, 24.49, 12.86. IR (cm^{-1}) 2979, 2935, 1741, 1696, 1409, 1368, 1381, 1162, 1081, 1031, 875.

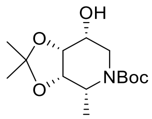

***tert*-Butyl (3a*S*,4*R*,7*R*,7a*R*)-7-hydroxy-2,2,4-trimethyltetrahydro-[1,3]dioxolo[4,5-c]pyridi-ne-5(4*H*)-carboxylate (46):** Prepared as described for **36** from the ketone mentioned above. Alcohol **46** was obtained (3.78 mmol scale, yield 610 mg, 56%). $[\alpha]_D^{23}$ = -4.0 (*c* = 1, $CHCl_3$); HRMS calculated for $[C_{14}H_{25}NO_5 + H]^+$: 288.18056; found: 288.18058. ^{1}H NMR (400 MHz, $CDCl_3$) δ 4.44 – 4.34 (m, 2H), 4.06 – 3.96 (m, 1H), 3.88 (dd, *J* = 12.0, 3.8 Hz, 1H), 3.68 – 3.56 (m, 1H), 2.99 (t, *J* = 12.0 Hz, 1H), 2.79 – 2.61 (m 1H), 1.53 (s, 3H), 1.46 (s, 9H), 1.39 (s, 3H), 1.34 (d, *J* = 6.6 Hz, 3H). ^{13}C NMR (101 MHz, $CDCl_3$) δ 155.14, 108.79, 80.13, 76.06, 72.83, 66.36, 47.44, 41.92, 28.49, 26.43, 24.81, 16.78. IR (cm^{-1}) 3500 – 3200, 2979, 2934, 1688, 1406, 1393, 1367, 1250, 1221, 1156, 1061, 1046, 1033, 867.

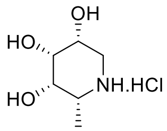

(2*R*,3*S*,4*R*,5*R*)-2-methylpiperidine-3,4,5-triol hydrochloride (11): Prepared as described for **6** from the protected iminosugar **46**. The title compound **10** was obtained as a white foam (1.50 mmol scale, yield 275 mg, quant.). $[\alpha]_D^{23}$ = -16 (*c* = 1, MeOH); HRMS calculated for $[C_6H_{13}NO_3 + Na]^+$: 170.07876; found: 170.07864. ^{1}H NMR (400 MHz, D_2O) δ 4.2 (ddd, *J* = 4.5, 2.8, 1.4 Hz, 1H), 4.00 – 3.97 (m, 1H), 3.84 (t, *J* = 3.5 Hz, 1H), 3.44 (dd, *J* = 13.7, 2.8 Hz, 1H), 3.42 – 3.37 (m, 1H), 3.23 (dd, *J* = 13.7, 1.4 Hz, 1H), 1.36 (d, *J* = 6.8 Hz, 3H). ^{13}C NMR (101 MHz, D_2O) δ 70.48, 67.33, 66.22, 55.05, 48.26, 14.20. IR (cm^{-1}) 3676, 3348, 3130, 2971, 1559, 1443, 1312, 1277, 1249, 1121, 1056, 1007, 988.

*Scheme S6: Preparation of iminosugars **12** and **13**.*

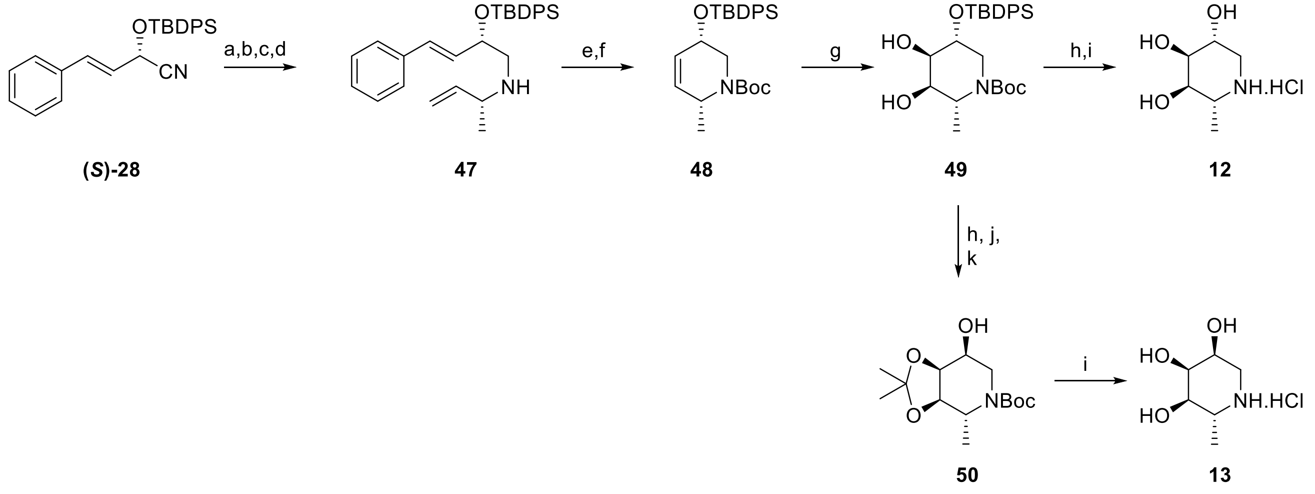

Reagents and conditions: a) Dibal-H, -78 → 5 °C; b) MeOH, -90 °C; c) amine **(*R*)-29**, NaOMe, RT, 18 h; d) $NaBH_4$, 5 °C → RT, 4 h; e) Boc_2O, 50 °C; f) Grubbs I, DCM, reflux; h) $K_2OsO_4.2H_2O$, NMO, acetone/water; h) TBAF, THF; i) 6M HCl, MeOH; j) Dess-Martin, DCM; k) $NaBH_4$, EtOH, -78 ^{0}C → RT.

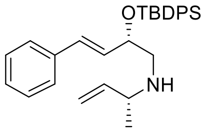

(*S*,*E*)-*N*-((*R*)-But-3-en-2-yl)-2-((*tert*-butyldiphenylsilyl)oxy)-4-phenylbut-3-en-1-amine (47): Preparation as described for compound **30** from **(*S*)-27** (3.61 g, 9.09 mmol) and **(*R*)-29.HCl** (3.17 g, 29.7 mmol, 3.3 eq.). afforded the target compound (3.63 g, 87 %). $[\alpha]_D^{23}$ = +95 (*c* = 1, $CHCl_3$); HRMS calculated for $[C_{30}H_{37}NOSi + H]^+$: 456.27172; found: 456.27147. ^1H NMR (400 MHz, $CDCl_3$) δ 7.74 – 7.62 (m, 4H) 7.44 – 7.13 (m, 11H), 6.20 (d, *J* = 16.0 Hz, 1H), 6.11 (dd, *J* = 16.0, 6.9 Hz, 1H), 5.57 (ddd, *J* = 17.4, 10.5, 7.5 Hz, 1H), 4.98 (d, *J* = 17.4 Hz, 1H), 4.96 (d, *J* = 10.5 Hz, 1H), 4.43 (td, *J* = 6.8, 5.1 Hz, 1H), 3.04 (m, 1H), 2.78 (dd, *J* = 11.9, 6.7 Hz, 1H), 2.59 (dd, *J* = 11.9, 5.2 Hz, 1H), 1.65, (broad s, 1H), 1.08 (s, 9H), 1.02 (d, *J* = 6.4Hz, 3H). ^{13}C NMR (101 MHz, $CDCl_3$) δ 142.57, 136.86, 136.10, 136.00, 134.99, 134.19, 134.06, 131.24, 130.91, 129.77, 129.66, 128.47, 127.70, 127.55, 126.56, 114.55, 74.13, 56.32, 53.72, 27.21, 21.84, 19.49. IR (cm^{-1}) 3053, 2957, 2930, 2857, 1471, 1428, 1111, 772, 701.

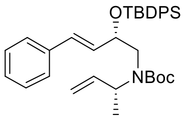

***tert*-Butyl ((*R*)-but-3-en-2-yl)((*S*,*E*)-2-((*tert*-butyldiphenylsilyl)oxy)-4-phenylbut-3-en-1-yl)carbamate:** Prepared as described for compound **31** (3.70 mmol scale in 98% yield). $[\alpha]_D^{23}$ = +76 (*c* = 1, $CHCl_3$); HRMS calculated for $[C_{35}H_{45}NO_3Si + H]^+$: 556.32415; found: 556.32427. ^1H NMR (400 MHz, $CDCl_3$) δ 7.71 (d, *J* = 6.8 Hz, 2H), 7.65 (d, *J* = 6.8 Hz, 2H), 7.48 - 7.16 (m, 11H), 6.19 - 6.00 (m, 2H), 5.78 - 5.64 (m, 1H), 5.06 - 4.80 (m, 2H), 4.47 - 4.32 (m, 1H), 3.59 - 3.39 (m, 1H), 3.39 - 3.20 (m, 1H), 3.14 - 3.00 (m, 1H), 1.43 - 1.19 (m, 9H), 1.07 (s, 9H), 1.05 (d, *J* = 2.5 Hz, 3H). ^{13}C NMR (101 MHz, $CDCl_3$) δ 139.08, 136.15, 136.05, 135.51, 131.02, 130.87, 129.81, 129.71, 128.44, 128.36 127.73, 127.58, 126.56, 114.92, 79.63, 73.92, 50.33, 47.00, 28.43, 27.55, 19.43, 17.40. IR (cm^{-1}) 3057, 2931, 2858, 1691, 1391, 1365, 1165, 1110, 1069, 740, 700.

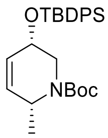

***tert*-Butyl (3*S*,6*R*)-3-((*tert*-butyldiphenylsilyl)oxy)-6-methyl-3,6-dihydropyridine-1(2*H*)-carboxylate (48):** Prepared as described for compound **32** (3.60 mmol scale, 1.61 g, 98%). $[\alpha]_D^{23}$ = -12 (*c* = 1, $CHCl_3$); HRMS calculated for $[C_{27}H_{37}NO_3Si + H]^+$: 452.26155; found: 452.26158. ^1H NMR (400 MHz, $CDCl_3$) δ 7.74 - 7.63 (m, 4H), 7.46 - 7.32 (m, 6H), 5.72 - 5.42 (m, 2H), 4.44 - 4.28 (m, 1H), 4.28 - 4.16 (m, 1H), 3.98 (dd, *J* = 12.5, 5.6 Hz, 1H), 2.79 - 2.68 (m, 1H), 1.35 (s, 9H), 1.16 (d, *J* = 6.7 Hz, 3H), 1.08 (s, 9H). ^{13}C NMR (101 MHz, $CDCl_3$) δ 153.92, 135.79, 134.91, 130.84, 130.55, 129.87, 128.77, 127.78, 126.59, 79.65, 65.60, 47.40, 43.96, 28.44, 27.21, 19.44, 17.82. IR (cm^{-1}) 2963, 2930, 2858, 1697, 1427, 1410, 1365, 1157, 1109, 1059, 740.

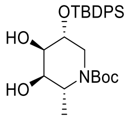

***tert*-Butyl (2*R*,3*R*,4*R*,5*R*)-5-((*tert*-butyldiphenylsilyl)oxy)-3,4-dihydroxy-2-methylpiperidine-1-carboxylate (49):** Compound **48** (6.90 g, 15.4 mmol) was dissolved in a mixture of acetone (70 mL) and water (70 mL) and cooled to -10 ℃. *N*-Methylmorpholine-*N*-oxide monohydrate (6.7 g, 49.8 mmol) and $K_2OsO_4.2H_2O$ (60.0 mg, 0.162 mmol, 1.05 mol %) were added subsequently. After 24 - 48 hours TLC analysis showed complete conversion of the starting material **48**. The reaction was quenched with an aqueous saturated Na_2SO_3 solution (100 mL) and stirred for 30 min. The mixture was diluted with water (100 mL) and extracted with EtOAc (3 x 60 mL). The combined organic layers were washed with 0.6 M HCl, saturated $NaHCO_3$ and brine. After drying (Na_2SO_4), filtering and evaporation of the solvent, the silicagel column chromatography (pentane/EtOAc = 9/1→ 3/1→ 1/1) afforded the compound **49** as a colorless oil (6.40 g, 86%). $[\alpha]_D^{23}$ = -34 (*c* = 1, $CHCl_3$); HRMS calculated for $[C_{27}H_{39}NO_5Si + H]^+$: 486.26703; found: 486.26736. ^1H NMR (400 MHz, $CDCl_3$) δ 7.71 (dd, *J* = 7.9, 1.5 Hz, 2H), 7.66 (dd, *J* = 7.9, 1.5 Hz, 2H), 7.48 - 7.34 (m, 6H), 4.56 - 4.26 (m, 1H), 4.26 - 3.95 (m, 1H), 3.90 (td, *J* = 10.3, 5.2 Hz, 1H), 3.83 - 3.73 (m, 1H), 3.71 (dd, *J* = 8.8, 3.0 Hz, 1H), 2.77 (dd, *J* = 13.3, 10.3 Hz, 1H), 2.40 - 2.09 (m, 2H), 1.49 - 1.19 (s, 9H), 1.13 (d, *J* = 7.3 Hz, 3H), 1.09 (s, 9H). ^{13}C NMR (101 MHz, $CDCl_3$) δ 155.32, 135.90, 135.81, 133.73, 130.13, 130.10, 128.00, 127.95, 80.03, 73.41, 72.50, 70.21, 48.65, 43.18, 28.40, 27.13, 19.45 , 14.15. IR (cm^{-1}) 3300, 3020, 2254, 1683, 1112, 904, 725.

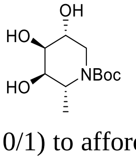

***tert*-Butyl (2*R*,3*R*,4*S*,5*R*)-3,4,5-trihydroxy-2-methylpiperidine-1-carboxylate:** The silyl ether **49** (1.72 g, 3.55 mmol) was dissolved in THF (30 mL) and $TBAF.3H_2O$ (3.50 g, 11.1 mmol) was added at room temperature. The reaction was stirred at ambient temperature overnight. TLC indicated complete conversion and the mixture was concentrated. The crude compound was purified by silica gel column chromatography (pentane/EtOAc = 1/1 → 0/1) to afford the title compound as a clear oil (794 mg, 91%). $[\alpha]_D^{23}$ = -40 (*c* = 1, $CHCl_3$); HRMS calculated for $[C_{11}H_{21}NO_5 + H]^+$: 248.14925; found: 248.14920. ^1H NMR (400 MHz, CD_3OD) δ 4.36 (m, 1H), 4.09 (dd, *J* = 13.2, 5.6 Hz, 1H), 3.78 – 3.71 (m, 2H), 3.55 (dd, *J* = 9.6, 2.4 Hz, 1H), 2.67 (app. t, *J* = 12.5 Hz, 1H), 1.46 (s, 9H), 1.16 (d, *J* = 7.2 Hz, 3H). ^{13}C NMR (101 MHz, CD_3OD) δ 157.28, 81.41, 74.02, 73.41, 68.22, 54.77, 46.40, 28.85, 14.45. IR (cm^{-1}) 3400 - 3200, 2977, 2932, 1665, 1419, 1366, 1167, 1074, 731.

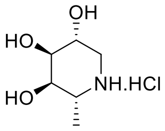

(2*R*,3*R*,4*S*,5*R*)-2-methylpiperidine-3,4,5-triol hydrochloride (12): *N*-Boc-protected-iminosugar from above (502 mg, 2.03 mmol) was dissolved in a mixture of MeOH (20 mL) and 6M HCl (3 mL) and the reaction was stirred overnight at room temperature. TLC indicated complete conversion and the mixture was concentrated to afford the title iminosugar **12** as a white foam (317 mg, 86%). $[\alpha]_D^{23}$ = +13 (*c* = 1, MeOH); HRMS calculated for $[C_6H_{13}NO_3 + H]^+$: 148.09682; found: 148.09674. ^{1}H NMR[13] (400 MHz, D_2O) δ 4.16 (dd, *J* = 6.4, 4.8 Hz, 1H), 4.02 (dd, *J* = 3.2, 3.2 Hz, 1H), 3.88 (dd, *J* = 9.8, 3.2 Hz, 1H), 3.42 (dq, *J* = 9.8, 6.7 Hz, 1H), 3.36 (m, 1H), 3.20 (dd, *J* = 13.4, 3.2 Hz, 1H), 1.47 (d, *J* = 6.7 Hz, 3H); ^{13}C NMR (101 MHz, D_2O) 68.50, 68.15, 66.36, 51.16, 43.95, 14.26 (**C**H_3-CH). IR (cm^{-1}) 3271, 3022, 2951, 2914, 1583, 1438, 1260, 1086, 1069, 1051, 965.

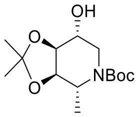

***tert*-Butyl (3a*R*,4*R*,7*R*,7a*S*)-7-hydroxy-2,2,4-trimethyltetrahydro-[1,3]dioxolo[4,5-c]pyridine-5(4*H*)-carboxylate:** Preparation from TBDPS-ether **49** as described en route towards compound **40** (9.40 mmol scale), afforded the title compound (2.27 g, 84% over two steps). $[\alpha]_D^{23}$ = -38 (*c* = 0.3, $CHCl_3$); ^{1}H NMR (400 MHz, $CDCl_3$) δ 4.64 – 4.54 (m, 1H), 4.05 (d, *J* = 5.8 Hz, 1H) 4.00 (app. t, *J* = 5.8 Hz, 1H), 3.93 (dd, *J* = 13.6, 4.9 Hz, 1H), 3.76 (ddd, *J* = 10.2, 6.4, 4.9 Hz, 1H), 2.53 – 2.19 (broad s, 1H), 2.82 (dd, J = 13.6, 10.2 Hz, 1H), 1.49 – 1.45 (m, 12H), 1.35 (s, 3H), 1.26 (d, *J* = 7.2 Hz, 3H). ^{13}C NMR (101 MHz, $CDCl_3$) δ 155.42, 108.92, 80.31, 78.13, 77.90, 69.67, 48.21, 41.48, 28.52, 28.24, 26.22, 16.91. IR (cm^{-1}) 3500 – 3200, 2979, 2929, 1694, 1671, 1413, 1367, 1166, 1059, 873.

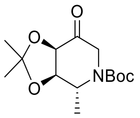

***tert*-Butyl (3a*R*,4*R*,7a*R*)-2,2,4-trimethyl-7-oxotetrahydro-[1,3]dioxolo[4,5-c]pyridine-5(4*H*)-carboxylate:** Preparation from TBDPS-ether **49** as described en route towards compound **40** (6.90 mmol scale), afforded the title compound (1.49 g, 76%). $[\alpha]_D^{23}$ = +2.2 (*c* = 1, $CHCl_3$); ^{1}H NMR (400 MHz, $CDCl_3$) δ 4.75 – 4.55 (m, 2H), 4.36 (dd, *J* = 6.8, 1.4 Hz, 1H), 4.30 (d, *J* = 6.8 Hz, 1H), 3.65 (d, *J* = 18.5 Hz, 1H), 1.48 (s, 9H), 1.46 (s, 3H), 1.35 (s, 3H), 1.16 (d, *J* = 7.5 Hz, 3H). ^{13}C NMR (101 MHz, $CDCl_3$) δ 203.12, 154.74, 111.62, 80.78, 80.22, 75.51, 54.68, 49.15, 28.49, 26.64, 24.95, 15.43. IR (cm^{-1}) 3426, 2981, 2837, 1773, 1728, 1695, 1369, 1252, 1214, 1150, 1050, 870, 753.

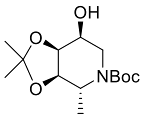

***tert*-Butyl (3a*R*,4*R*,7*S*,7a*S*)-7-hydroxy-2,2,4-trimethyltetrahydro-[1,3]dioxolo[4,5-c]-pyridine-5(4*H*)-carboxylate (50):** Prepared as described for alcohol **40** from the ketone mentioned above (4.30 mmol scale, yield 1.02 g, 83%, d.r. = 93:7). $[\alpha]_D^{23}$ = -35 (*c* = 1, $CHCl_3$); HRMS calculated for $[C_{14}H_{25}NO_5 + H]^+$: 288.18056; found: 288.18056. ^{1}H NMR (400 MHz, $CDCl_3$) δ 4.42 (dd, *J* = 7.3, 4.4 Hz, 1H), 4.33 (dd, *J* = 7.3, 1.8 Hz, 1H), 4.28 – 4.19 (m, 1H), 3.95 (ddd, *J* = 10.7, 4.4, 4.4 Hz, 1H), 3.61 (dd, *J* = 11.9, 4.4 Hz, 1H), 3.07 (app. t, *J* = 11.9 Hz, 1H), 2.55 – 2.36 (m 1H), 1.50 (s, 3H), 1.47 (s, 9H), 1.38 (s, 3H), 1.17 (d, *J* = 7.2 Hz, 3H). ^{13}C NMR (101 MHz, $CDCl_3$) δ 155.21, 108.95, 79.91, 77.42, 71.80, 65.15, 47.70, 42.56, 28.56, 26.30, 24.45, 19.16. IR (cm^{-1}) 3432, 2979, 2936, 1739, 1687, 1401, 1366, 1167, 1049, 877, 750.

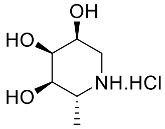

(2*R*,3*R*,4*S*,5*S*)-2-methylpiperidine-3,4,5-triol hydrochloride (13): Prepared as described for **6** from protected iminosugar **50**, to afford **13** as a colorless foam (3.40 mmol scale, yield 613 mg, 98%) in a d.r. of 93:7. $[\alpha]_D^{23}$ = +21 (*c* = 1, MeOH); HRMS calculated for $[C_6H_{13}NO_3 + Na]^+$: 148.09682; found: 148.09658. ^{1}H NMR (400 MHz, D_2O) δ 4.13 – 4.17 (m, 1H), 3.96 (ddd, *J* = 11.5, 4.9, 2.6 Hz, 1H) 3.57 (dd, *J* =10.4, 2.5 Hz, 1H), 3.28 (dq, *J* = 6.5, 2.5 Hz, 1H), 3.20 (dd, *J* = 12.0, 4.9 Hz, 1H), 3.05 (app. t, *J* = 12.0 Hz, 1H), 1.33 (d, *J* = 6.5 Hz, 3H). ^{13}C NMR (101 MHz, D_2O) δ 69.93, 69.61, 64.70, 49.08, 41.22, 14.16. IR (cm^{-1}) 3400 - 3200, 2942, 2810, 2512, 1457, 1044, 1016, 962, 818, 707.

5. Biological assays

5.1

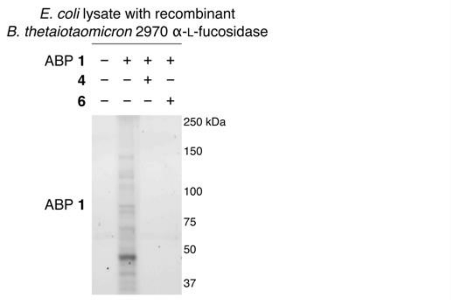

Fig. S1. *In vitro* activity-based protein profiling of GH29 α-L-fucosidase. Labeling of lysate of *E. coli* expressing recombinant α-L-fucosidase from *Bacteroides thetaiotaomicron* 2970 with ABP **1**.

5.2

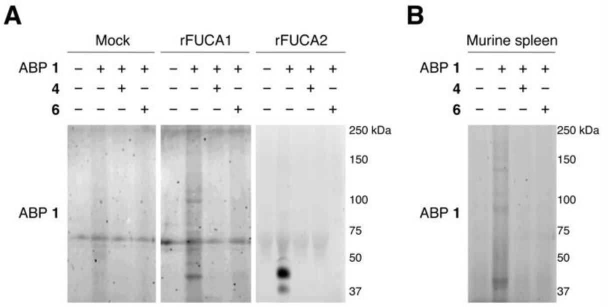

Fig. S2. *In vitro* activity-based protein profiling of GH29 α-L-fucosidase. A) ABP **1** labeling of α-L-fucosidases present in lysate of COS-7 cells transfected with empty plasmid (Mock) or plasmid encoding human FUCA1 or FUCA2. B) Labeling of tissue homogenate of wild-type murine spleen with ABP **1**.

5.3

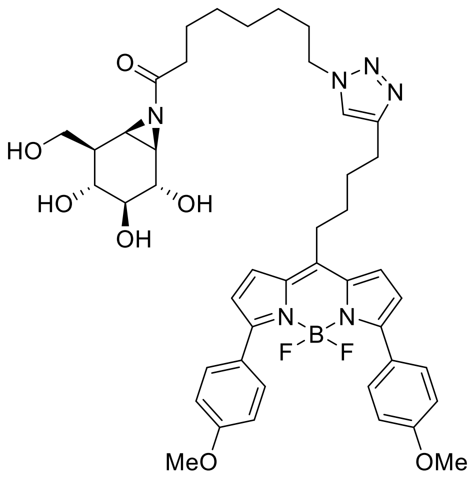

Fig. S3. Structure of JJB75.

5.4

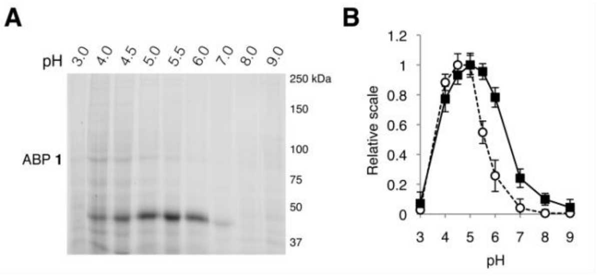

Fig. S4. *In vitro* pH profile of recombinant FUCA1. A) *In vitro* labeling of COS-7 cell lysate containing over-expressed human FUCA1 at various pH with ABP **1**. B) Relative *in vitro* labeling of FUCA1 with ABP **1** (closed squares) compared to relative enzymatic activity towards artificial 4-methylumbelliferyl-α-L-fucopyranoside substrate (open circles) at various pH. Data average of n = 3 experiments, ± standard deviation.

5.5

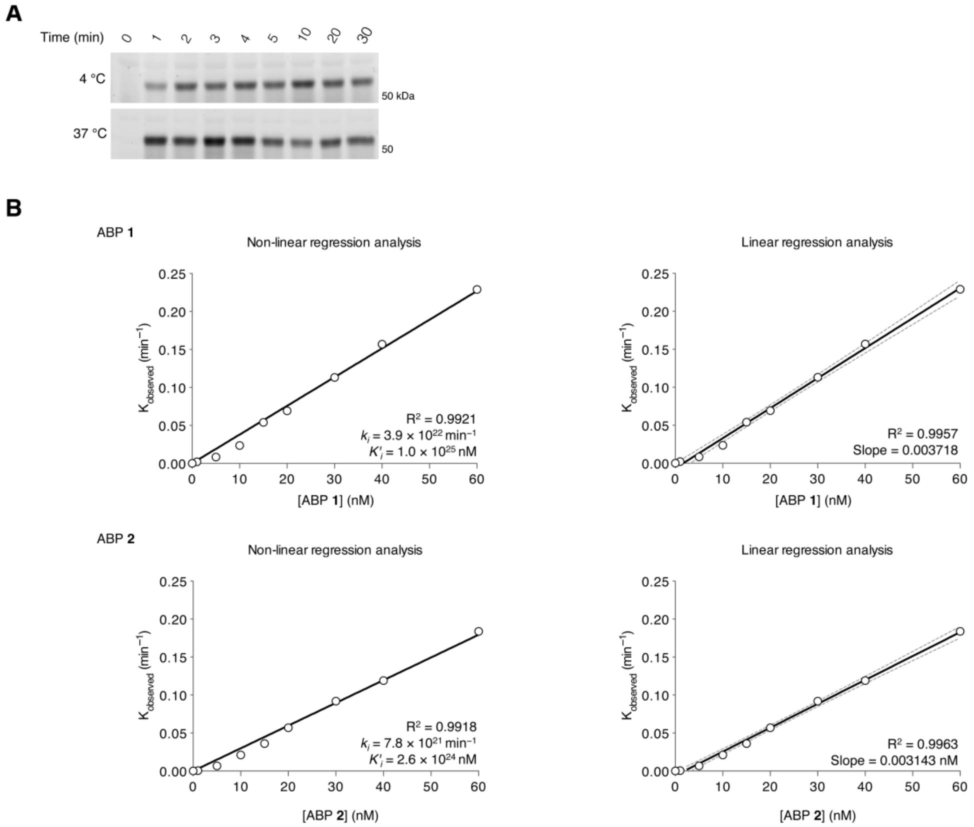

Fig. S5. *In vitro* inhibition kinetics of ABPs **1** and **2** towards recombinant human FUCA1. A) Stoichiometric *in situ* labeling of COS-7 lysate containing over-expressed human FUCA1 with 10 nM ABP **1** at 4 ℃ and 37 ℃. B) Plots of $K_{observed}$ against the concentration of ABP **1** and **2**. Rates determined through direct kinetic measurements of liberated 4-methylumbelliferone from 4-methylumbelliferyl-α-L-fucopyranoside substrate whilst in the presence of varying concentrations of ABP **1** and **2**.

5.6

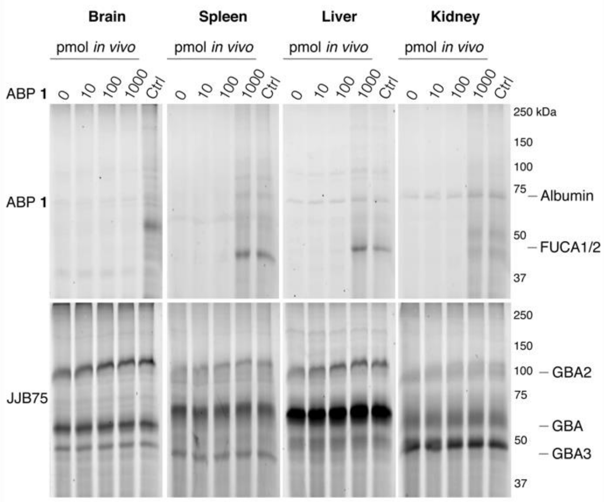

Fig. S6. *In vivo* labeling of α-L-fucosidases in mice with various concentrations of ABP **1** during 2 hours. Top: *in vivo* labeling compared to maximal *in situ* labeling with excess ABP **1** of matched homogenates of untreated animals (Ctrl). Bottom: *in vitro* labeling of retaining β-glucosidases GBA, GBA2 and GBA3 with JJB75 in all homogenates as loading control.

5.7

Table S1(A):

Proteins identified by affinity purification with the biotinylated Probe **3**(JJB243, on bead digest, LC-MS/MS analysis and Mascot search engine match to the human Uniprot database (jan 2015).

nr	accession	protein description	prot score	prot mass	prot cover	emPAI
1	P04264	Keratin, type II cytoskeletal 1, KRT1	677	66	18	0,62
2	P13645	Keratin, type I cytoskeletal 10, KRT10	580	59	22	0,92
3	P05165	Propionyl-CoA carboxylase, mitochondrial, PCCA	541	80	17	0,38
4	P35527	Keratin, type I cytoskeletal 9, KRT9	374	62	23	0,44
5	P35908	Keratin, type II cytoskeletal 2 epidermal, KRT2	366	65	10	0,34
6	P11498	Pyruvate carboxylase, mitochondrial, PC	262	130	8	0,19
7	P04066	Tissue alpha-L-fucosidase, FUCA1	210	54	17	0,35
8	H0YA55	Serum albumin, ALB	192	52	5	0,2
9	U3KQK0	Histone H2B, HIST1H2BN	191	19	9	0,39
10	H7C469	Uncharacterized protein	144	28	12	0,25
11	H0YFX9	Histone H2A, HISTH2A	142	10	21	0,82
12	P19013	Keratin, type II cytoskeletal 4, KRT4	132	57	4	0,12
13	Q14956	Transmembrane glycoprotein NMB, GPNMB	131	64	3	0,11
14	P81605	Dermcidin OS, DCD	96	113	13	0,70
15	F8W6P5	LVV-hemorphin-7, HBB	93	10	26	0,85
16	P31025	Lipocalin-1, LCN1	71	19	6	0,18
17	P69905	Hemoglobin subunit alpha, HBA1	71	15	37	0,83
18	A5A3E0	POTE ankyrin domain family member F, POTEF	70	121	2	0,03
19	F5GWP8	Keratin, type I cytoskeletal 17, KRT17	69	40	5	0,17

Table S1(B):

Proteins identified after competitive experiment between the fluorescent fucosidase probe **1** and the biotin probe **3**, followed by affinity purification, on bead digest, LC-MS/MS analysis and Mascot search engine match to the human UniProt database (jan 2015)

nr	accession	protein description	prot score	prot mass	prot cover	emPAI
1	P05165	Propionyl-CoA carboxylase, mitochondrial, PCCA	426	80	22	0,69
2	P13645	Keratin, type I cytoskeletal 10, KRT10	418	59	21	1,03
3	E9PS68	Pyruvate carboxylase, mitochondrial, PC	276	32	16	0,47
4	Q14956	Transmembrane glycoprotein NMB, GPNMB	236	64	3	0,11
5	A0A087WY73	Proline-rich protein 4, PRR4	227	17	14	0,44
6	P35908	Keratin, type II cytoskeletal 2 epidermal, KRT2	218	65	12	0,48
7	P04264	Keratin, type II cytoskeletal 1, KRT1	218	66	12	0,4
8	P35527	Keratin, type I cytoskeletal 9, KRT9	213	62	11	0,29
9	U3KQK0	Histone H2B, HIST1H2BN	147	19	9	0,39
10	H0YI76	Keratin, type II cytoskeletal 5, KRT5	126	23	12	0,31
11	H7C469	Uncharacterized protein	97	28	5	0,12
12	H0YFX9	Histone H2A, H2A	84	10	21	0,35
13	P81605	Dermcidin, DCD	64	113	10	0,3
14	A8MUF7	Hemoglobin subunit epsilon, HBE1	48	10	12	0,37
15	P69905	Hemoglobin subunit alpha, HBA1	47	15	11	0,22
16	F8W0V3	Extracellular glycoprotein lacritin, LACRT	43	13	6	0,26

Table S1(C):

Proteins identified after no probe control experiment, aspecific background stickiness of proteins to the paramagnetic beads, followed by affinity purification, on bead digest, LC-MS/MS analysis and Mascot search engine match to the human UniProt database (jan 2015)

nr	accession	protein description	prot score	prot mass	prot cover	emPAI
1	P04264	Keratin, type II cytoskeletal 1, KRT1	1166	66	35	1,91
2	P35527	Keratin, type I cytoskeletal 9, KRT9	972	62	39	1,67
3	P05165	Propionyl-CoA carboxylase, mitochondrial, PCCA	701	80	28	0,98
4	P35908	Keratin, type II cytoskeletal 2 epidermal, KRT2	427	65	20	0,71
5	P13645	Keratin, type I cytoskeletal 10, KRT10	342	59	12	0,46
6	P02533	Keratin, type I cytoskeletal 14, KRT14	341	52	18	0,54
7	P13647	Keratin, type II cytoskeletal 5, KRT5	283	62	13	0,43
8	P11498	Pyruvate carboxylase, mitochondrial, PC	282	130	7	0,16
9	P02538	Keratin, type II cytoskeletal 6A, KRT6A	270	60	12	0,38
10	P48668	Keratin, type II cytoskeletal 6C, KRT6C	257	60	12	0,38
11	P08779	Keratin, type I cytoskeletal 16, KRT16	252	51	15	0,55
12	P04259	Keratin, type II cytoskeletal 6B, KRT6B	222	60	9	0,31
13	Q7Z794	Keratin, type II cytoskeletal 1b, KRT77	179	62	4	0,11
14	P81605	Dermcidin, DCD	136	113	23	1,22
16	F8W6P5	LVV-hemorphin-7, HBB	82	10	36	1,52
17	Q14956	Transmembrane glycoprotein NMB, GPNMB	73	64	3	0,11
18	P31025	Lipocalin-1, LCN1	48	19	6	0,18

19	P69905	Hemoglobin subunit alpha, HBA1	47	15	11	0,22
20	A0A075B6N7	Ig alpha-2 chain C region, IGHA2	46	37	3	0,09
21	Q5T8M7	Actin, alpha skeletal muscle, ACTA1	43	38	3	0,09

Protein score is the Mascot score calculated for the peptide matches of the protein to the human protein database, protein mass is given in kDa, protein coverage is the percentage of the amino acid sequence that has been identified, emPAI value gives an indication of the abundancy or relative concentration of the protein in the LC-MS run.

5.8

Table S2: Analysis parameters of peptides derived from the P04066 Tissue alpha-L-fucosidase, FUCA1 protein after affinity purification, on bead digest and LC-MS/MS analysis

start-end	m/z obs	z	ppm	Ion score	Sequence
114-130	982.4723	2	2	75	FFHPEEWADLFQAAGAK
163-173	572.3213	2	-1	58	DLVGELGTALR
392-420	1049.211	3	0	84	GSAVYAIFLHWPENGVLNLESPITTSTTK
439-461	828.1304	3	1	49	GLFISLPQLPPSAVPAEFAWTIK

Start-end gives the position of the identified peptide in the protein sequence, m/z observed is the measured m/z of the peptide, z is the charge, ppm is the measurement accuracy between the calculated and the observed m/z, ion score is the Mascot search engine score calculated for the match of the MS/MS fragmentation to the human protein database, sequence is the identified peptide sequence.

5.9

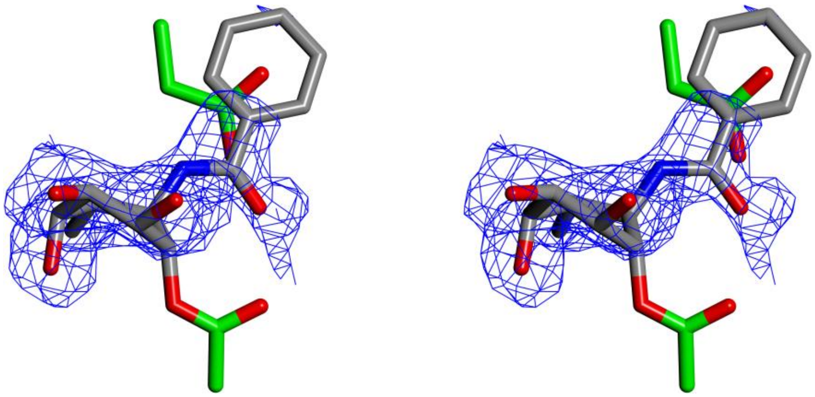

Fig. S7. Crystal structure of α-L-fucosidase from *Bacteroides thetaiotaomicron* in complex with **4**. Electron density displayed is F_o-F_c density from phases calculated prior to the inclusion of **4** in refinement, contoured at 2σ. Figure was prepared using CCP4MG[14]. Note that there is no electron density, at this level, for the aryl group presumably reflecting considerable disorder and/or steric clashes.

5.10

Table S3. X-ray crystallographic data table

	*Bt*Fuc2970-**5**	*Bt*Fuc2970-**4**
**Data collection**		
Beamline/Date	Diamond i03 18/10/14	Diamond i03 02/02/14
Wavelength (Å)	0.97625	0.97625
Cell dimensions		
a, *b*, *c* (Å)	55.5, 187.0, 98.2	55.6, 186.5, 98.2
α, β, γ (°)	90, 94.2, 90	90, 94.2, 90
Resolution (Å)	93.5-1.64	62.17-1.92
R_{merge}	0.058(0.62)	0.095(0.86)*
$I / \sigma I$	11.2(2.0)	7.0(1.8)
Completeness (%)	98.1(98.5)	96.9(96.7)
Redundancy	4.1(4.3)	4.0(3.7)
Wilson B value	20.4	26.1
**Refinement**		
Resolution (Å)	97.9-1.64	97.9-1.71
No. reflections	328440	292076
R_{work} / R_{free}	0.16/0.19	0.18/0.23
R.m.s. deviations		
Bond lengths (Å)	0.019	0.019
Bond angles (°)	1.78	1.81
Ramachandran Statistics (%)		
Preferred	96.7	95.8
Allowed	2.4	3.1
Outliers	0.9	1.1
PDB codes	4WSJ	4WSK

Values in parentheses are for highest-resolution shell.

6. References

(1) D. A. Evans, K. T. Chapman and J. Bisaha, *J. Am. Chem. Soc.* **1988**, 110, 1238-1256.
(2) F. G. Hansen, E. Bundgaard and R. Madsen, *J. Org. Chem.* **2005**, 70, 10139-10142.
(3) O. David, W. J. N. Meester, H. Bieräugel, H. E. Schoemaker, H. Hiemstra and J. H. van Maarseveen, *Angew. Chem., Int. Ed.* 2003, **42**, 4373-4375.
(4) M. D. Witte, W. W. Kallemeijn, J Aten, K. Y. Li, A. Srtijland, W. E. Donker-Koopman, A. M. C. H. van den Nieuwendijk, B. Bleijlevens, G. Kramer, B. I. Florea, B. Hooibrink, C. E. M. Hollak, R. Ottenhoff, R. G. Boot, G. A. van der Marel, H. S. Overkleeft and J. M. F. G. Aerts, *Nat. Chem. Biol.* 2010, **6**, 907- 913.
(5) H. Griengl, N. Klempier, P. Poechlauer, M. Schmidt, N. Shi, A.A. Zabelinskaja-Mackova *Tetrahedron*, 1998, **54**, 14477 -14486.
(6) E. Blocka, M. J. Bosiak, M. Welniak, A. Ludwiczak, A. Wojtczak *Tetrahedron: Asymm.* 2014, **25**, 554-562.
(7) Y. Hamashima, D. Sawada, H. Nogami, M. Kanai, M. Shiwasaki *Tetrahedron*, 2001, **57**, 805-814.
(8) E. G. J. C. Warmerdam, A. M. C. H. van den Nieuwendijk, C. G. Kruse, J. Brussee, A. van der Gen *Recl. Trav. Chim. Pays-Bas*, 1996 , **115**, p. 20-24.
(9) N. Kurono, T. Yoshikawa, M. Yamasaki, T. Ohkuma, *Org. Lett.*, 2011 , **13**, 1254-1257.
(10) A. D. Campbell, T. M. Raynham, R. J. K. Taylor *Synthesis* 1998, 1707-1709.
(11) T. Moriwake, S. Hamano, S. Saito, S. Torii, *Chem. Lett.* 1987, 2085-2088.
(12) M. Pallavicini, E. Valoti, L. Villa, O. Piccolo *Tetrahedron: Asymm.* 2000, **11**, 4017-4025.
(13) J. Streith, A. Boiron, J-L. Paillaud, E-M. Rodriguez-Perez, C. Strehler, T. Tschamber, M. Zehnder, *Helv. Chim. Acta* 1995, **78**, 61-72.
(14) S. Mcnicholas, E. Potterton, K. S. Wilson and M. E. M. Noble, *Acta Crystallogr., Sect. D: Biol.*, 2011, **67**, 386-394.

^{1}H and ^{13}C NMR ($CDCl_3$)

(*R*,*E*)-3-(But-2-enoyl)-4-isopropyloxazolidin-2-one (**14**)

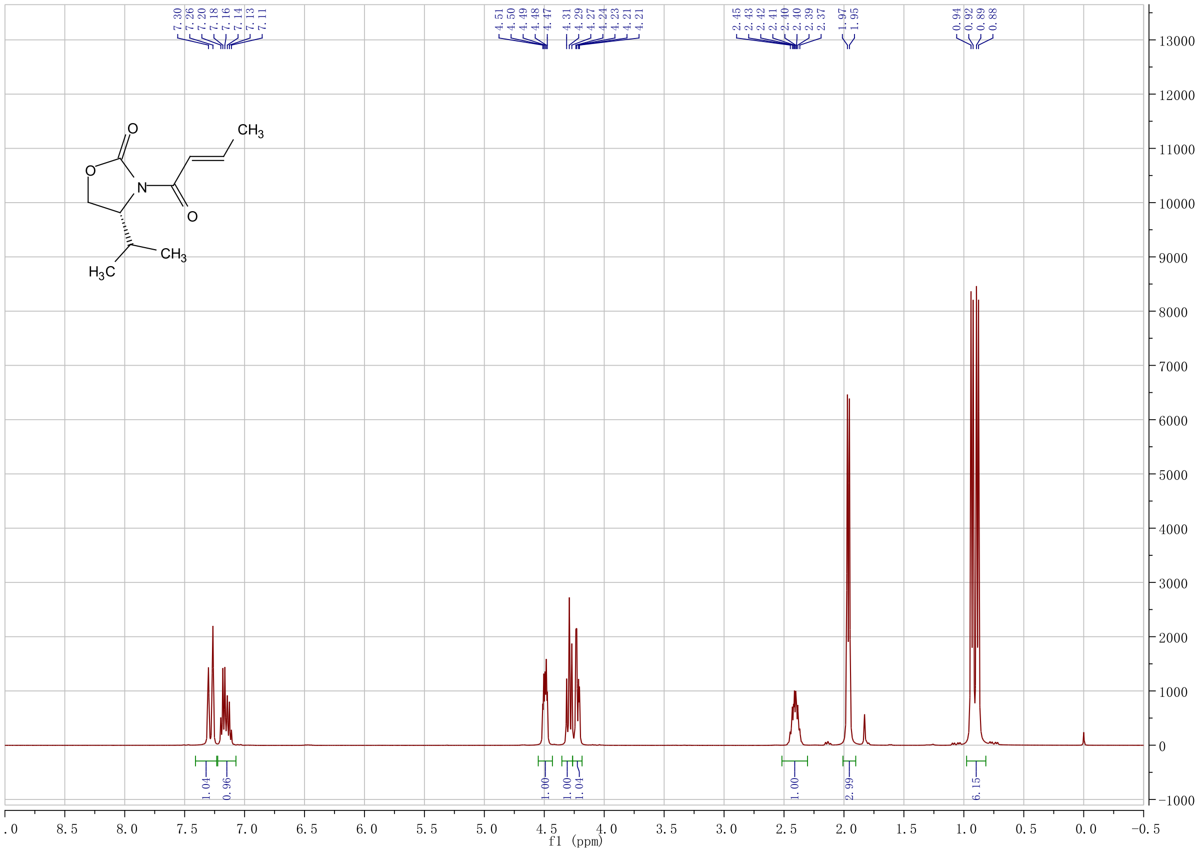

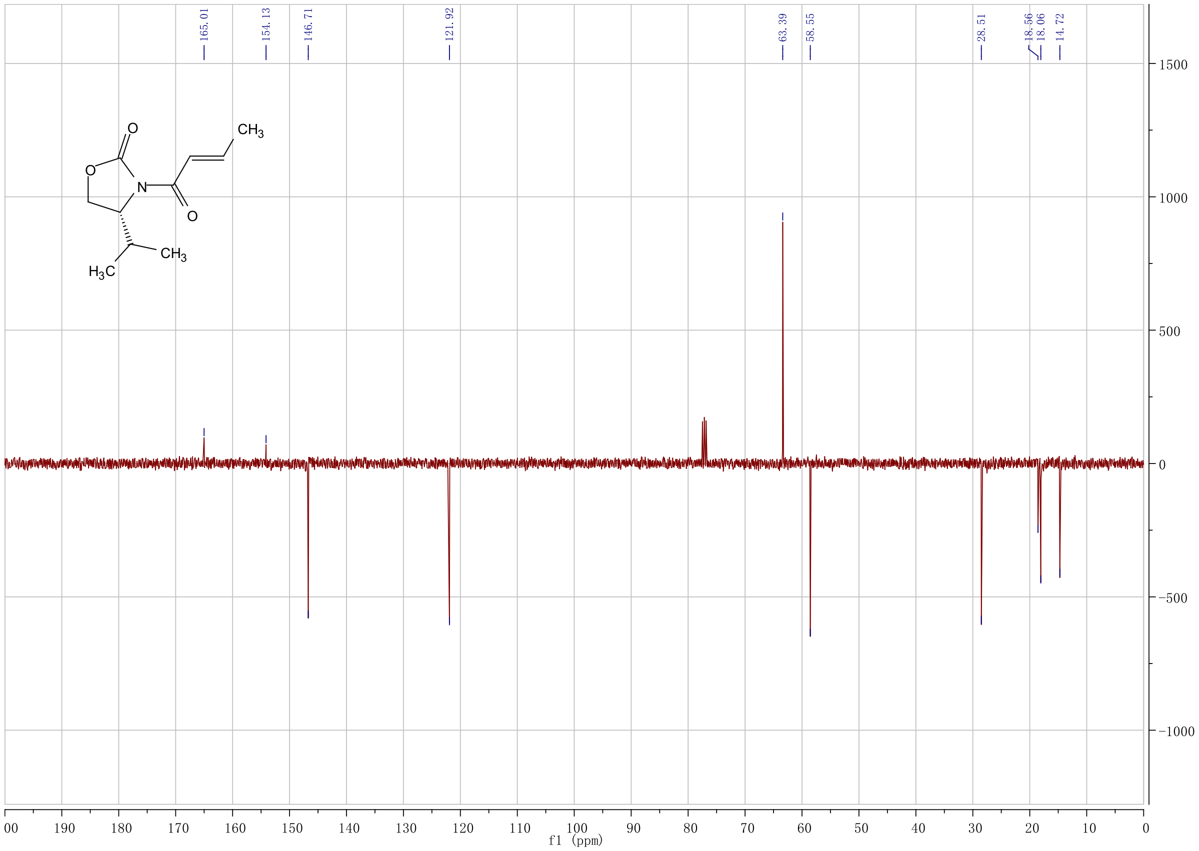

^{1}H and ^{13}C NMR ($CDCl_3$)

(2*R*,3*S*)-2,3-Bis(benzyloxy)pent-4-enal (**15**)

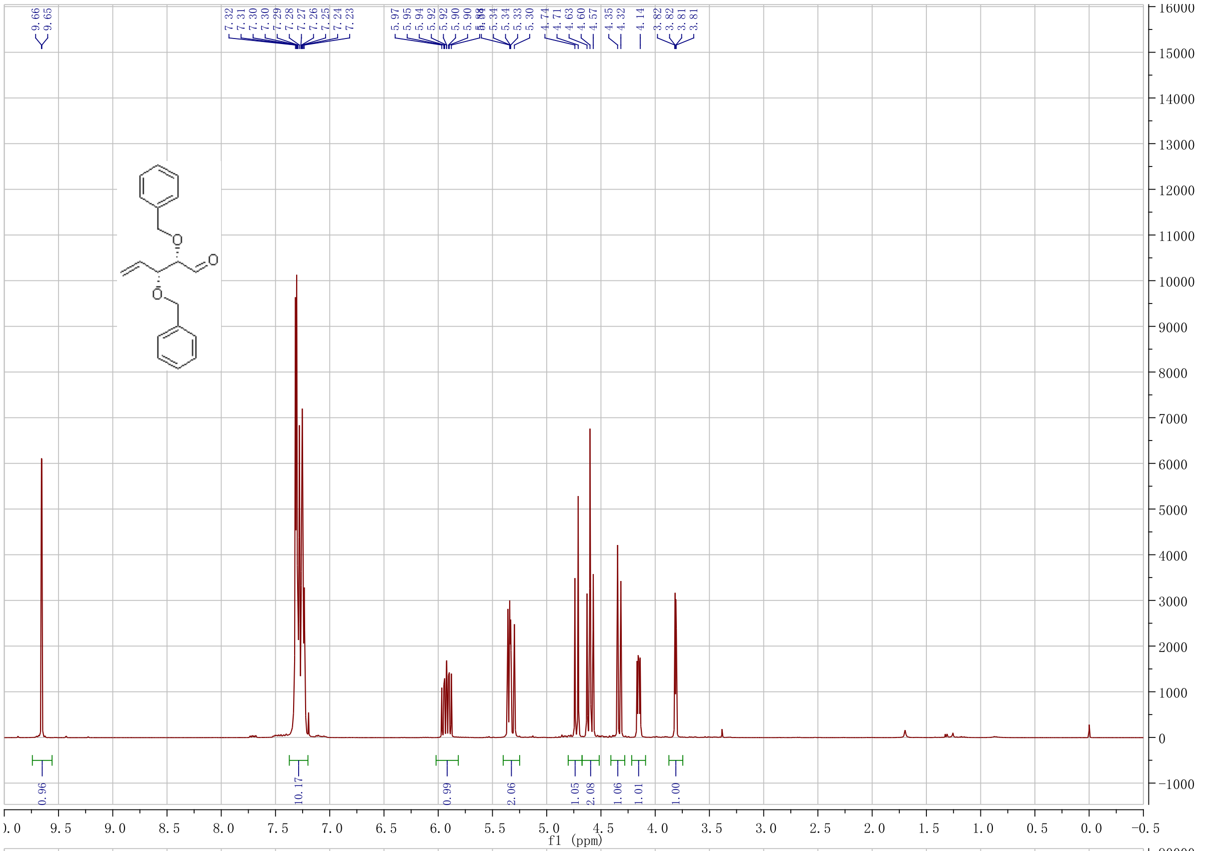

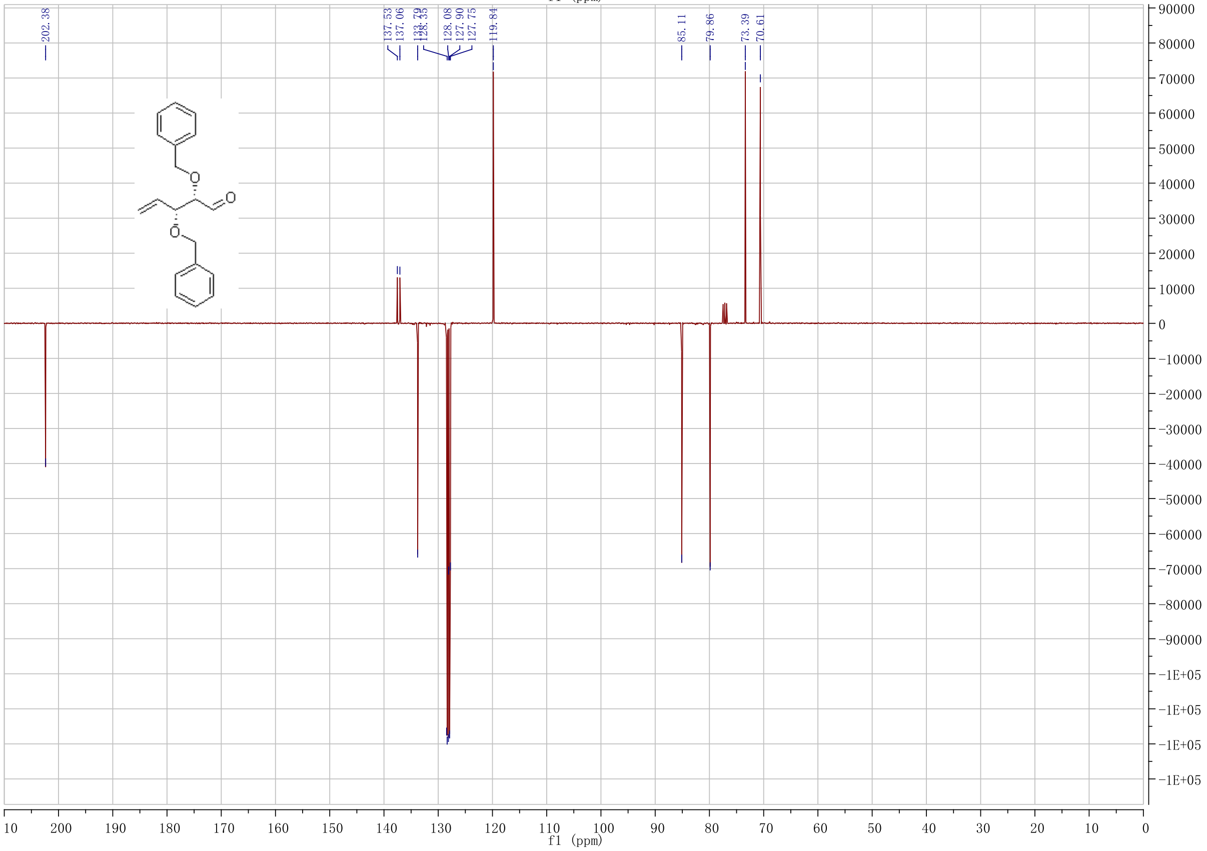

^{1}H and ^{13}C NMR ($CDCl_3$)

isopropyloxazolidin-2-one (**16**):

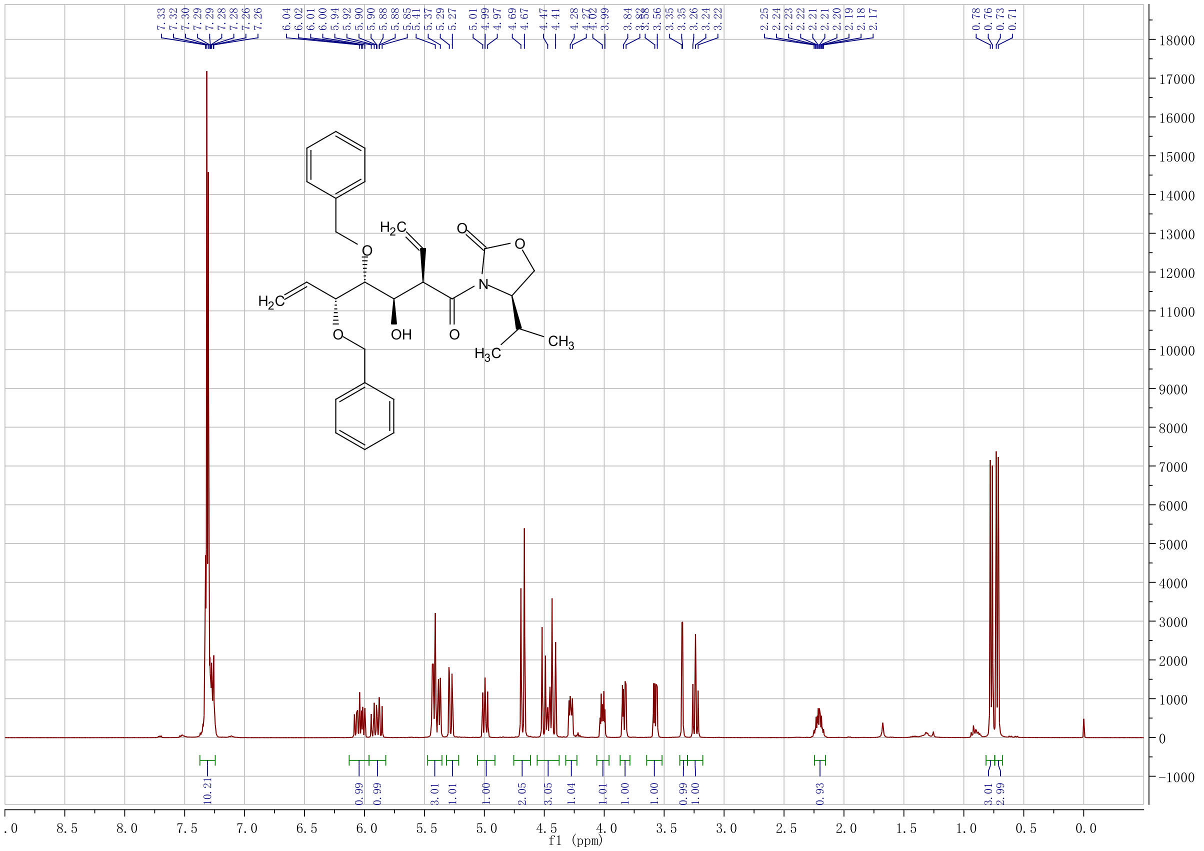

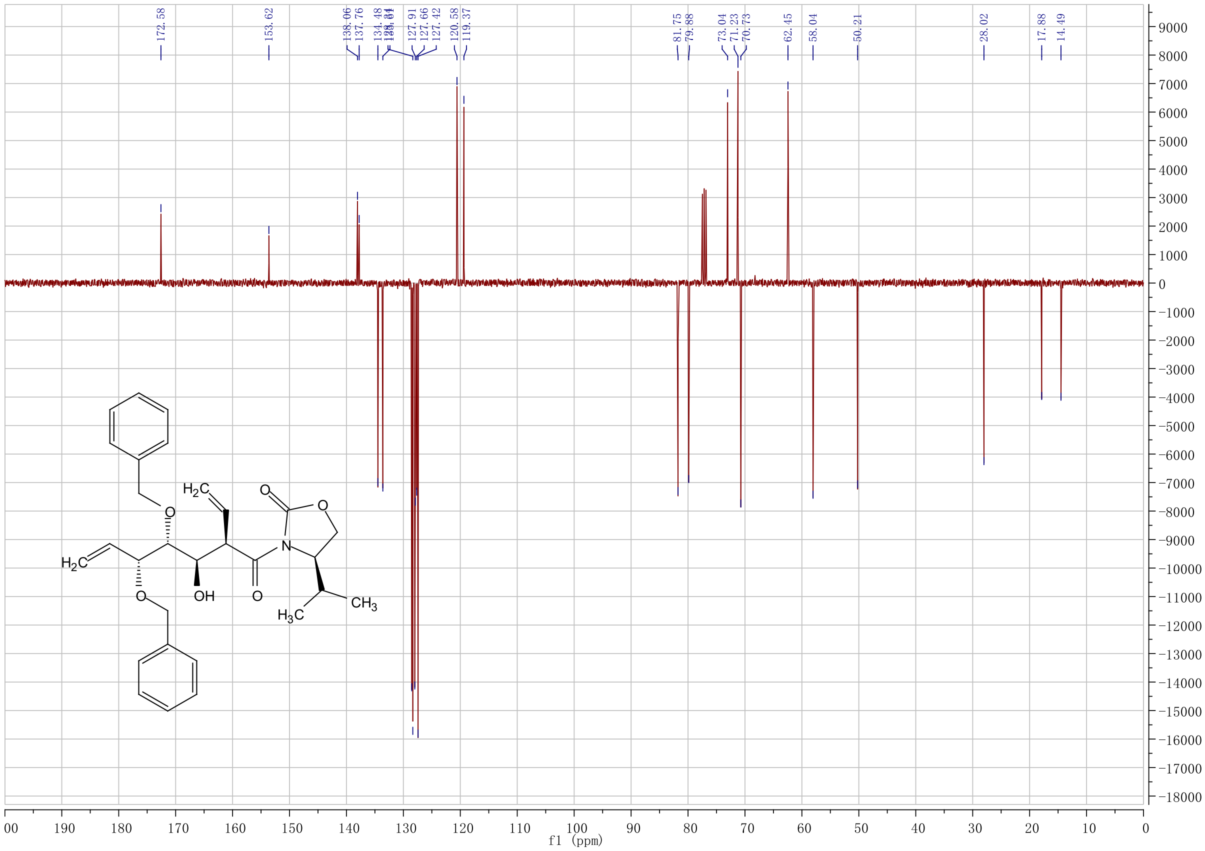

^{1}H and ^{13}C NMR ($CDCl_3$)

(2*S*,3*R*,4*R*,5*R*)-4,5-bis(benzyloxy)-2-vinylhept-6-ene-1,3-diol

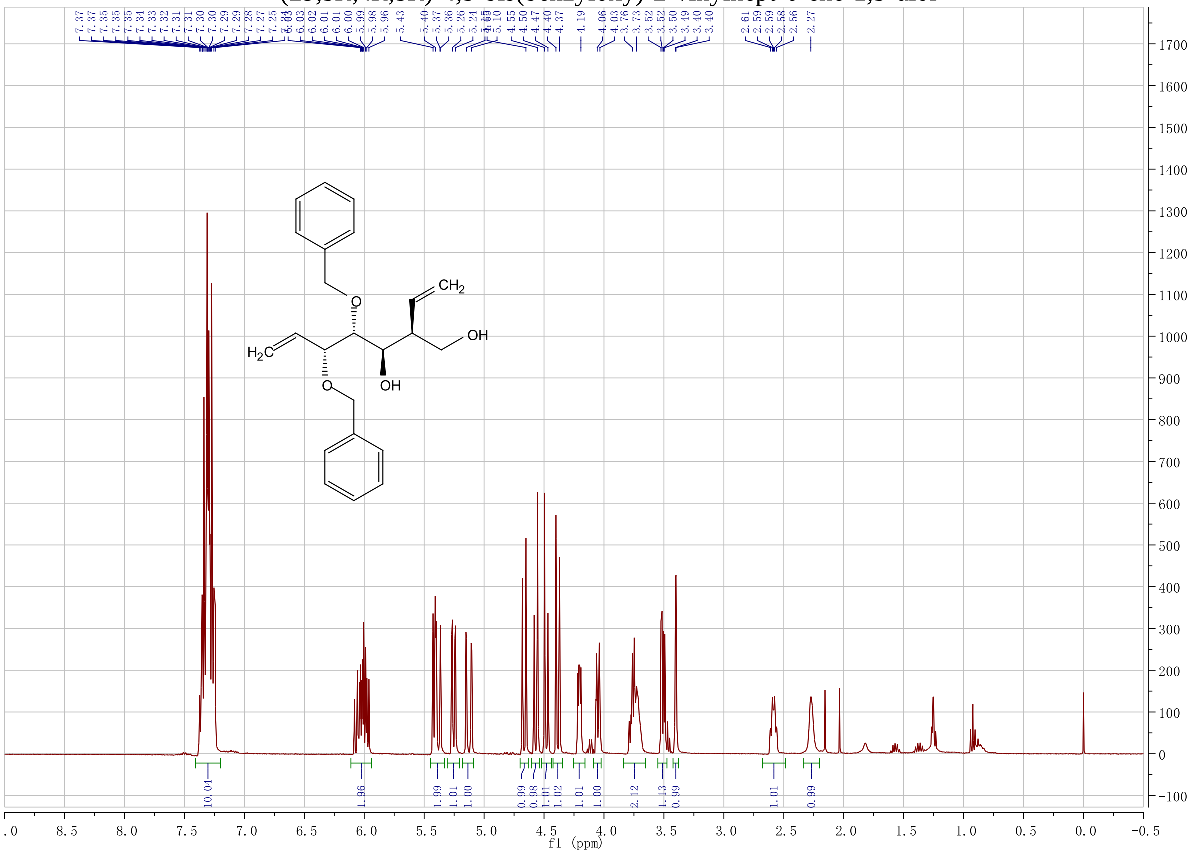

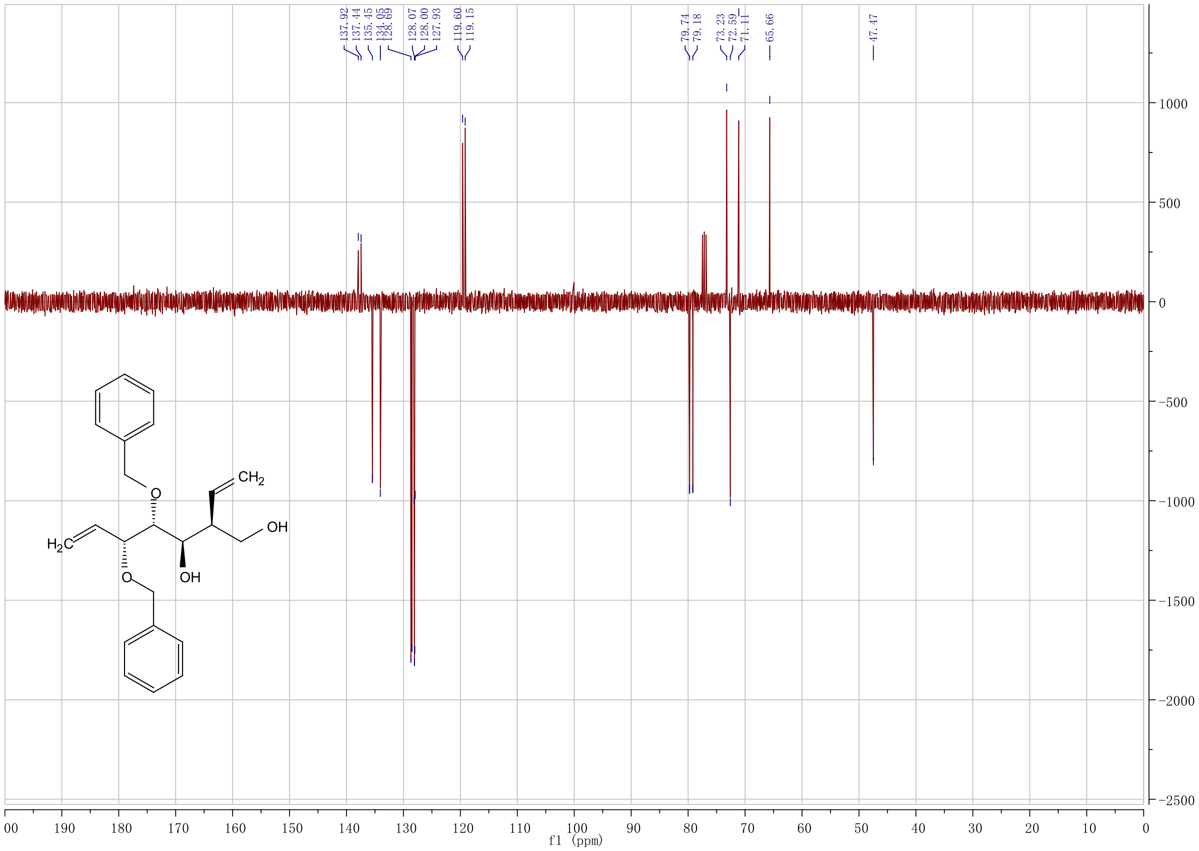

^{1}H and ^{13}C NMR ($CDCl_3$)

(1*R*,2*S*,5*R*,6*R*)-5,6-Bis(benzyloxy)-2-(hydroxymethyl)cyclohex-3-en-1-ol (**17**)

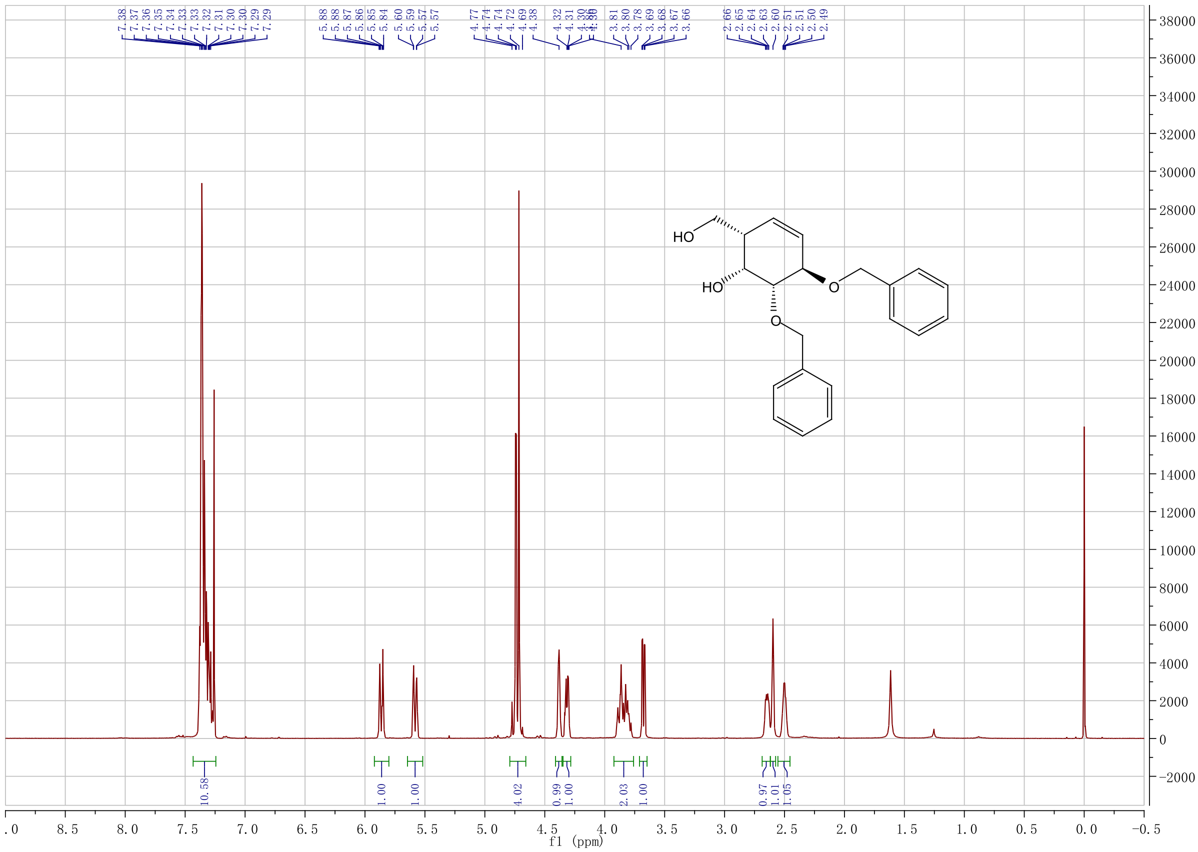

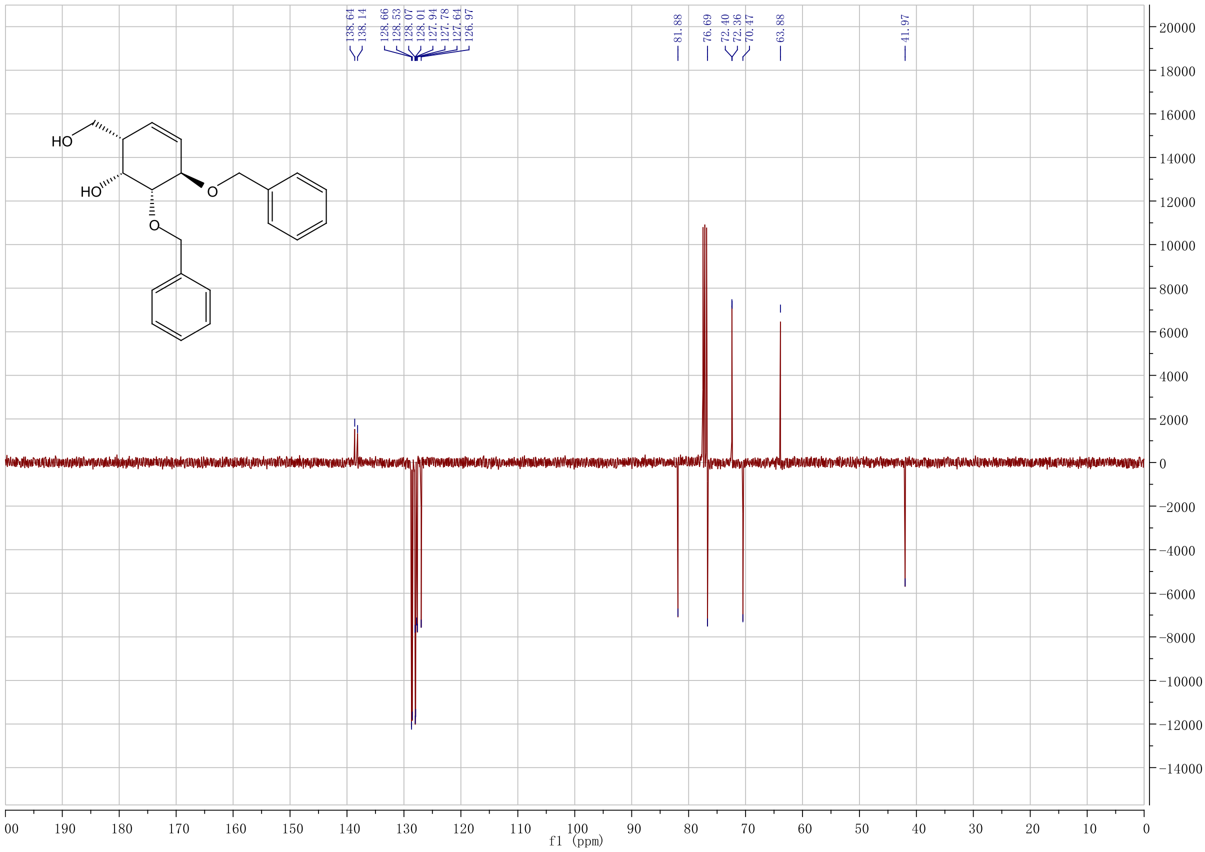

^{1}H and ^{13}C NMR ($CDCl_3$)

((1*S*,4*R*,5*R*,6*R*)-4,5-Bis(benzyloxy)-6-hydroxycyclohex-2-en-1-yl)methyl 4-methylbenzenesulfonate **(18)**

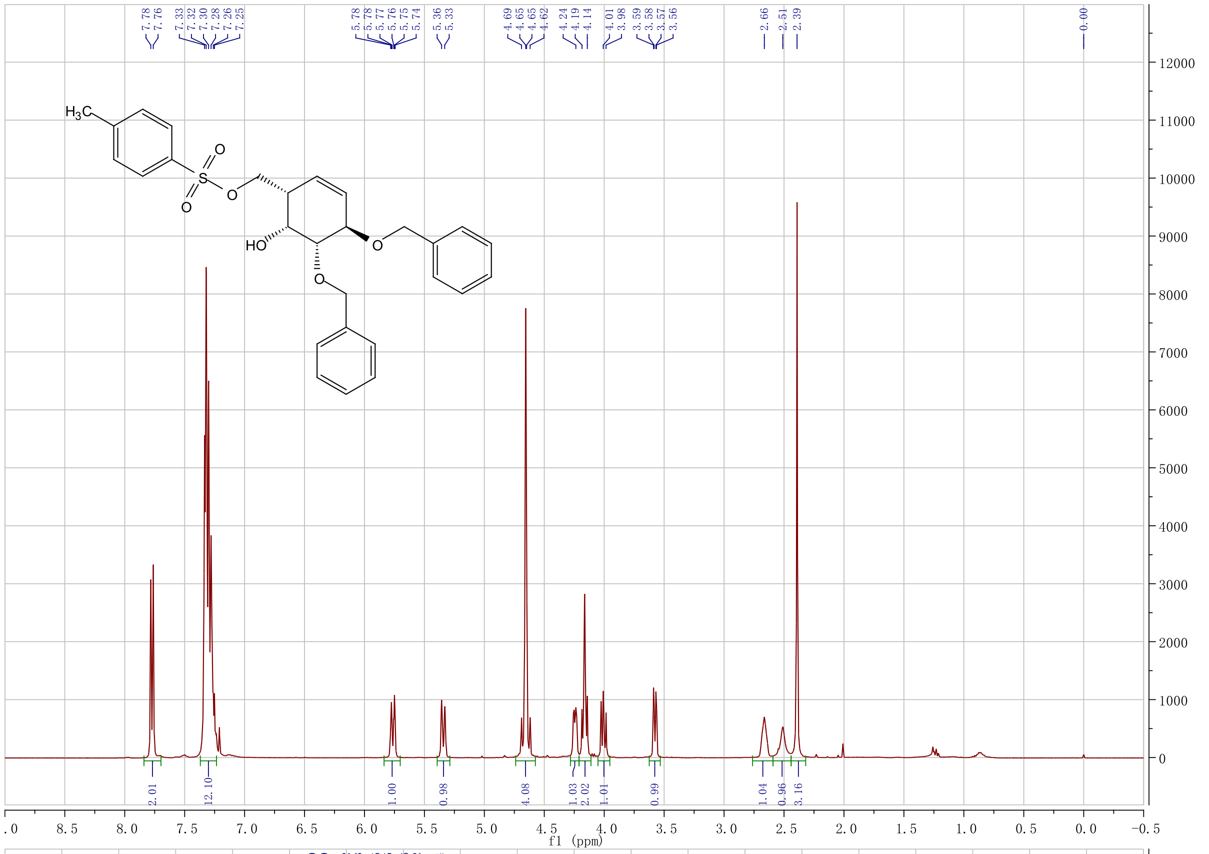

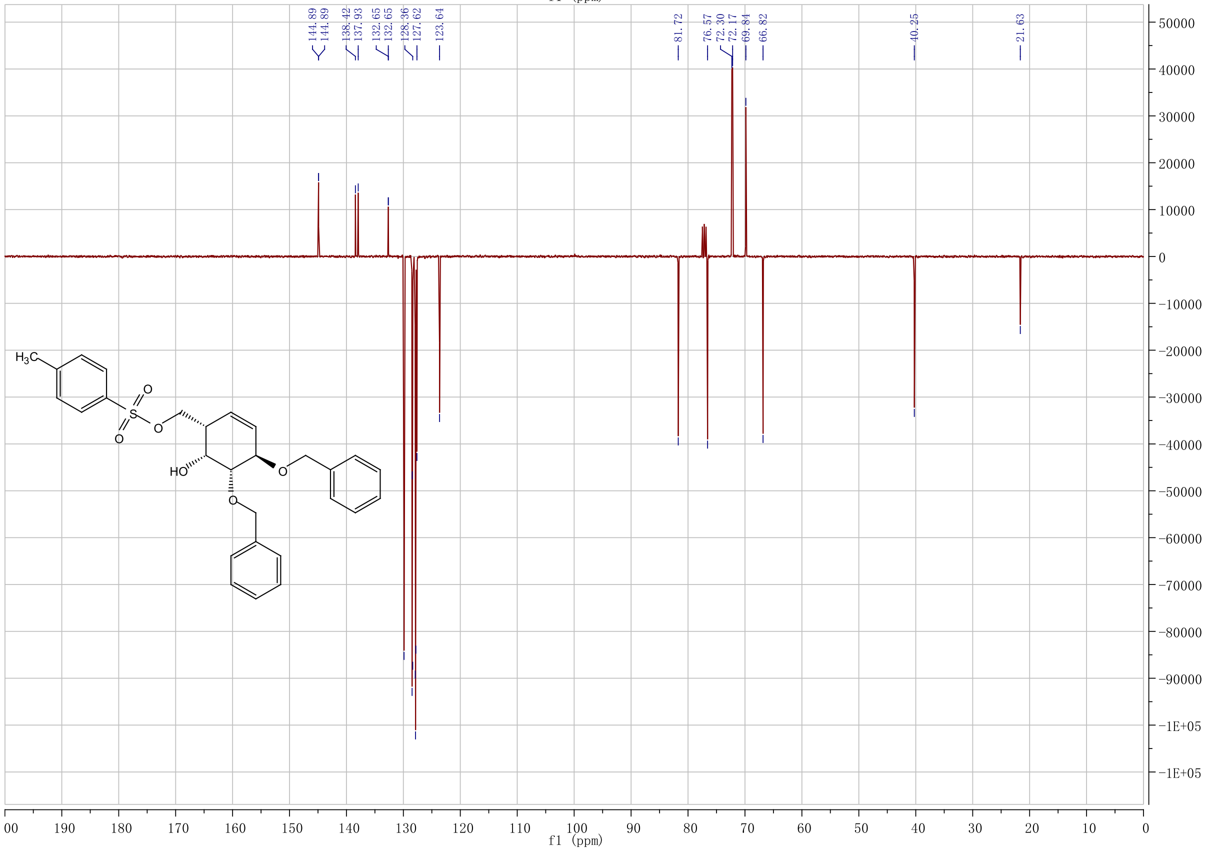

^{1}H and ^{13}C NMR ($CDCl_3$)

(1*R*,2*R*,5*R*,6*R*)-5,6-Bis(benzyloxy)-2-methylcyclohex-3-en-1-ol **(19):**

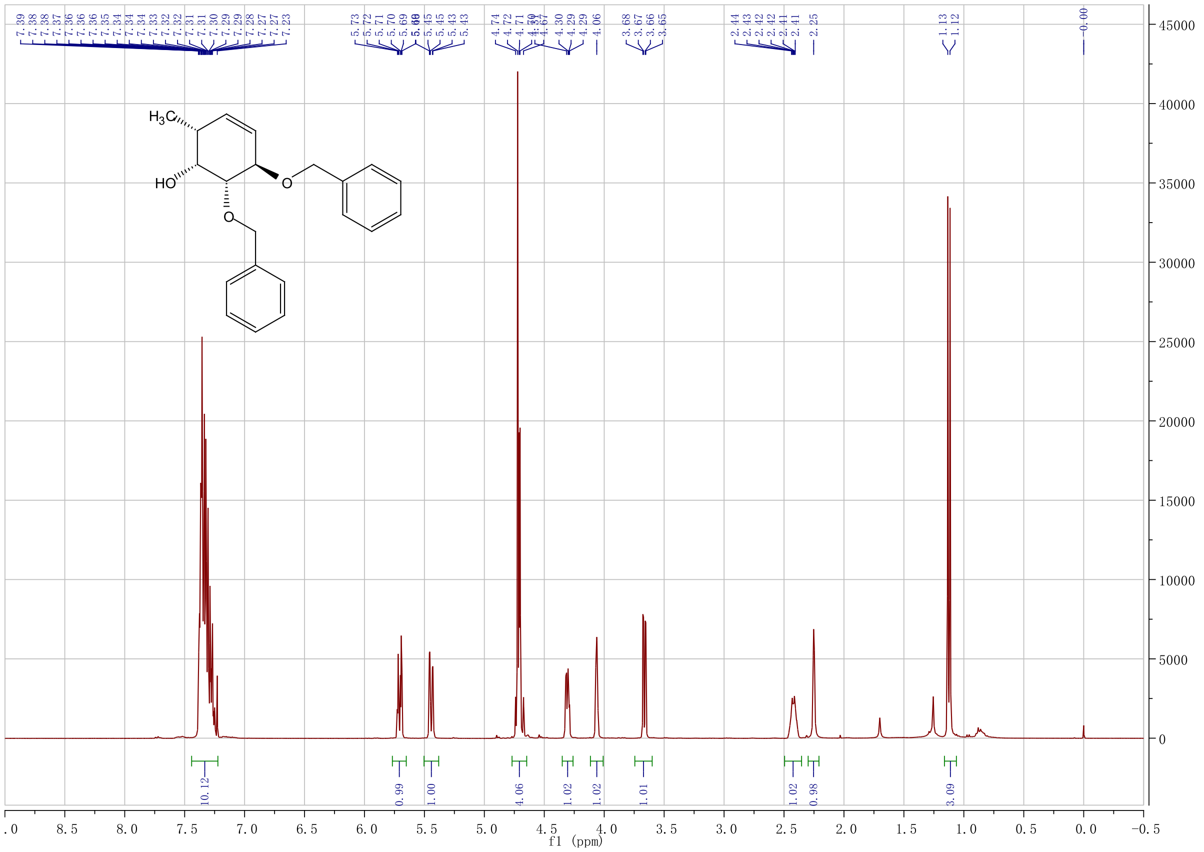

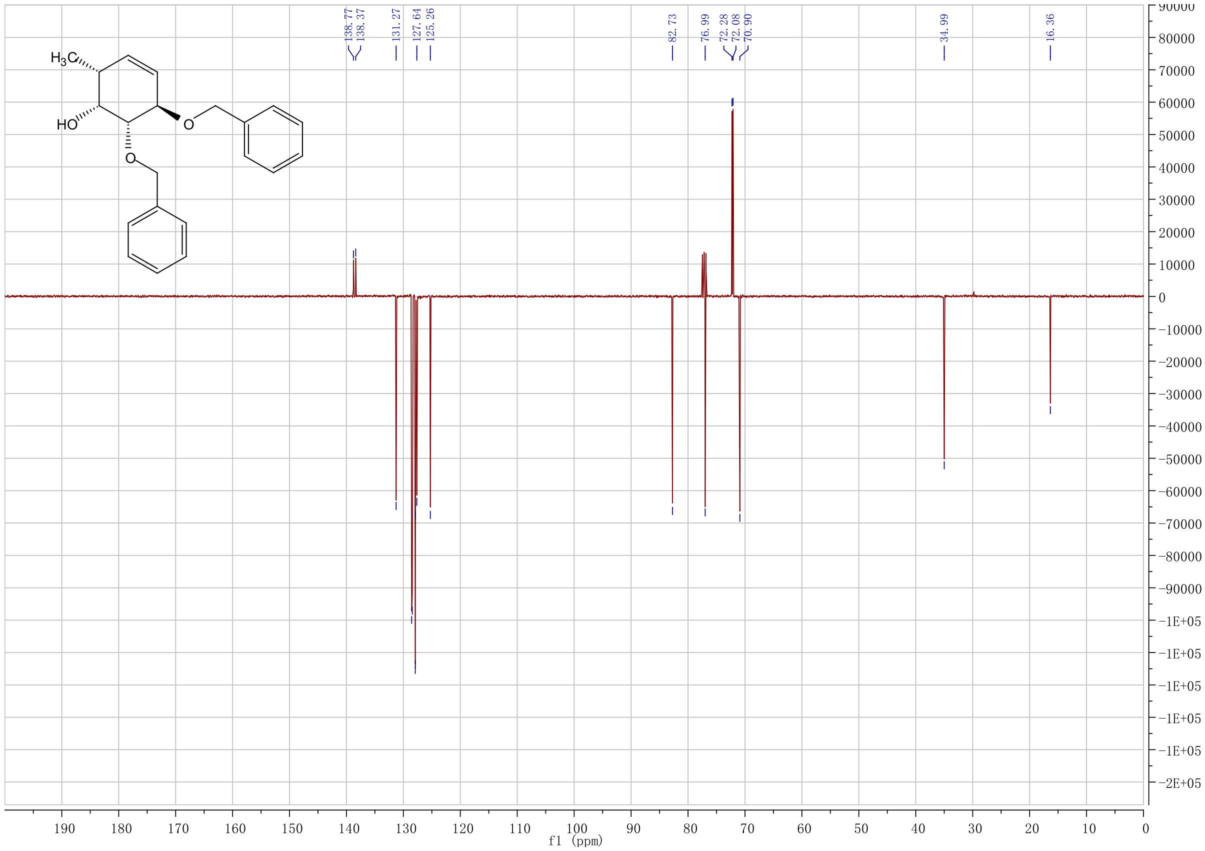

^{1}H and ^{13}C NMR (CD_3OD)

(1*R*,2*S*,3*R*,6*S*)-6-(hydroxymethyl)cyclohex-4-ene-1,2,3-triol

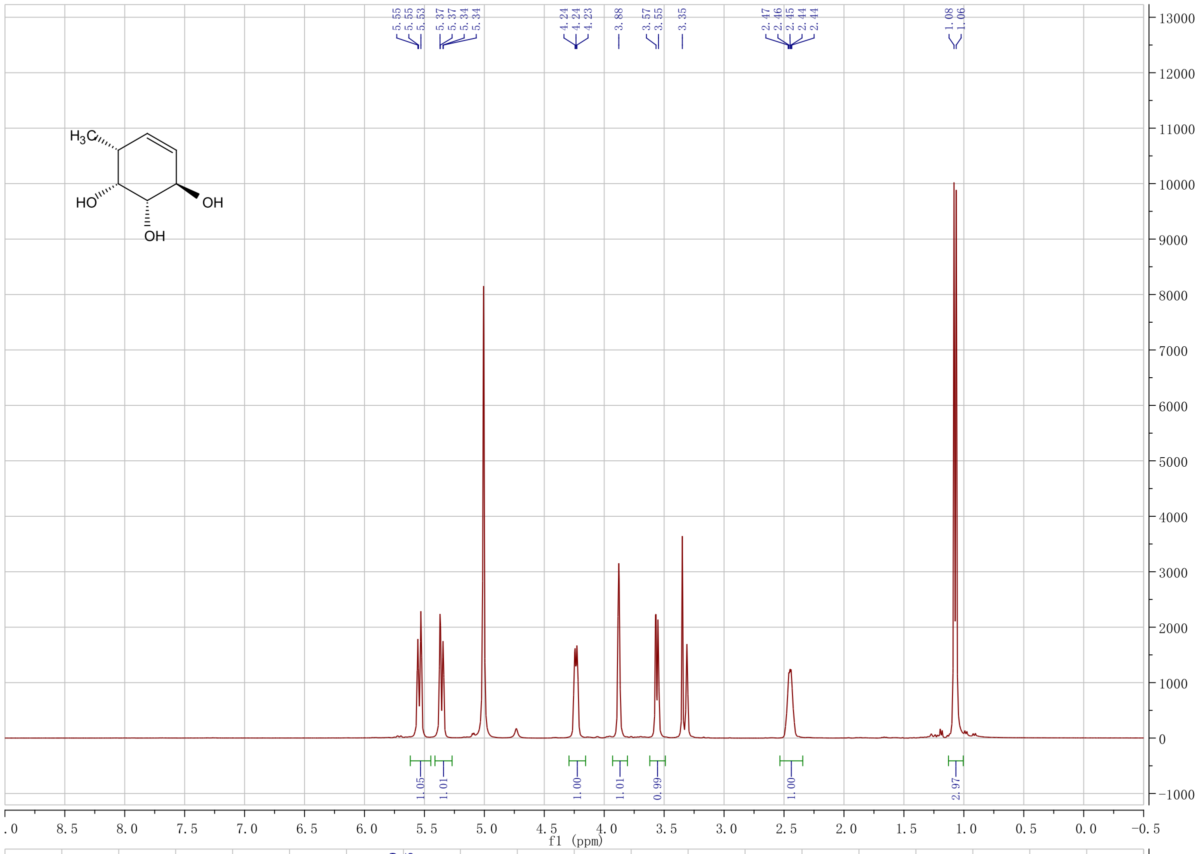

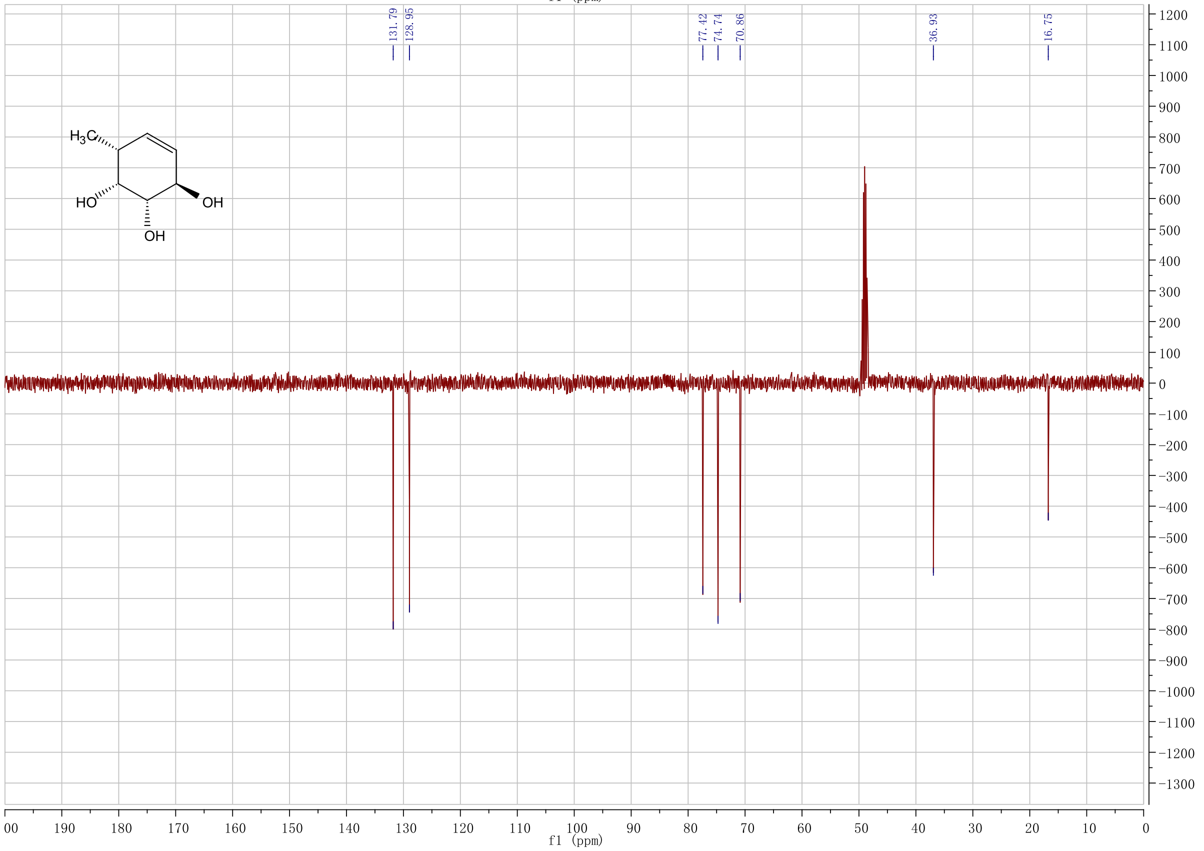

^{1}H and ^{13}C NMR ($CDCl_3$)

(3a*S*,4*R*,7*R*,7a*R*)-2,2,7-trimethyl-3a,4,7,7a-tetrahydrobenzo[d][1,3]dioxol-4-ol **(20)**

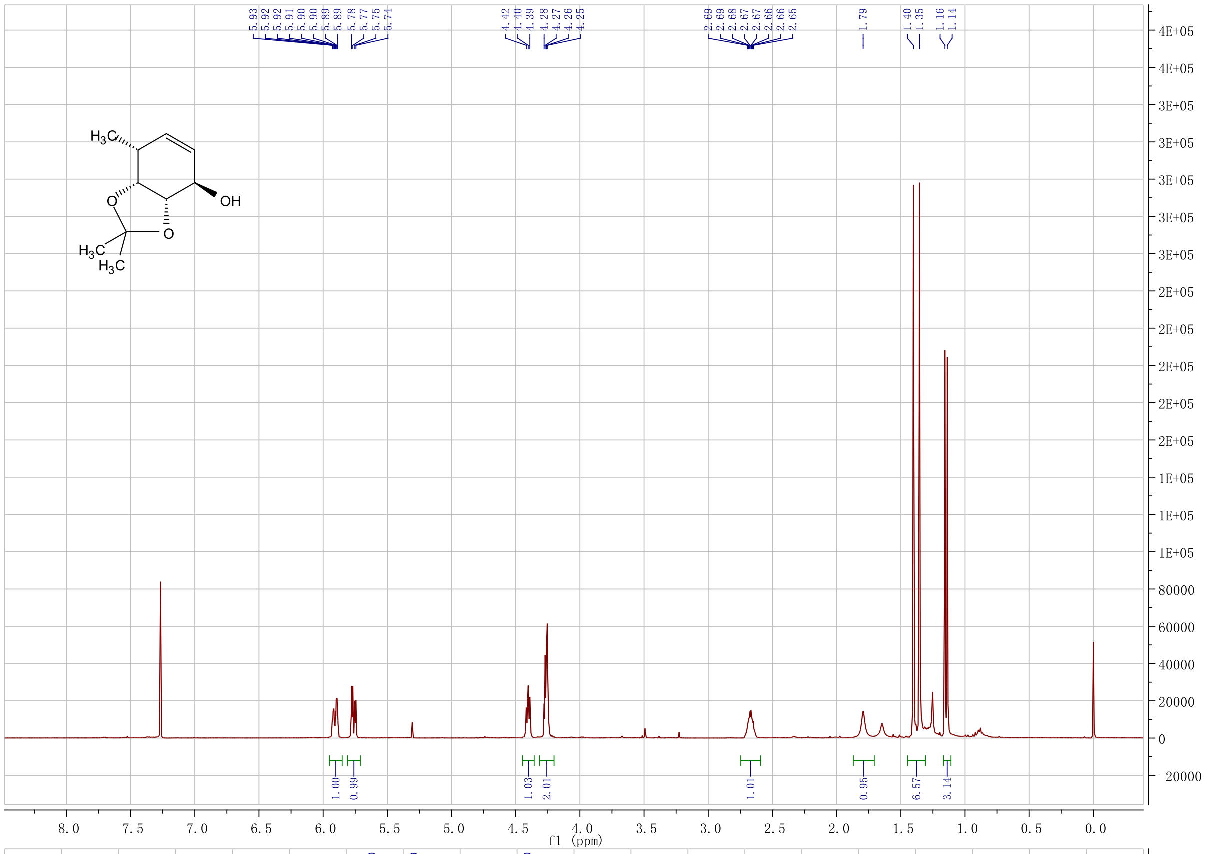

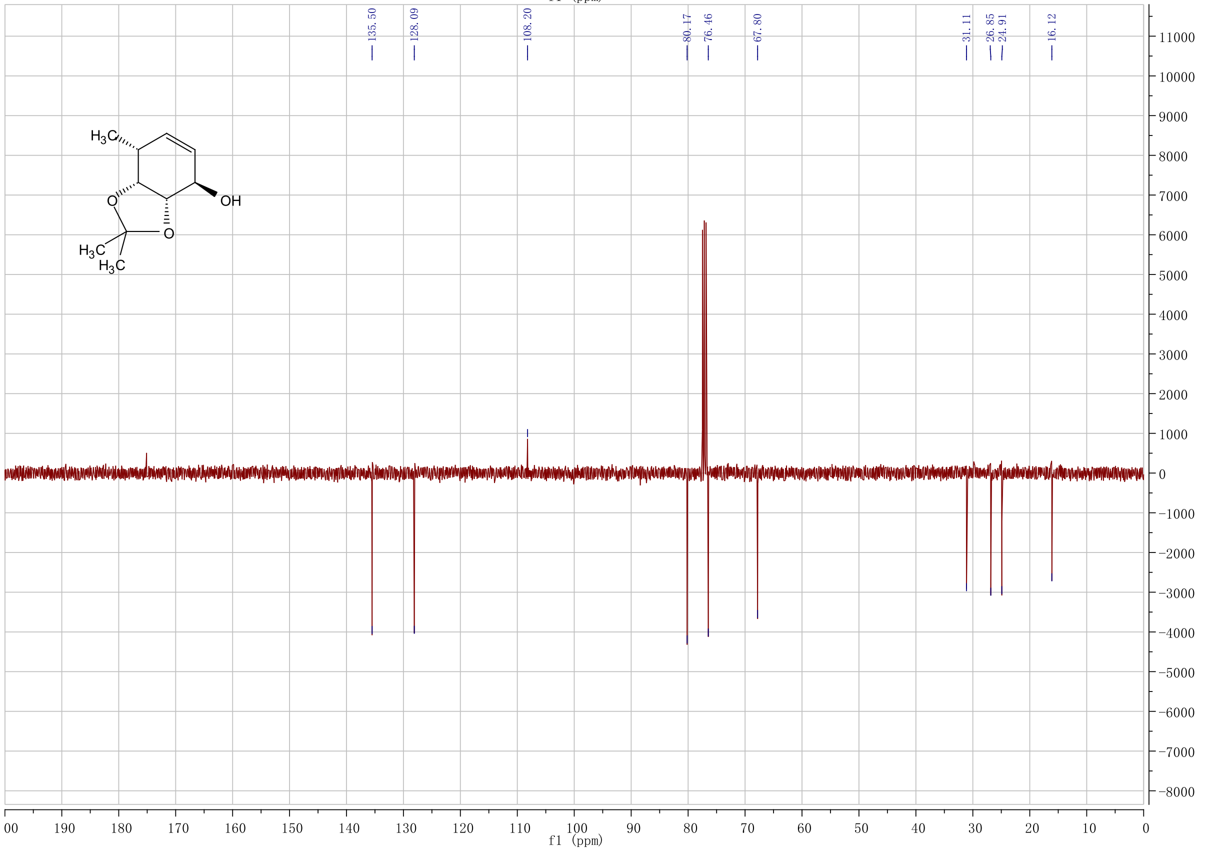

^1H and ^{13}C NMR ($CDCl_3$)

Iodide **21**

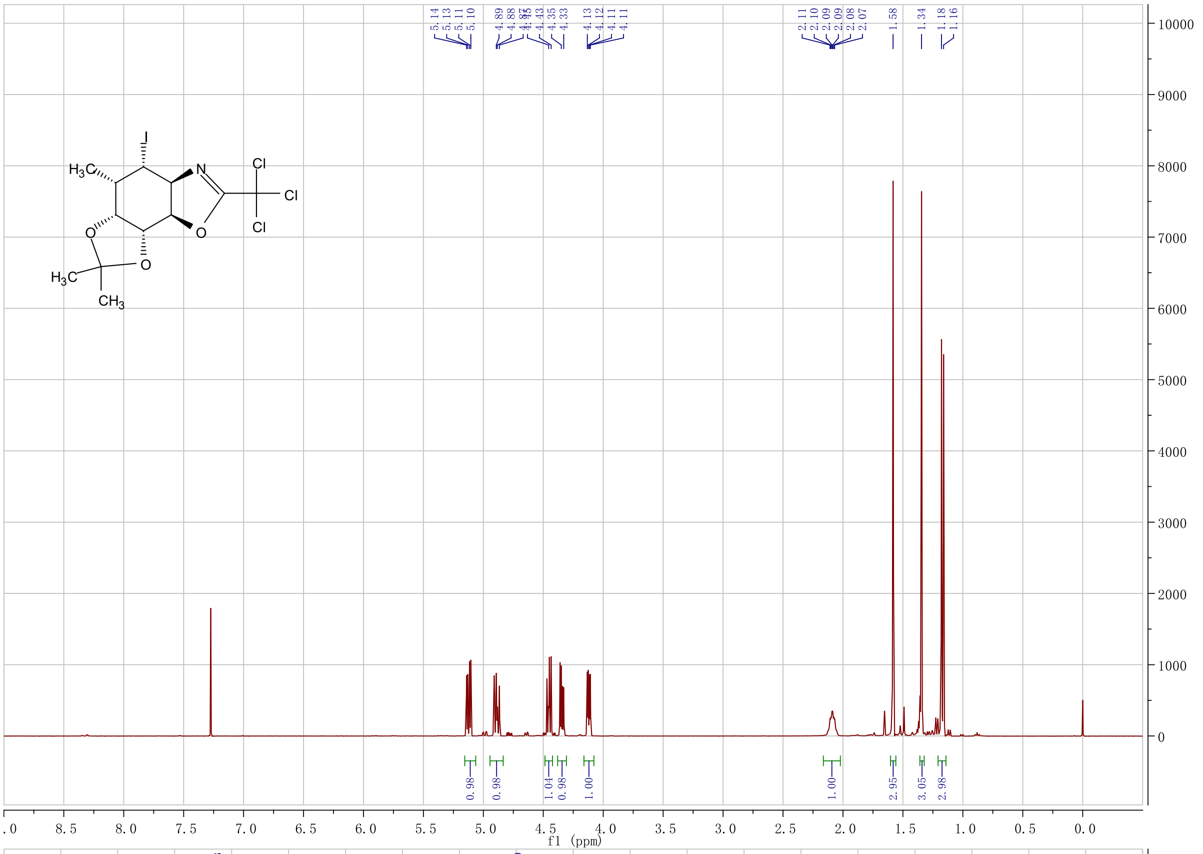

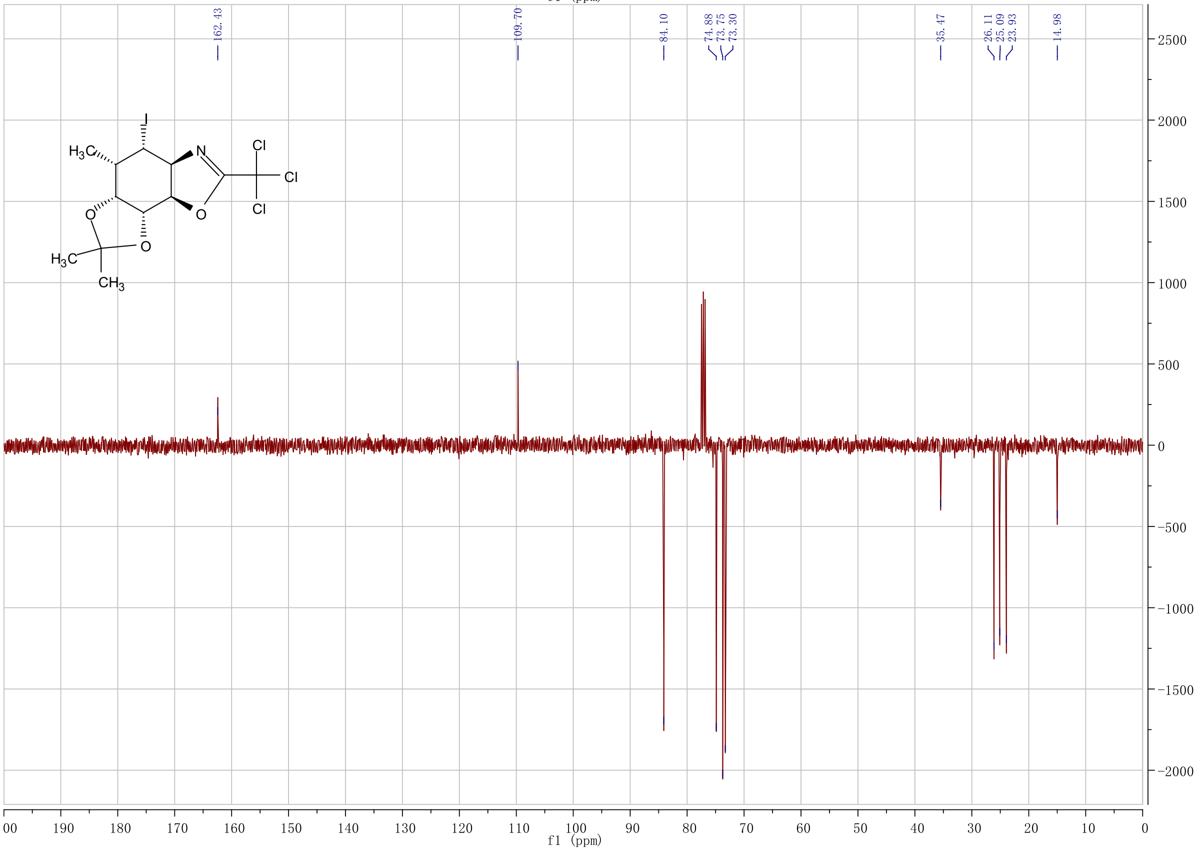

^{1}H and ^{13}C NMR (CD_3OD)

(1*R*,2*R*,3*R*,4*R*,5*R*,6*R*)-5-methyl-7-azabicyclo[4.1.0]heptane-2,3,4-triol (**22**)

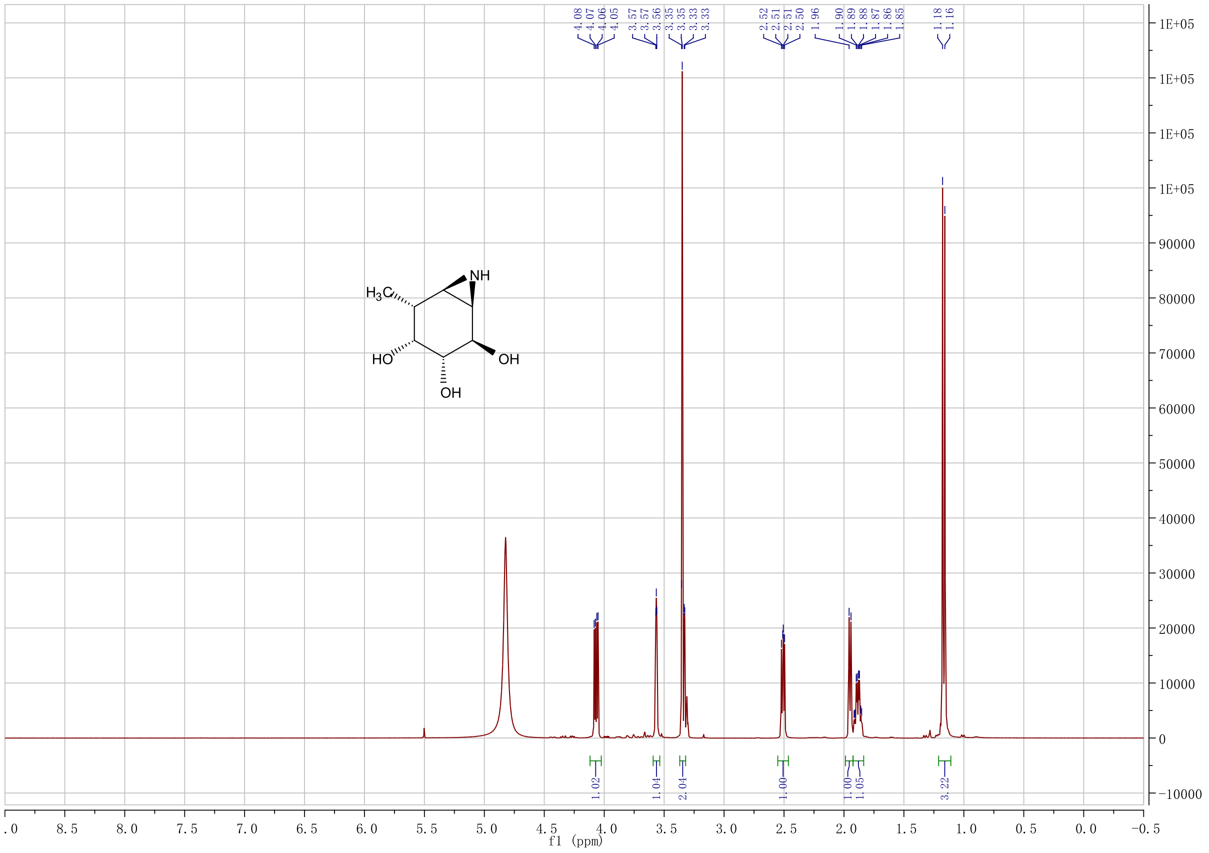

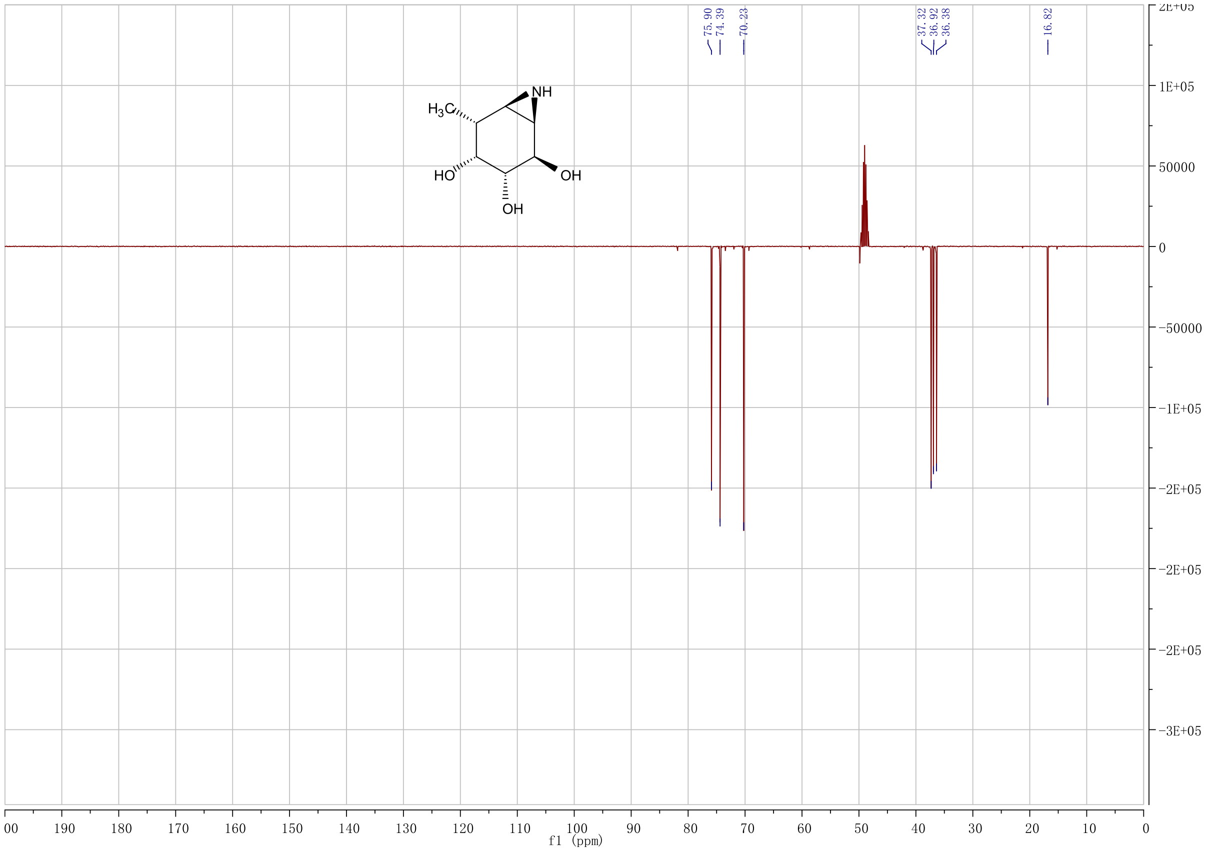

^{1}H and ^{13}C NMR (CD_3OD)

8-Azido-1-((1*R*,2*R*,3*R*,4*R*,5*R*,6*R*)-2,3,4-trihydroxy-5-methyl-7-azabicyclo[4.1.0]heptan-7-yl)-octan-1-one **(23)**

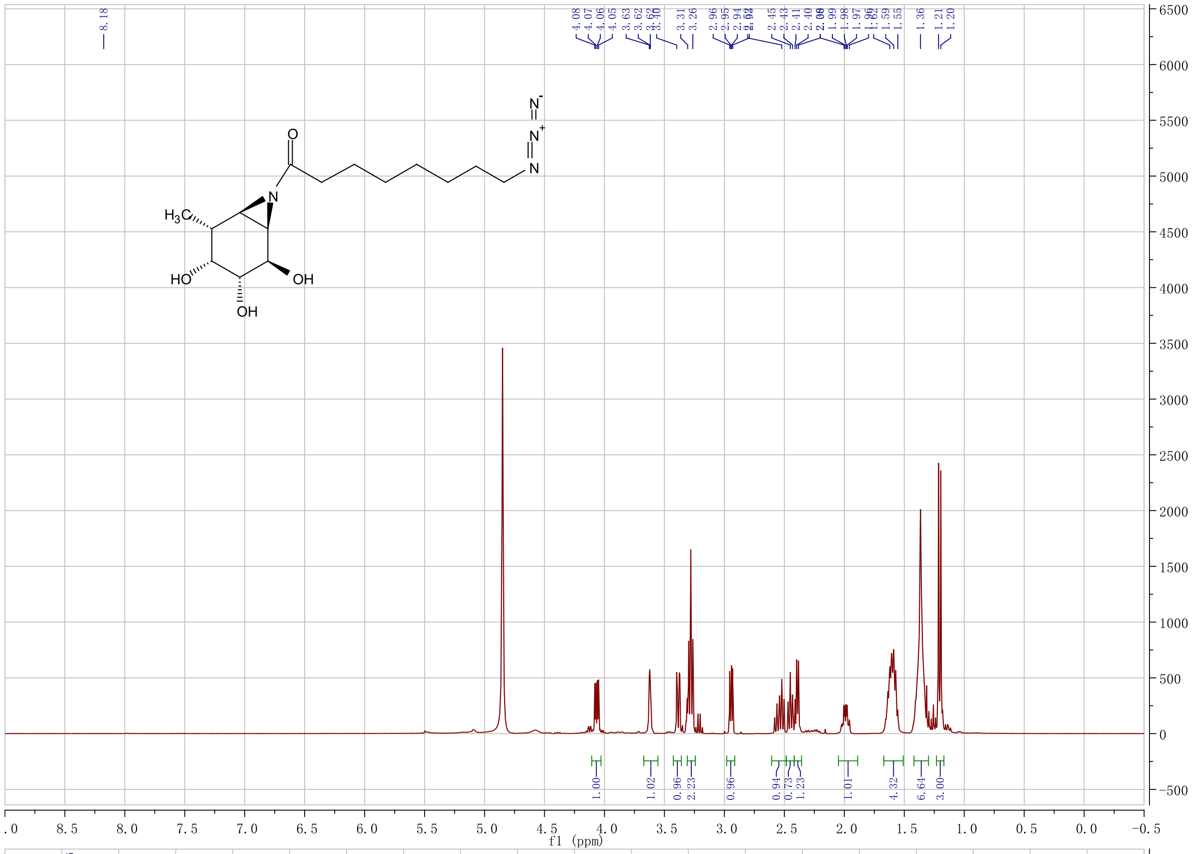

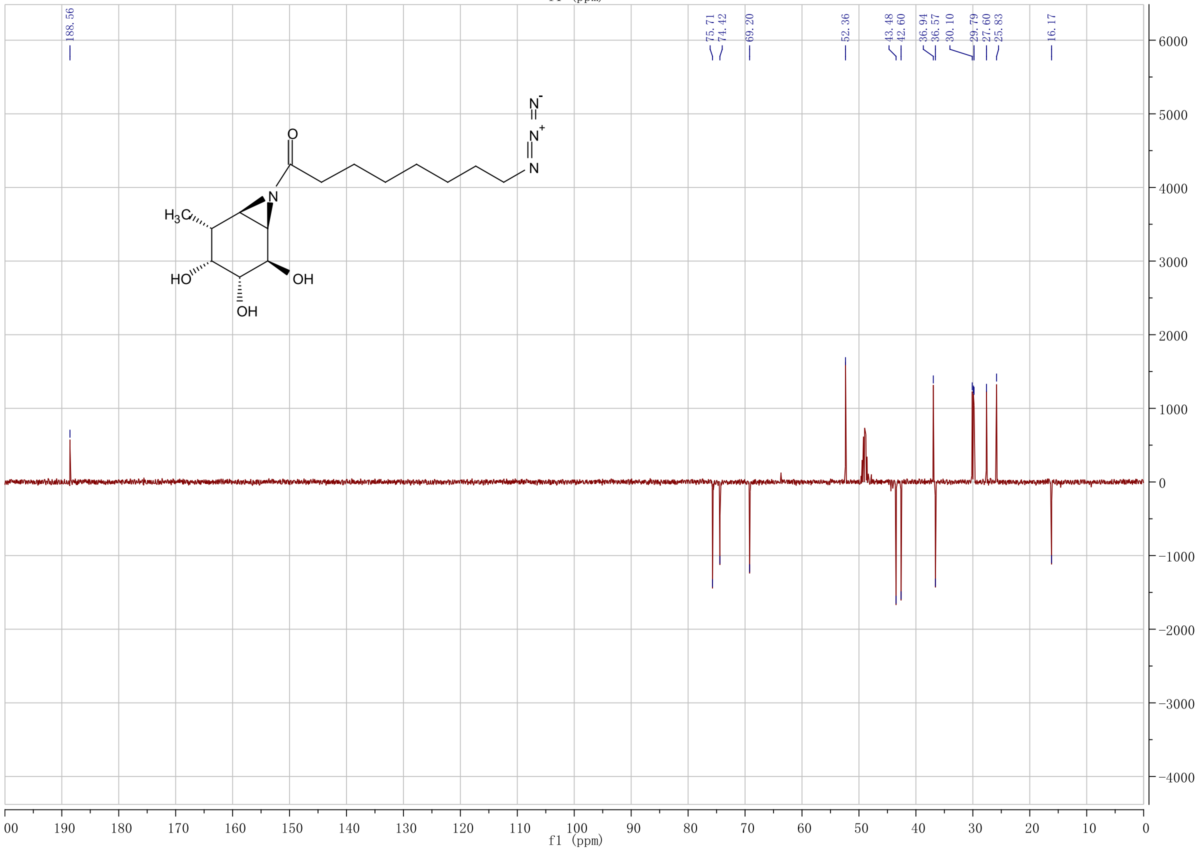

^{1}H and ^{13}C NMR (CD_3OD)

Bodipy compound **1**

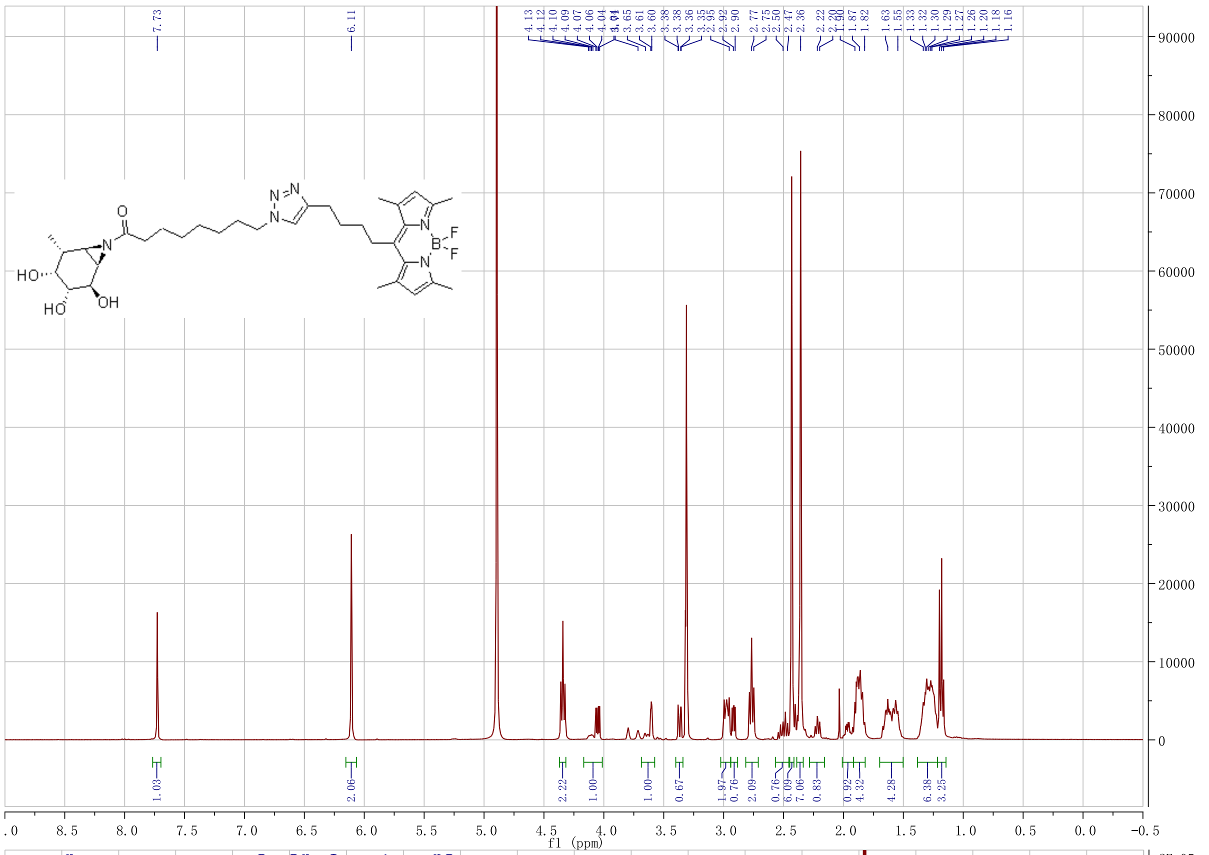

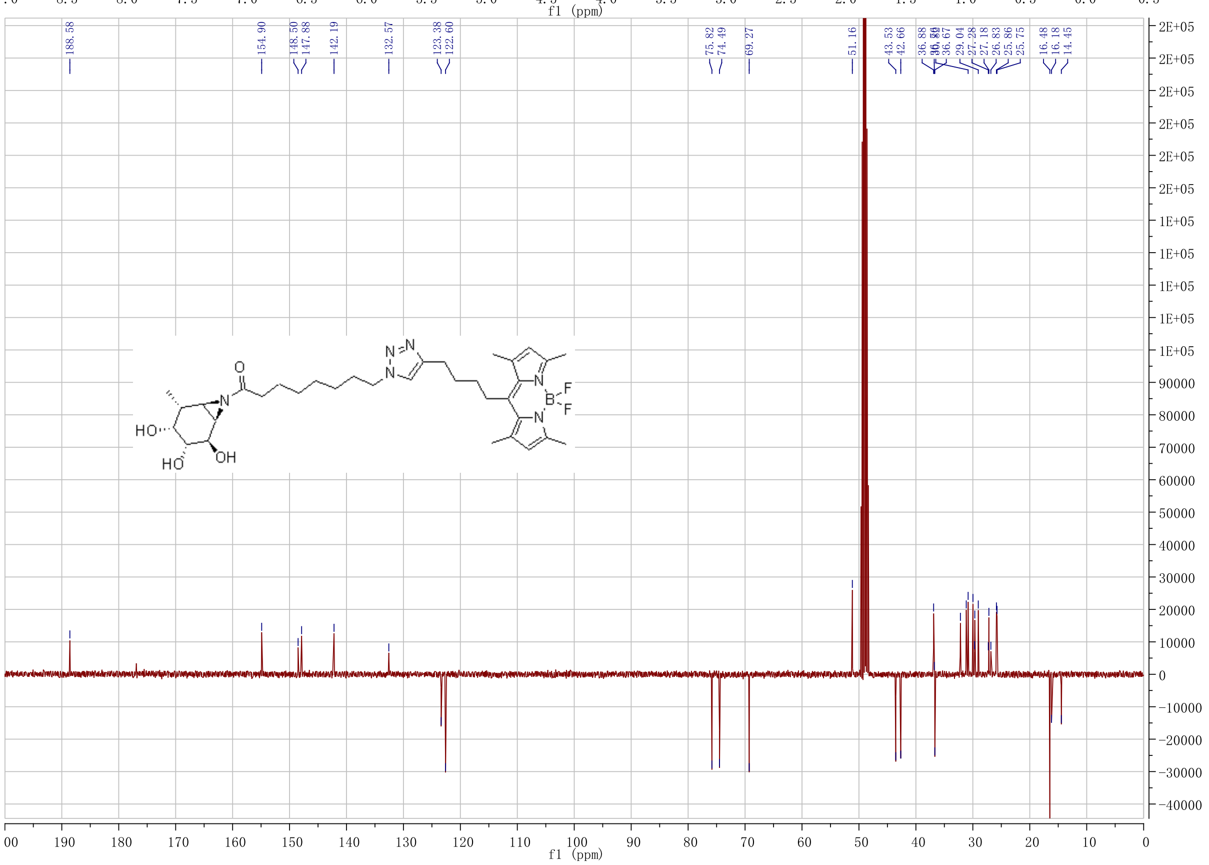

^{1}H and ^{13}C NMR (CD_3CN) Bodipy compound **2**

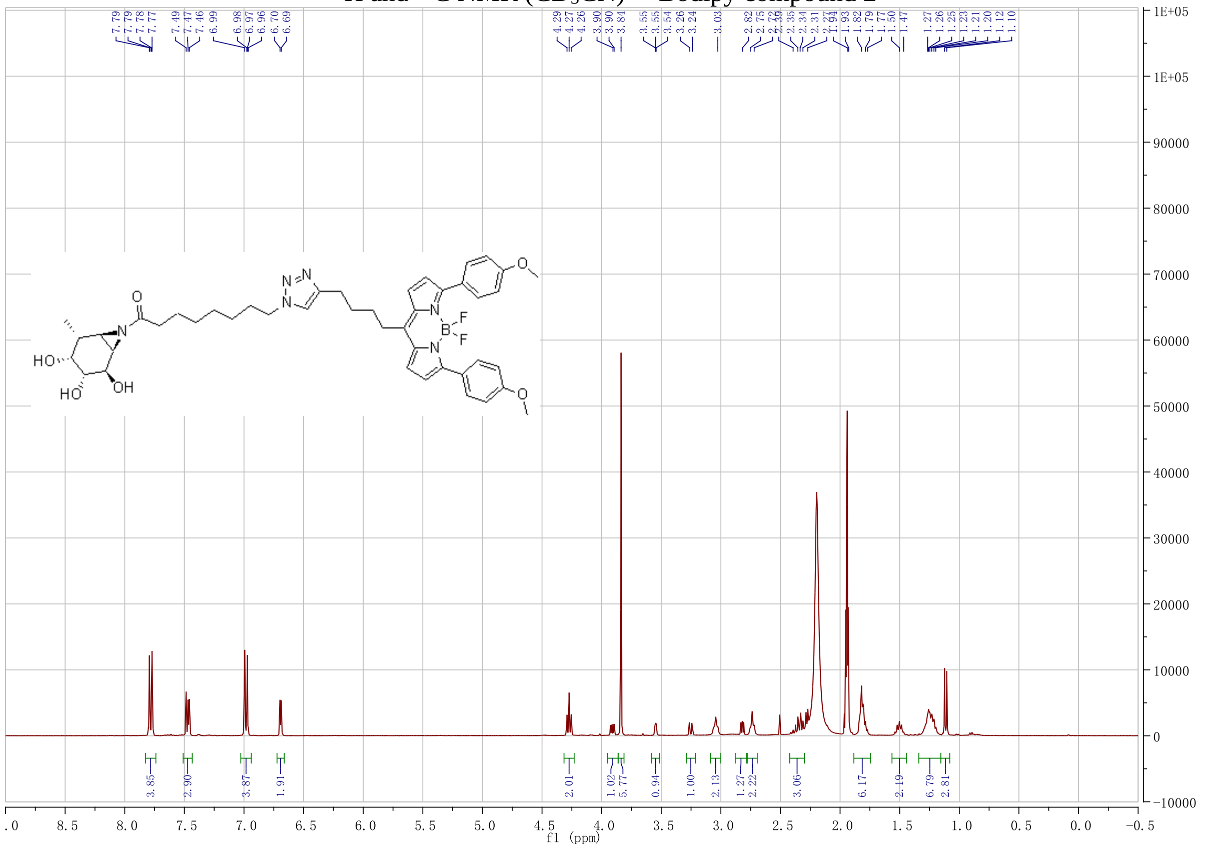

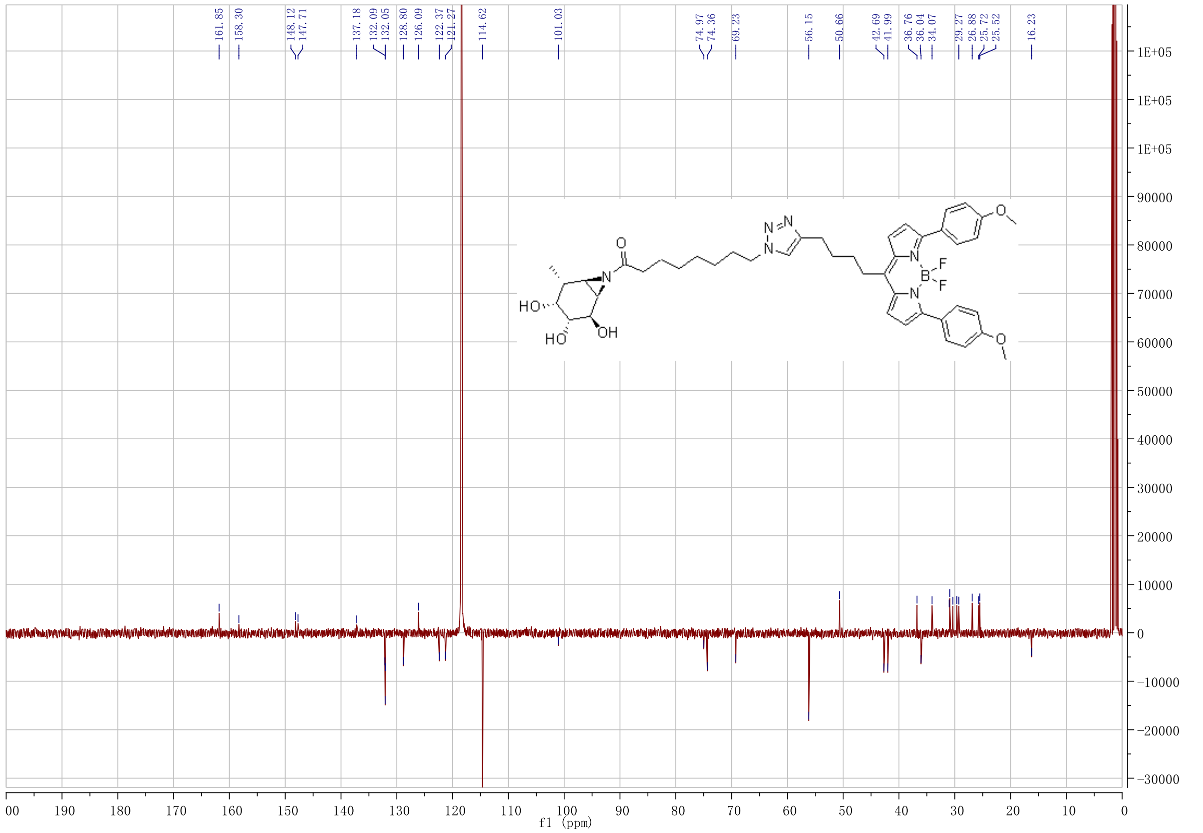

^{1}H and ^{13}C NMR (CD_3OD) Biotin compound **3**

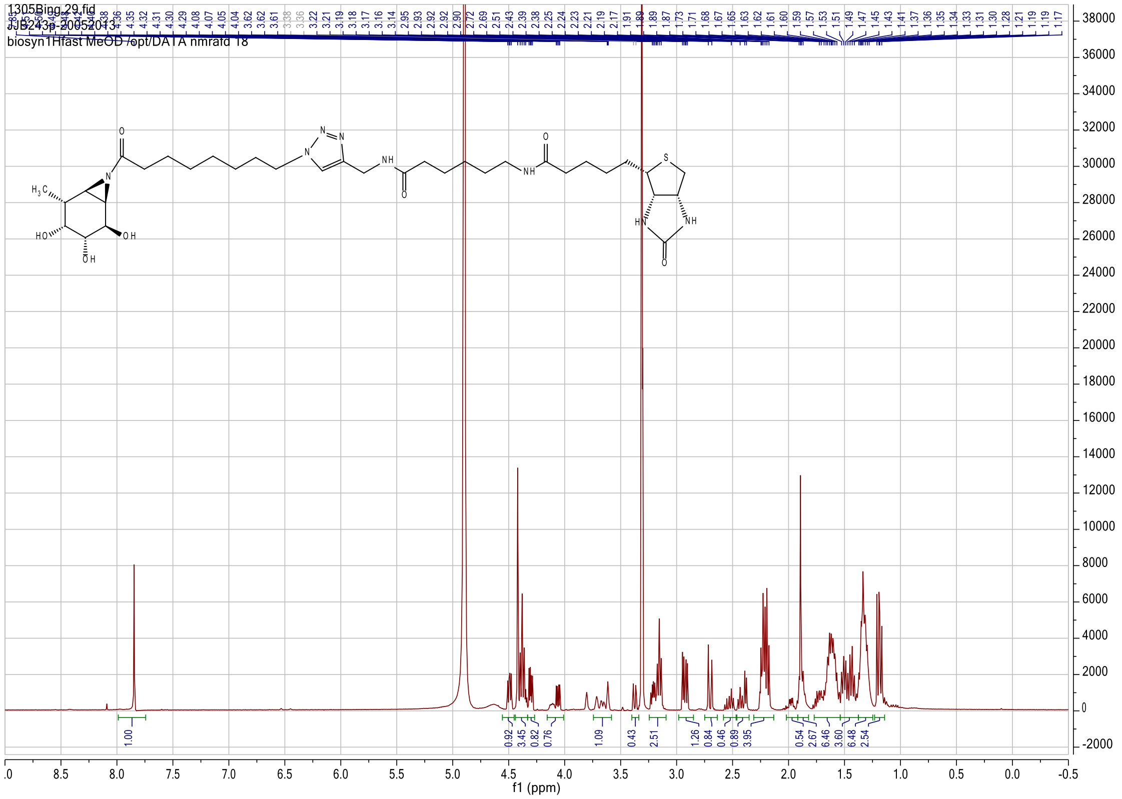

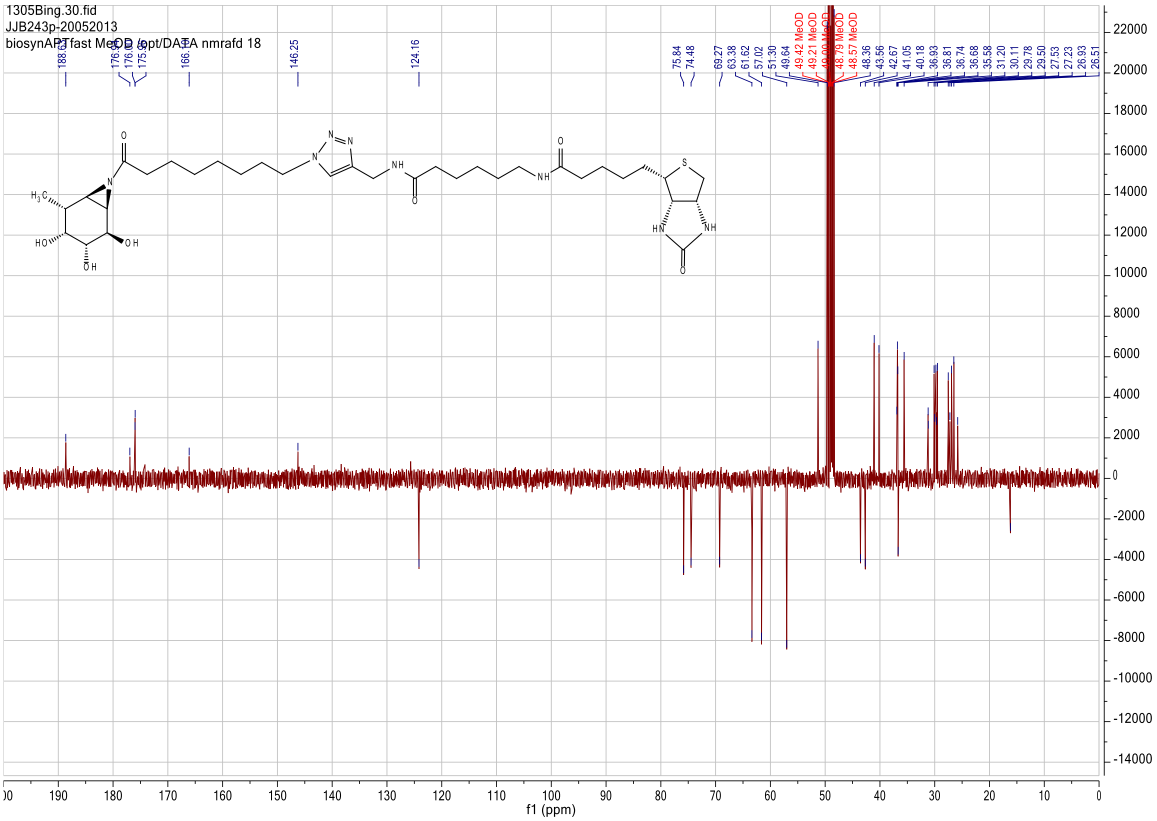

^{1}H and ^{13}C NMR (CD_3OD)

Phenyl((1*R*,2*R*,3*R*,4*R*,5*R*,6*R*)-2,3,4-trihydroxy-5-methyl-7-azabicyclo[4.1.0]heptan-7-yl)methanone **(4)**

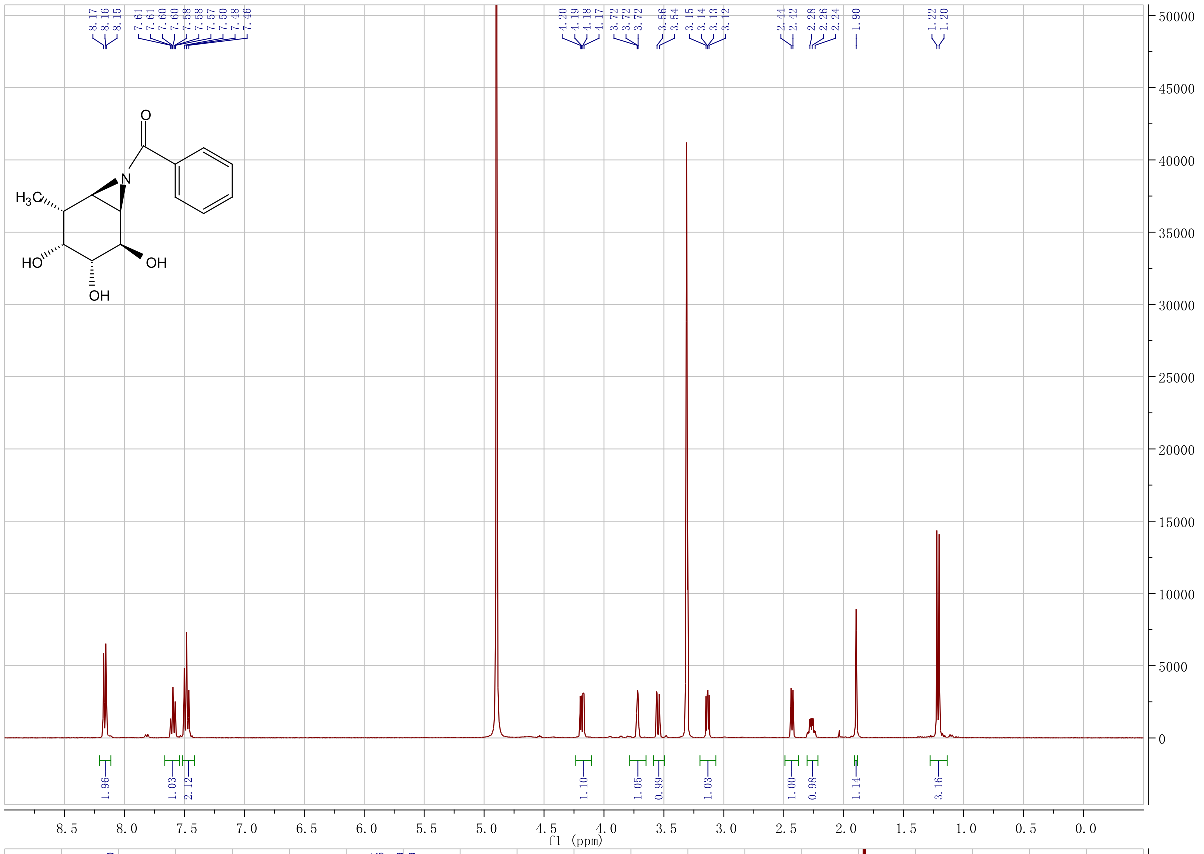

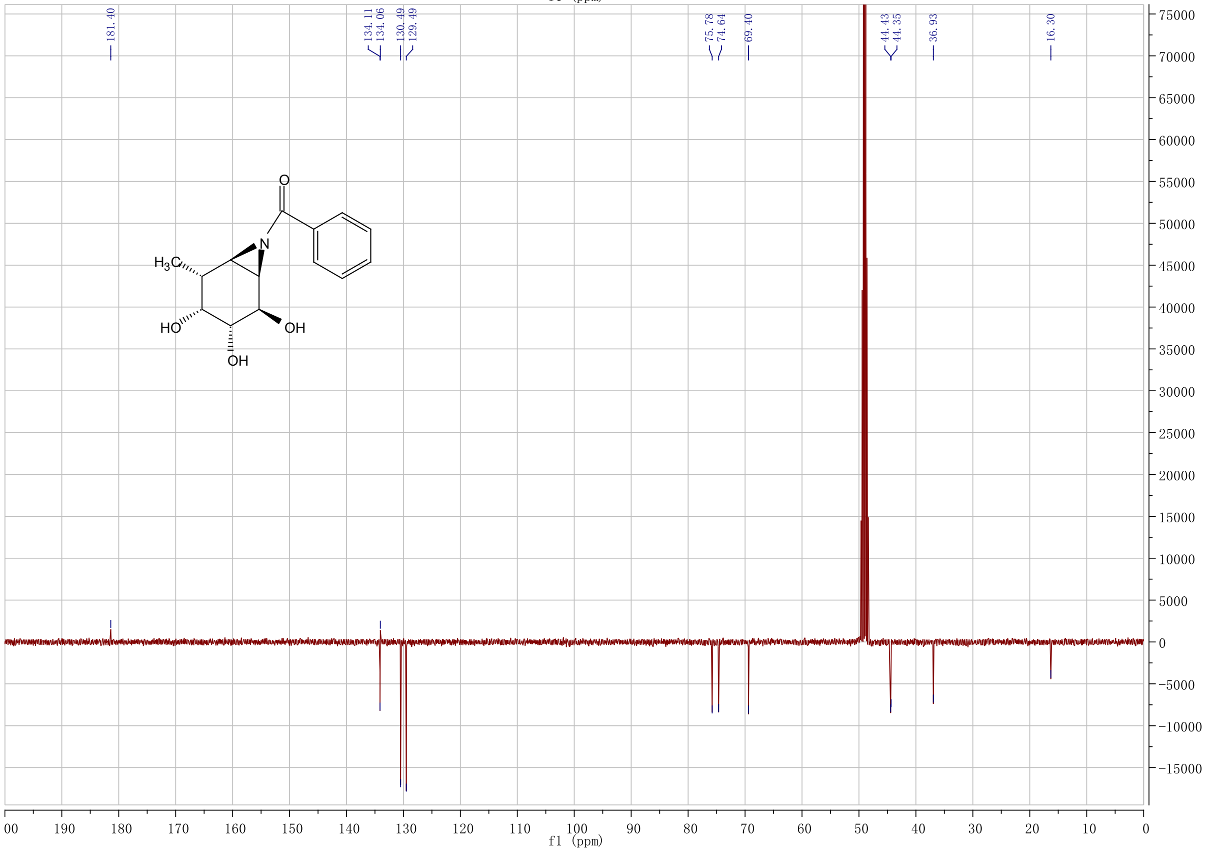

^{1}H and ^{13}C NMR (D_2O)

1-((1*R*,2*R*,3*R*,4*R*,5*R*,6*R*)-2,3,4-trihydroxy-5-methyl-7-azabicyclo[4.1.0]heptan-7-yl)ethan-1-one **(5)**

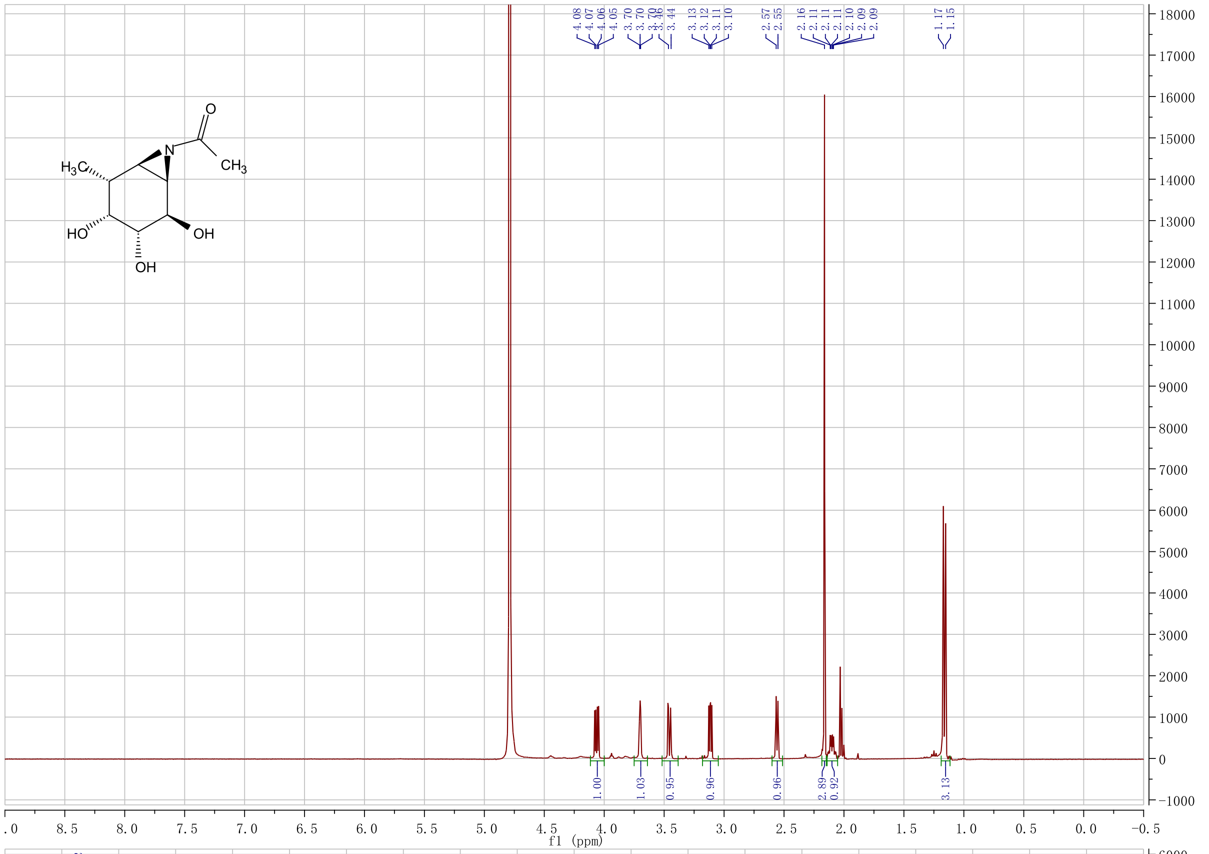

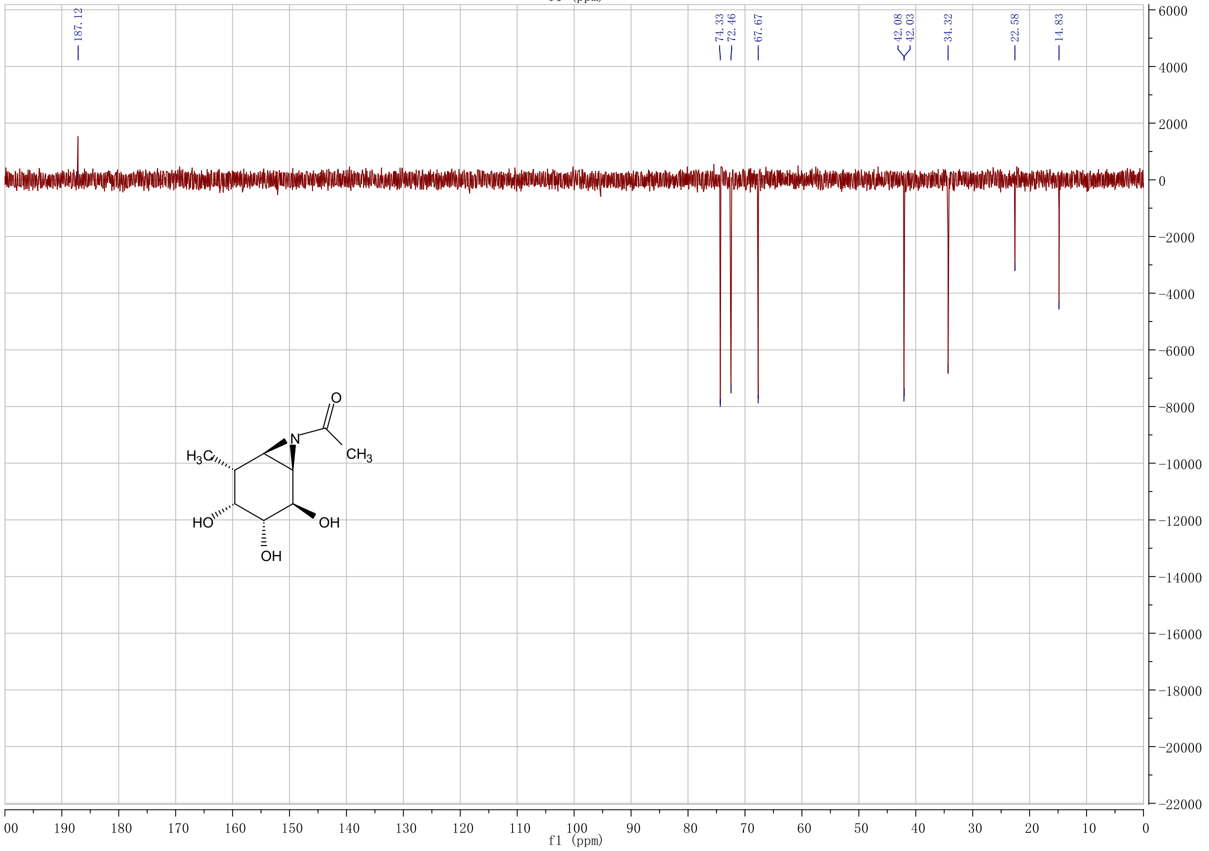

^{1}H NMR ($CDCl_3$): (2*S*,3*E*)-2-hydroxy-4-phenylbut-3-enenitrile

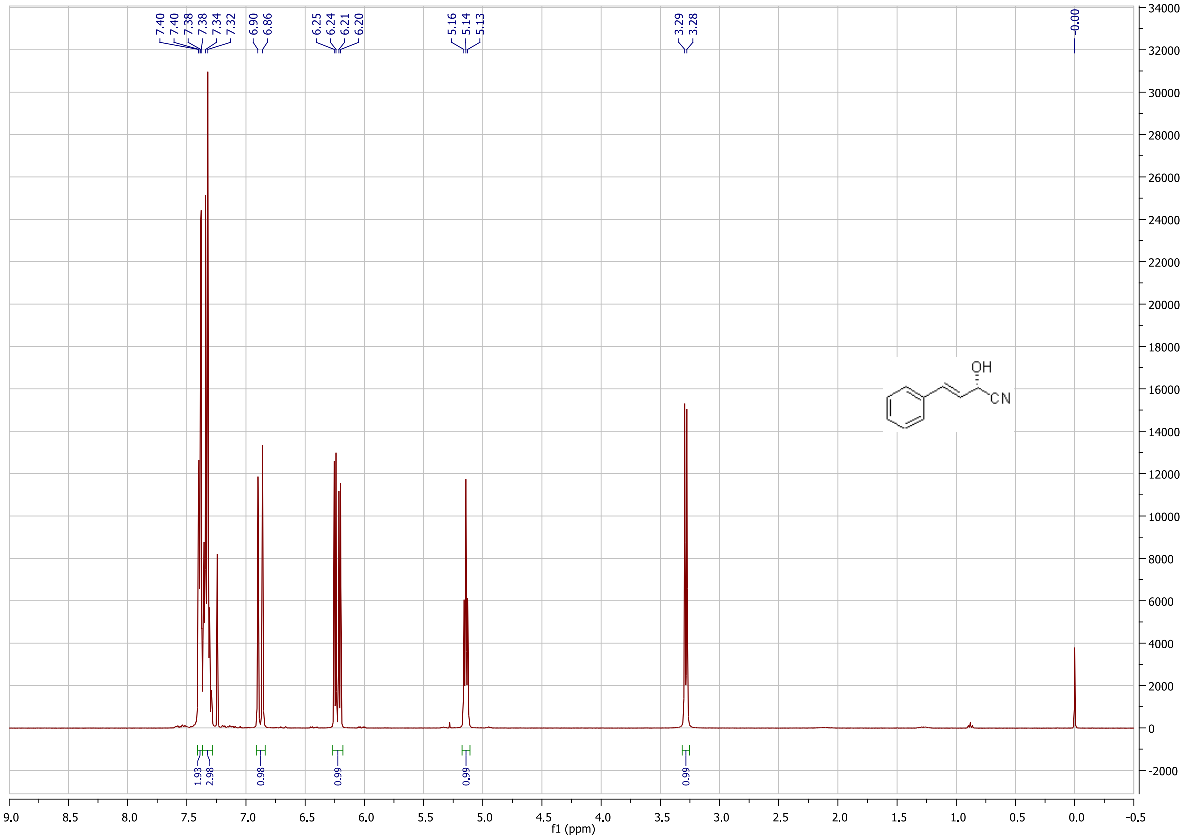

^{1}H NMR ($CDCl_3$): (*S*,*E*)-2-((*tert*-Butyldiphenylsilyl)oxy)-4-phenylbut-3-enenitrile(**(*S*)-28**)

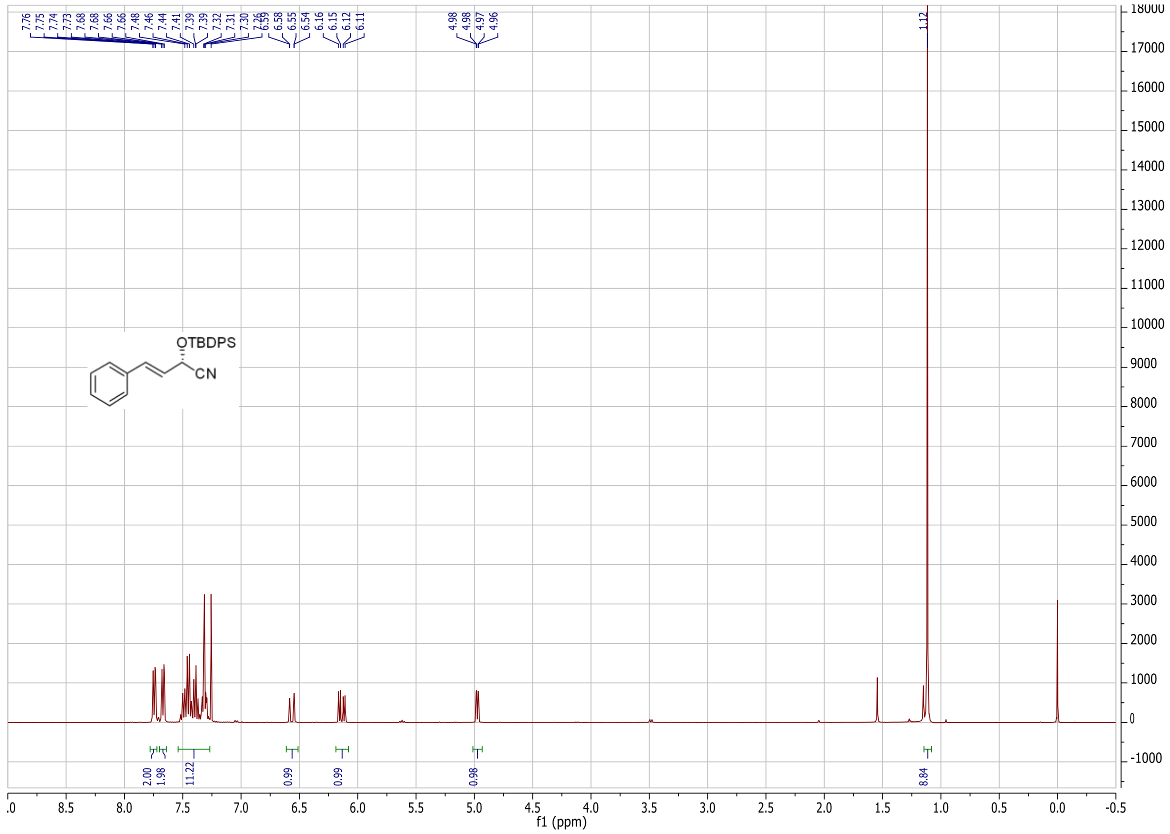

^{1}H NMR ($CDCl_3$): *tert*-Butyl (*S*)-(1-(methoxy(methyl)amino)-1-oxopropan-2-yl)carbamate

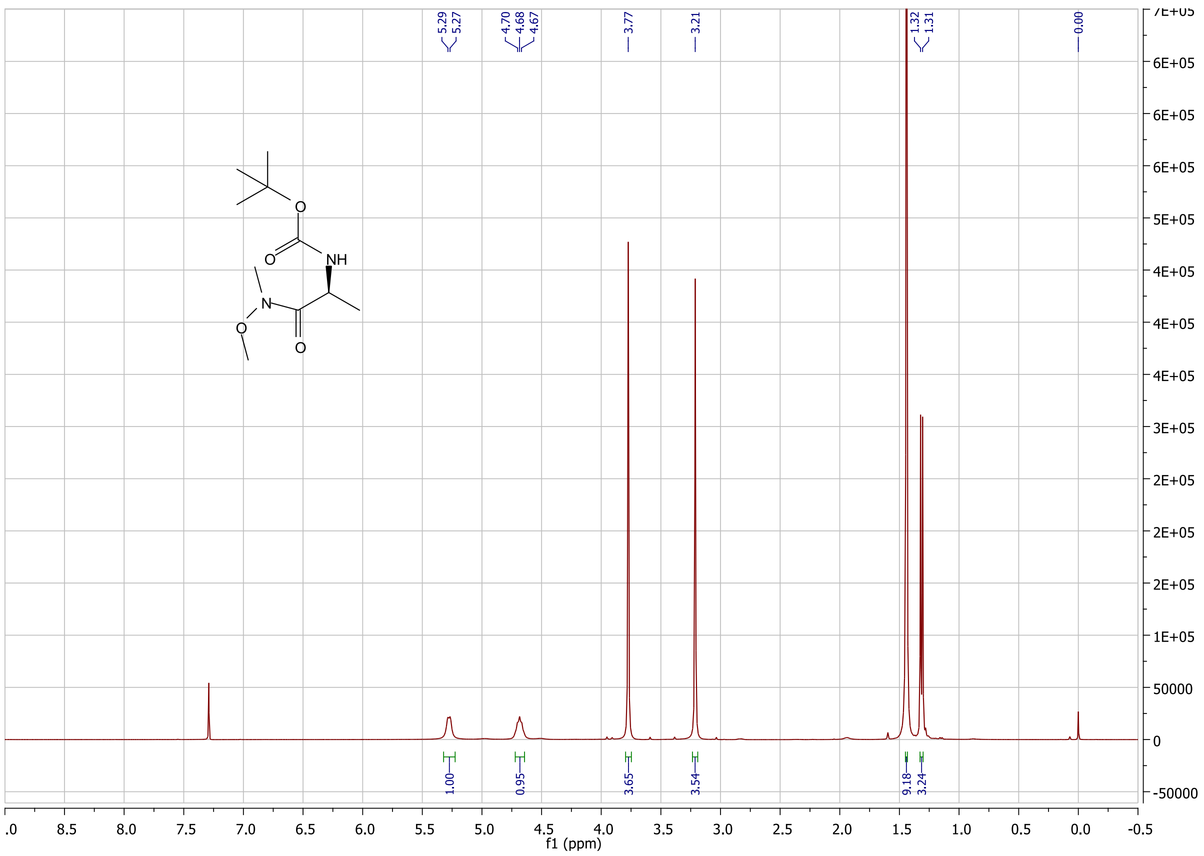

^{1}H NMR ($CDCl_3$): *tert-B*utyl (*S*)-(1-oxopropan-2-yl)carbamate

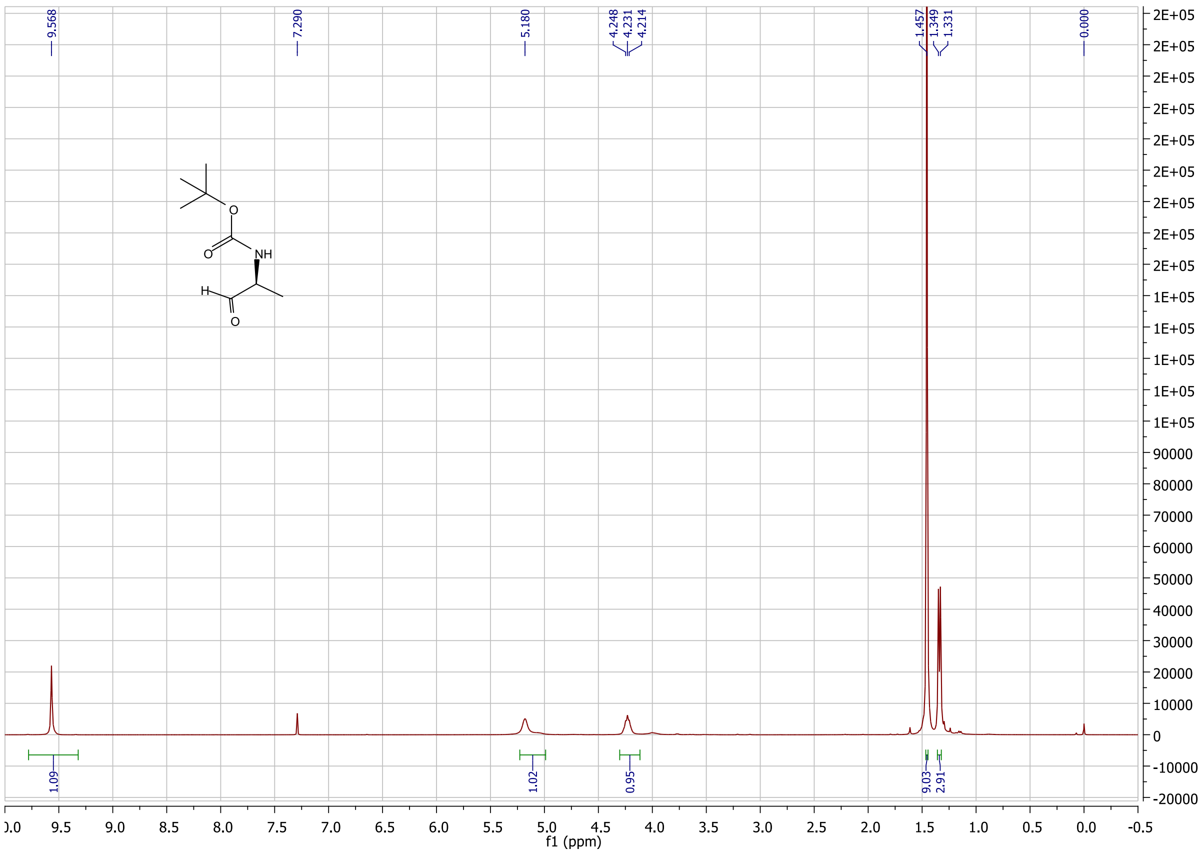

^1H and ^{13}C NMR ($CDCl_3$)

tert-Butyl (*S*)-but-3-en-2-ylcarbamate

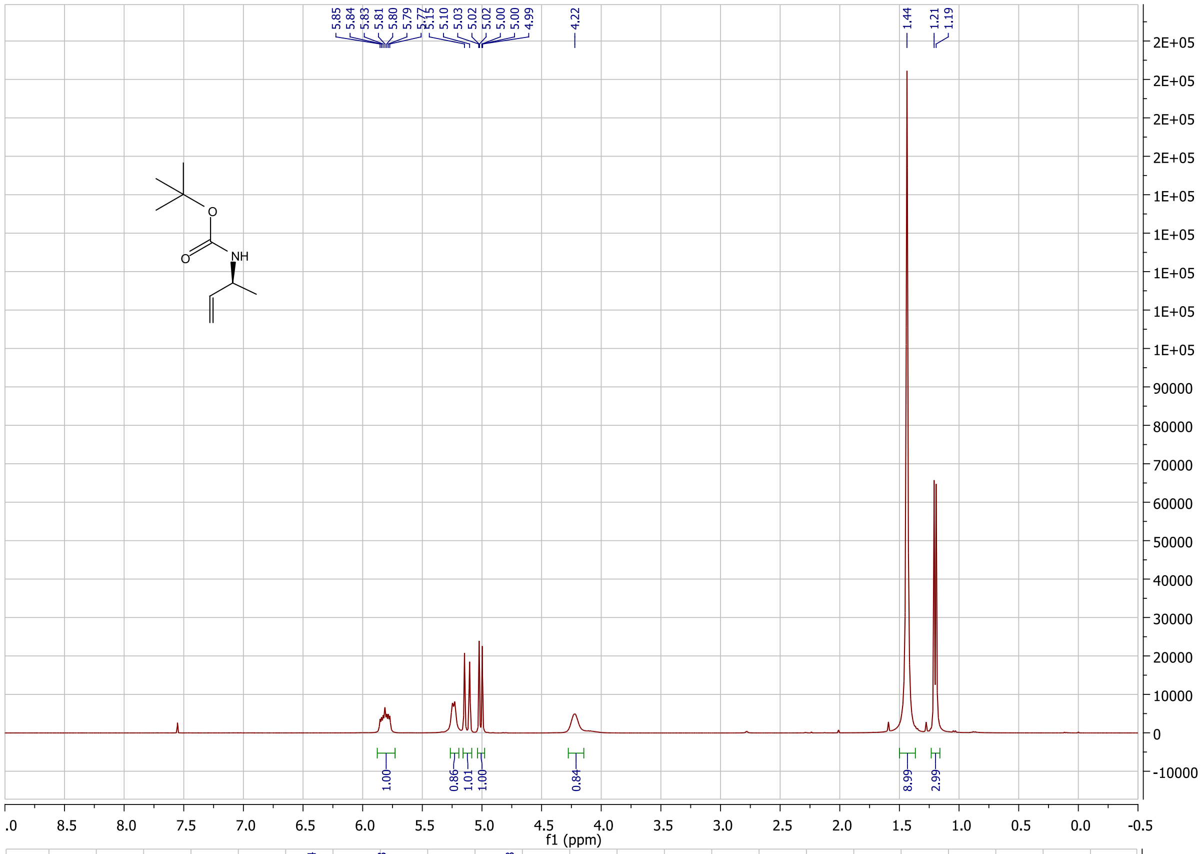

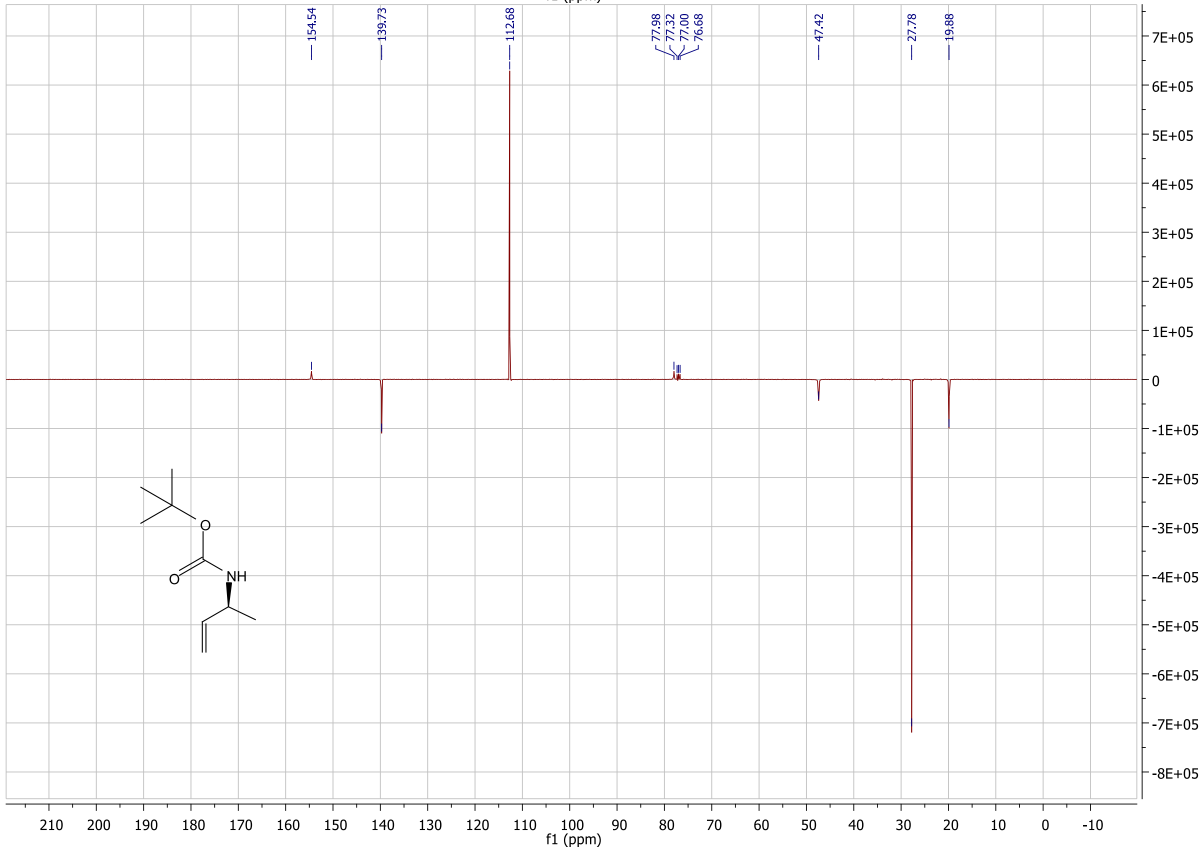

^1H and ^{13}C NMR (D_2O)

(*S*)-But-3-en-2-amine hydrochloride (**(*S*)-29.HCl**)

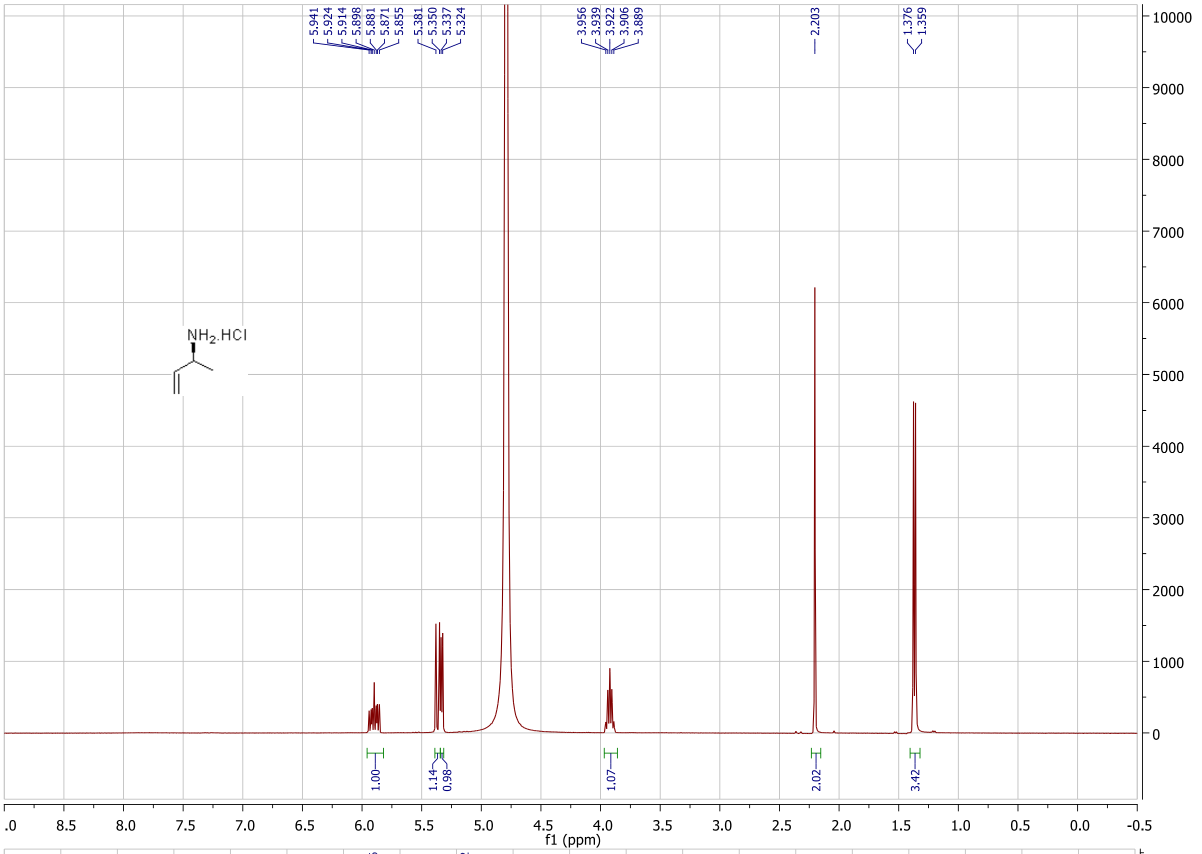

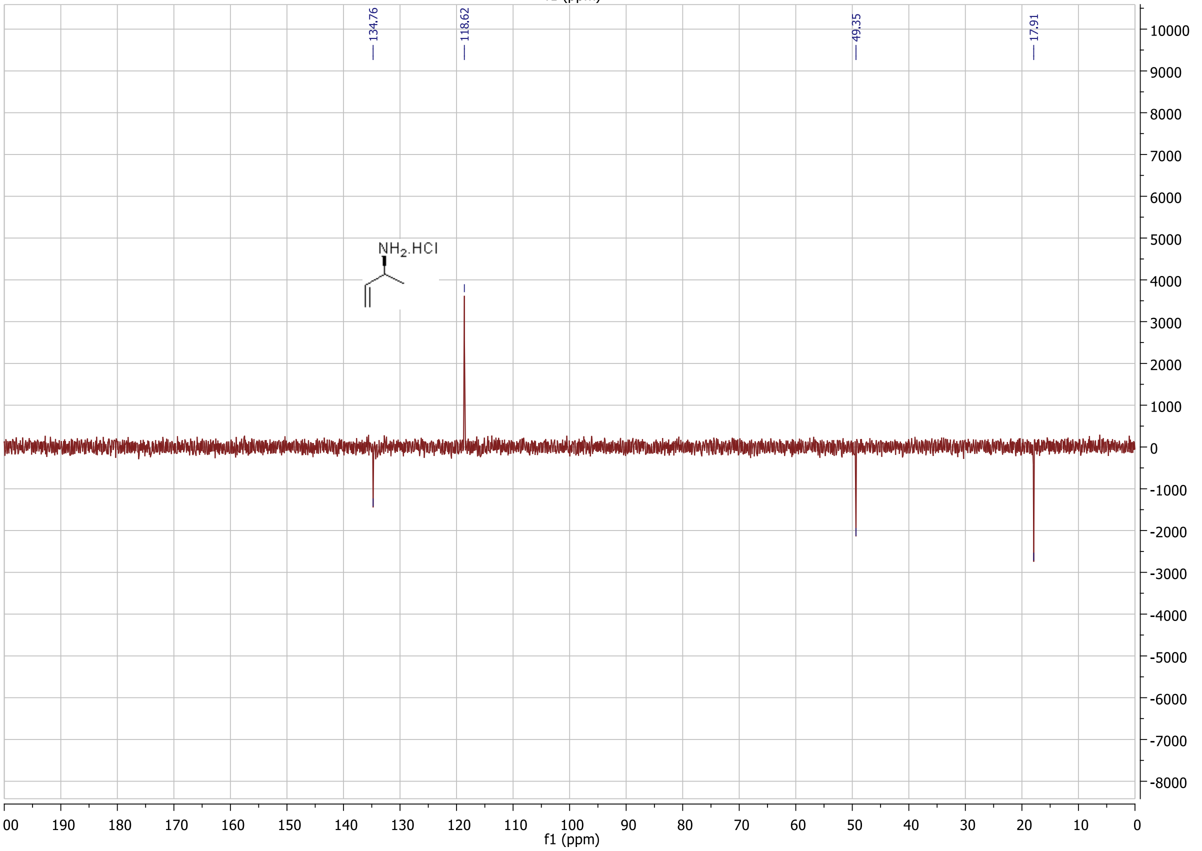

^{1}H and ^{13}C NMR ($CDCl_3$) compound **30**

(*S*,*E*)-*N*-((*S*)-But-3-en-2-yl)-2-((*tert*-butyldiphenylsilyl)oxy)-4-phenylbut-3-en-1-amine

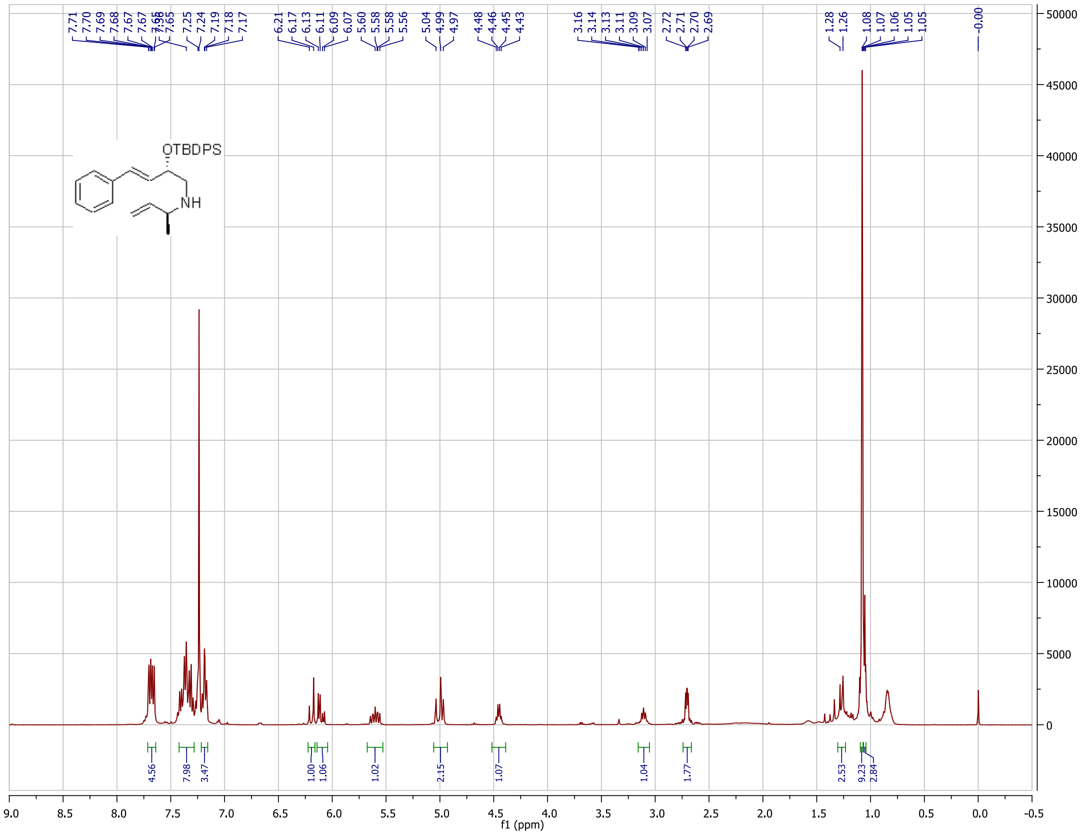

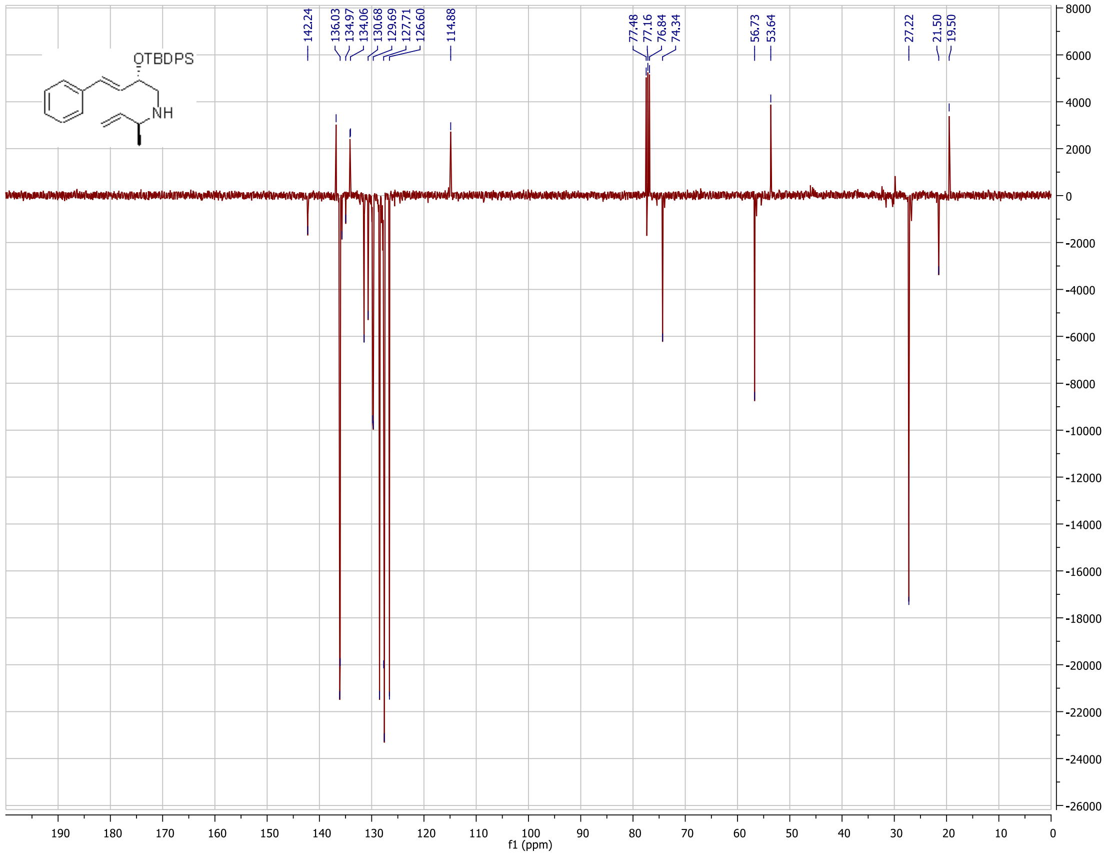

^{1}H and ^{13}C NMR ($CDCl_3$) compound **31**

tert-Butyl((*S*)-but-3-en-2-yl)((*S*,*E*)-2-((*tert*-butyldiphenylsilyl)oxy)-4-phenylbut-3-en-1-yl)carbamate

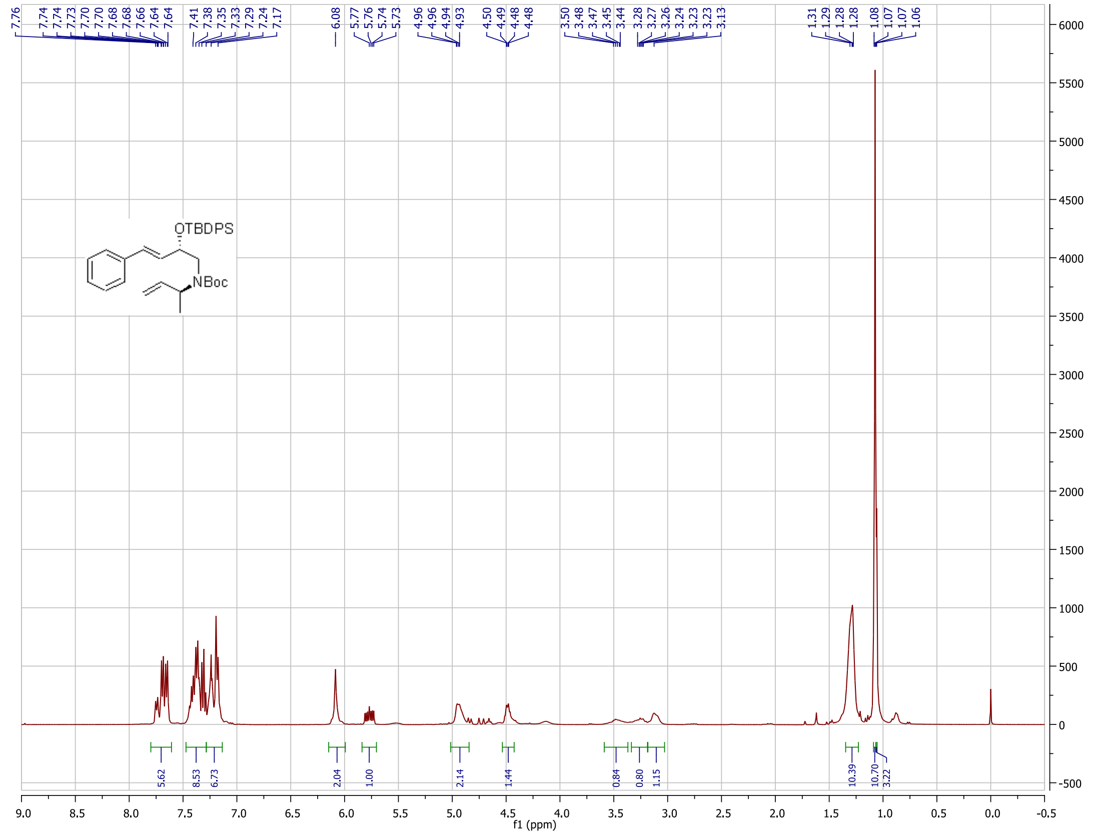

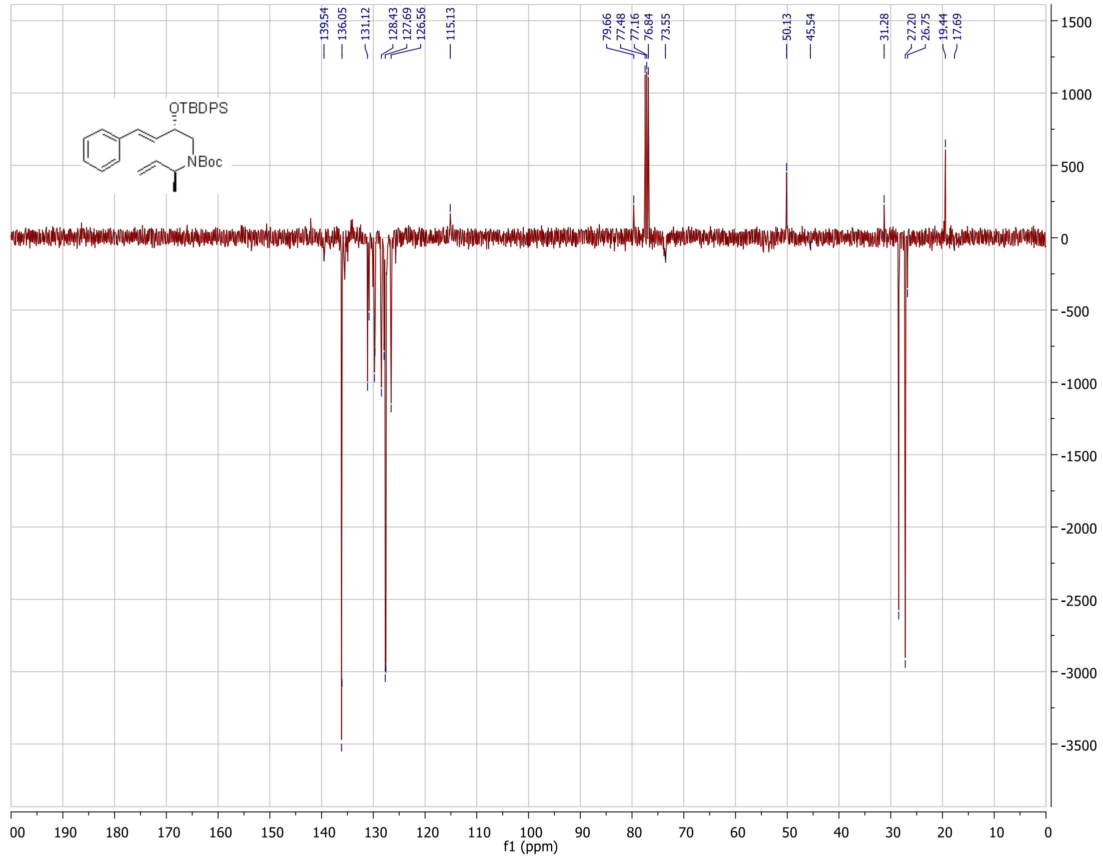

^{1}H and ^{13}C NMR ($CDCl_3$) compound **32**

tert-Butyl (3*S*,6*S*)-3-((*tert*-butyldiphenylsilyl)oxy)-6-methyl-3,6-dihydropyridine-1(2*H*)-carboxylate

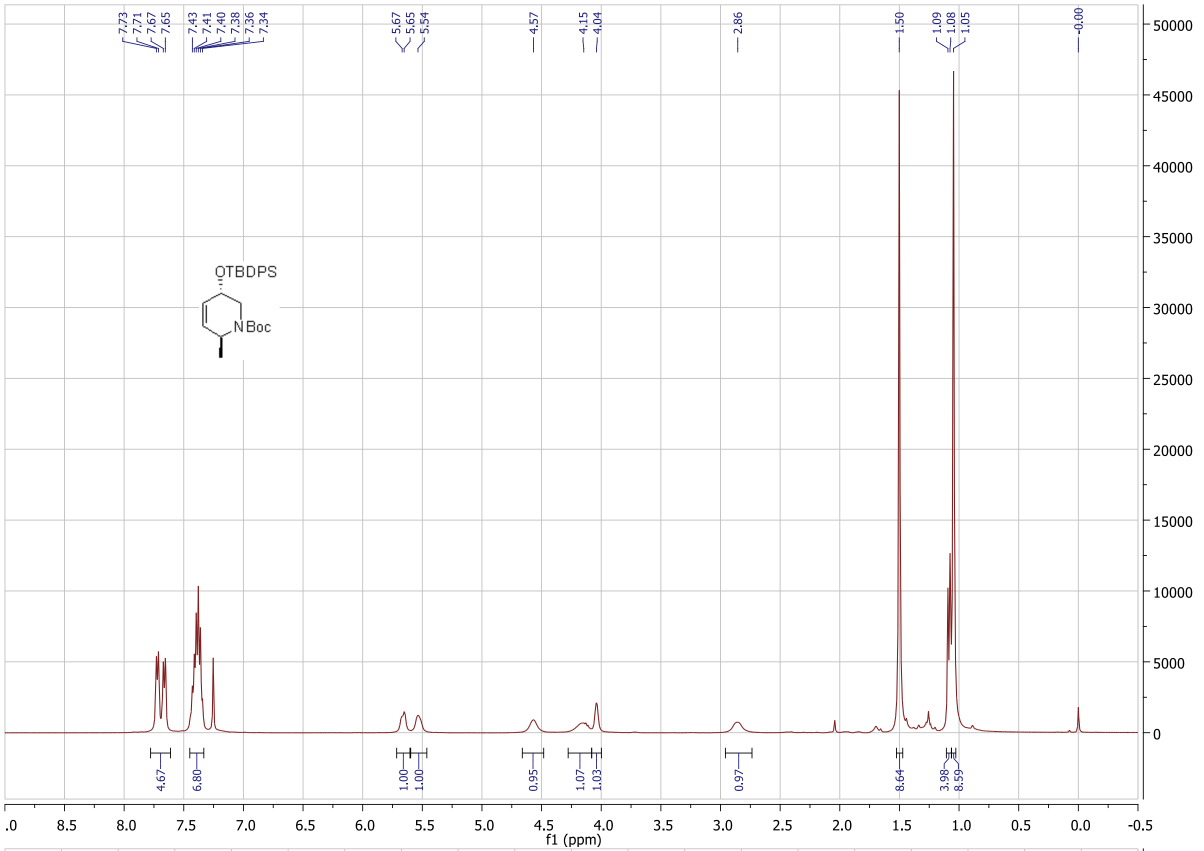

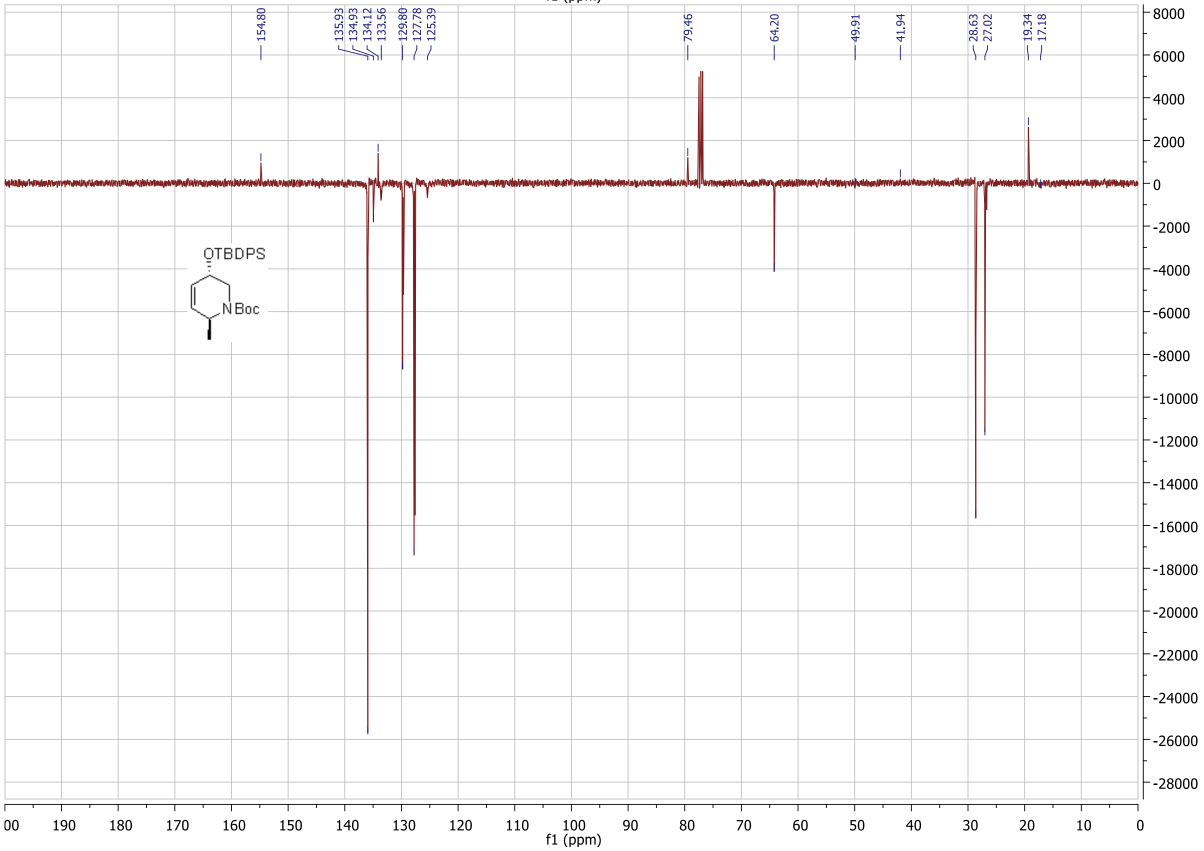

^{1}H and ^{13}C NMR ($CDCl_3$) compound **33**

(2*S*,3*S*,4*S*,5*R*)-1-(*tert*-butoxycarbonyl)-5-((*tert*-butyldiphenylsilyl)oxy)-2-methylpiperidine-3,4-diyl diacetate

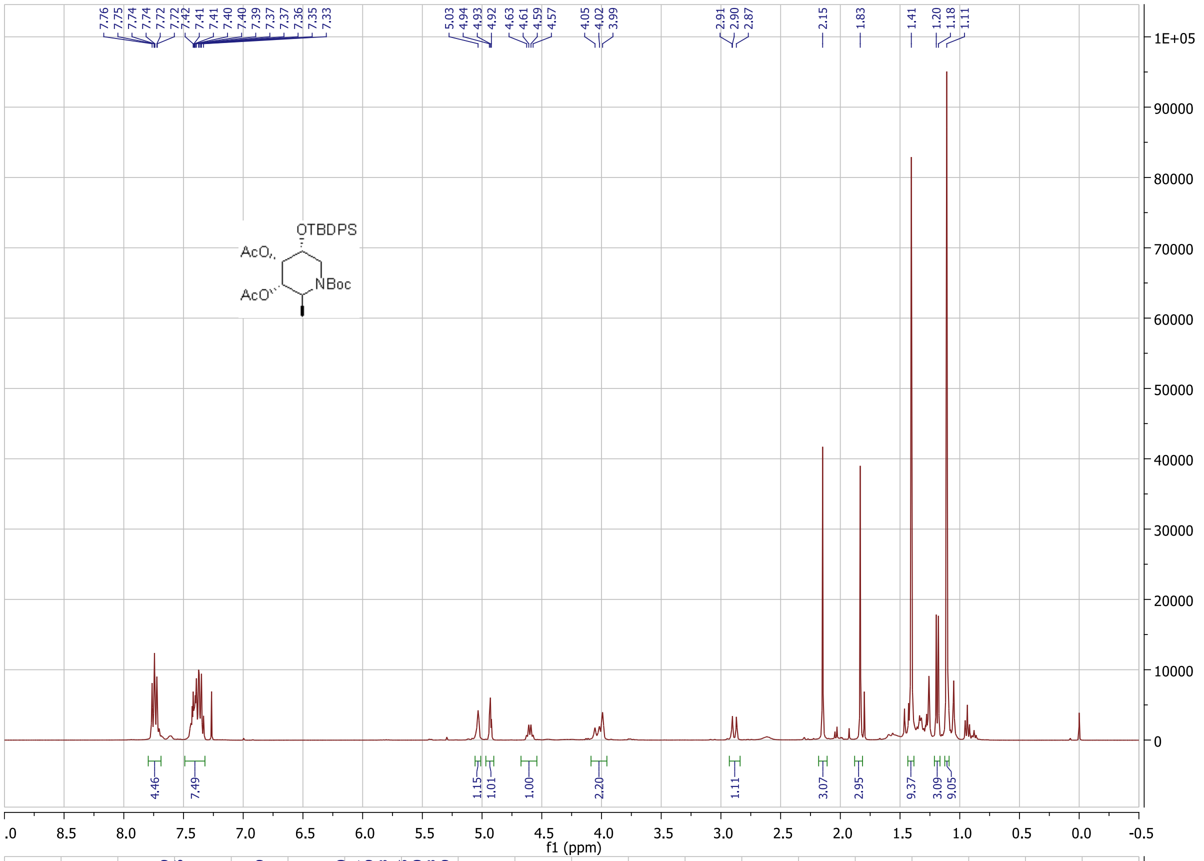

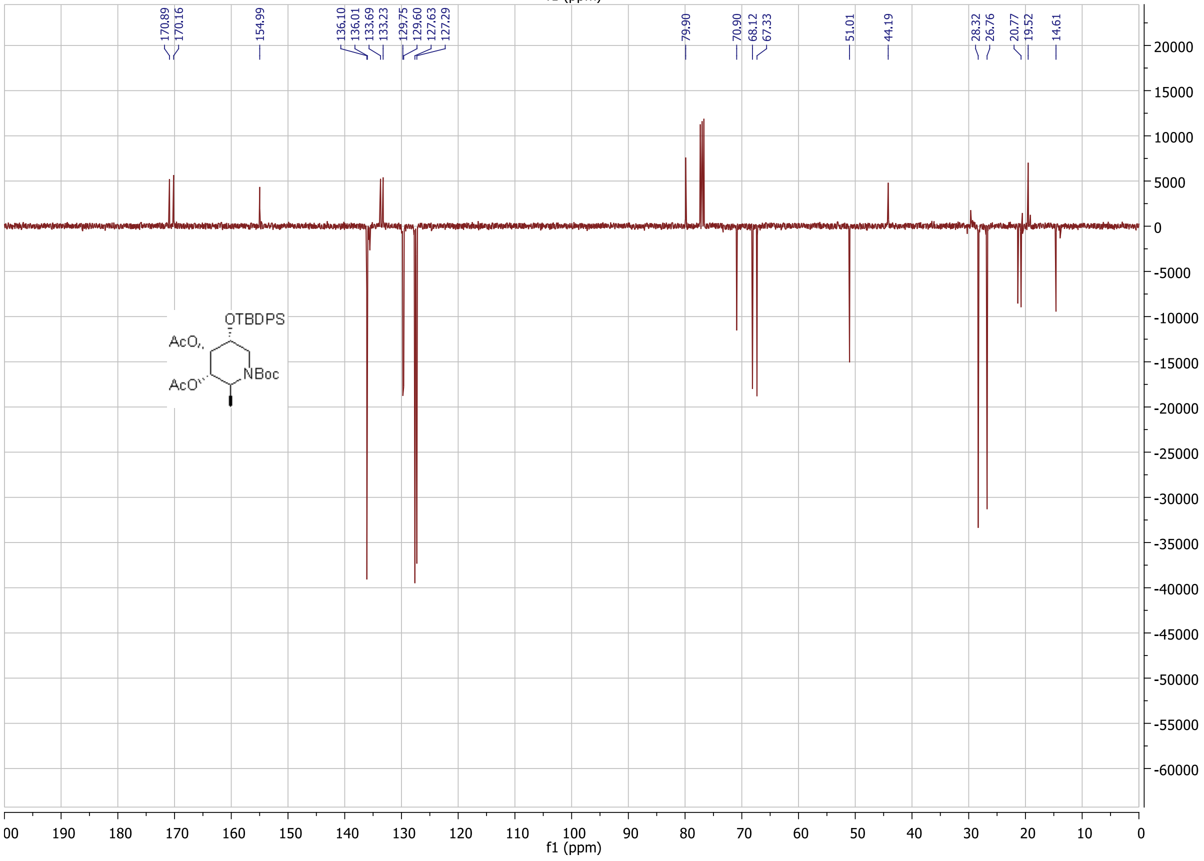

^{1}H and ^{13}C NMR ($CDCl_3$) compound **34**

(2*S*,3*R*,4*R*,5*R*)-1-(*tert*-butoxycarbonyl)-5-((*tert*-butyldiphenylsilyl)oxy)-2-methylpiperidine-3,4-diyl diacetate

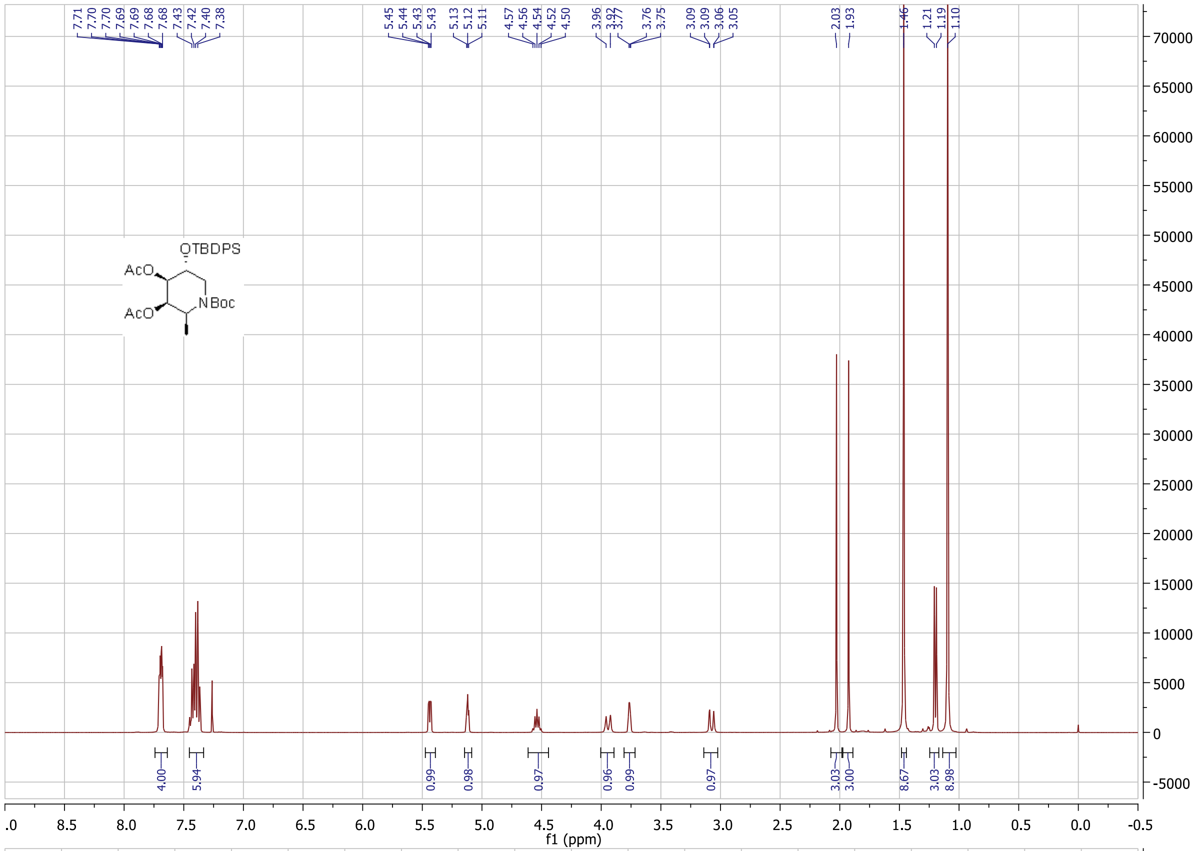

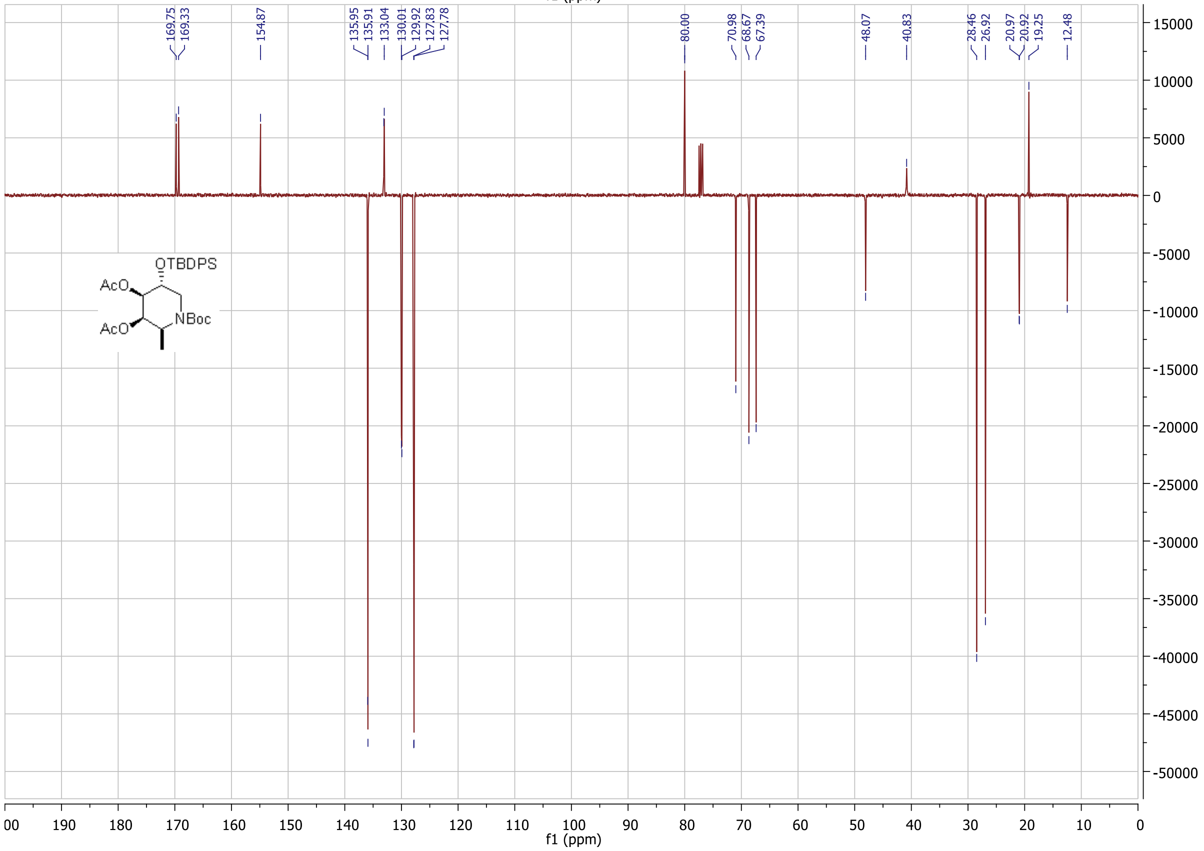

^{1}H and ^{13}C NMR ($CDCl_3$)

tert-Butyl (2*S*,3*R*,4*R*,5*R*)-5-((*tert*-butyldiphenylsilyl)oxy)-3,4-dihydroxy-2-methylpiperidine-1-carboxylate

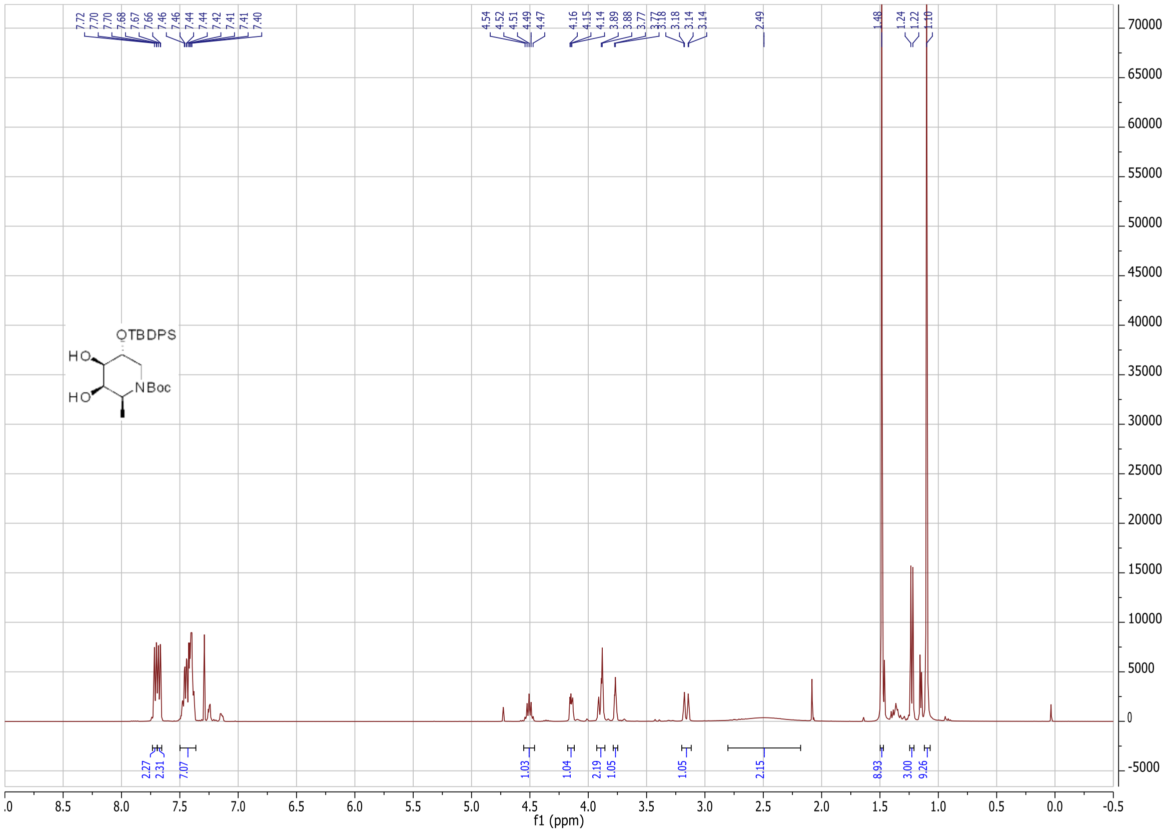

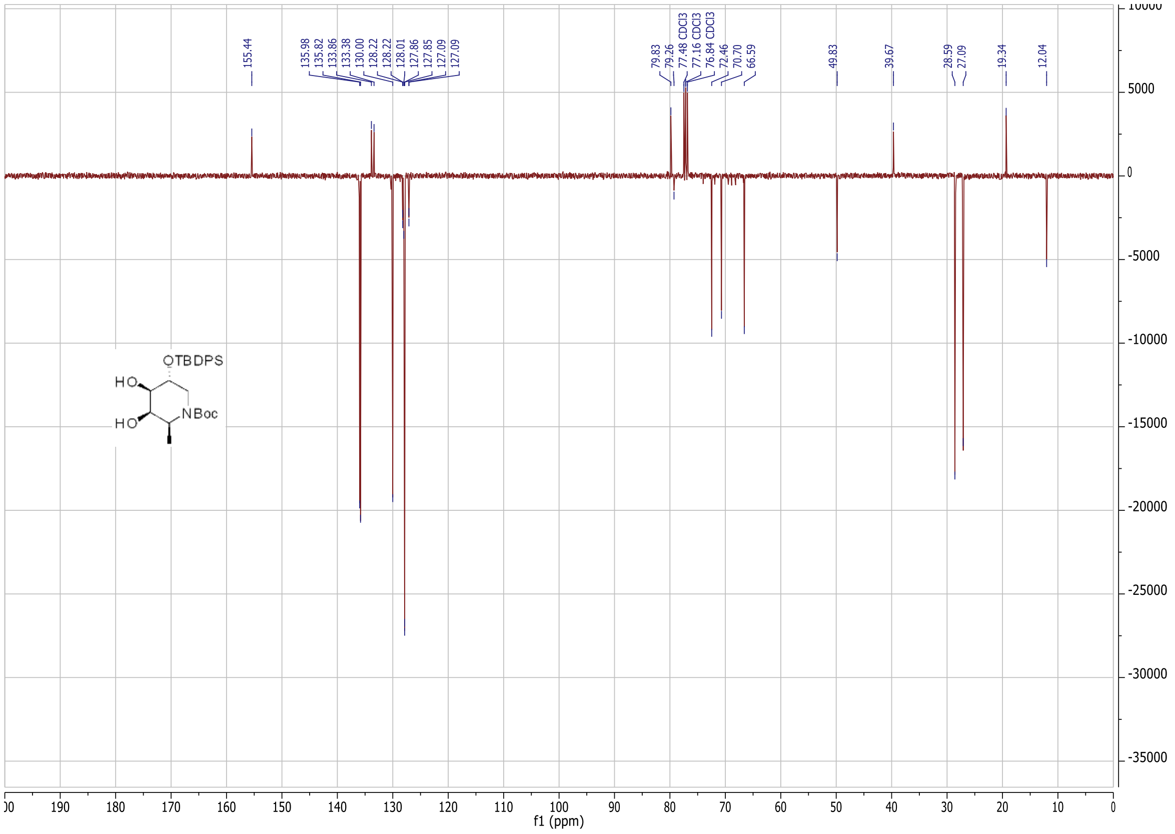

^1H and ^{13}C NMR (MeOH-d_4)

tert-Butyl (2*S*,3*R*,4*S*,5*R*)-3,4,5-trihydroxy-2-methylpiperidine-1-carboxylate

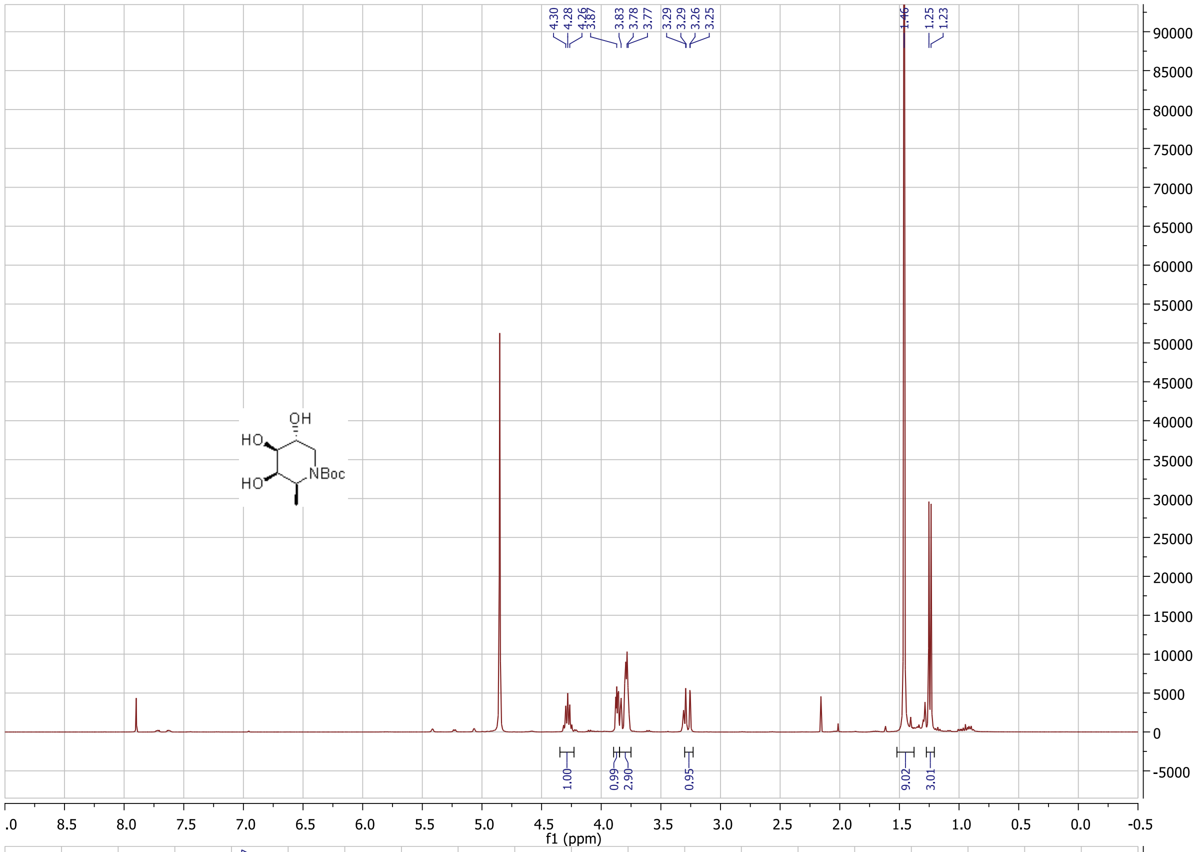

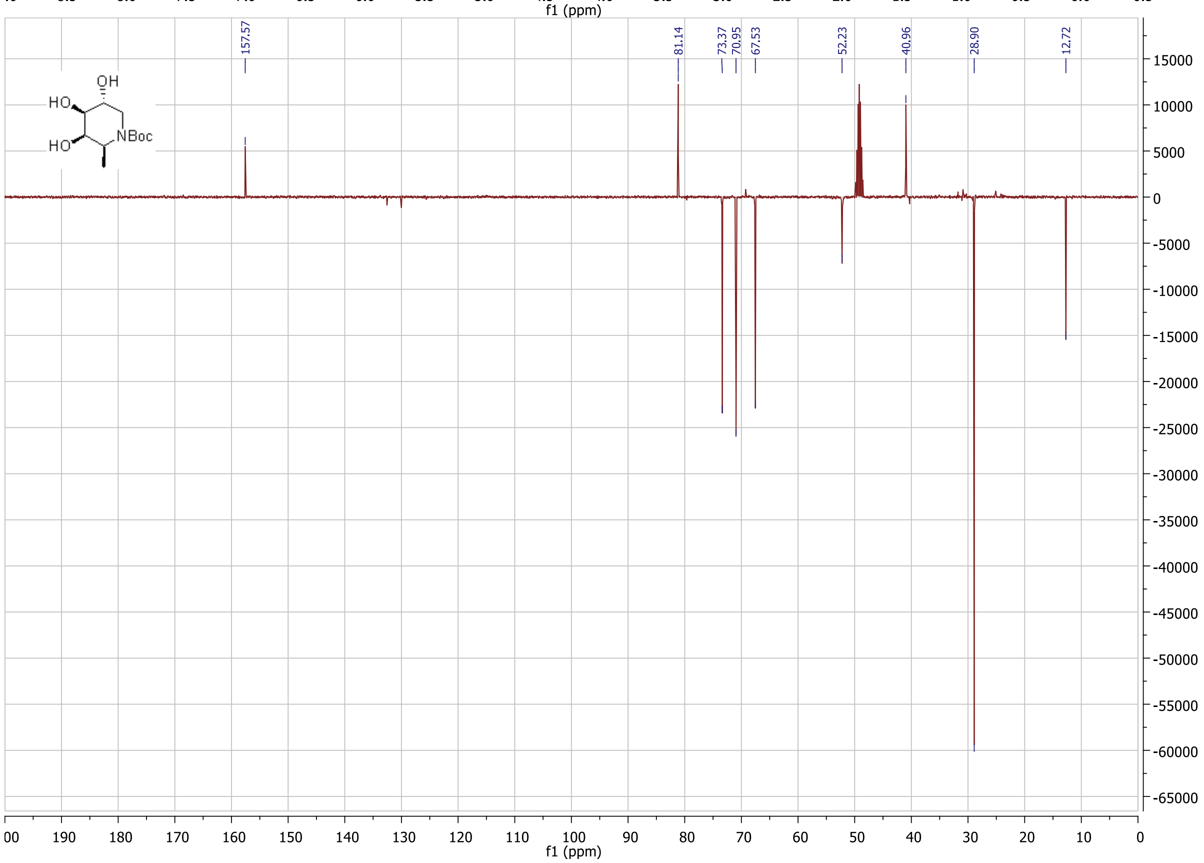

^{1}H and ^{13}C NMR (D_2O) fuconojirimycin (6.**HCl**)

(2*S*,3*R*,4*S*,5*R*)-2-methylpiperidine-3,4,5-triol hydrochloride (**6**)

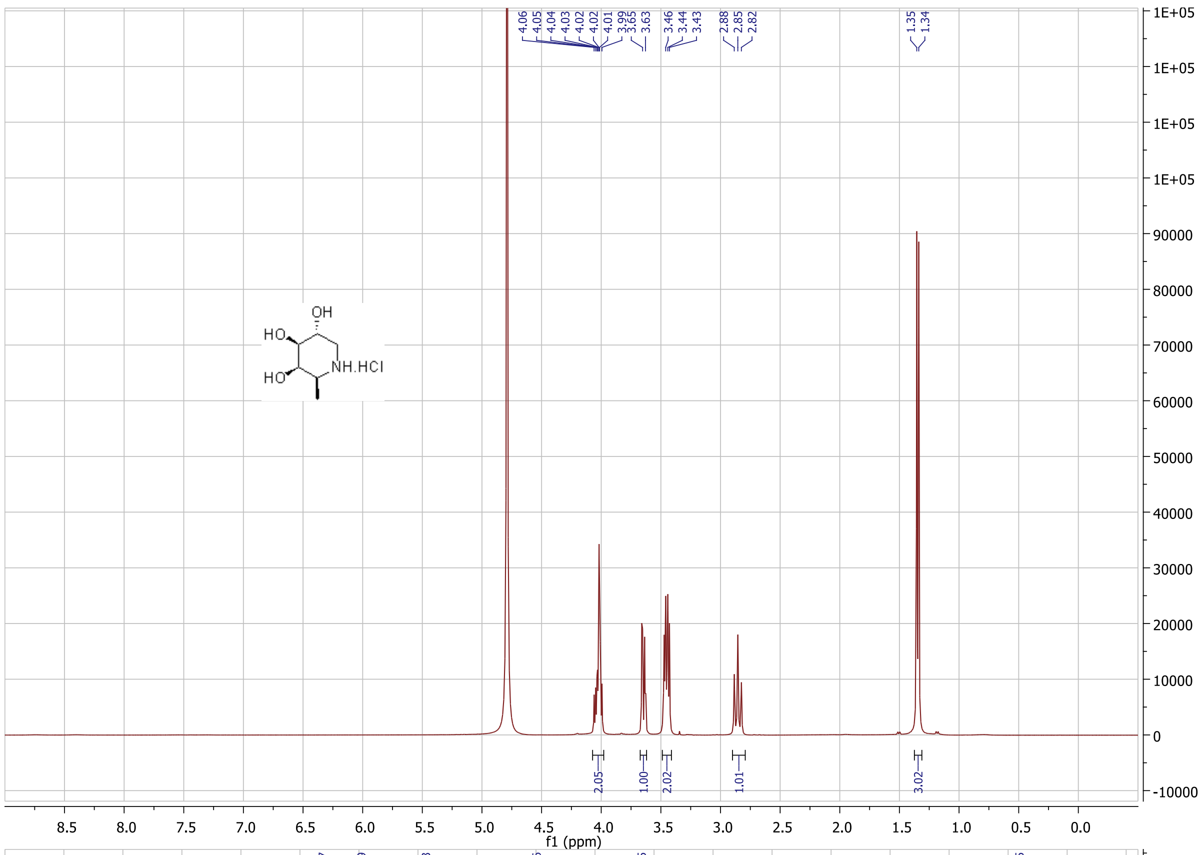

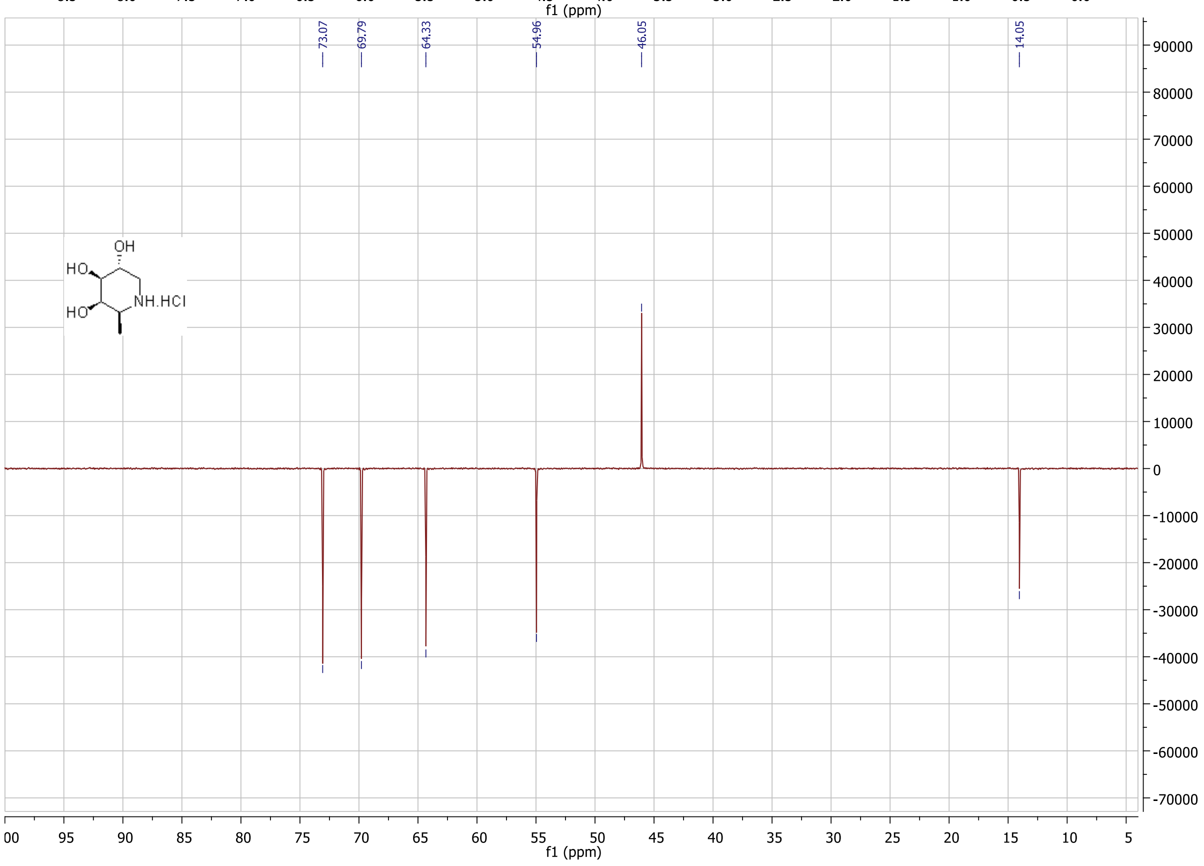

^{1}H and ^{13}C NMR ($CDCl_3$) compound (**35**)

tert-Butyl (3a*R*,4*S*,7*R*,7a*S*)-7-hydroxy-2,2,4-trimethyltetrahydro-[1,3]dioxolo[4,5-c]pyridine-5(4*H*)-carboxylate

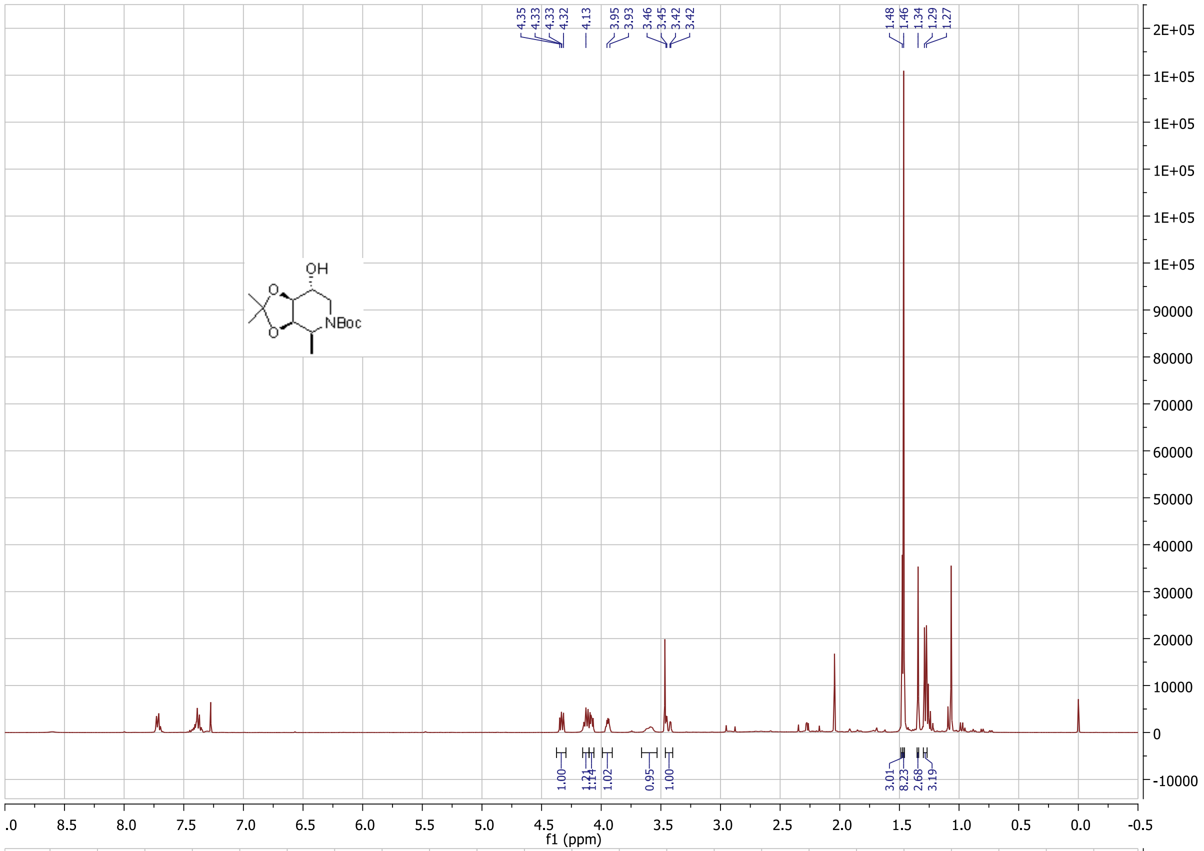

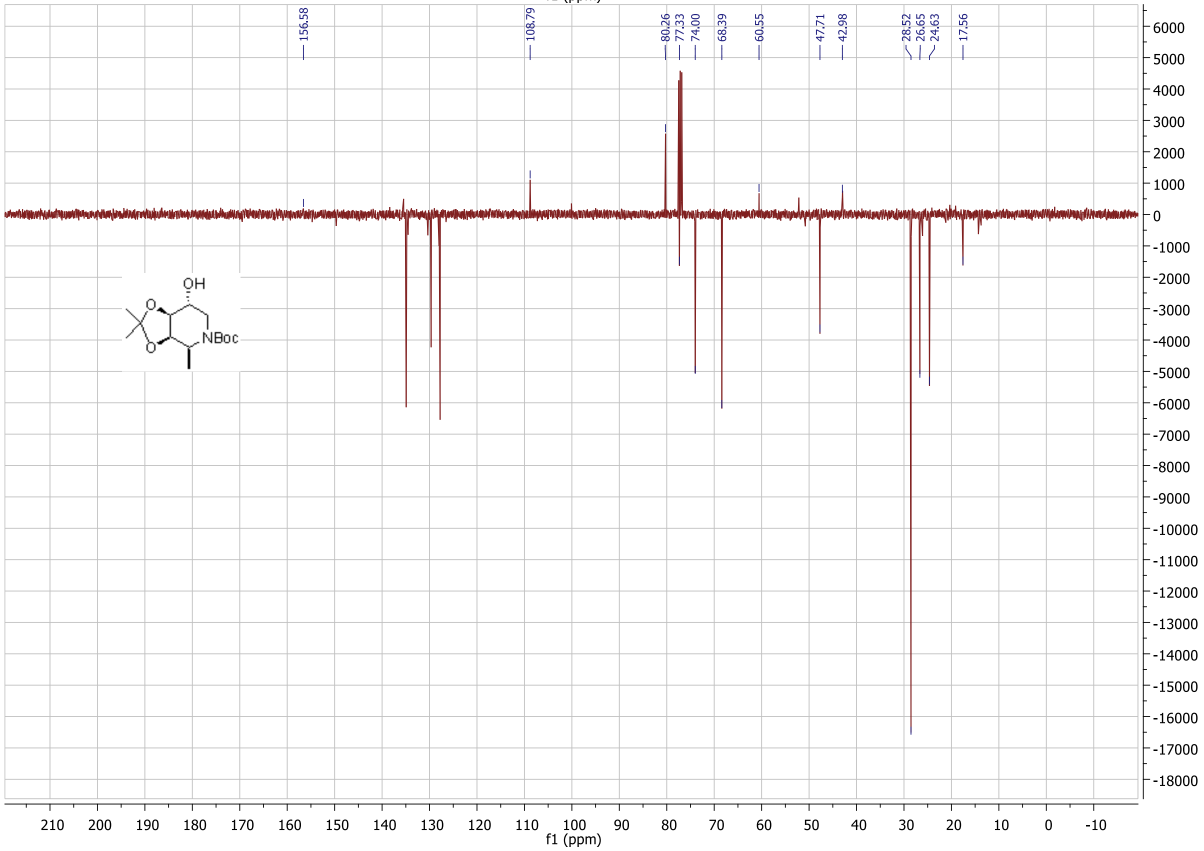

^{1}H and ^{13}C NMR ($CDCl_3$) compound (**36**)

tert-Butyl (3a*R*,4*S*,7*S*,7a*S*)-7-hydroxy-2,2,4-trimethyltetrahydro-[1,3]dioxolo[4,5-c]pyridine -5(4*H*)-carboxylate

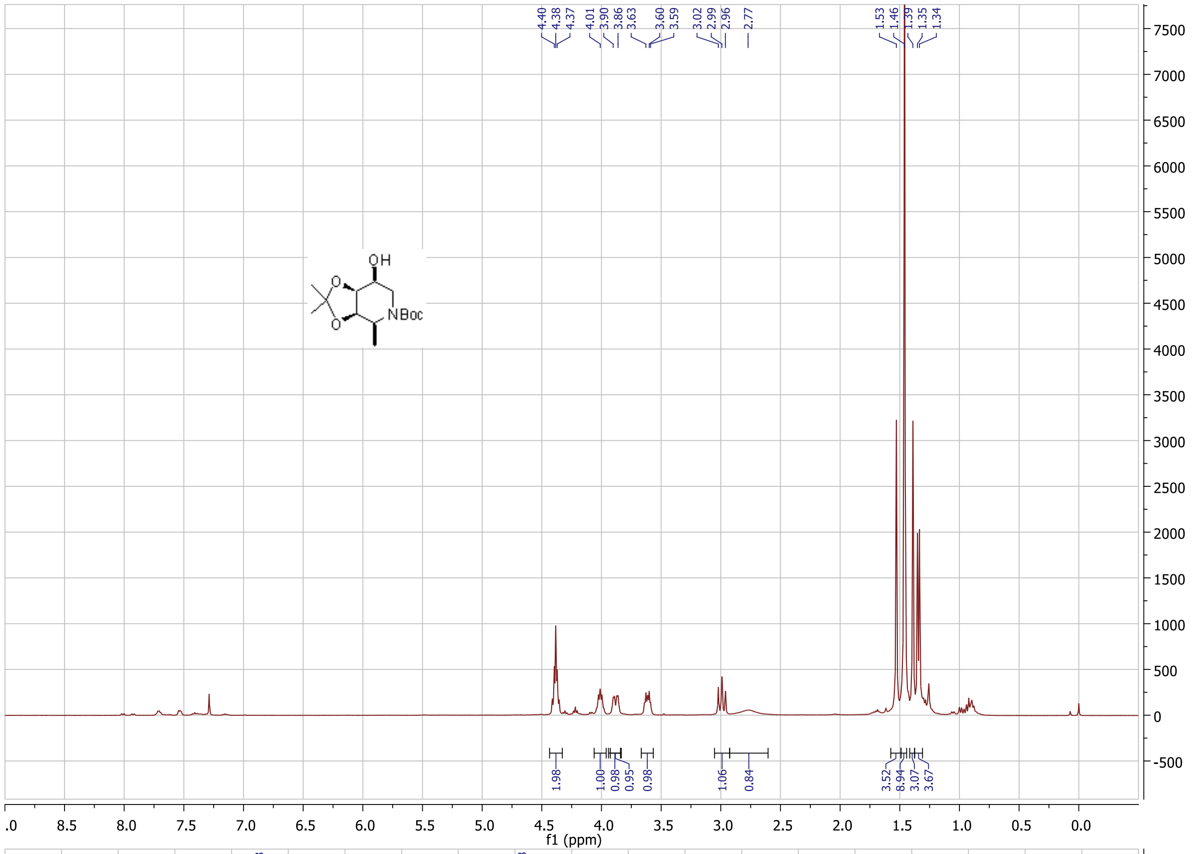

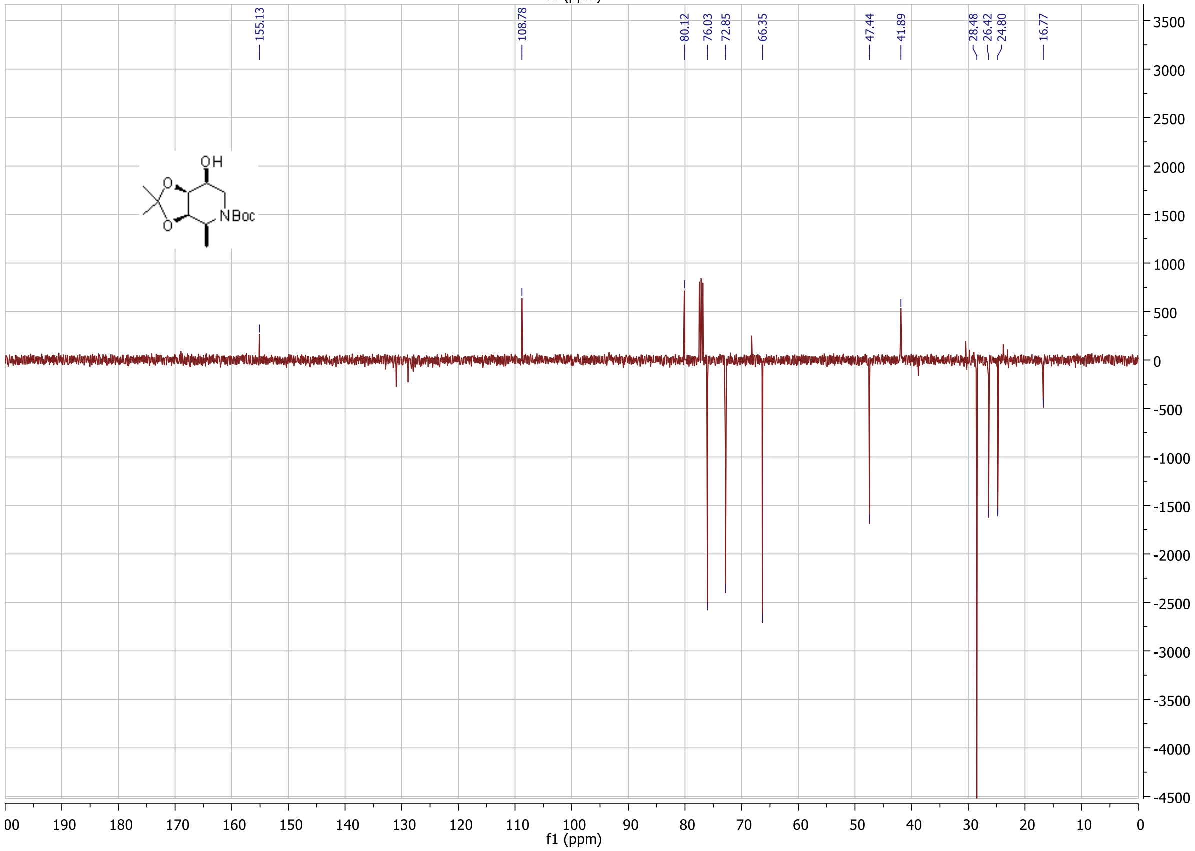

^{1}H and ^{13}C NMR (D_2O)

(2*S*,3*R*,4*S*,5*S*)-2-methylpiperidine-3,4,5-triol hydrochloride (**7.HCl**)

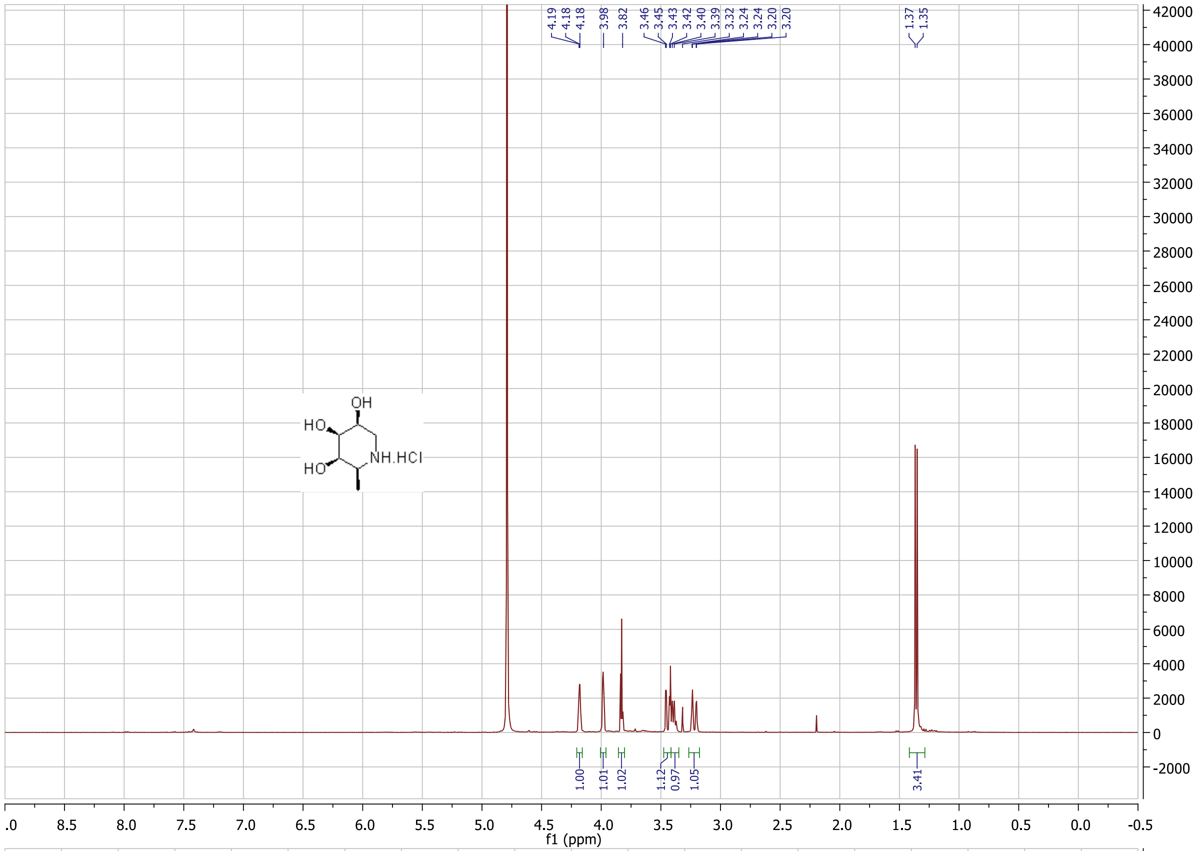

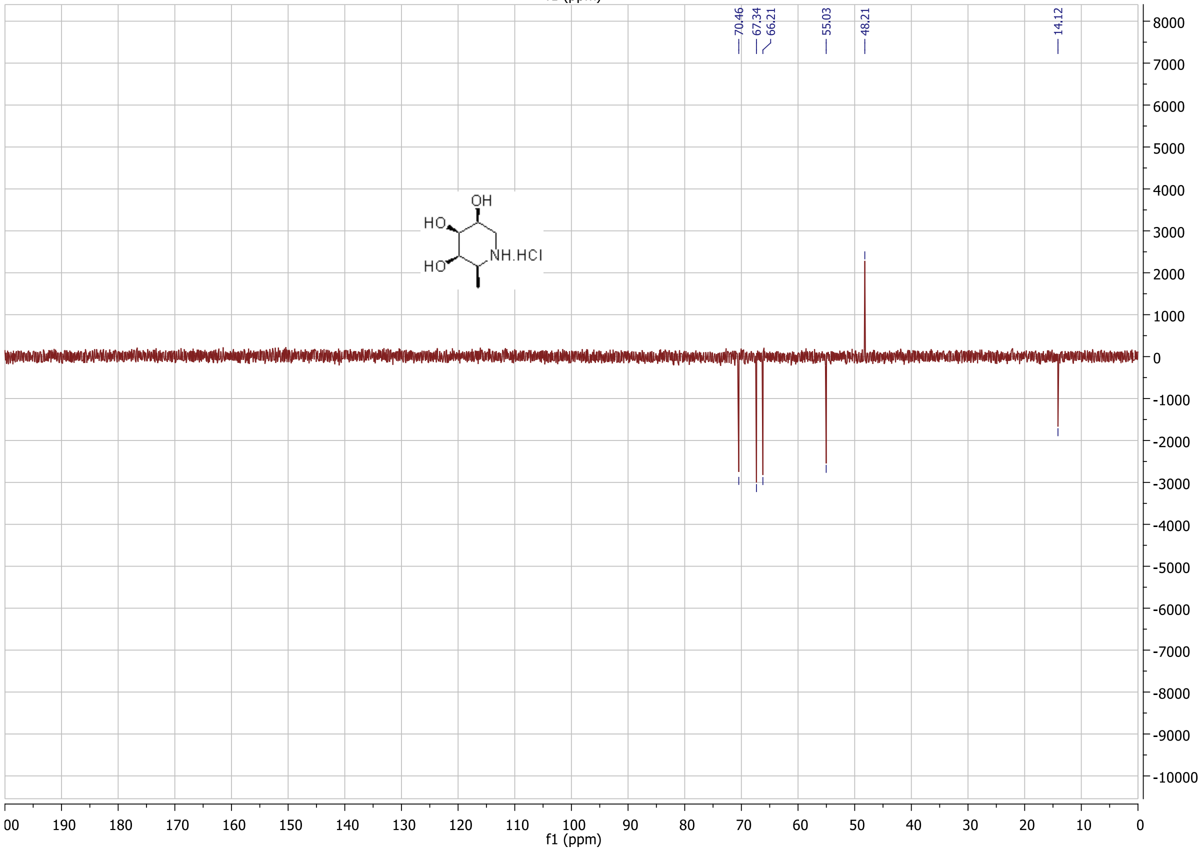

^{1}H and ^{13}C NMR ($CDCl_3$)

(*R*,*E*)-*N*-((*S*)-But-3-en-2-yl)-2-((*tert*-butyldiphenylsilyl)oxy)-4-phenylbut-3-en-1-amine (**37**)

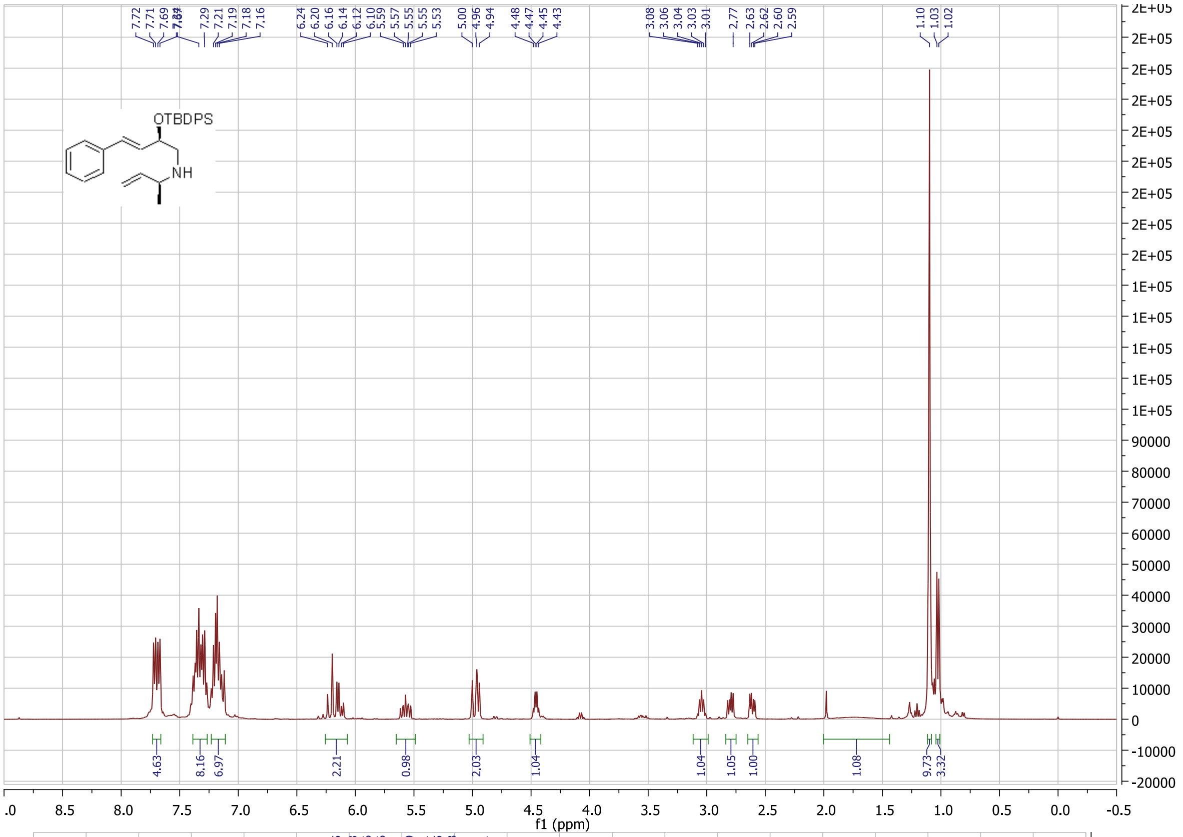

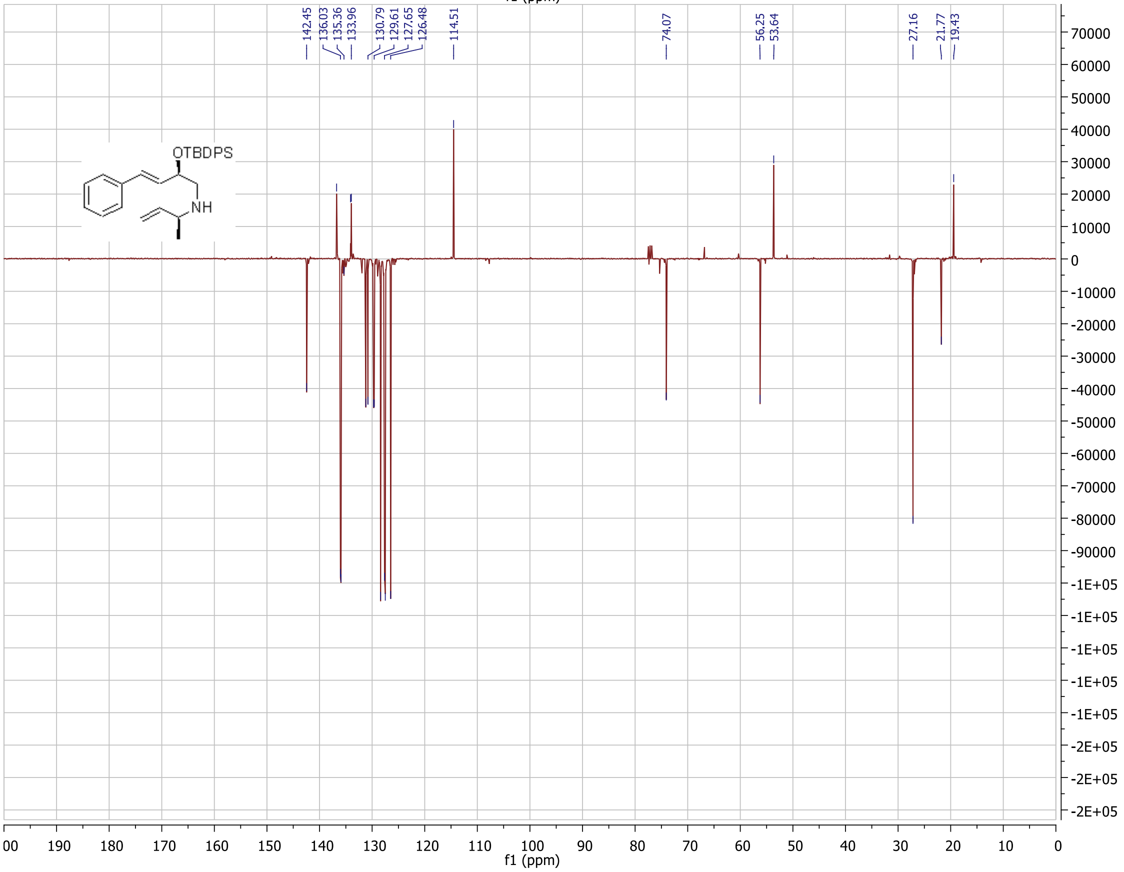

^1H and ^{13}C NMR ($CDCl_3$)

tert-Butyl((*S*)-but-3-en-2-yl)((*R*,*E*)-2-((*tert*-butyldiphenylsilyl)oxy)-4-phenylbut-3-en-1-yl)carbamate:

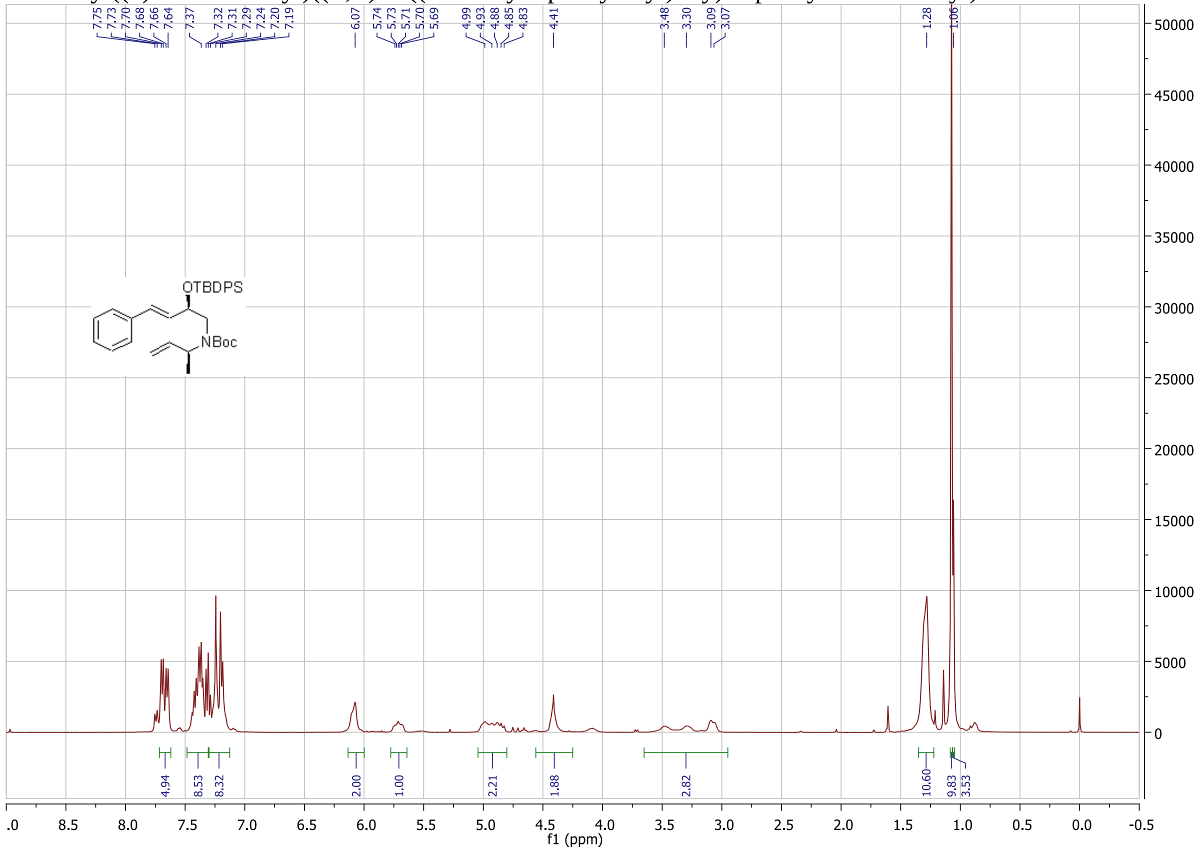

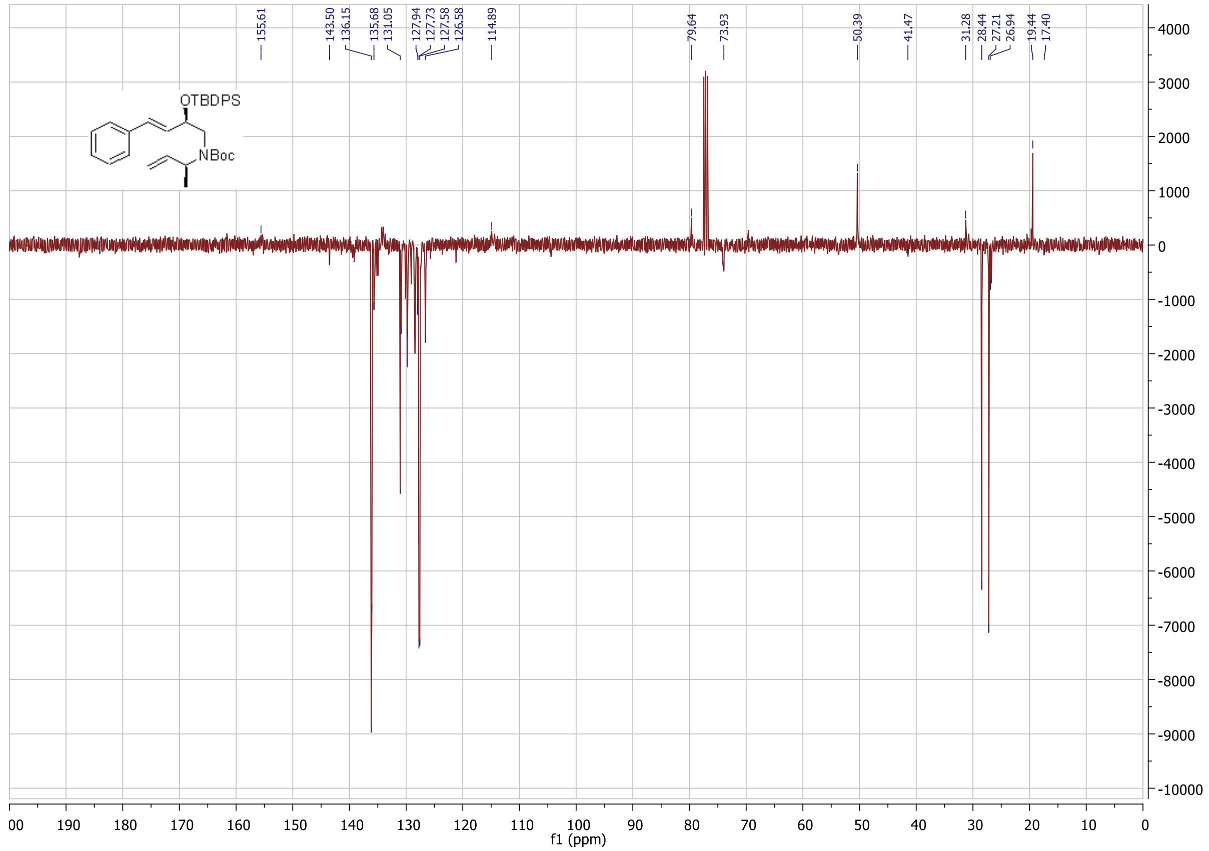

^{1}H and ^{13}C NMR ($CDCl_3$)

tert-Butyl (3*R*,6*S*)-3-((*tert*-butyldiphenylsilyl)oxy)-6-methyl-3,6-dihydropyridine-1(2*H*)-carboxy-late **(38)**

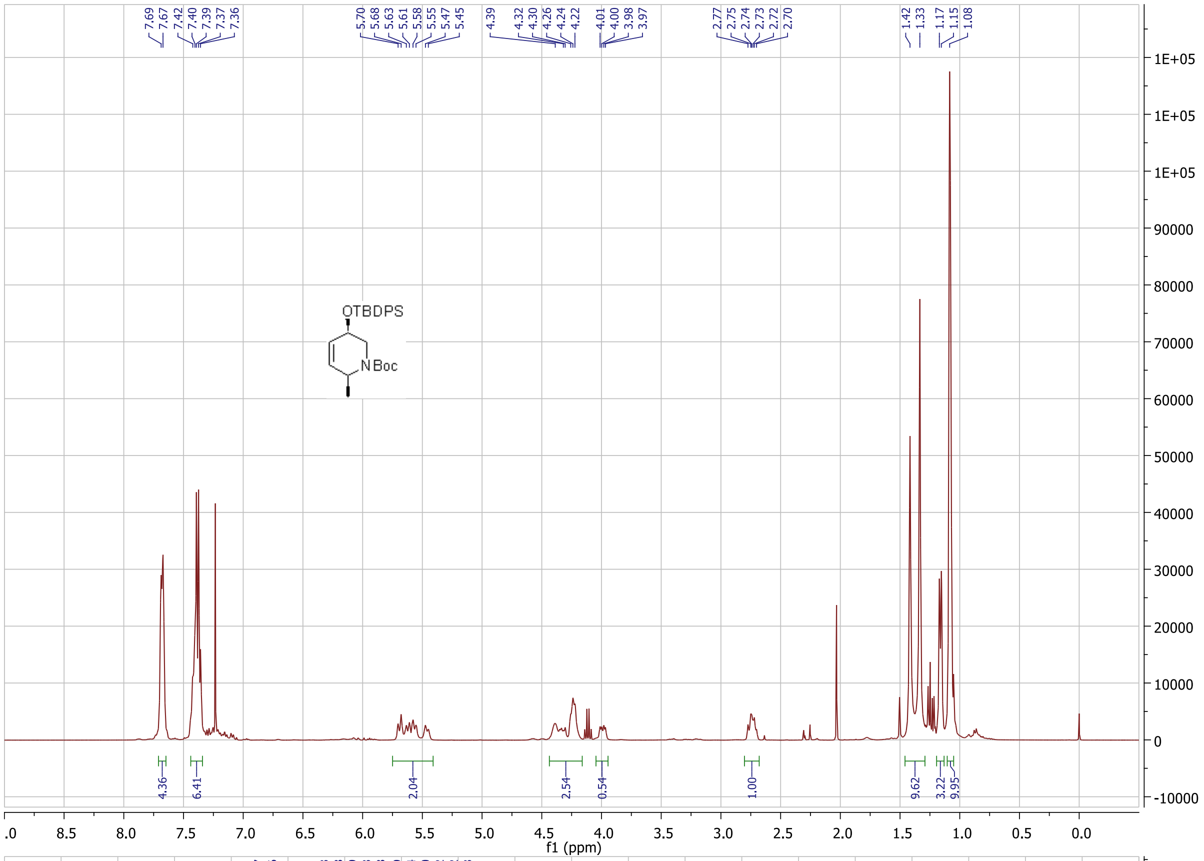

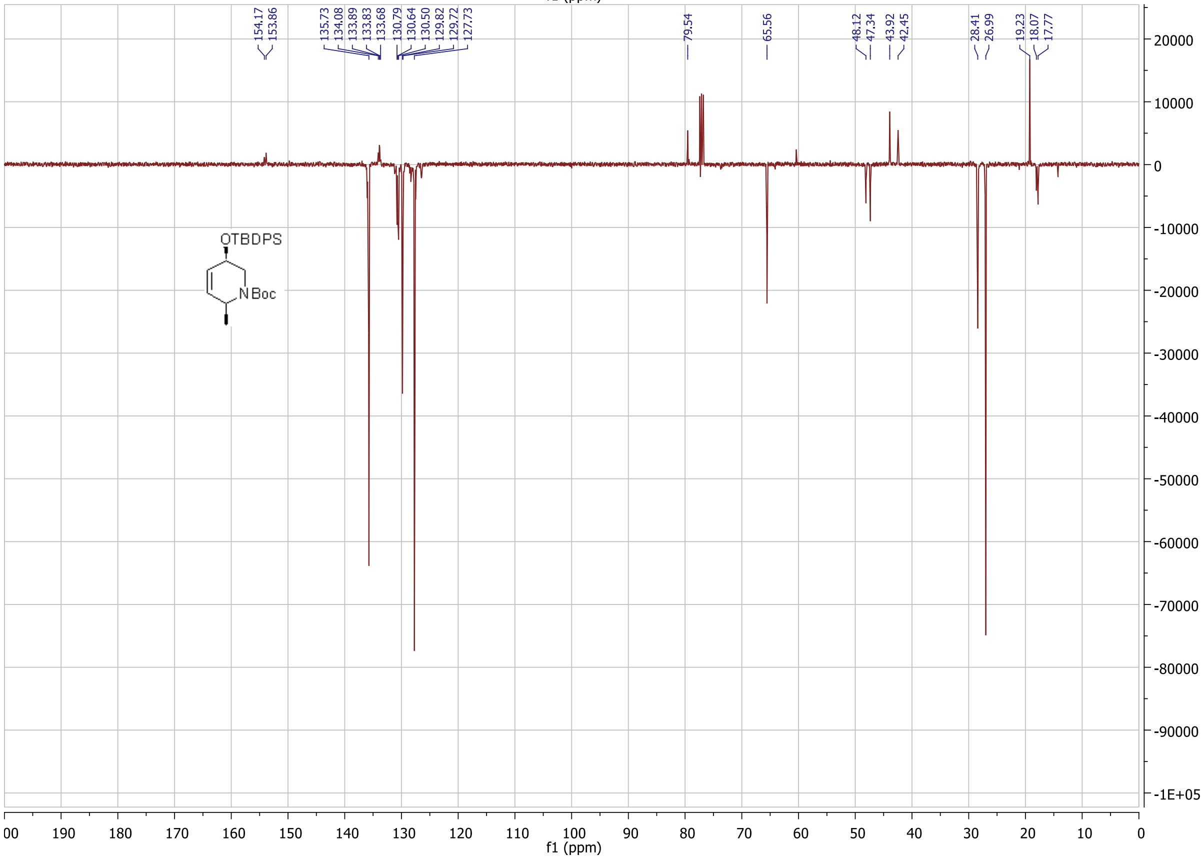

^{1}H and ^{13}C NMR ($CDCl_3$)

tert-Butyl (2*S*,3*S*,4*S*,5*S*)-5-((*tert*-butyldiphenylsilyl)oxy)-3,4-dihydroxy-2-methylpiperidine-1-carboxylate (**39**)

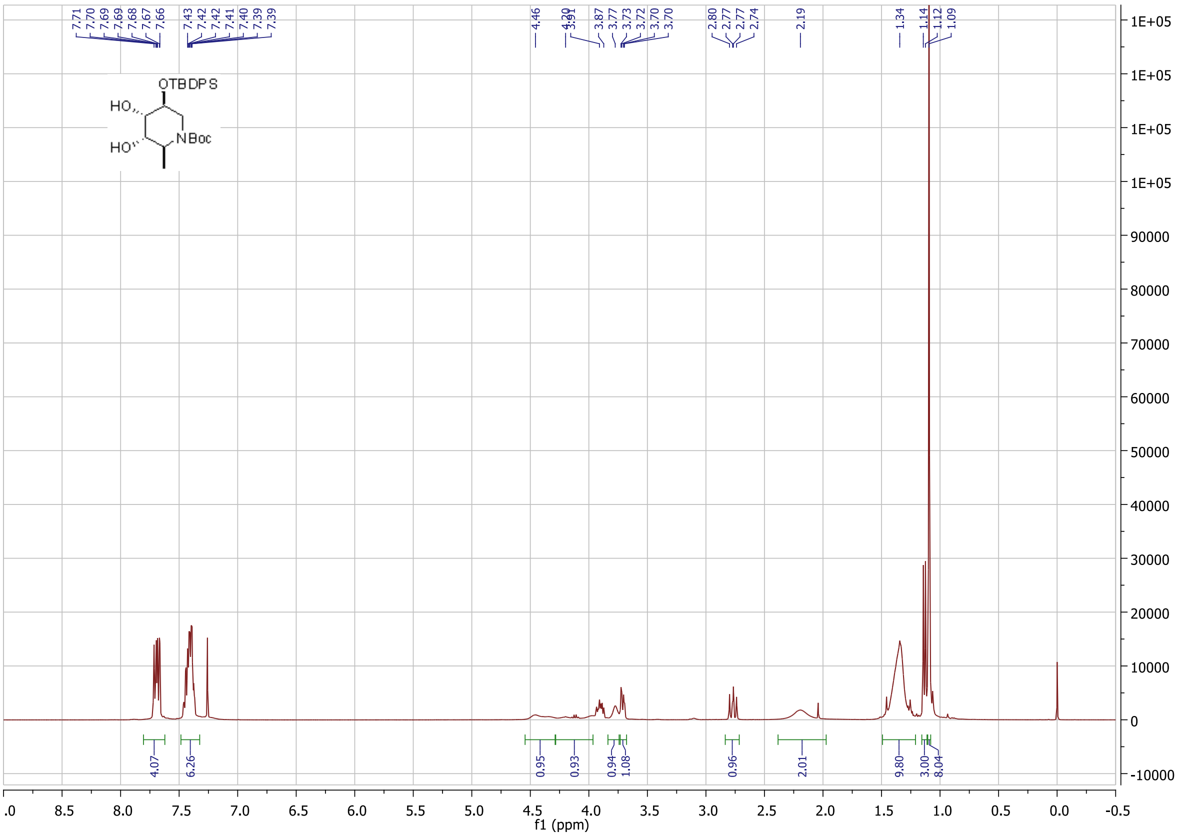

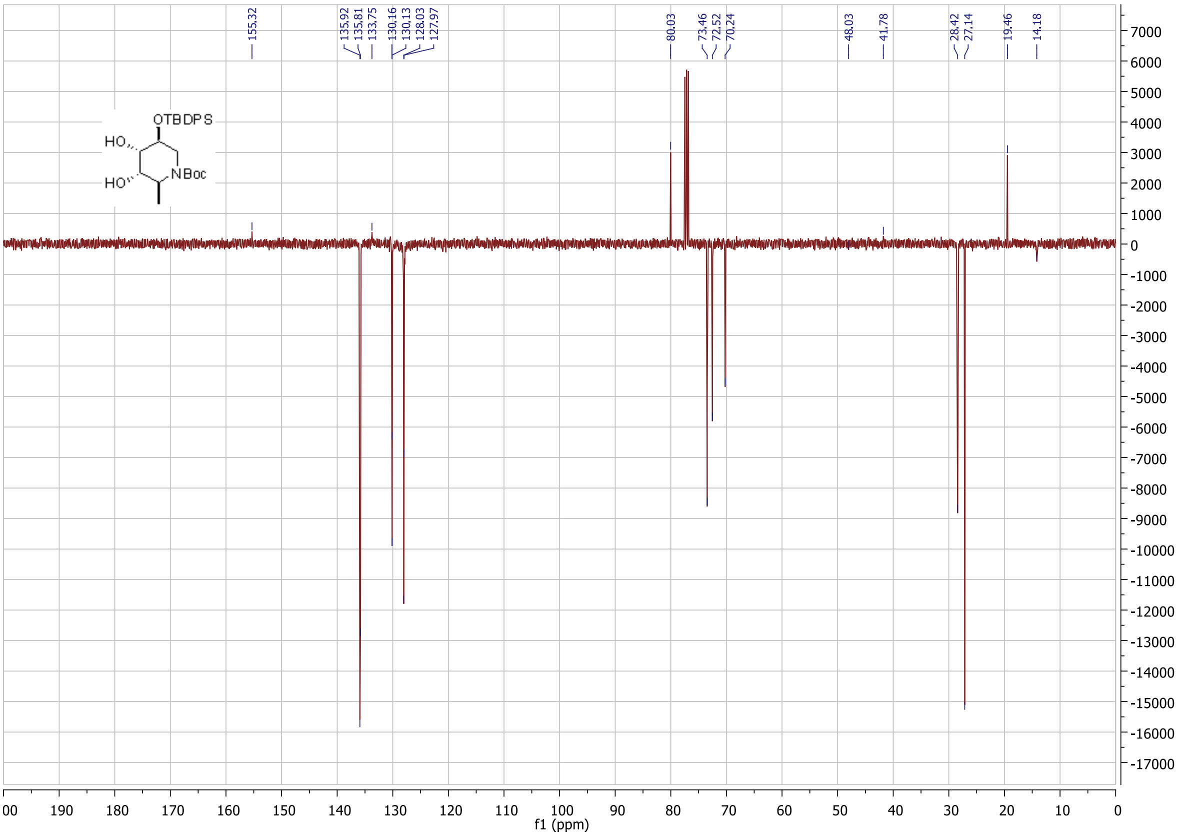

^{1}H and ^{13}C NMR (CD_3OD)

tert-Butyl (2*S*,3*S*,4*R*,5*S*)-3,4,5-trihydroxy-2-methylpiperidine-1-carboxylate

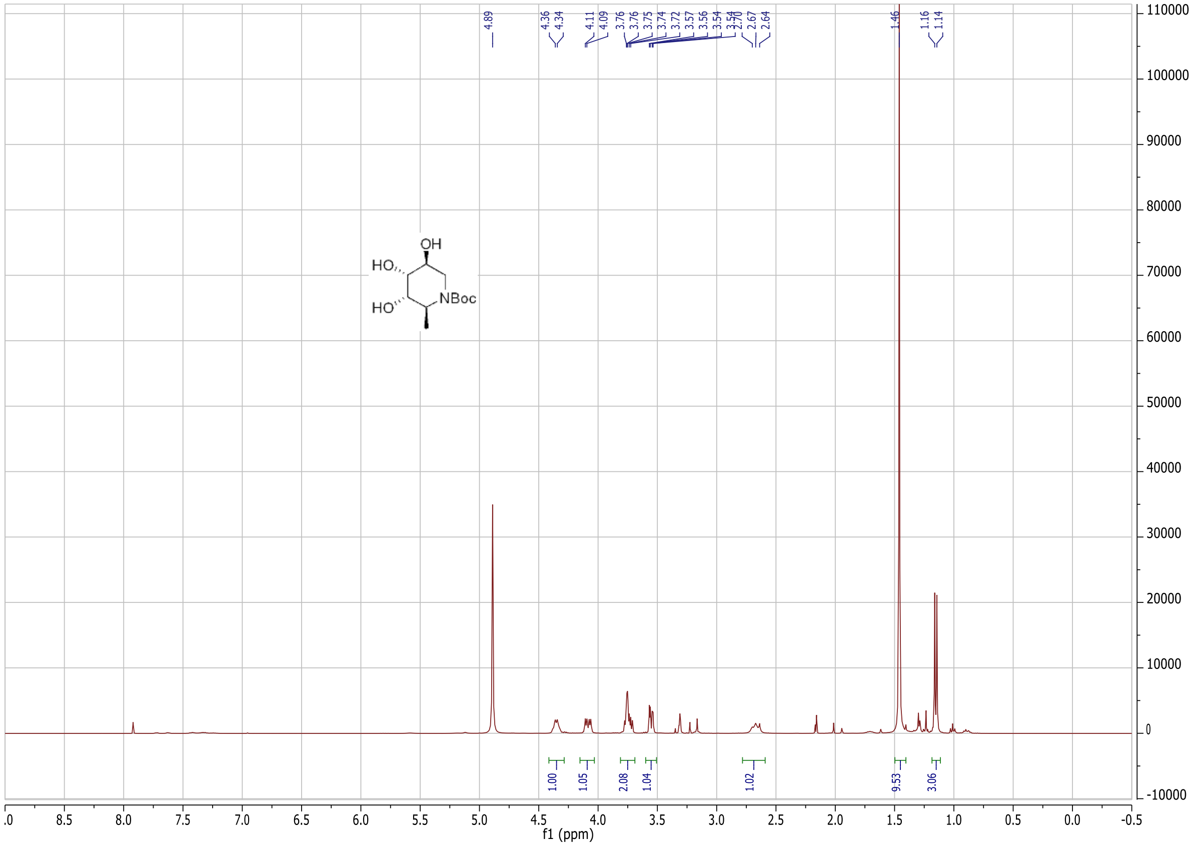

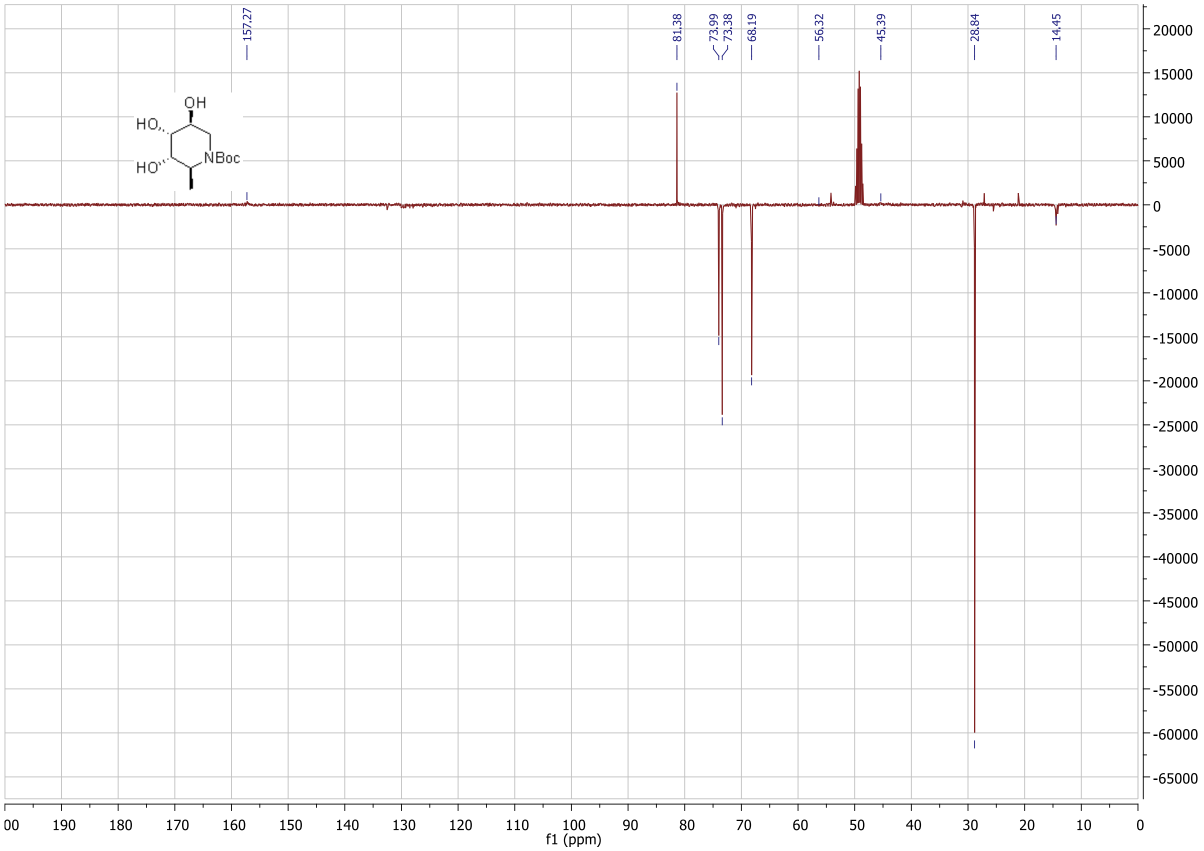

^{1}H and ^{13}C NMR (D_2O)

(2*S*,3*S*,4*R*,5*S*)-2-methylpiperidine-3,4,5-triol hydrochloride (**8.HCl**)

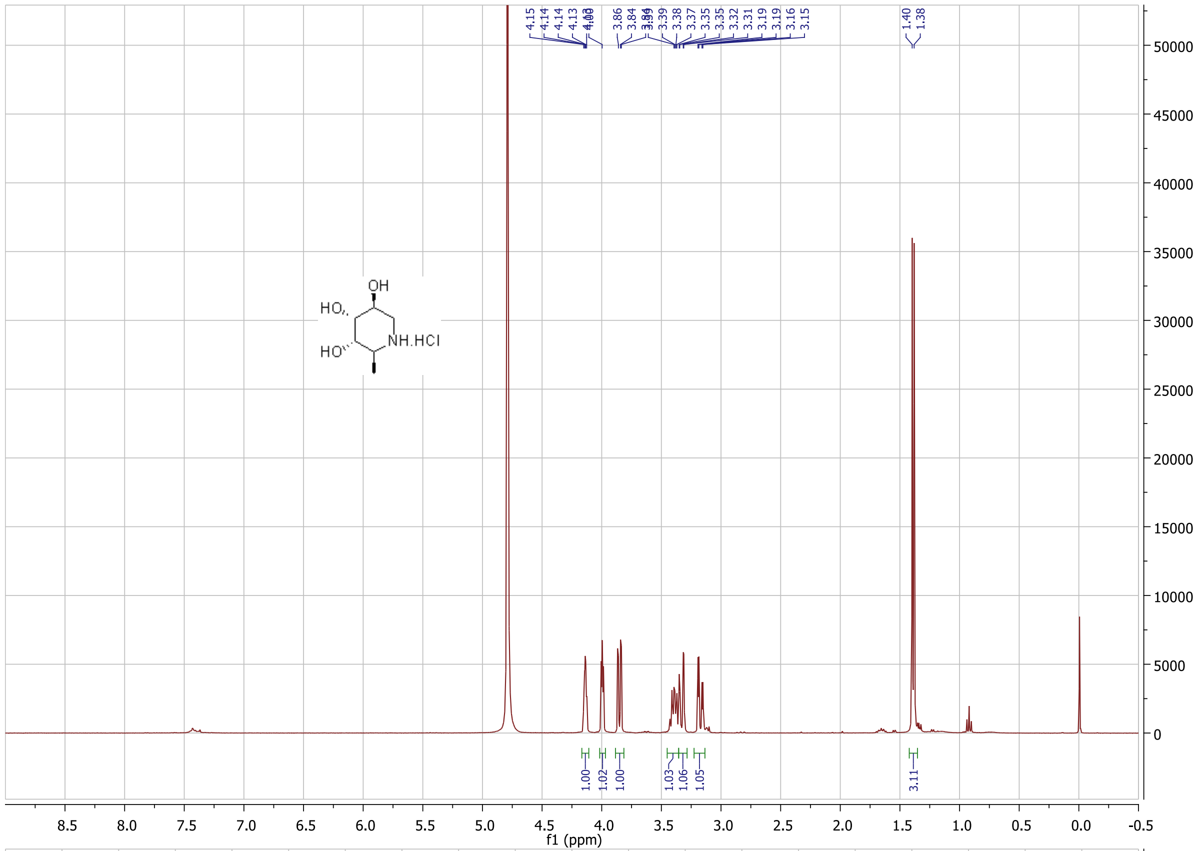

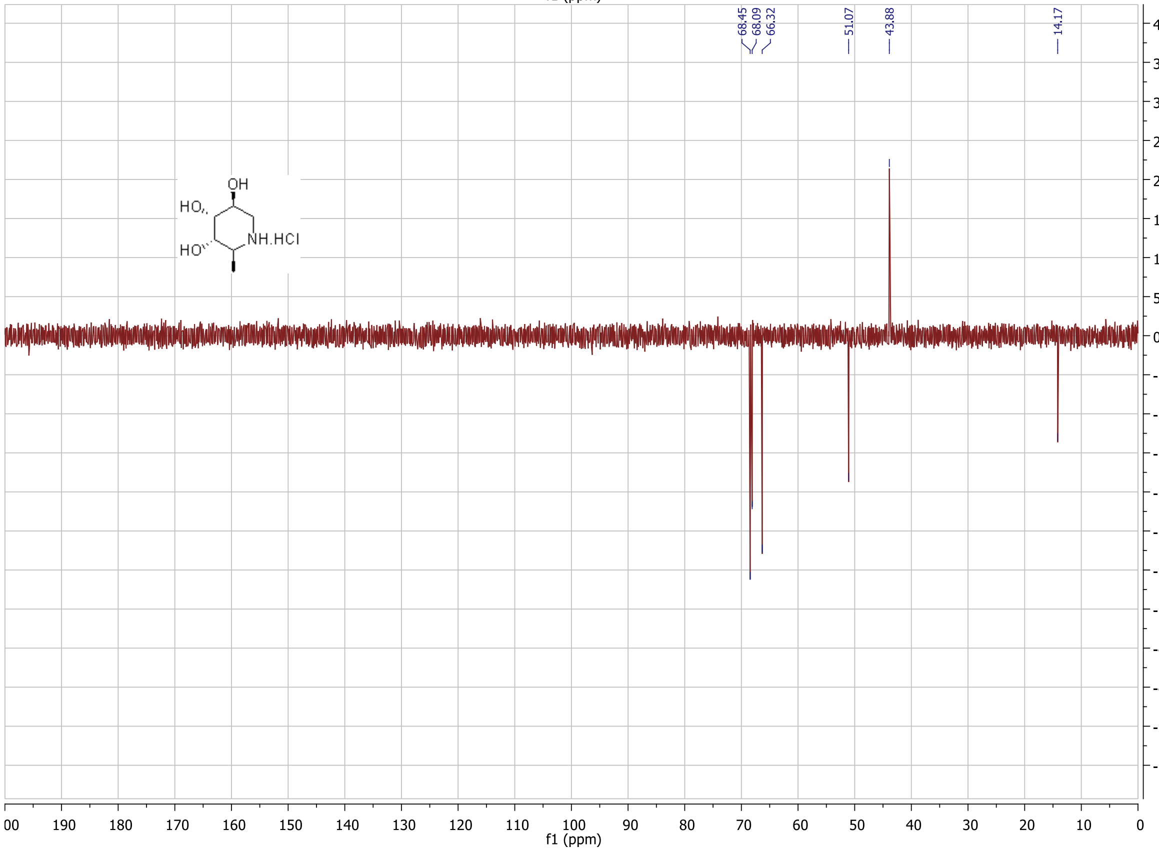

^{1}H and ^{13}C NMR ($CDCl_3$)

tert-Butyl (3a*S*,4*S*,7*S*,7a*R*)-7-hydroxy-2,2,4-trimethyltetrahydro-[1,3]dioxolo[4,5-c]pyridine-5(4*H*)-carboxylate:

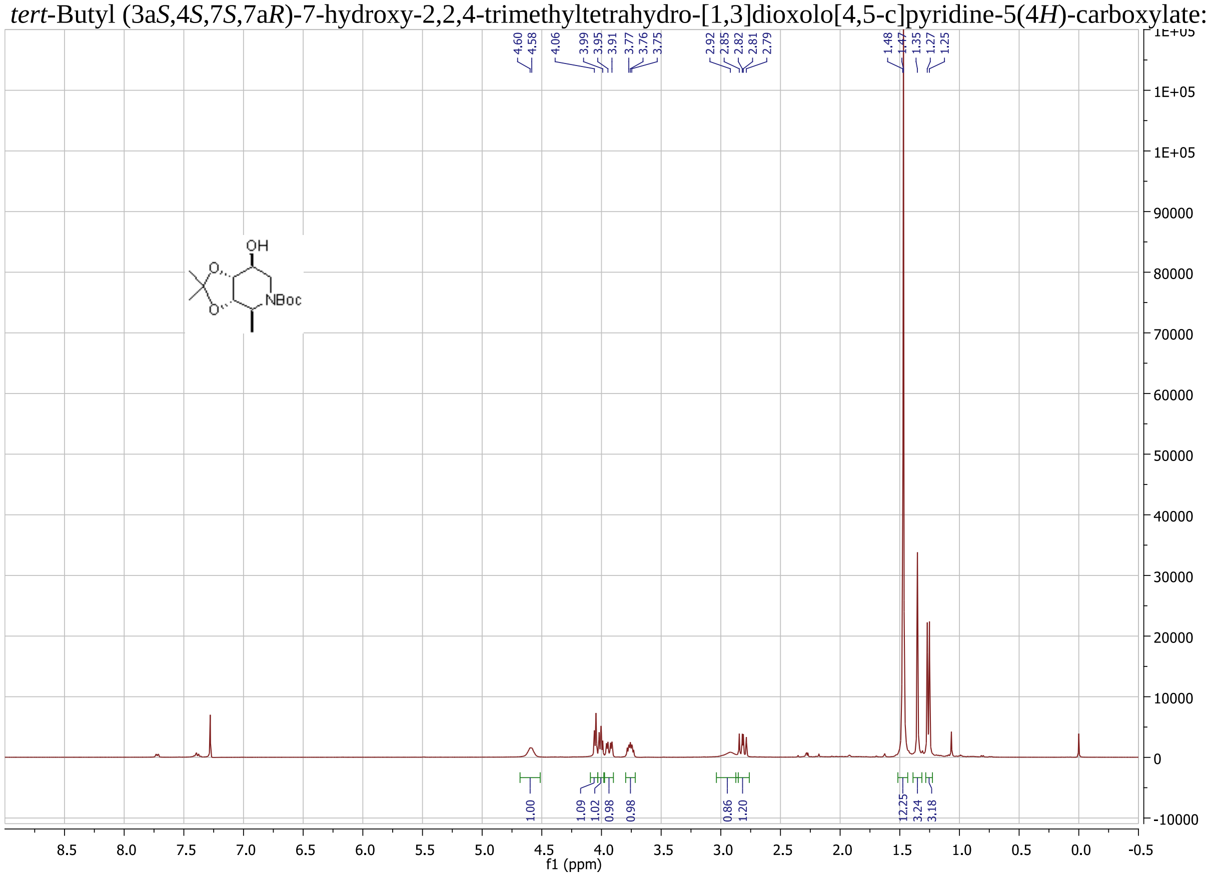

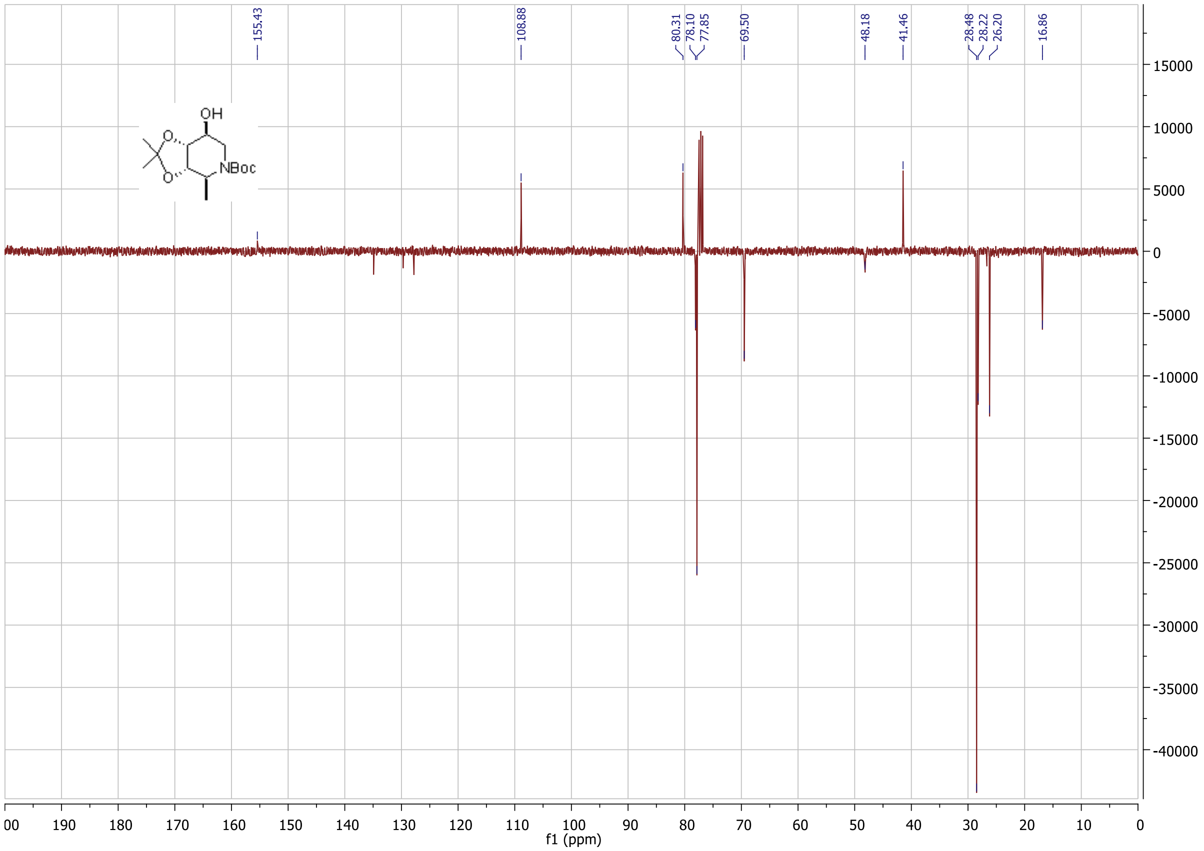

^{1}H and ^{13}C NMR ($CDCl_3$)

tert-Butyl (3a*S*,4*S*,7a*S*)-2,2,4-trimethyl-7-oxotetrahydro-[1,3]dioxolo[4,5-c]pyridine-5(4*H*)-carboxylate

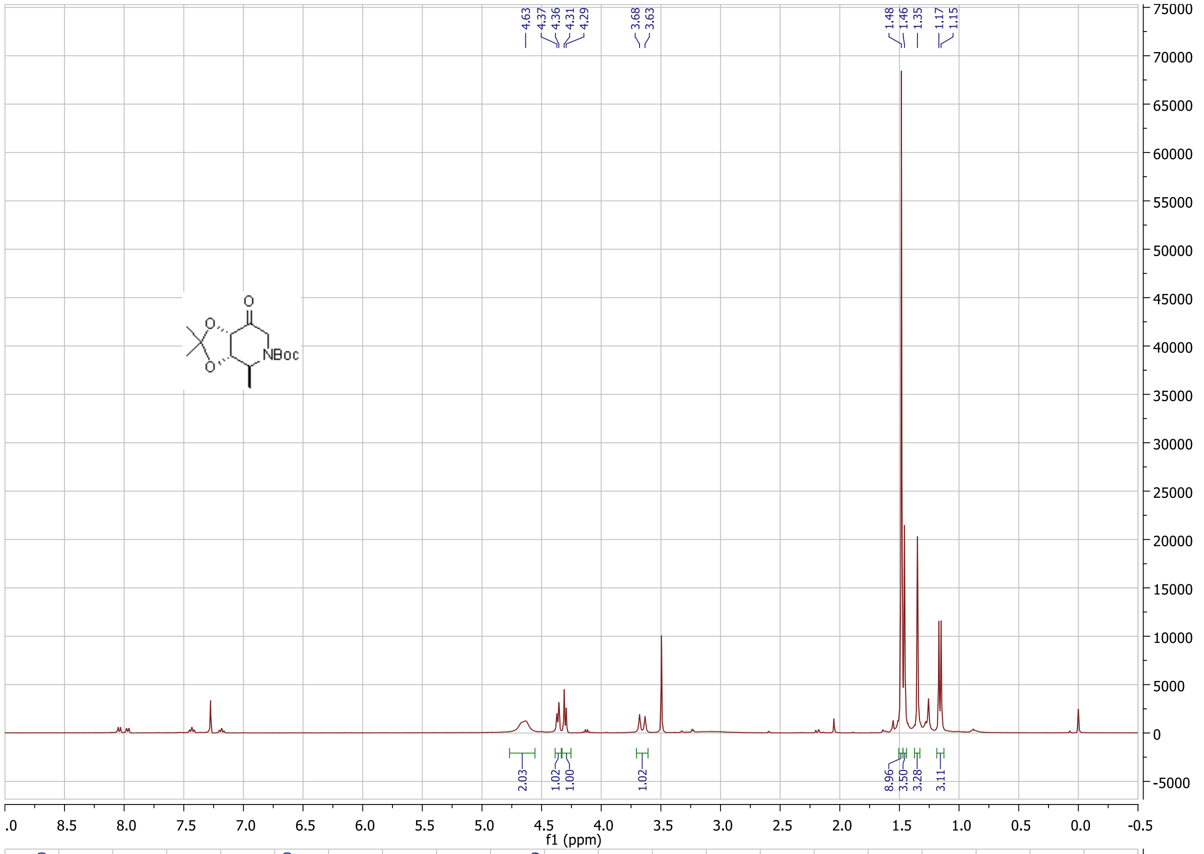

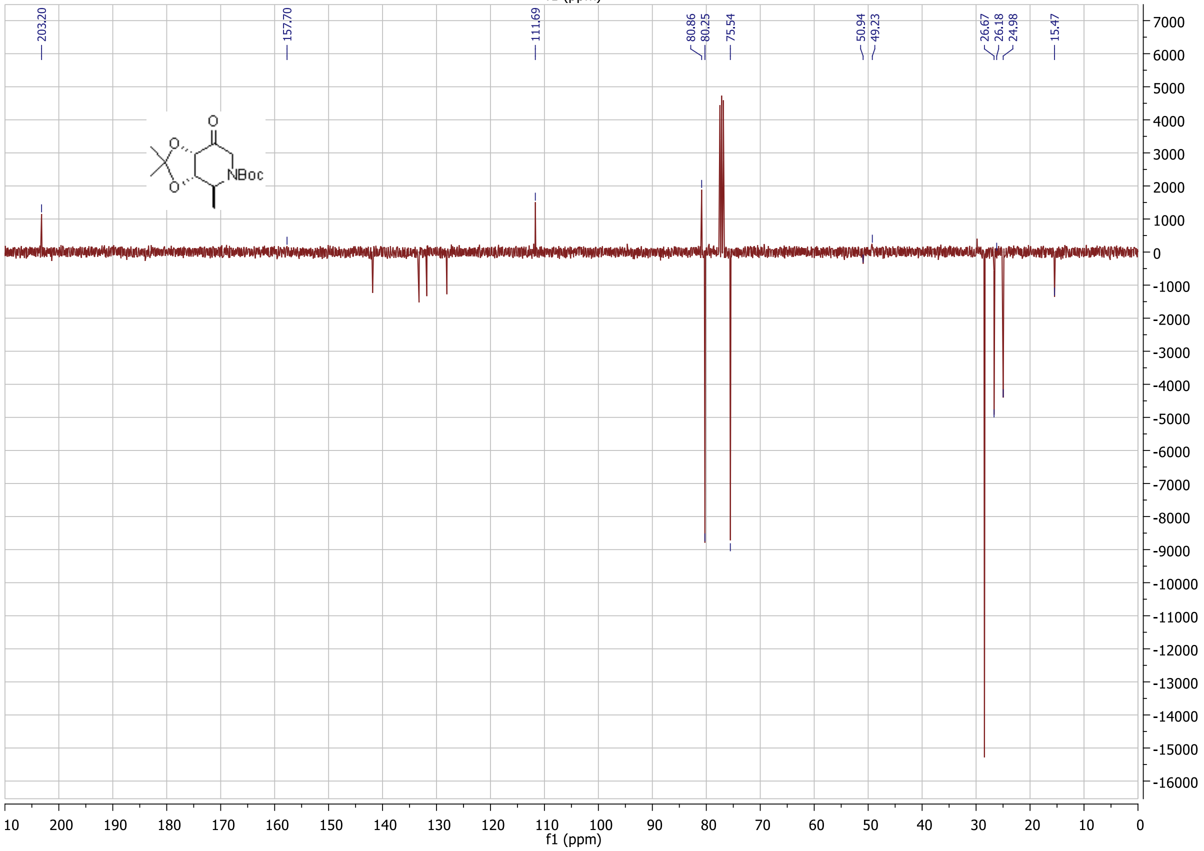

^{1}H and ^{13}C NMR ($CDCl_3$) compound (**40**)

tert-Butyl (3a*S*,4*S*,7*R*,7a*R*)-7-hydroxy-2,2,4-trimethyltetrahydro-[1,3]dioxolo[4,5-c]pyridine-5(4*H*)-carboxylate

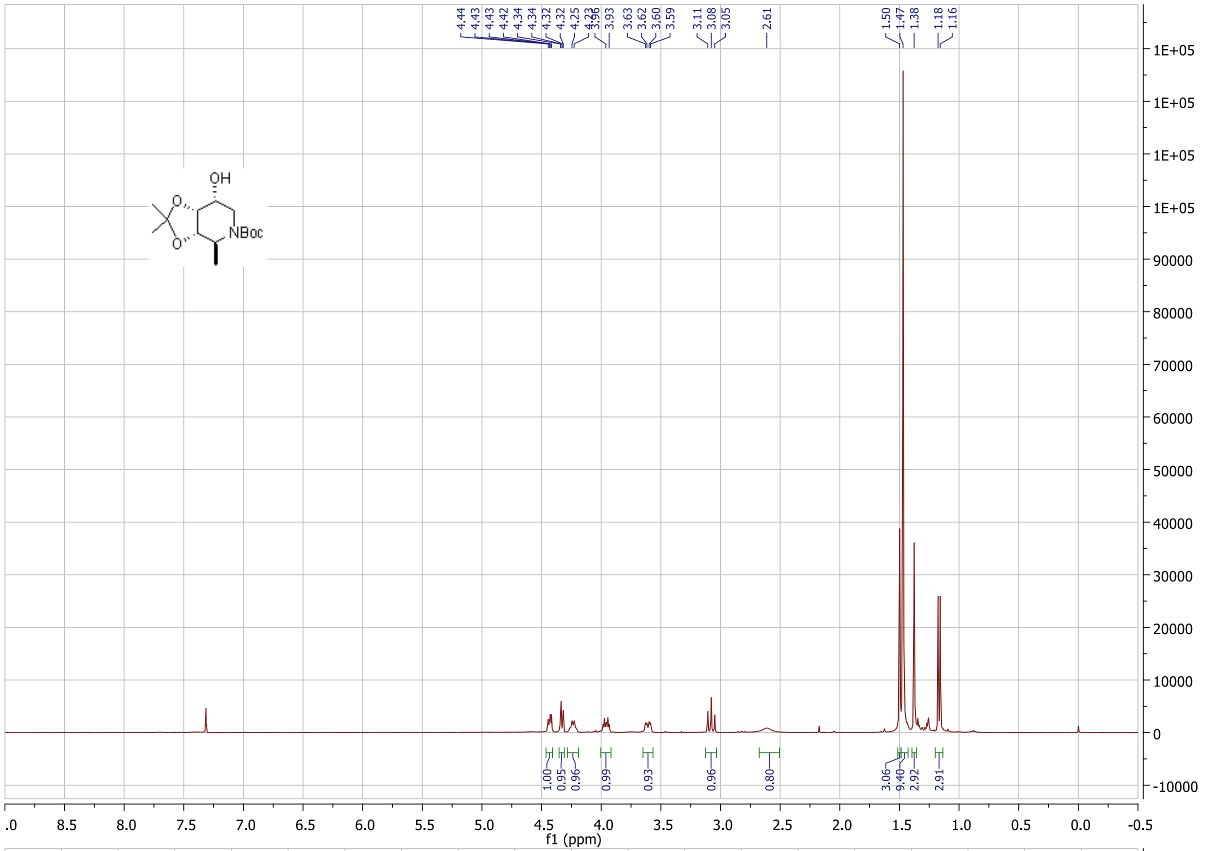

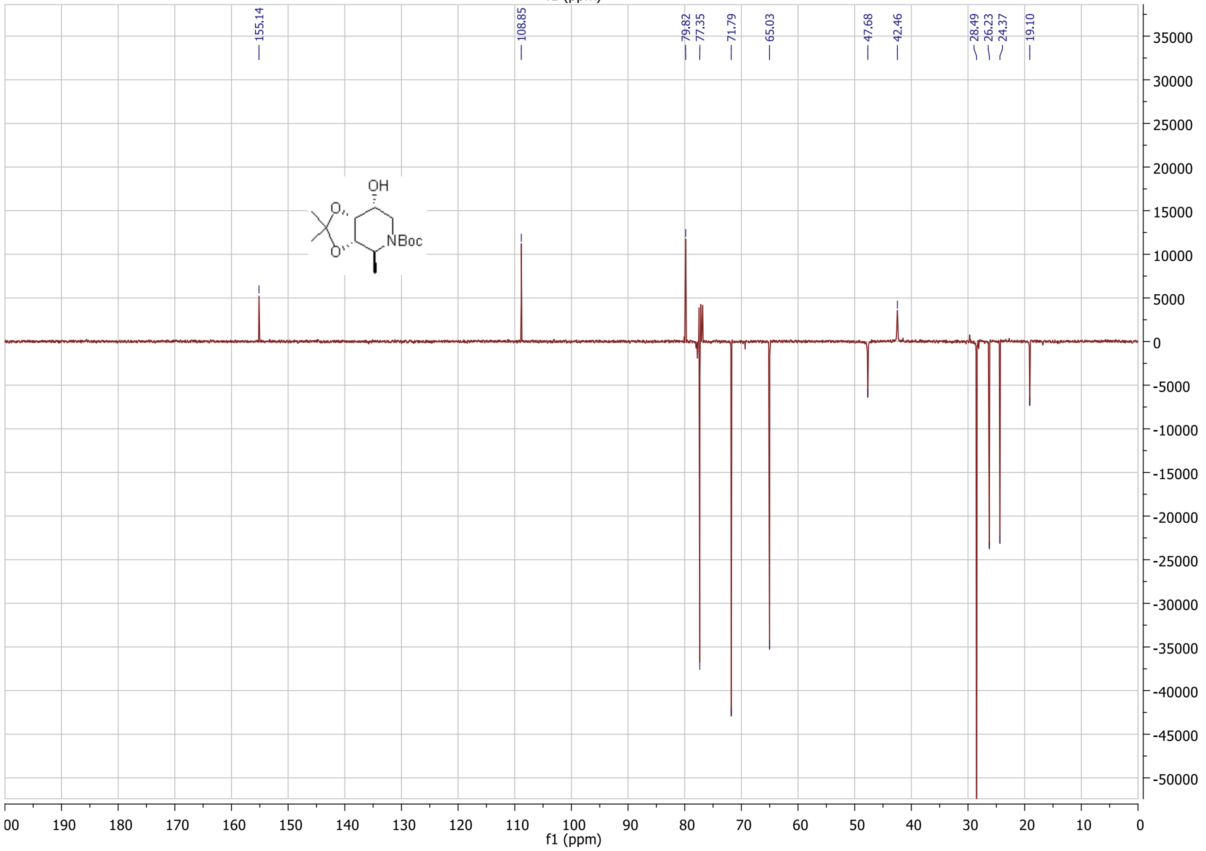

^{1}H and ^{13}C NMR (D_2O)

(2*S*,3*S*,4*R*,5*R*)-2-methylpiperidine-3,4,5-triol hydrochloride (**9.HCl**)

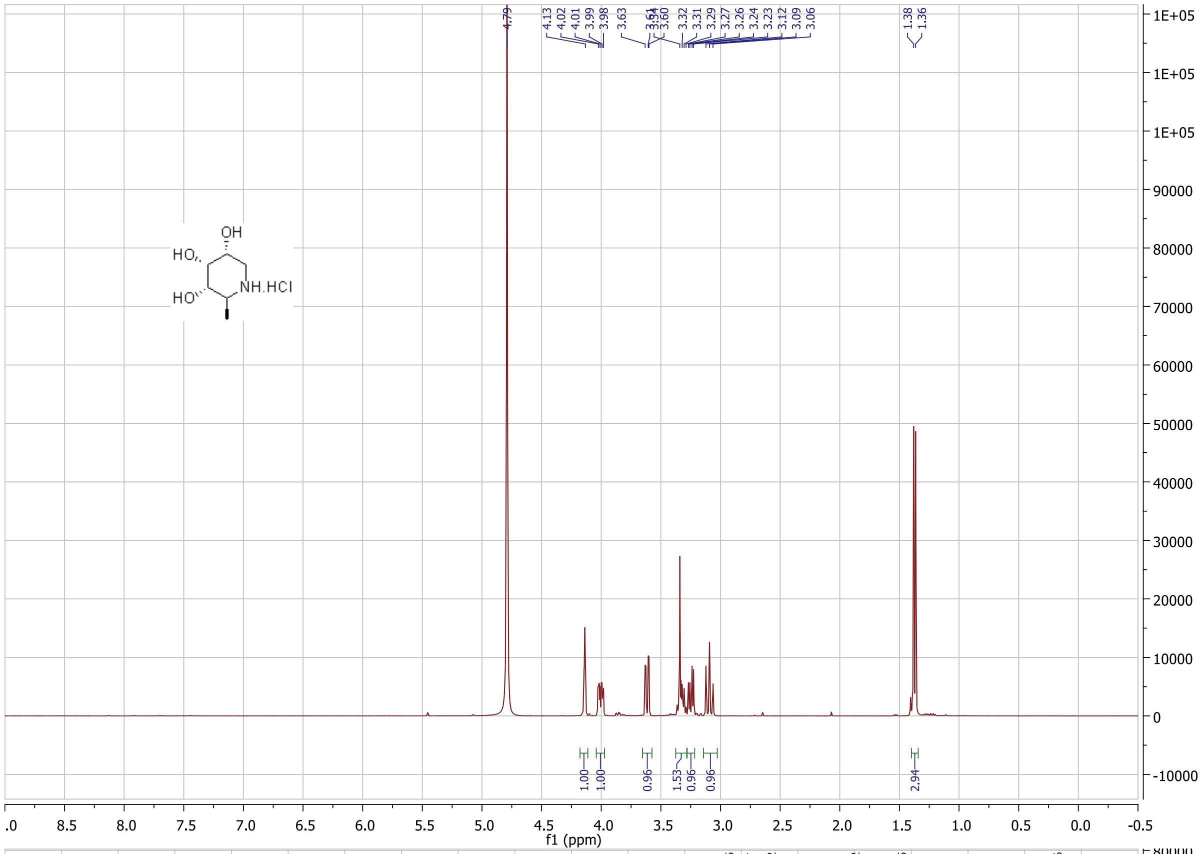

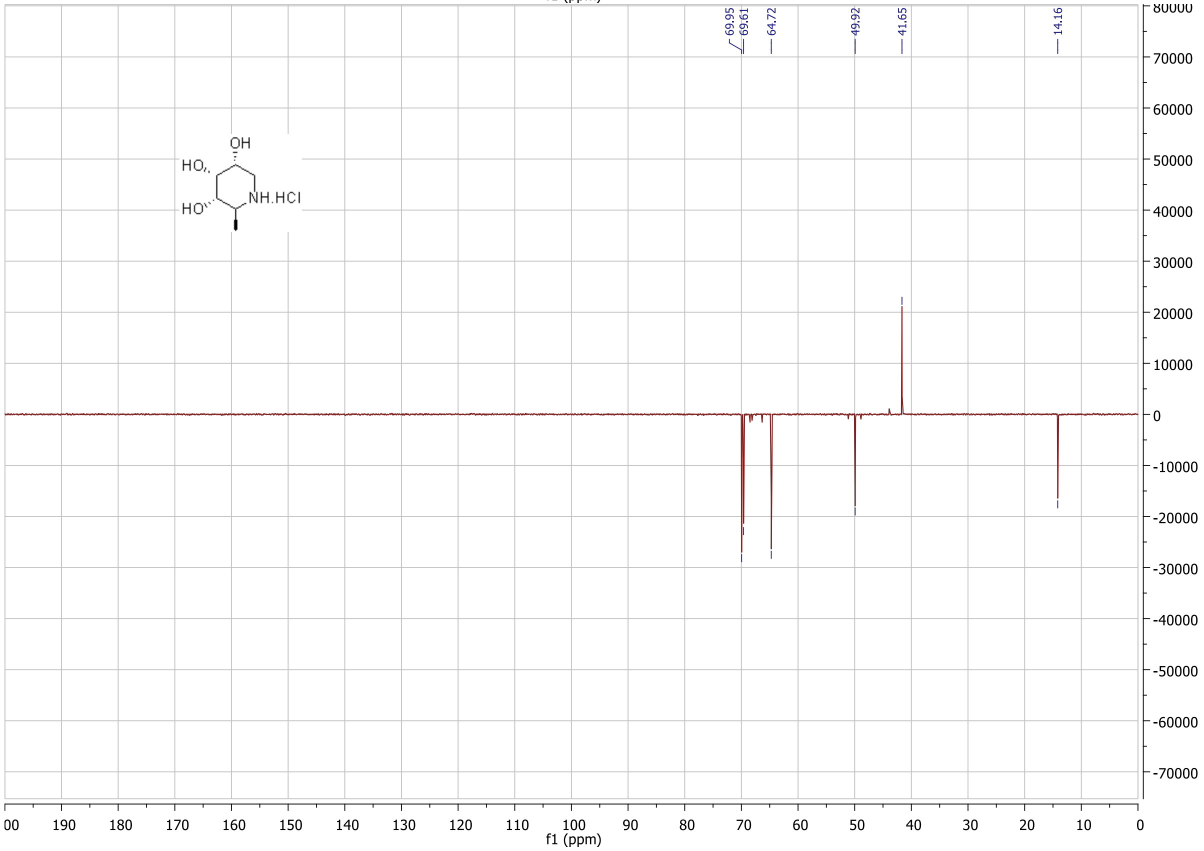

^{1}H and ^{13}C NMR ($CDCl_3$)

(*R*,*E*)-*N*-((*R*)-But-3-en-2-yl)-2-((*tert*-butyldiphenylsilyl)oxy)-4-phenylbut-3-en-1-amine (**41**)

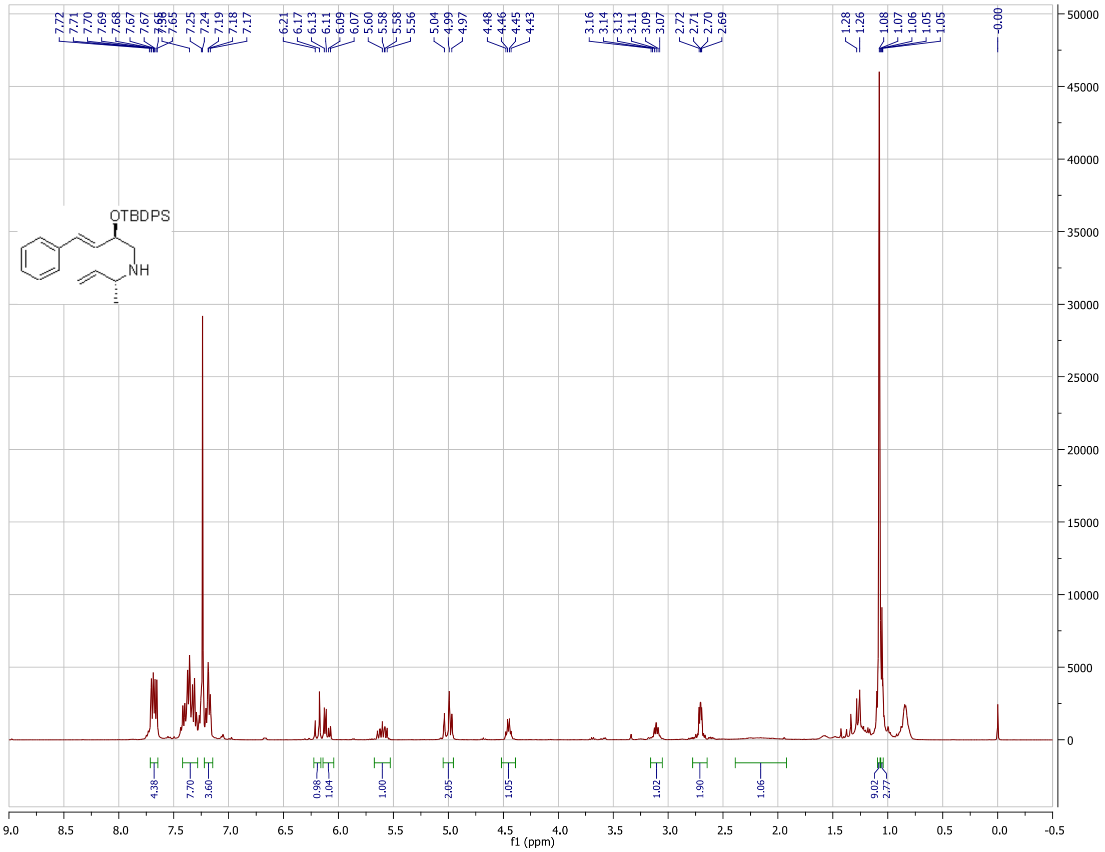

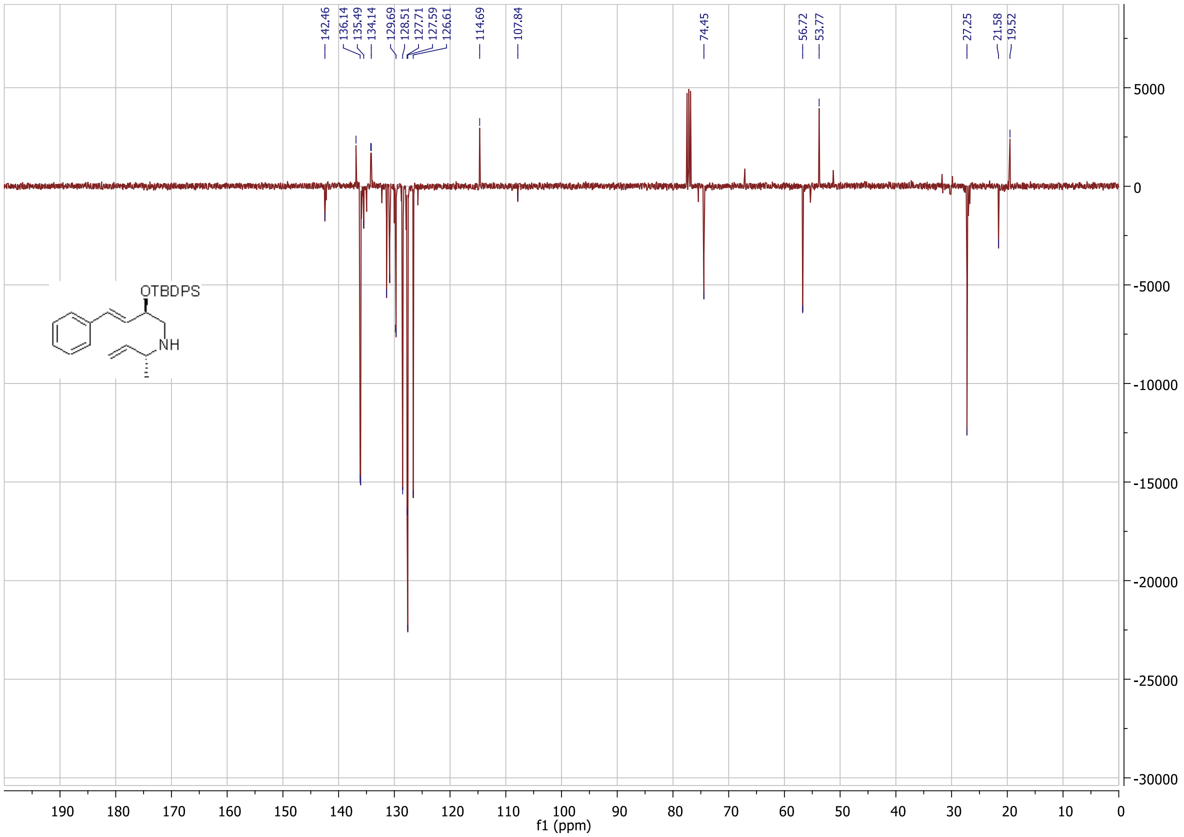

^{1}H and ^{13}C NMR ($CDCl_3$)

tert-Butyl ((*R*)-but-3-en-2-yl)((*R*,*E*)-2-((*tert*-butyldiphenylsilyl)oxy)-4-phenylbut-3-en-1-yl)carbamate

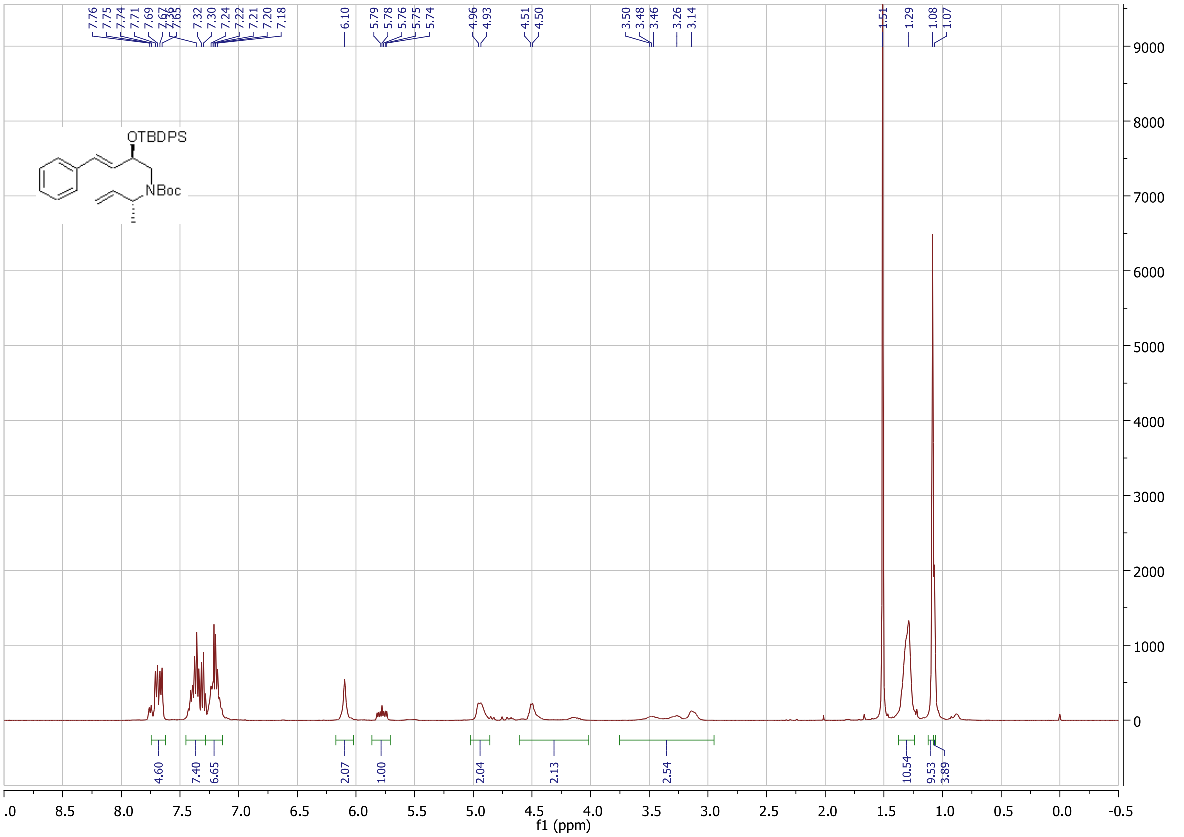

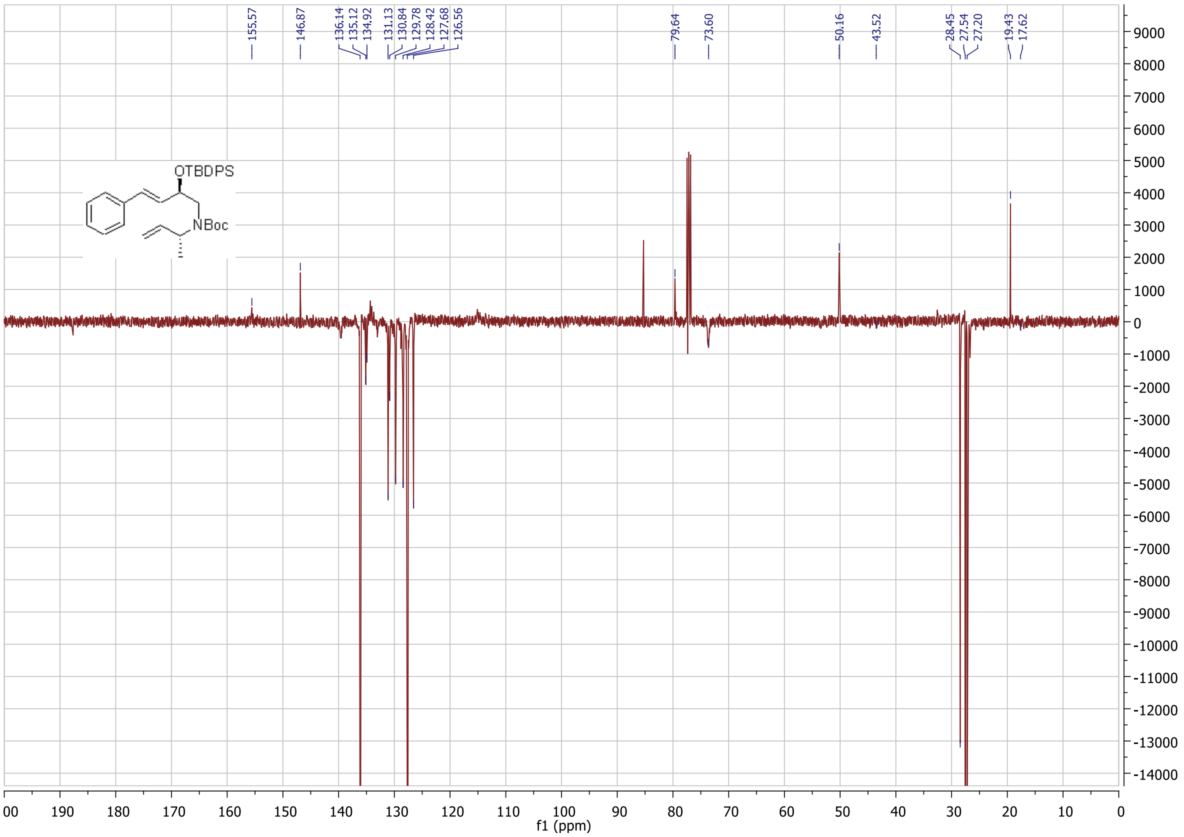

^1H and ^{13}C NMR ($CDCl_3$)

tert-Butyl (3*R*,6*R*)-3-((*tert*-butyldiphenylsilyl)oxy)-6-methyl-3,6-dihydropyridine-1(2*H*)-carboxylate (**42**)

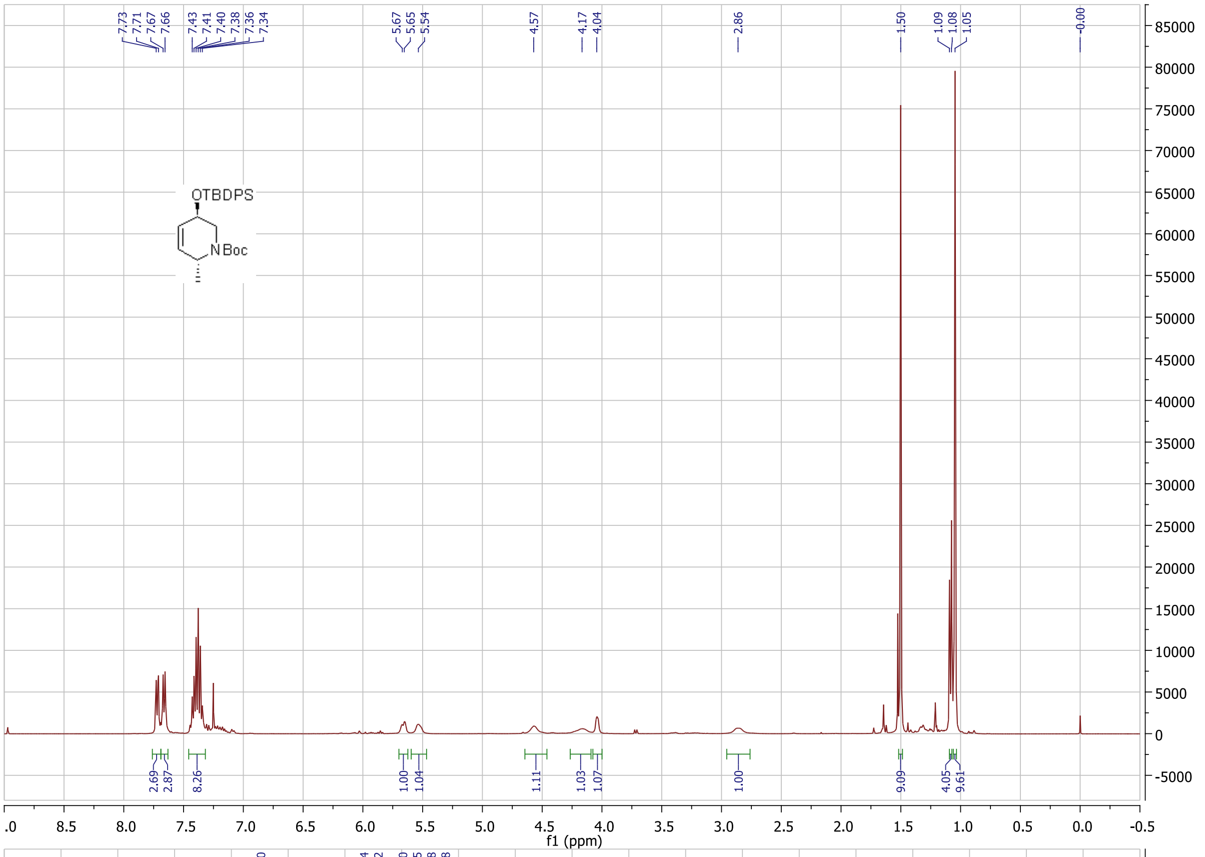

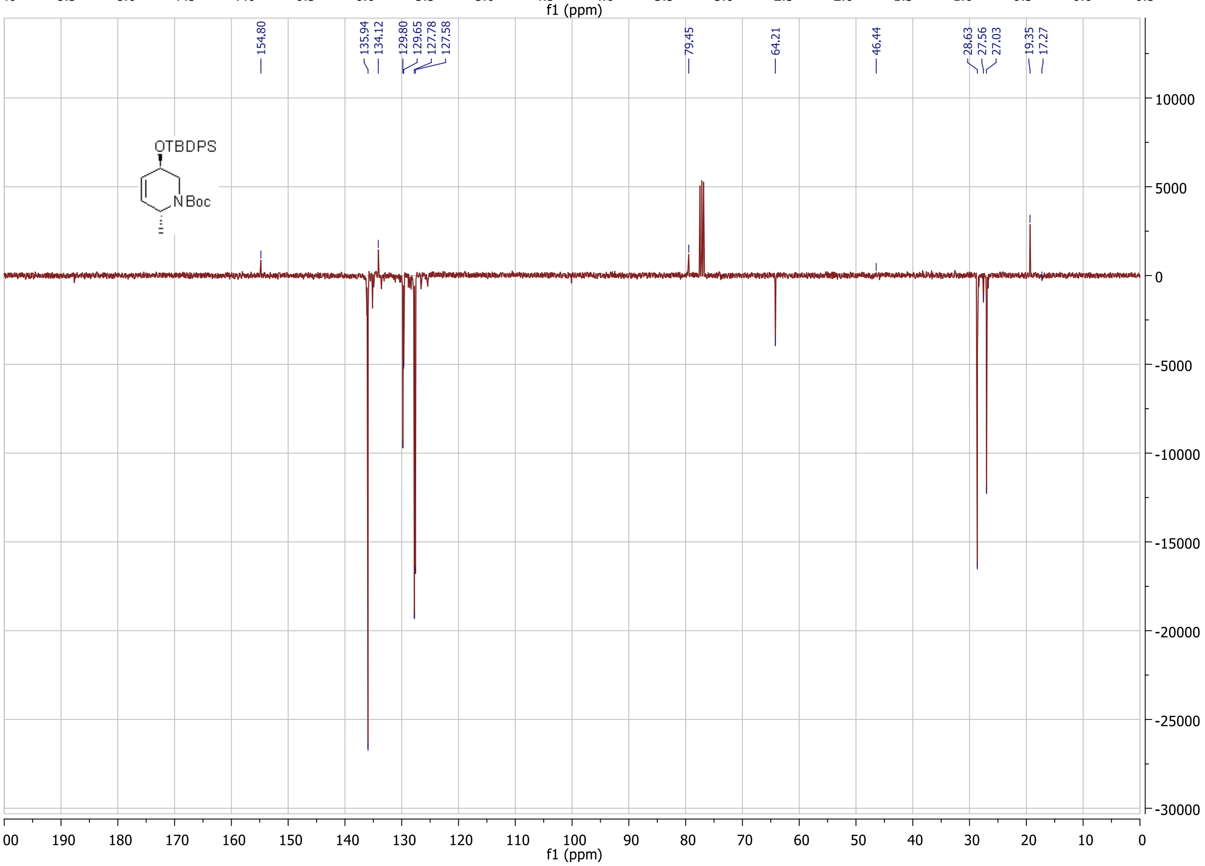

^{1}H and ^{13}C NMR ($CDCl_3$): compound **44**

(2*R*,3*S*,4*S*,5*S*)-1-(*tert*-butoxycarbonyl)-5-((*tert*-butyldiphenylsilyl)oxy)-2-methylpiperidine-3,4-diyldiacetate

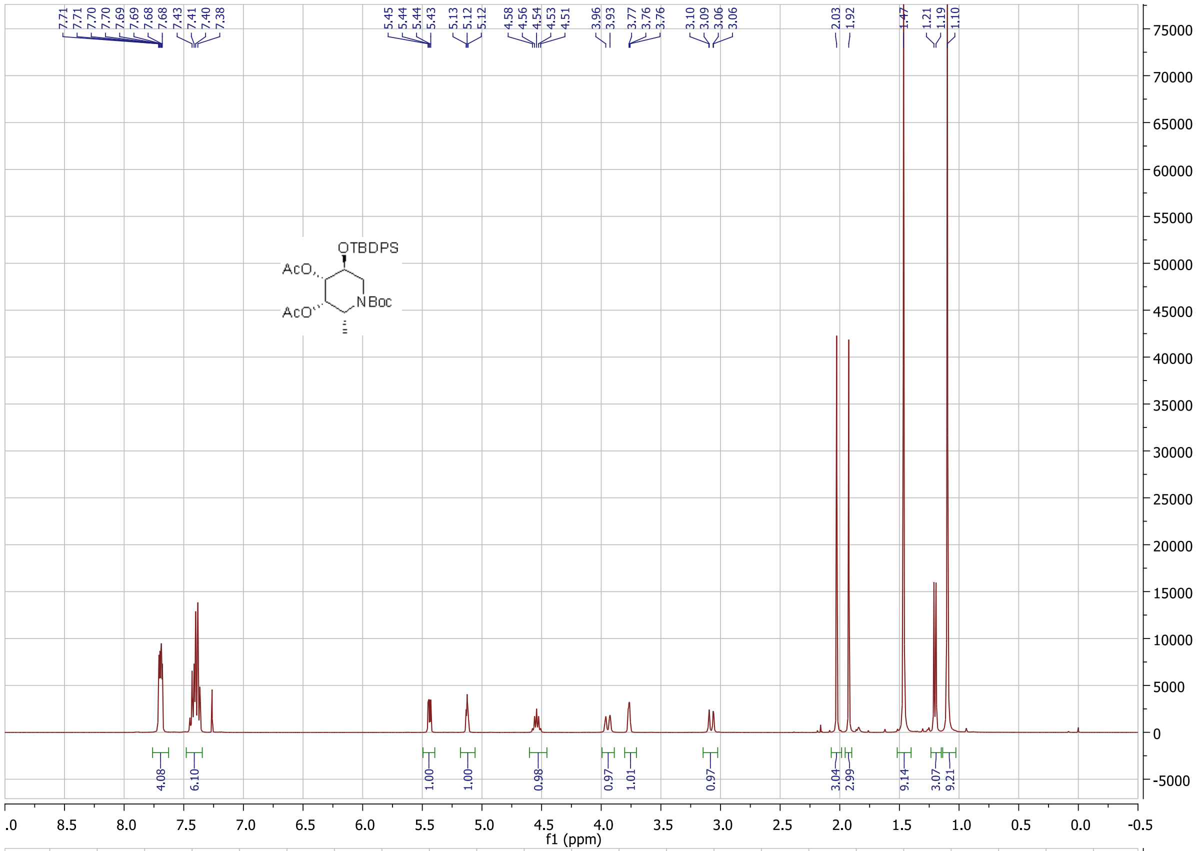

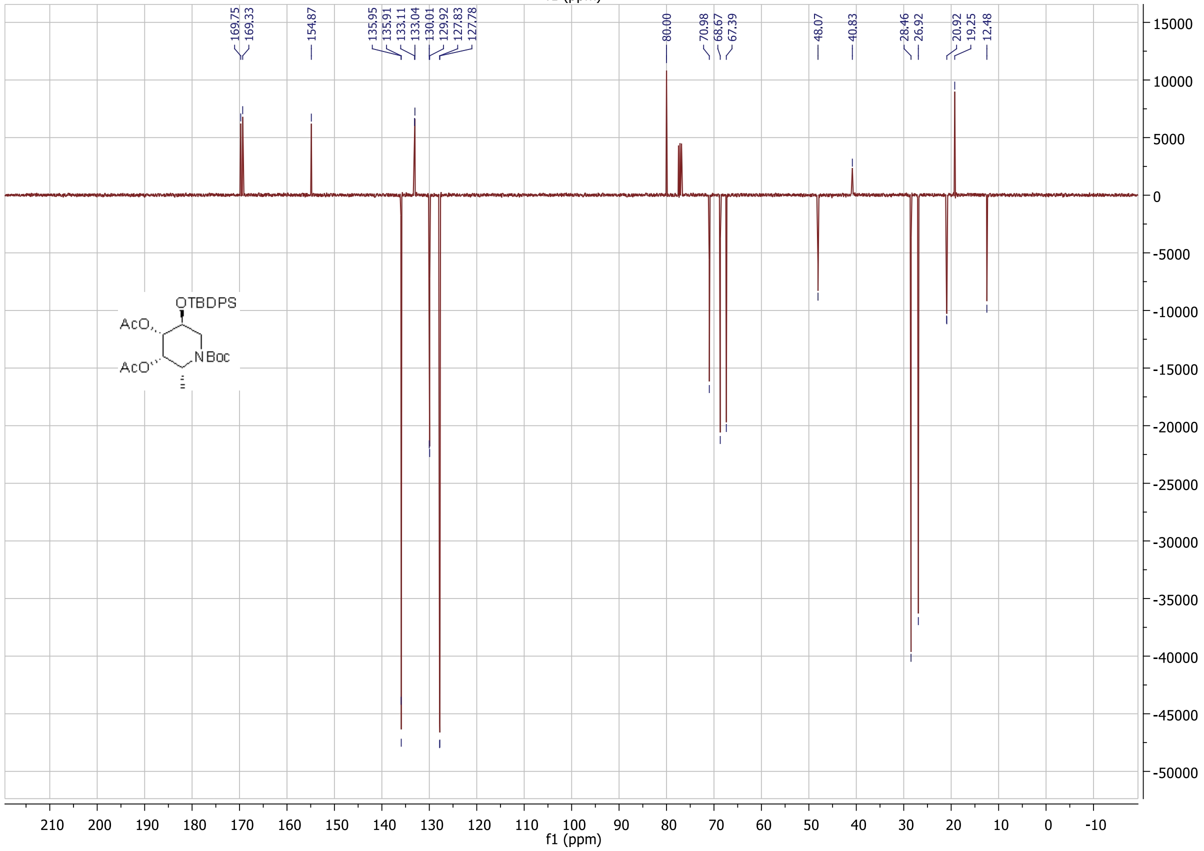

^{1}H and ^{13}C NMR (CDCl$_3$)

*t*ert-Butyl (2*R*,3*S*,4*S*,5*S*)-5-((*tert*-butyldiphenylsilyl)oxy)-3,4-dihydroxy-2-methylpiperidine-1-carboxylate

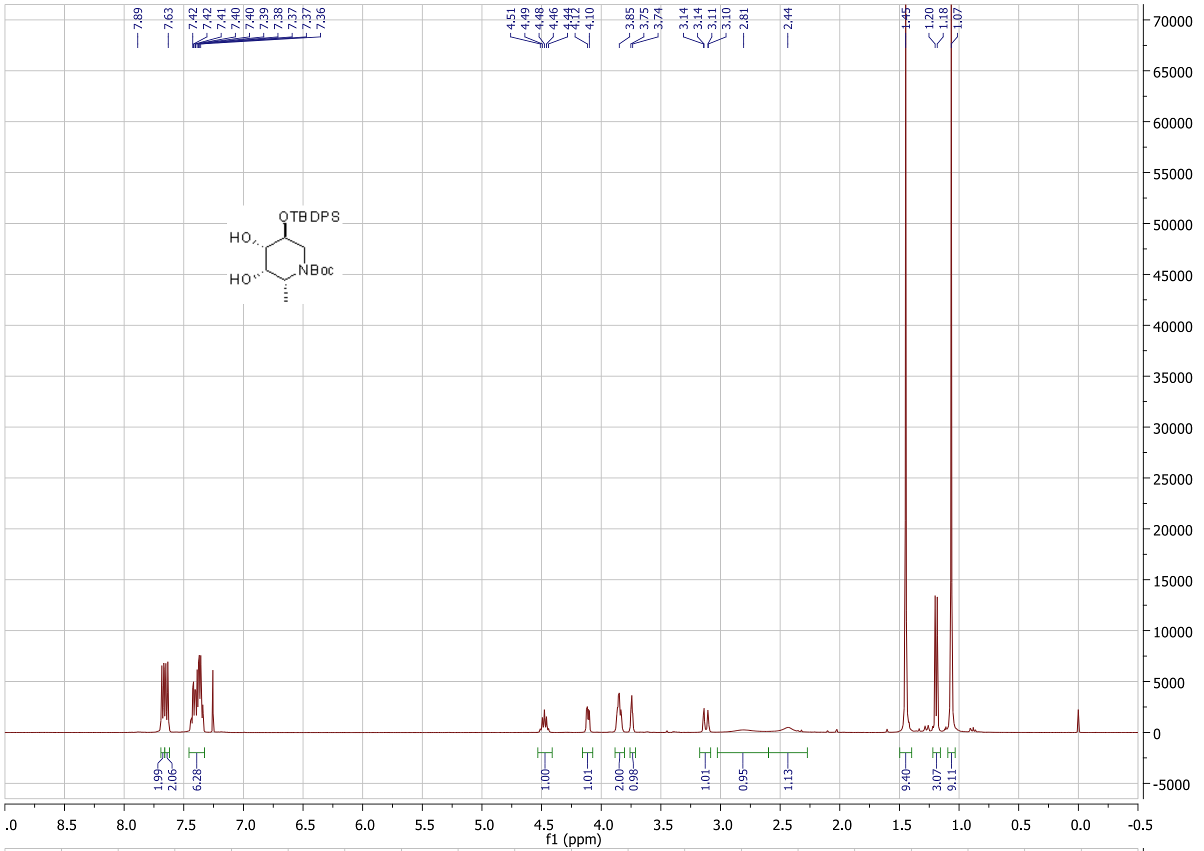

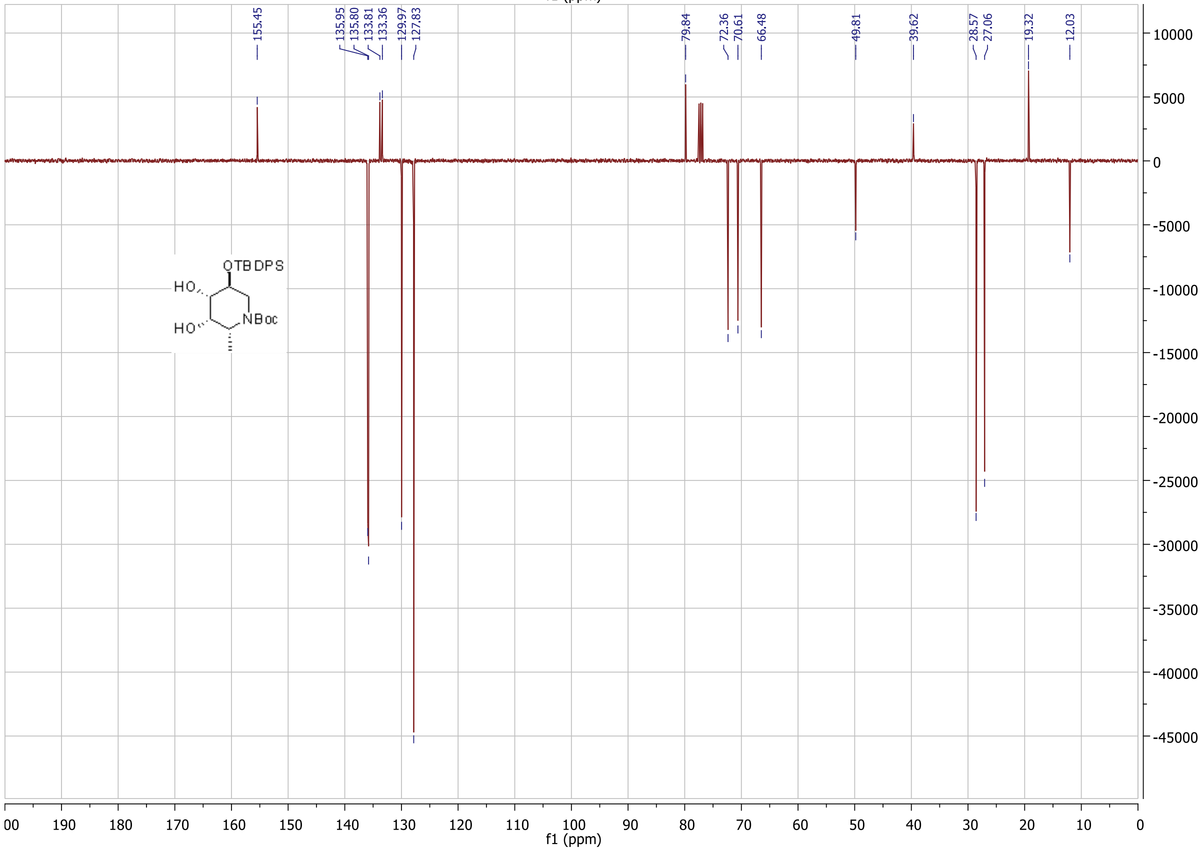

^{1}H and ^{13}C NMR (CD_3OD)

tert-Butyl (2*S*,3*R*,4*S*,5*R*)-3,4,5-trihydroxy-2-methylpiperidine-1-carboxylate

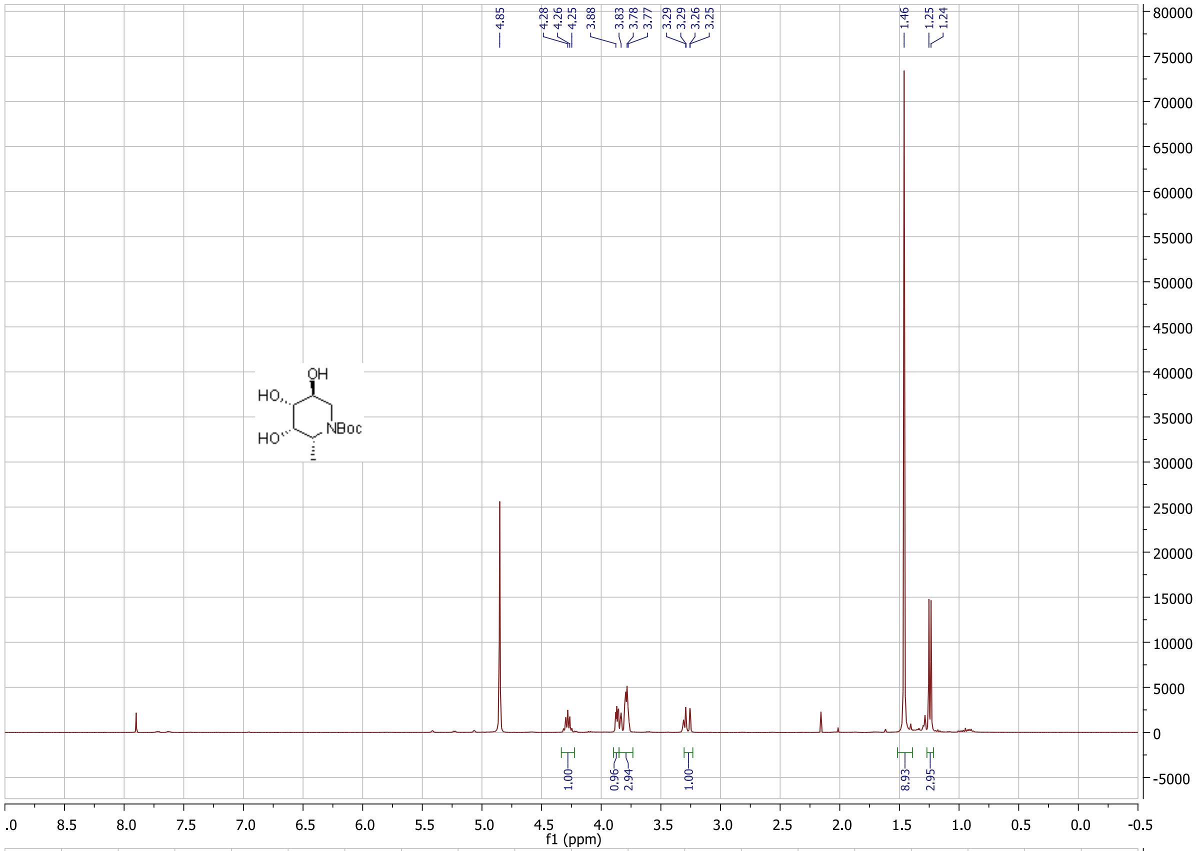

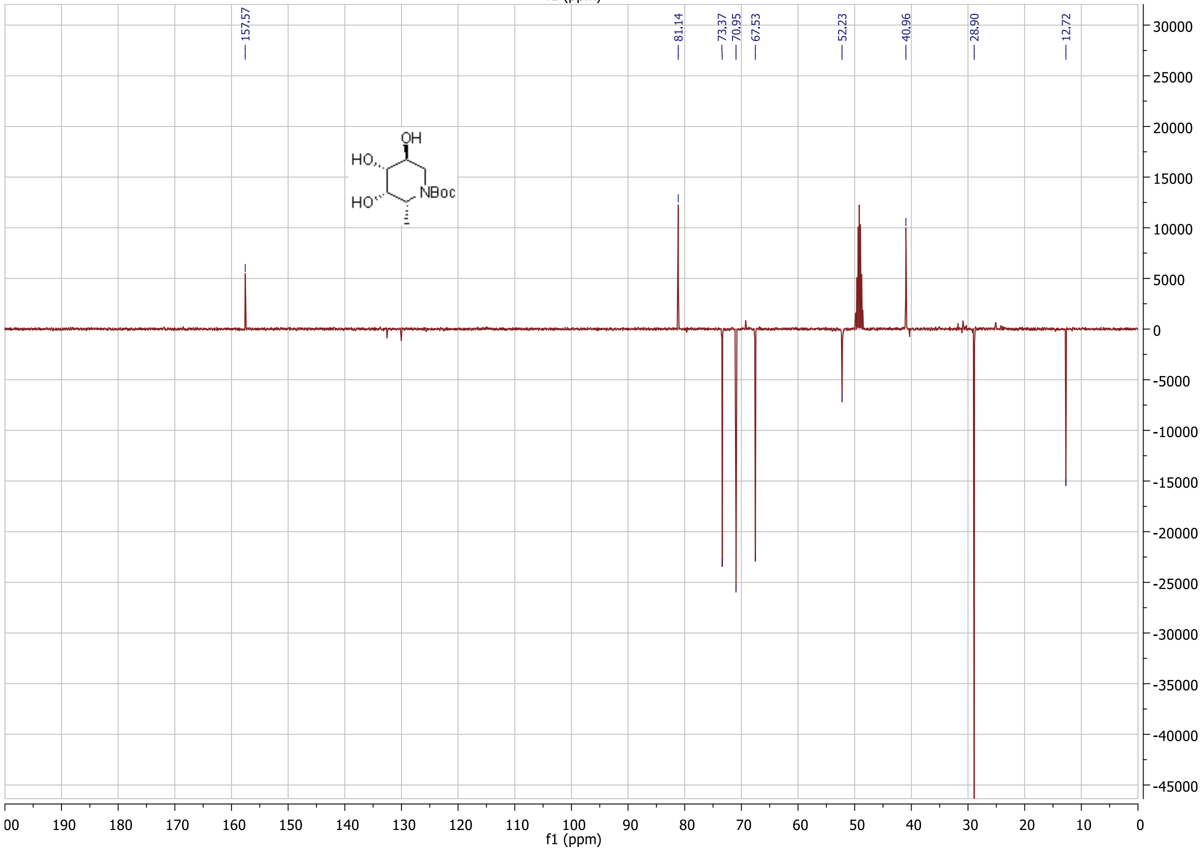

^{1}H and ^{13}C NMR (D_2O)

(2*R*,3*S*,4*R*,5*S*)-2-methylpiperidine-3,4,5-triol hydrochloride **(10)**

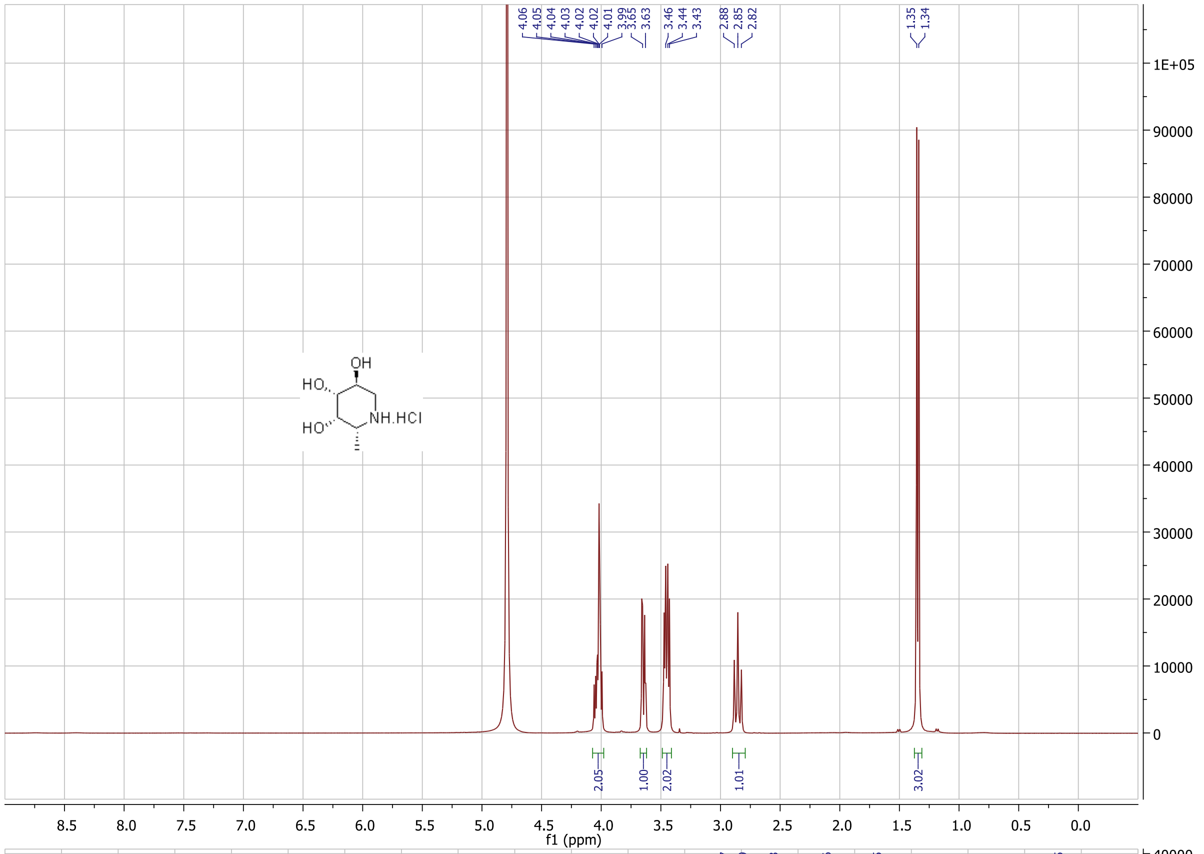

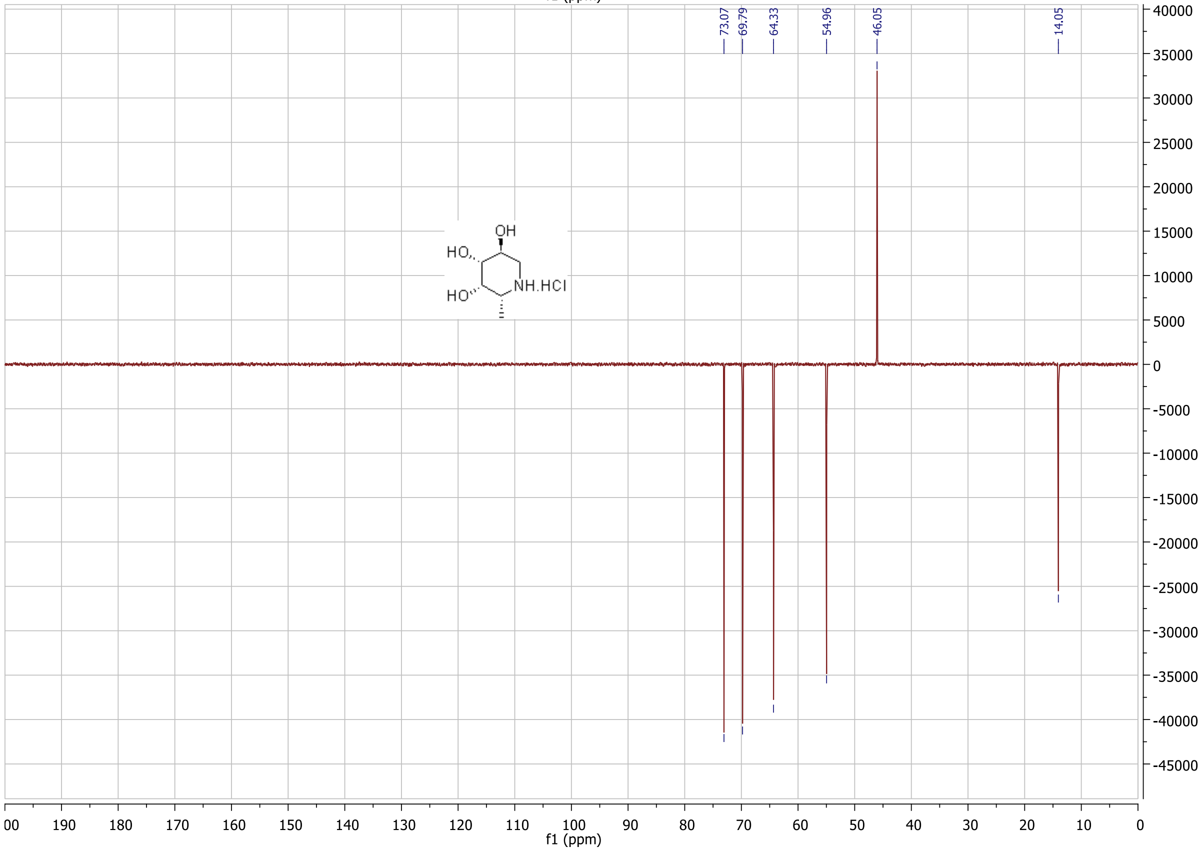

^{1}H and ^{13}C NMR ($CDCl_3$) compound **45**

tert-Butyl (3a*S*,4*R*,7*S*,7a*R*)-7-hydroxy-2,2,4-trimethyltetrahydro-[1,3]dioxolo[4,5-c]pyridine-5(4*H*)-carboxylate

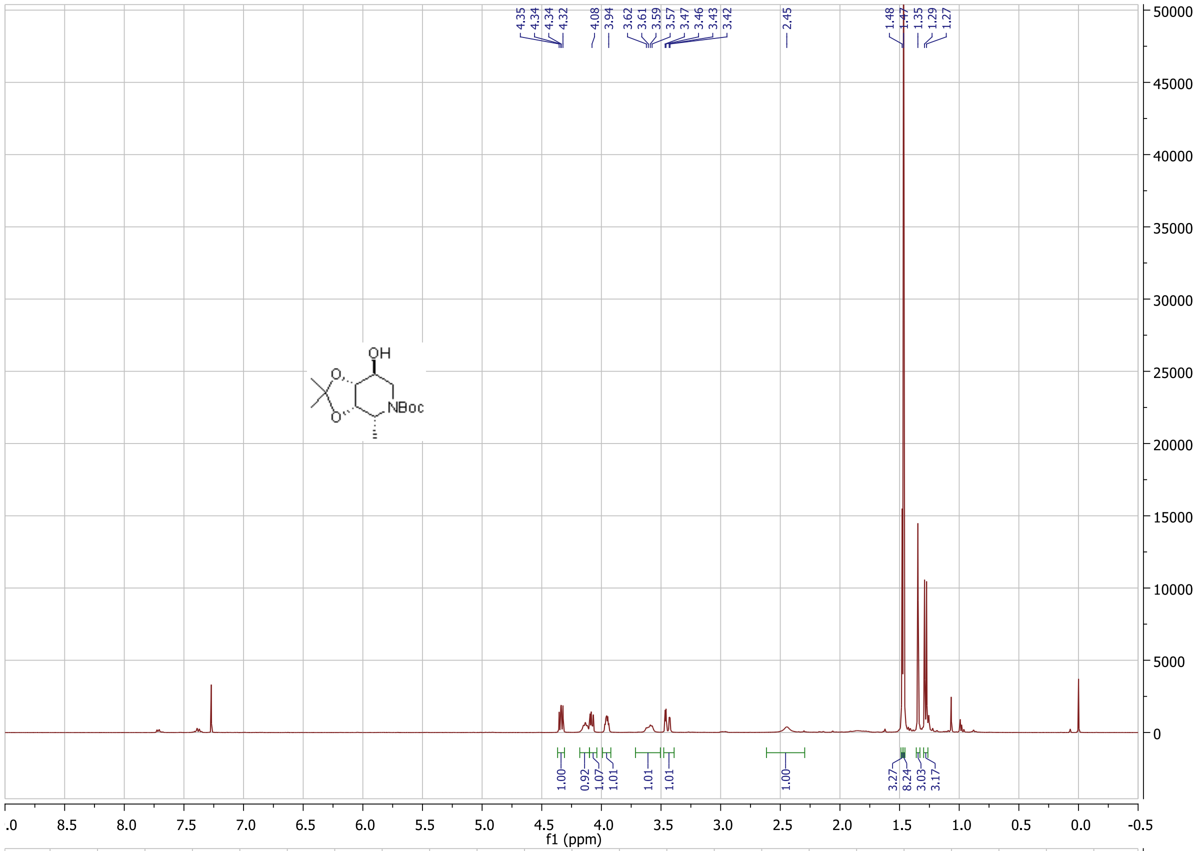

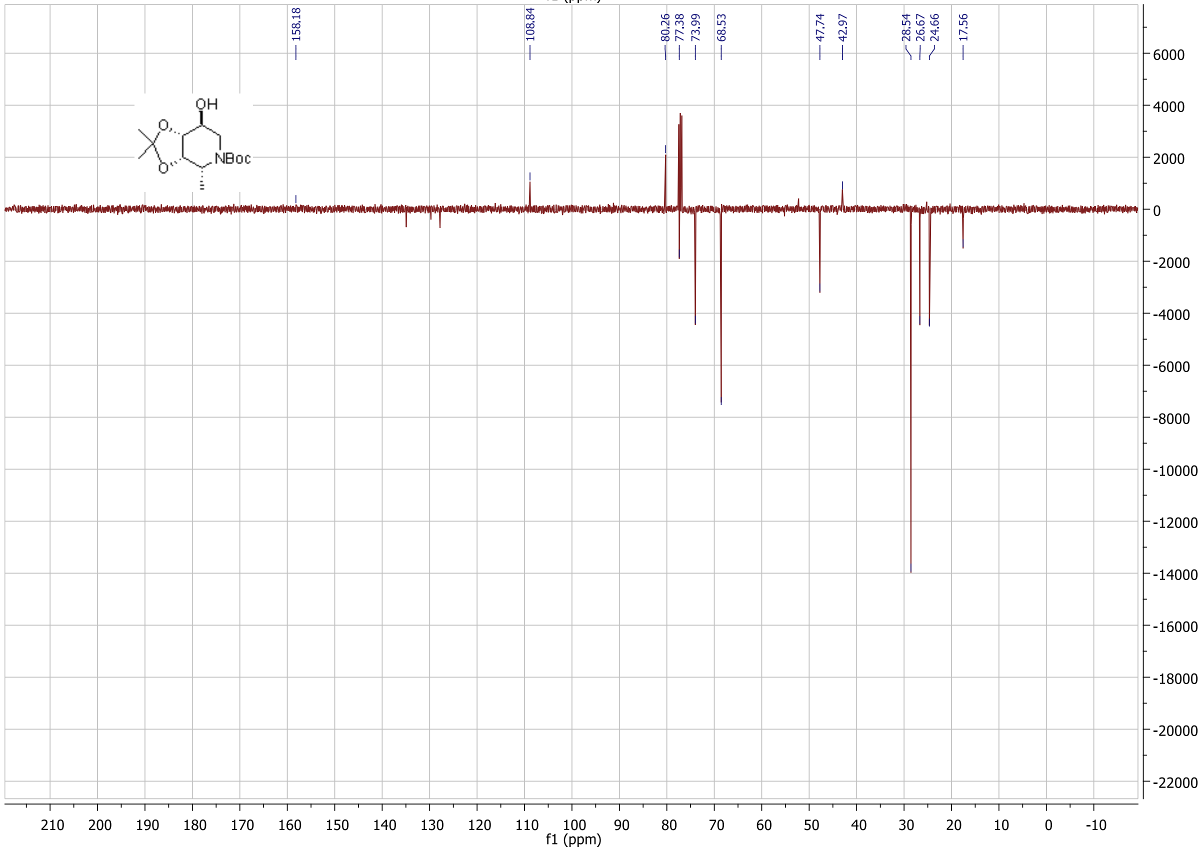

^{1}H and ^{13}C NMR ($CDCl_3$)

tert-Butyl (3a*S*,4*R*,7a*S*)-2,2,4-trimethyl-7-oxotetrahydro-[1,3]dioxolo[4,5-c]pyridine-5(4*H*)-carboxylate

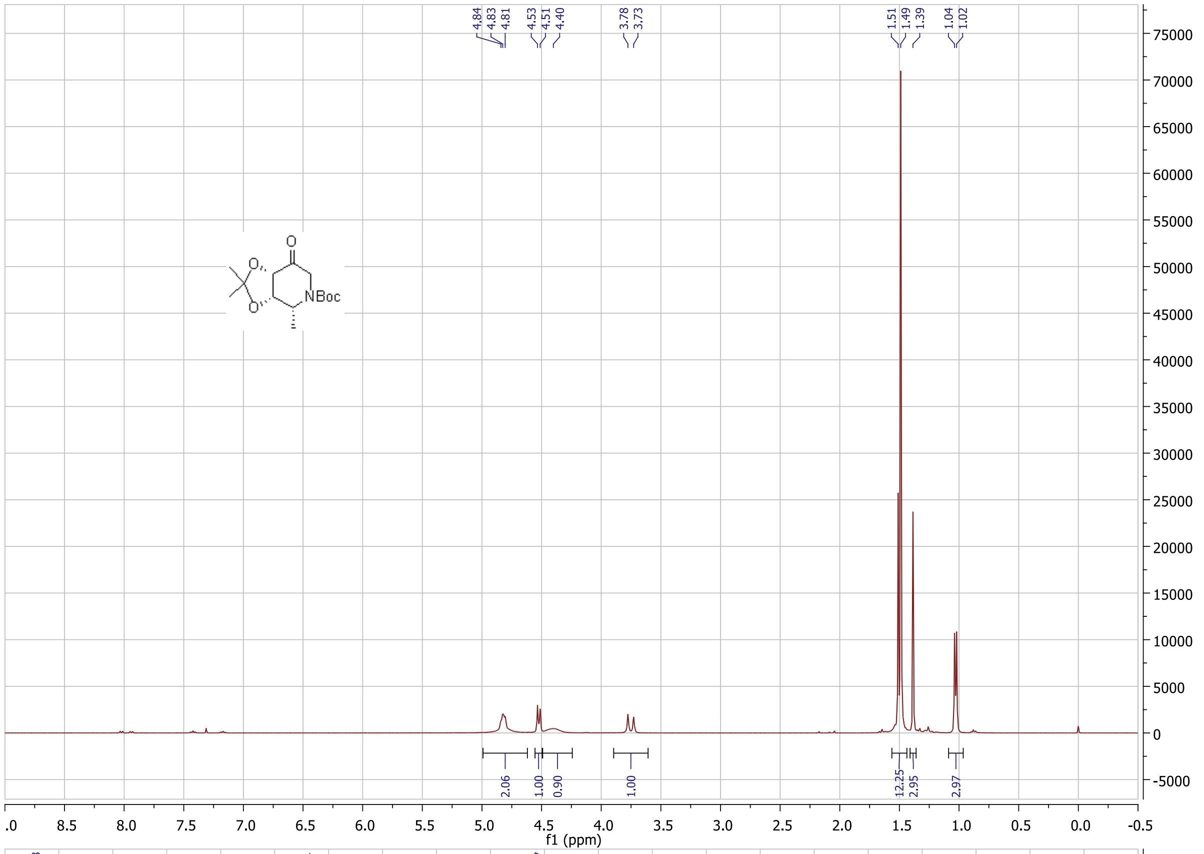

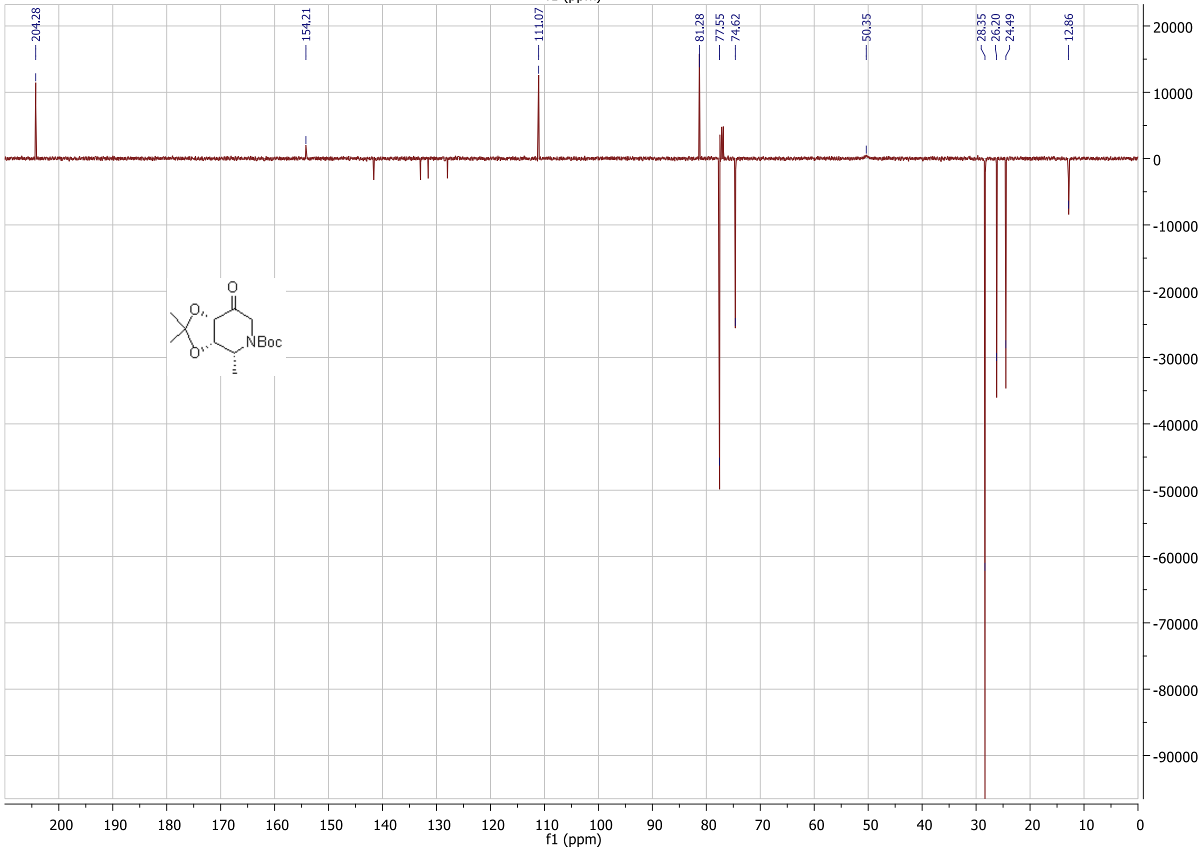

^{1}H and ^{13}C NMR ($CDCl_3$) compound **46**

tert-Butyl (3a*S*,4*R*,7*R*,7a*R*)-7-hydroxy-2,2,4-trimethyltetrahydro-[1,3]dioxolo[4,5-c]pyridi-ne-5(4*H*)-carboxylate

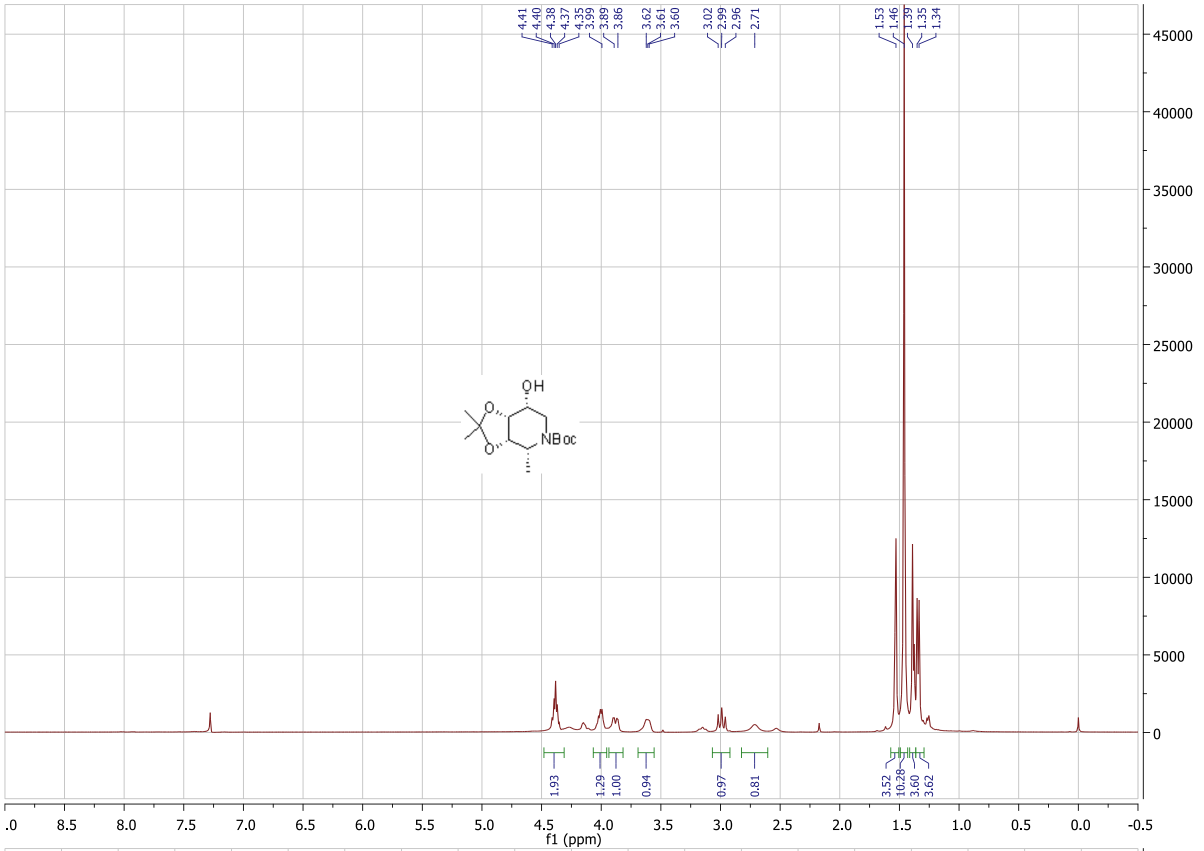

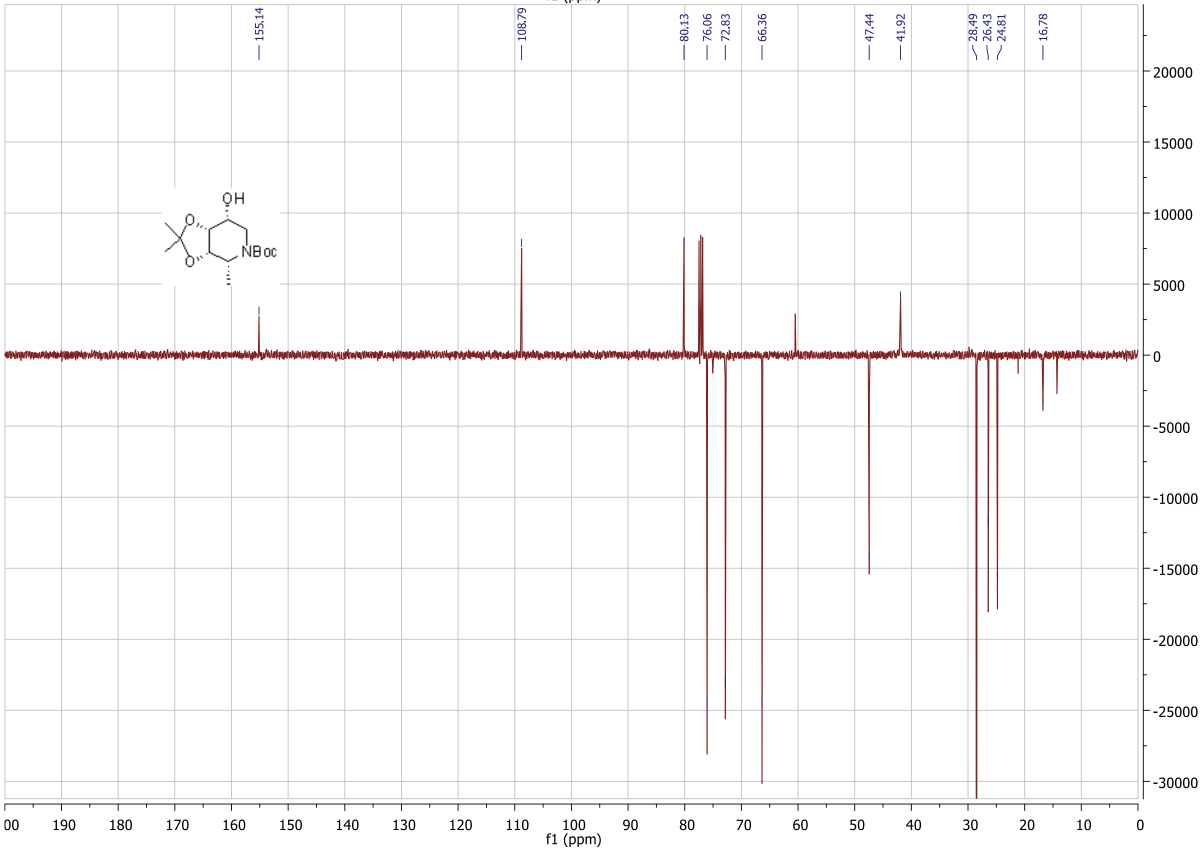

^1H and ^{13}C NMR (D_2O)

(2*R*,3*S*,4*R*,5*R*)-2-methylpiperidine-3,4,5-triol hydrochloride **(11)**

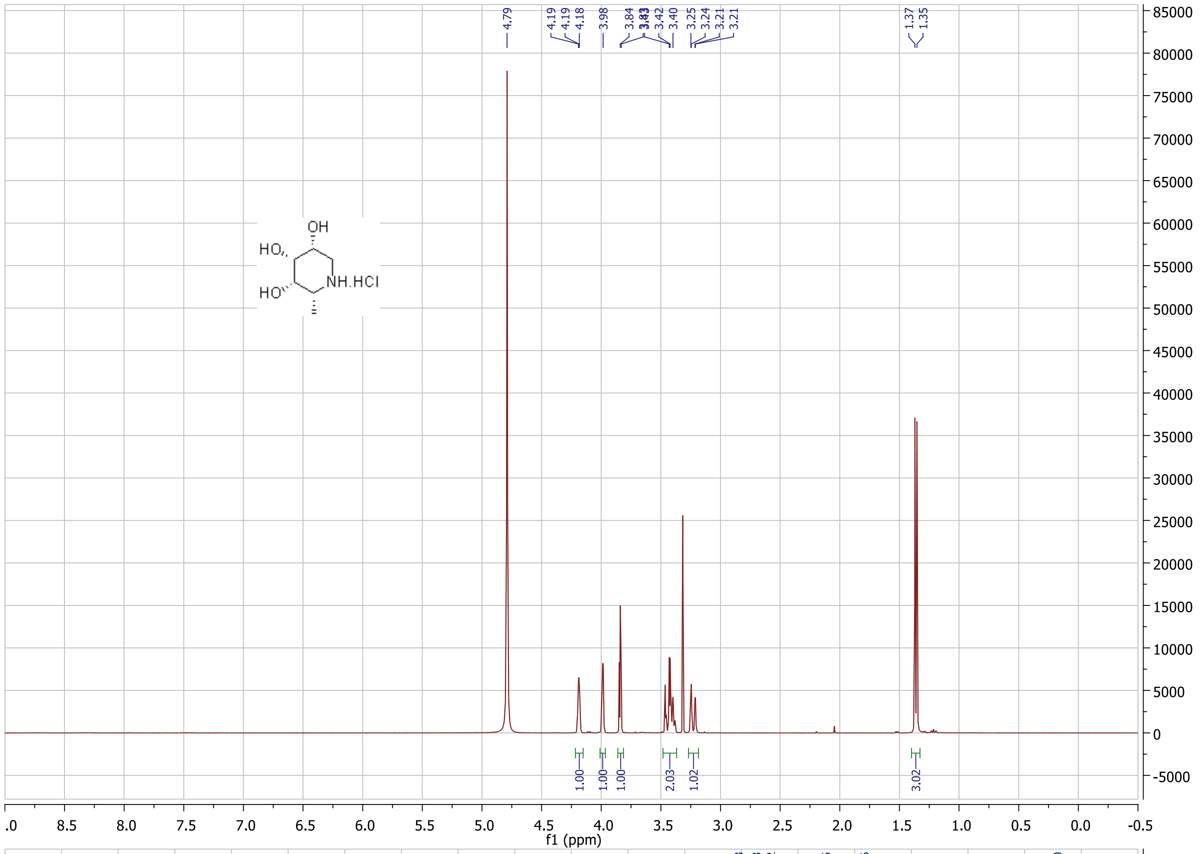

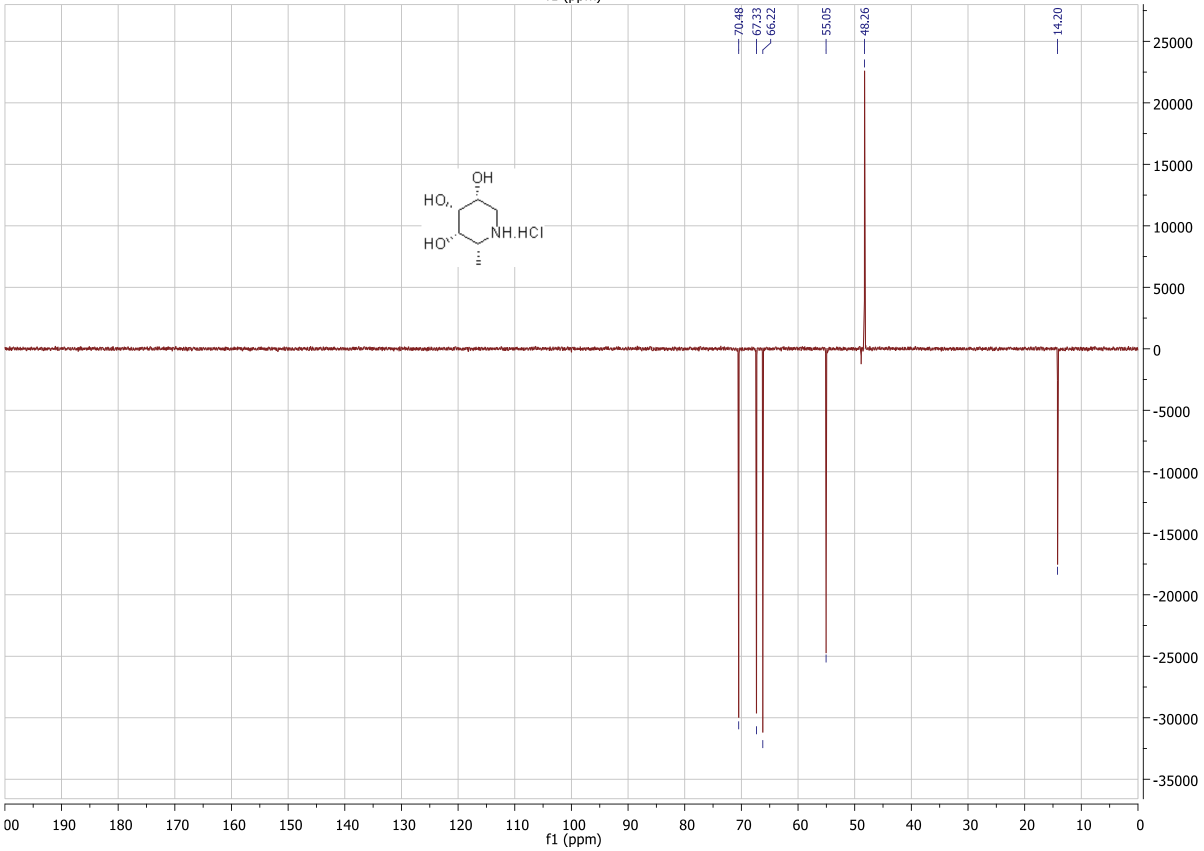

^{1}H and ^{13}C NMR ($CDCl_3$) compound **47**

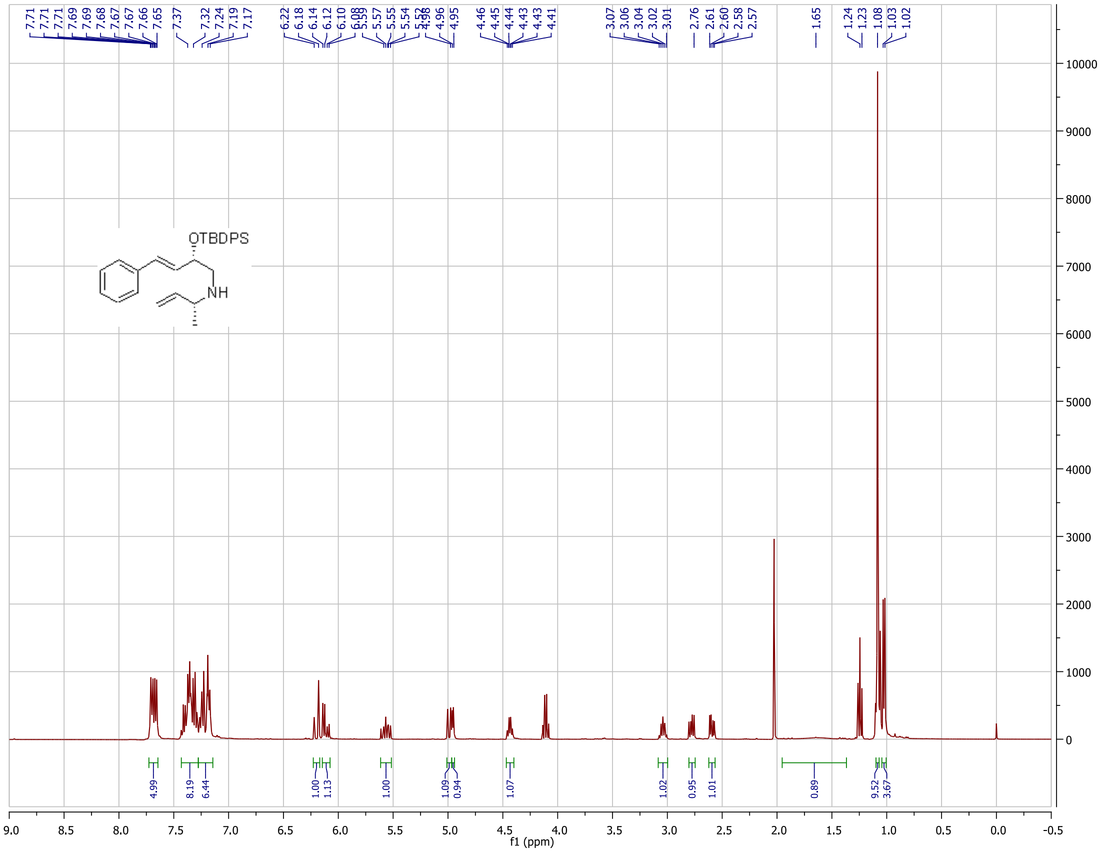

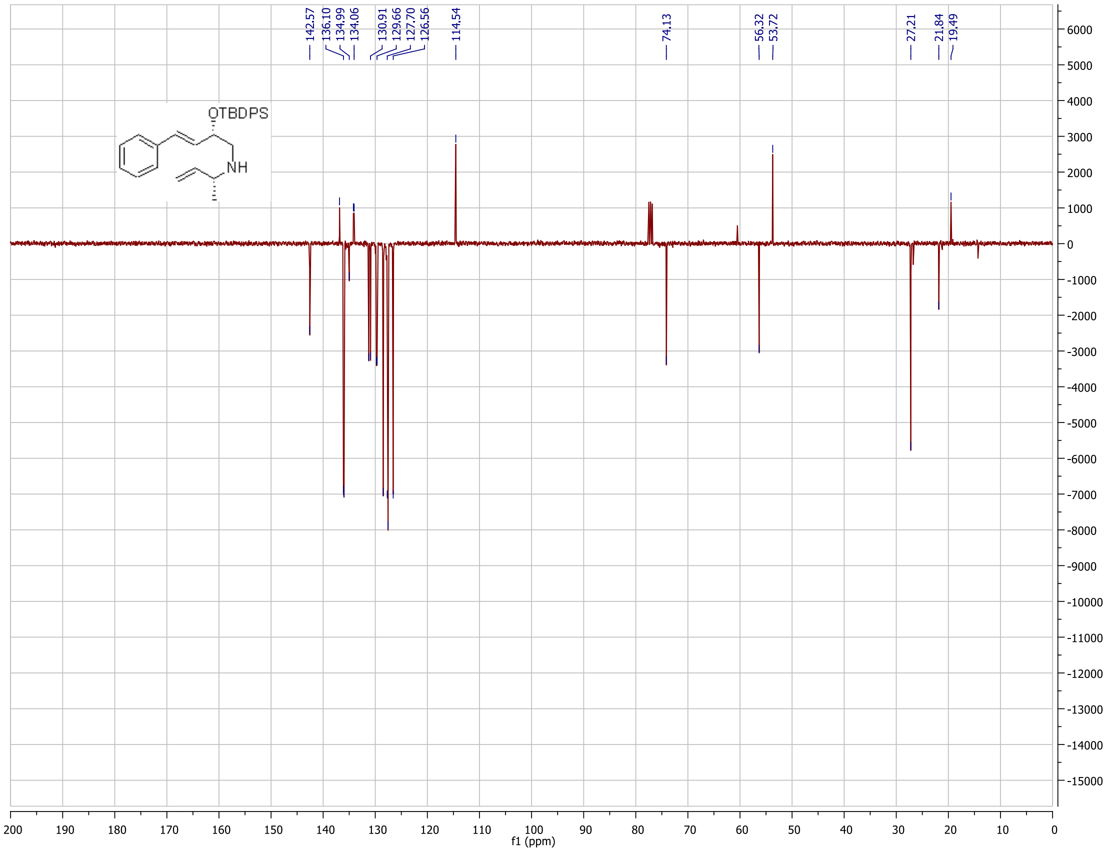

^{1}H and ^{13}C NMR ($CDCl_3$)

tert-Butyl ((*R*)-but-3-en-2-yl)((*S*,*E*)-2-((*tert*-butyldiphenylsilyl)oxy)-4-phenylbut-3-en-1-yl)carbamate

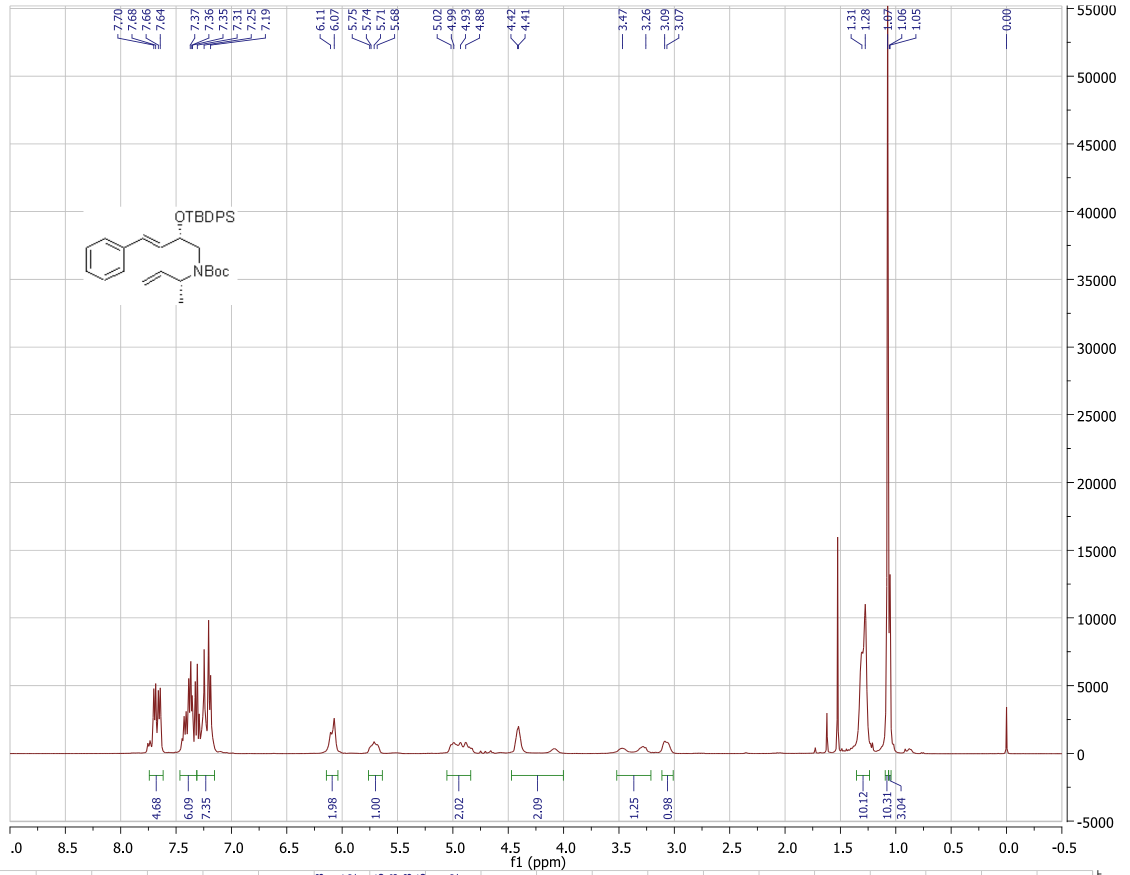

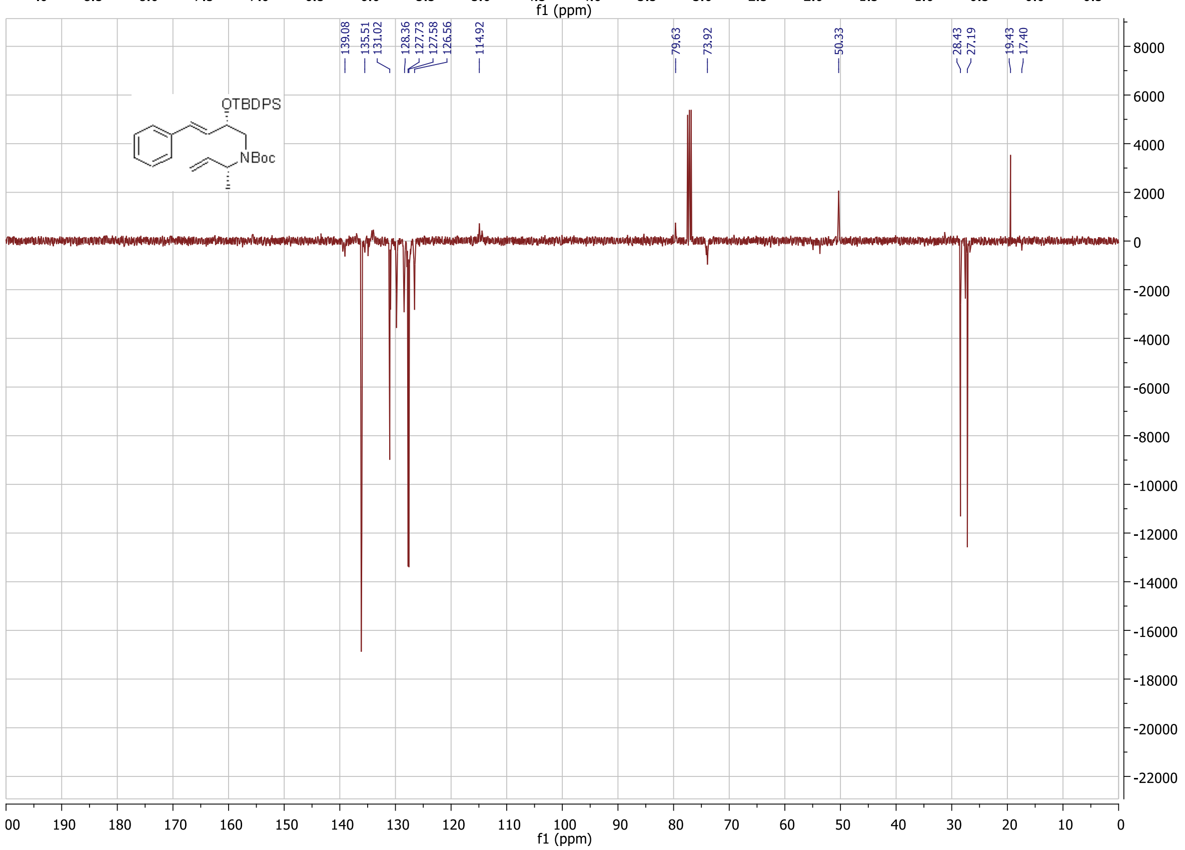

^{1}H and ^{13}C NMR ($CDCl_3$)

tert-Butyl (3*S*,6*R*)-3-((*tert*-butyldiphenylsilyl)oxy)-6-methyl-3,6-dihydropyridine-1(2*H*)-carboxylate **(48)**

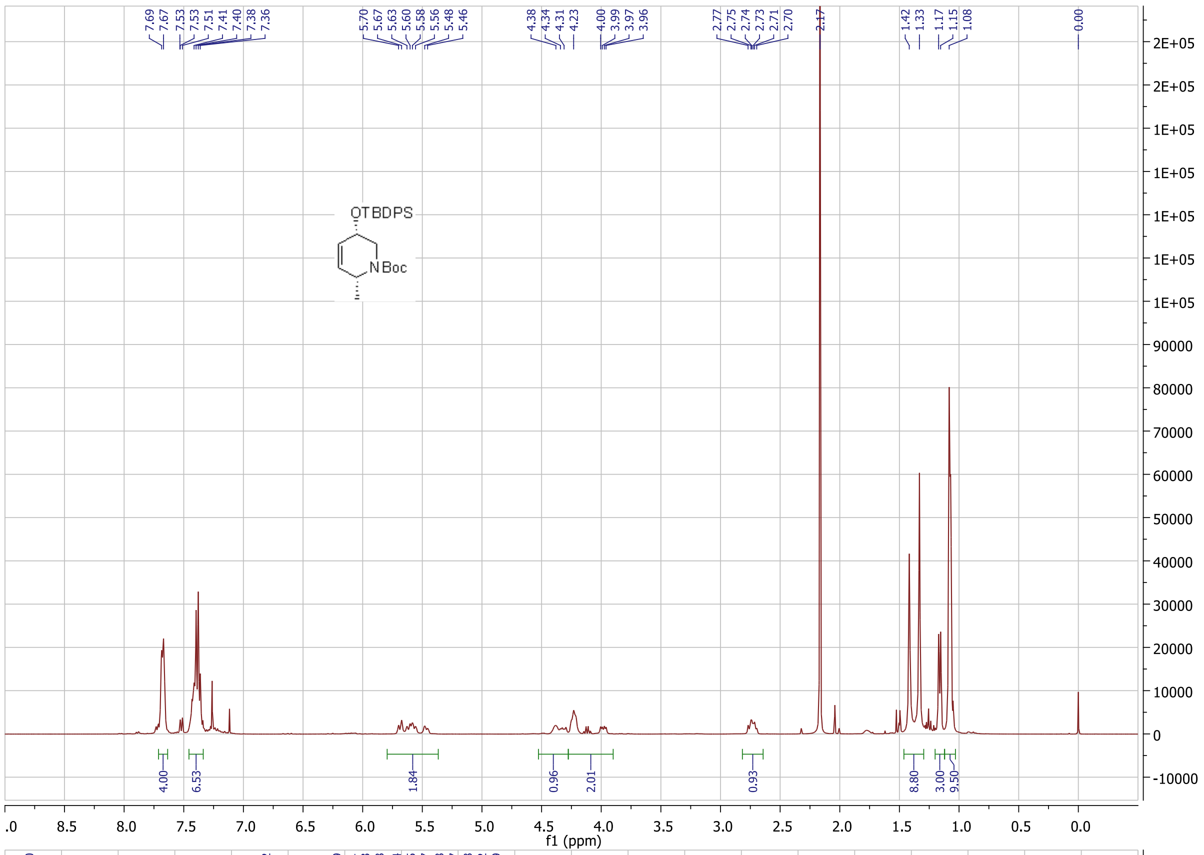

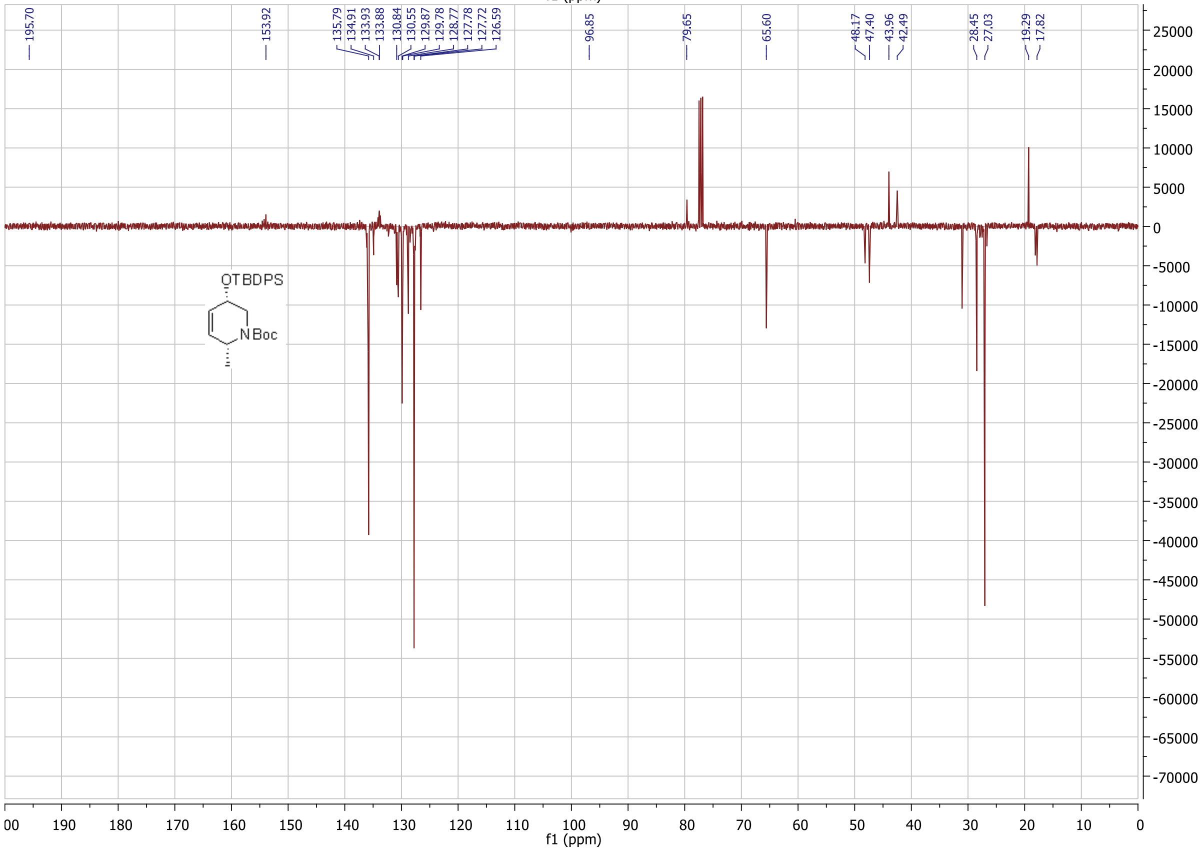

^{1}H and ^{13}C NMR ($CDCl_3$)

tert-Butyl (2*R*,3*R*,4*R*,5*R*)-5-((*tert*-butyldiphenylsilyl)oxy)-3,4-dihydroxy-2-methylpiperidine-1-carboxylate (**49**)

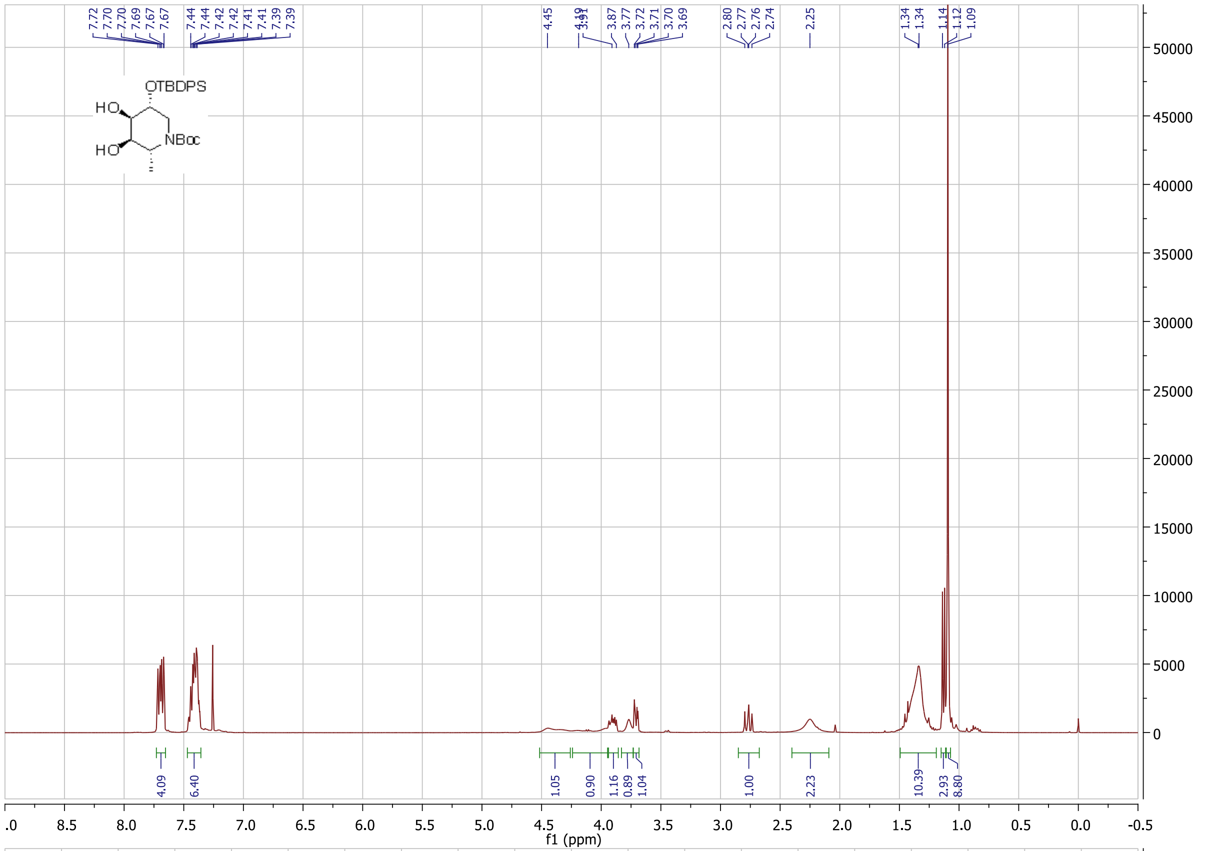

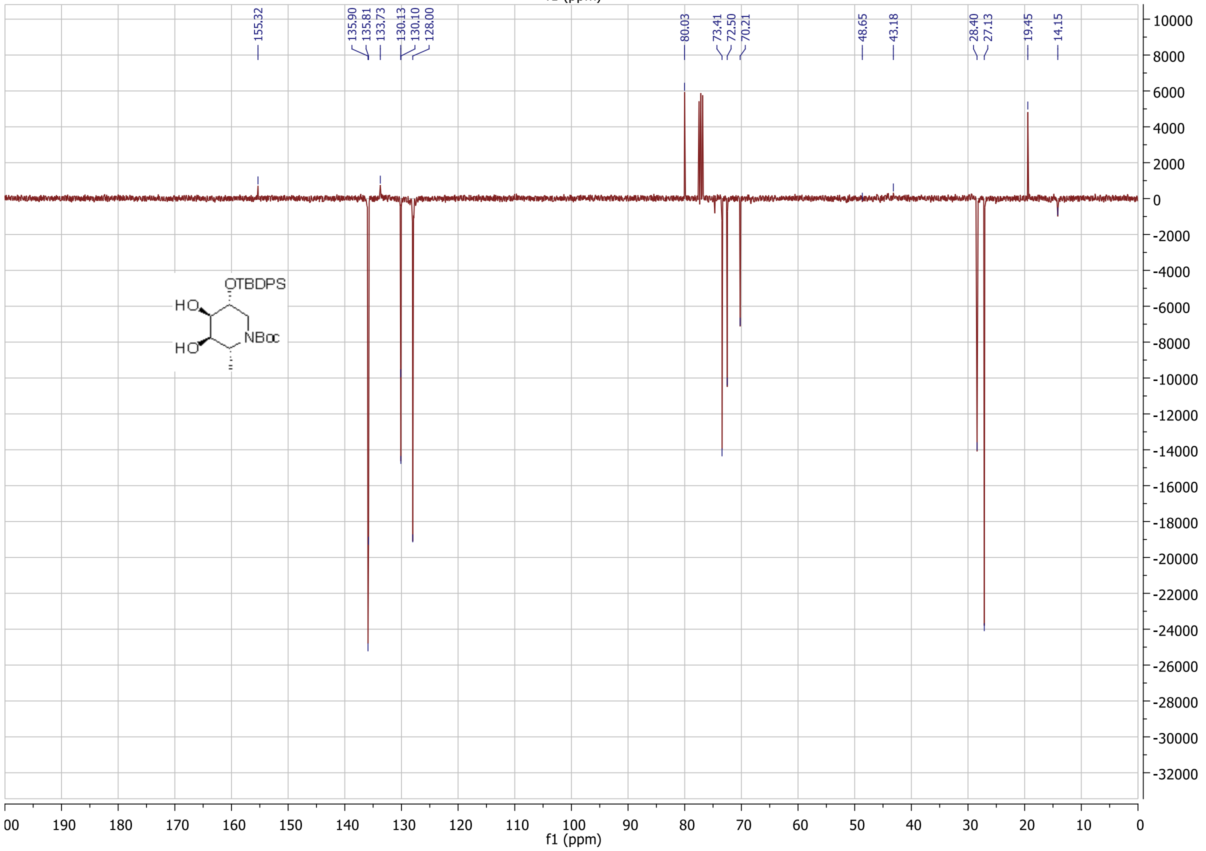

^1H and ^{13}C NMR (CD_3OD)

tert-Butyl (2*R*,3*R*,4*S*,5*R*)-3,4,5-trihydroxy-2-methylpiperidine-1-carboxylate

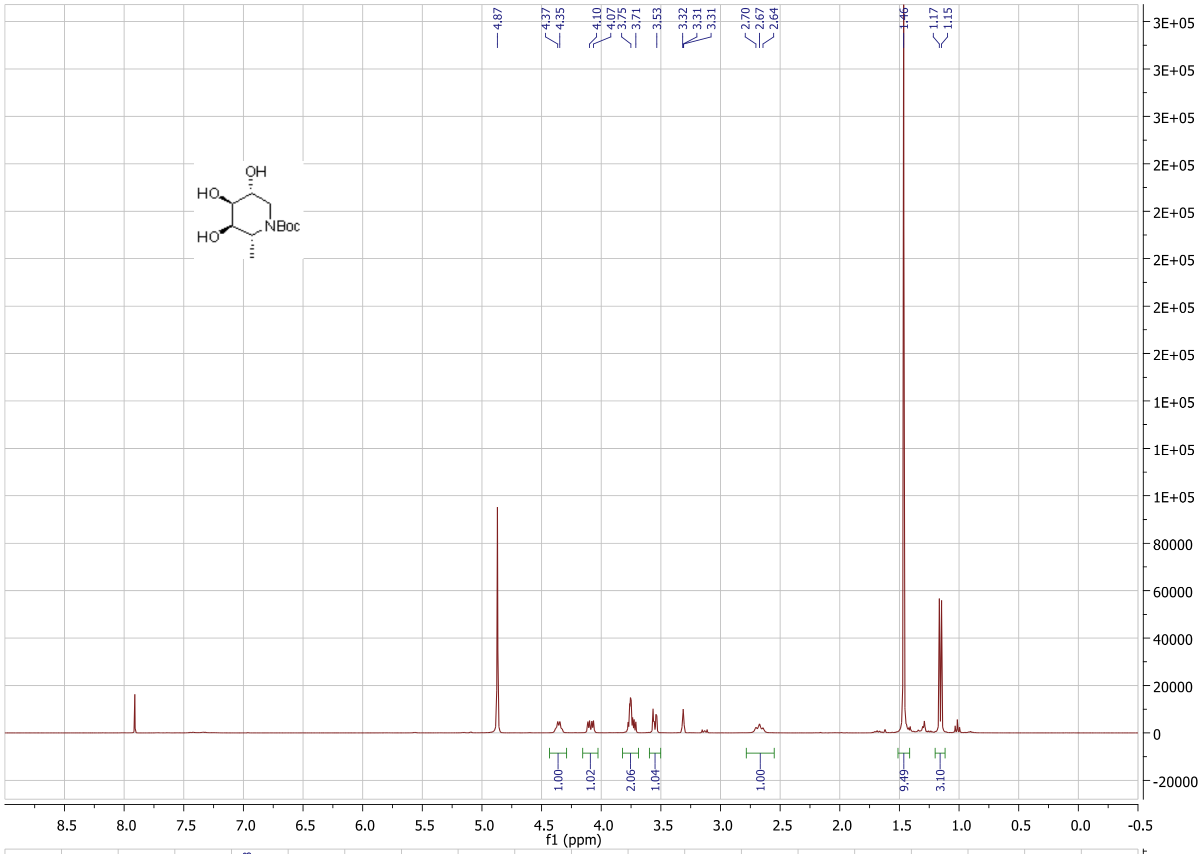

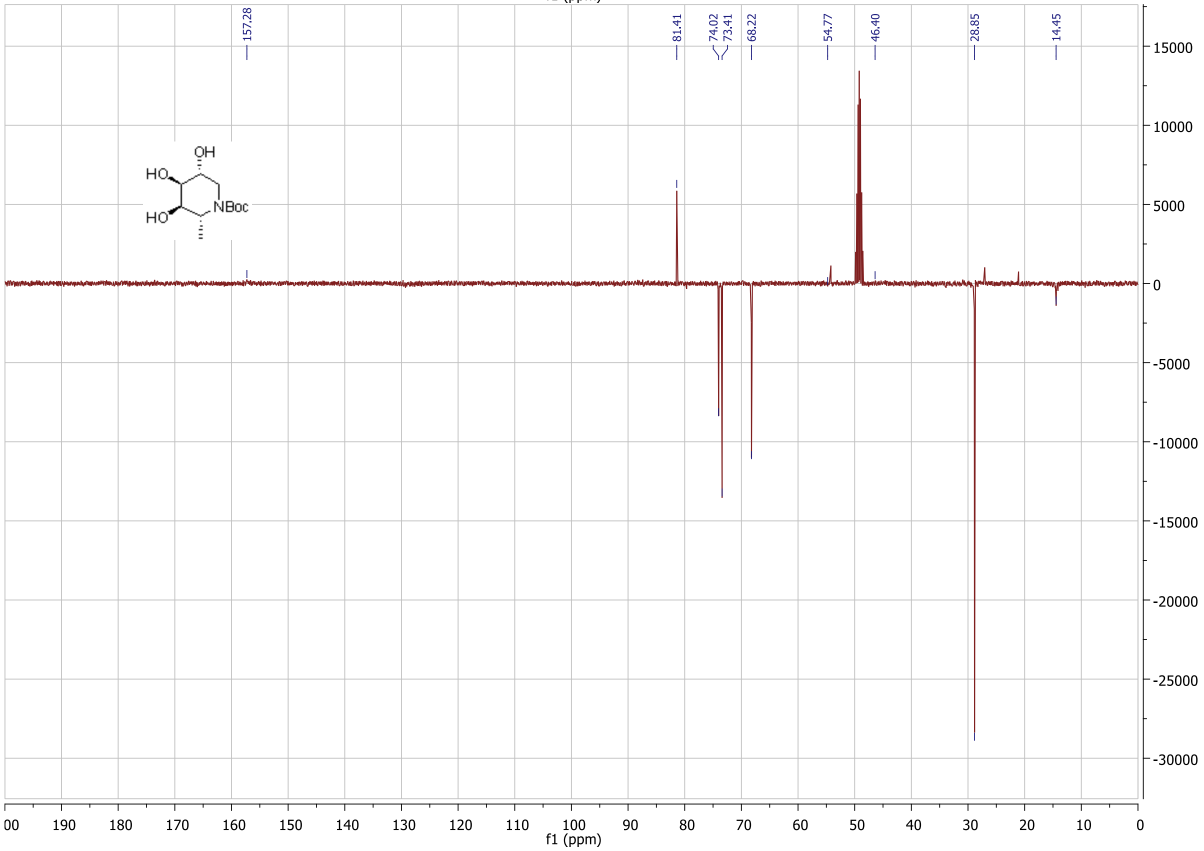

^{1}H and ^{13}C NMR (D_2O)

(2*R*,3*R*,4*S*,5*R*)-2-methylpiperidine-3,4,5-triol hydrochloride (**12)**

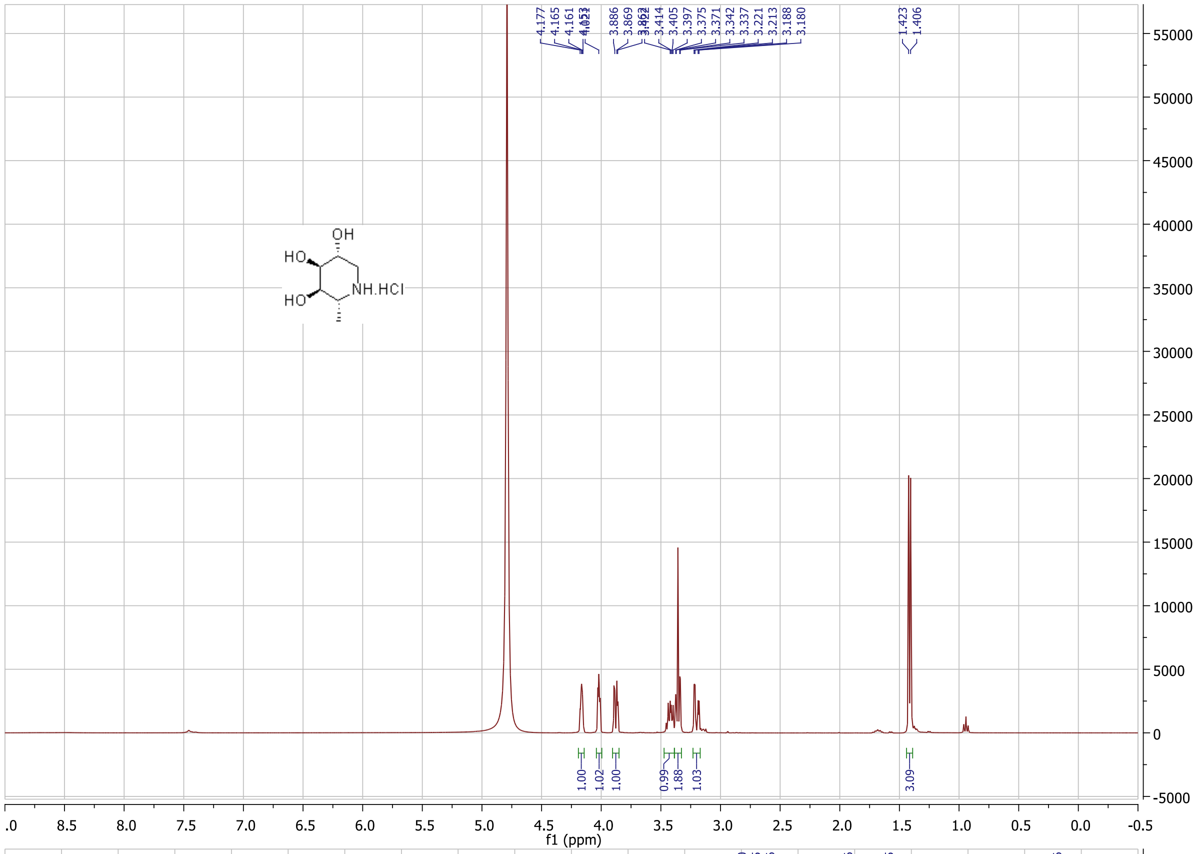

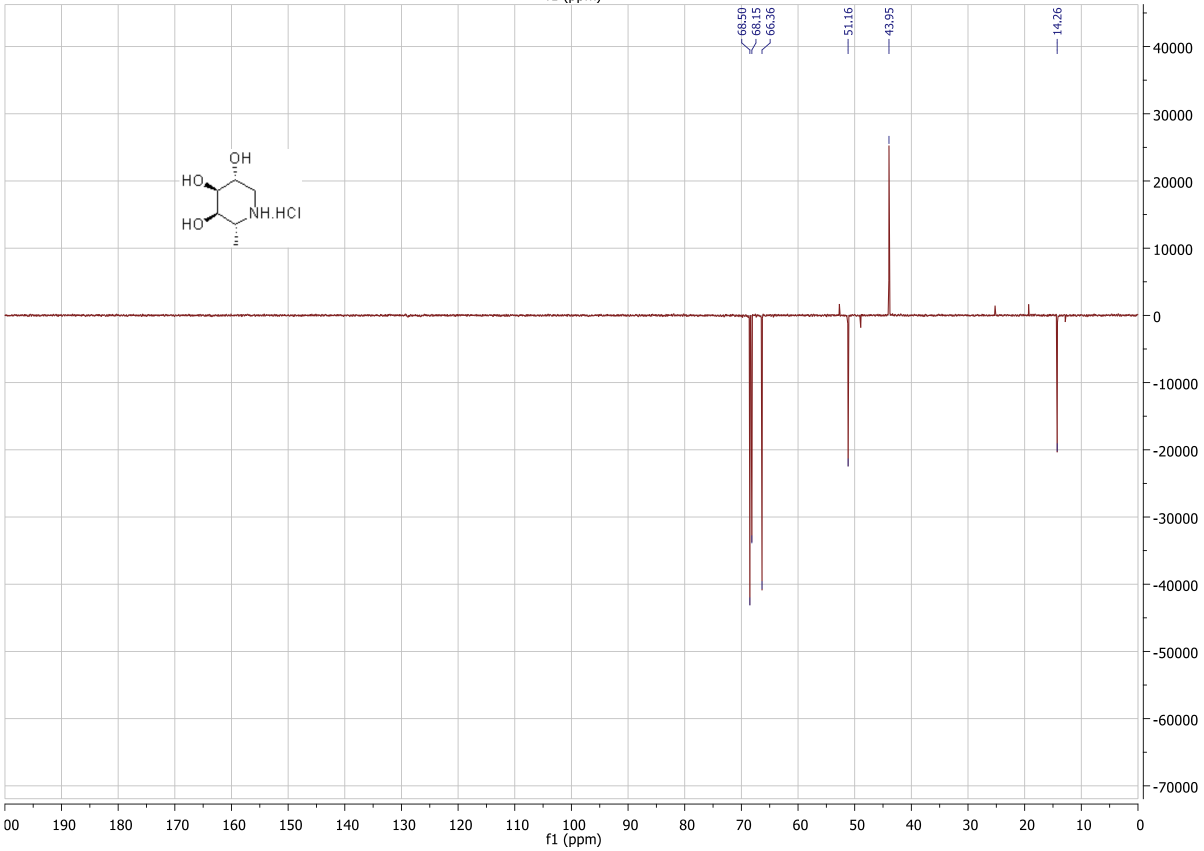

^{1}H and ^{13}C NMR ($CDCl_3$)

tert-Butyl (3a*R*,4*R*,7*R*,7a*S*)-7-hydroxy-2,2,4-trimethyltetrahydro-[1,3]dioxolo[4,5-c]pyridine-5(4*H*)-carboxylate

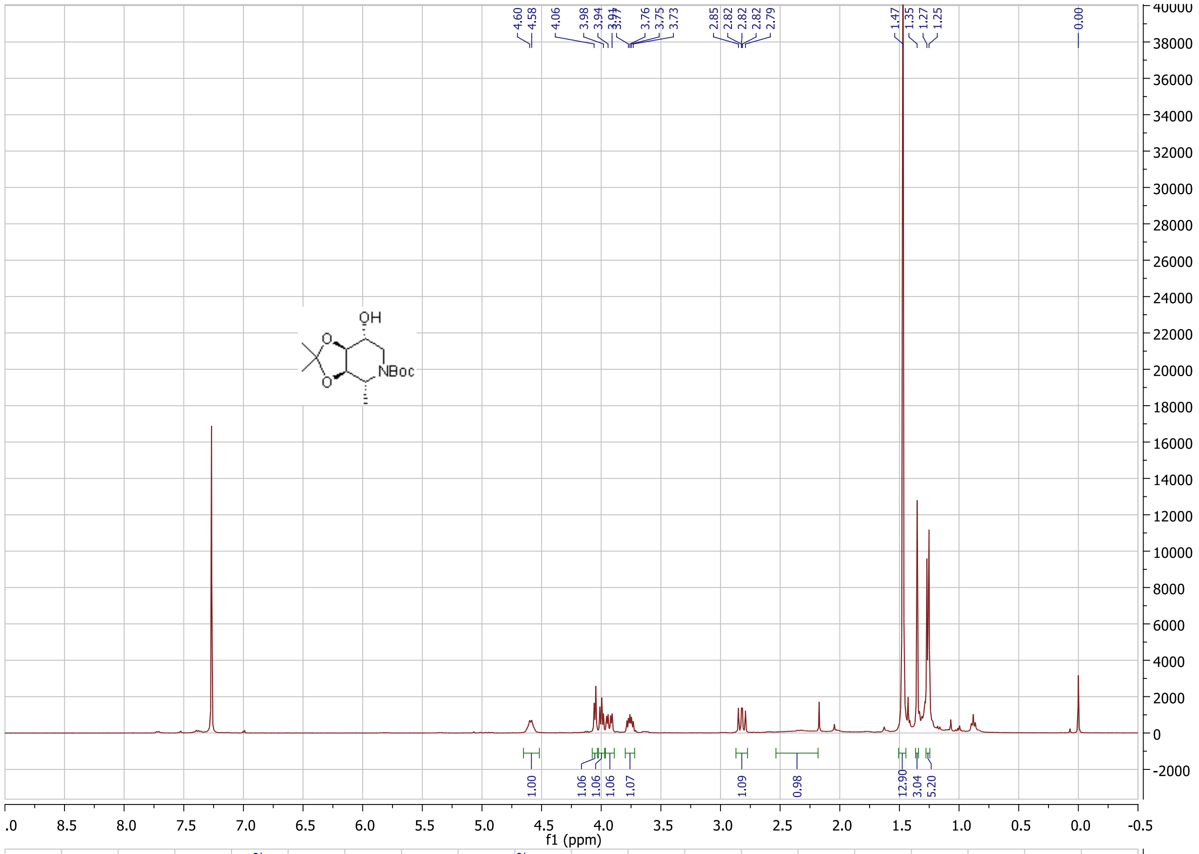

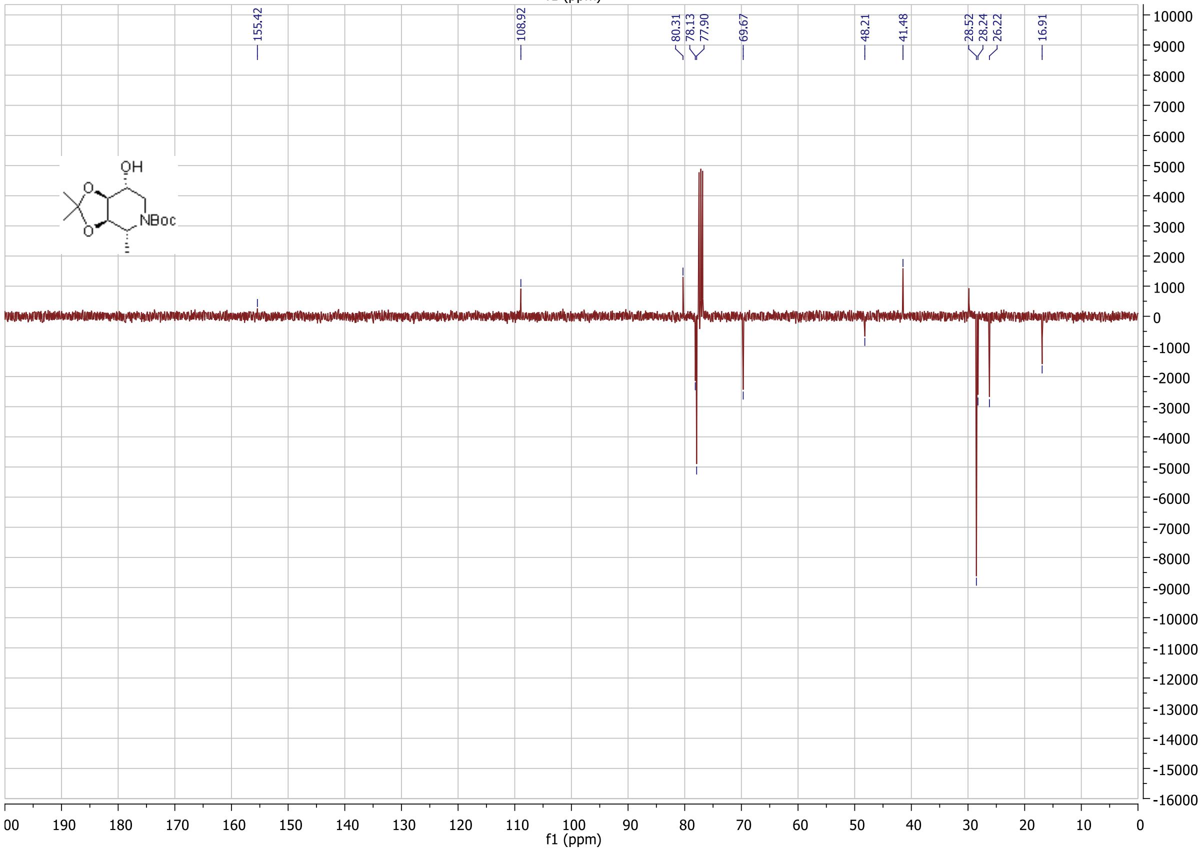

^{1}H and ^{13}C NMR ($CDCl_3$)

tert-Butyl (3a*R*,4*R*,7a*R*)-2,2,4-trimethyl-7-oxotetrahydro-[1,3]dioxolo[4,5-c]pyridine-5(4*H*)-carboxylate

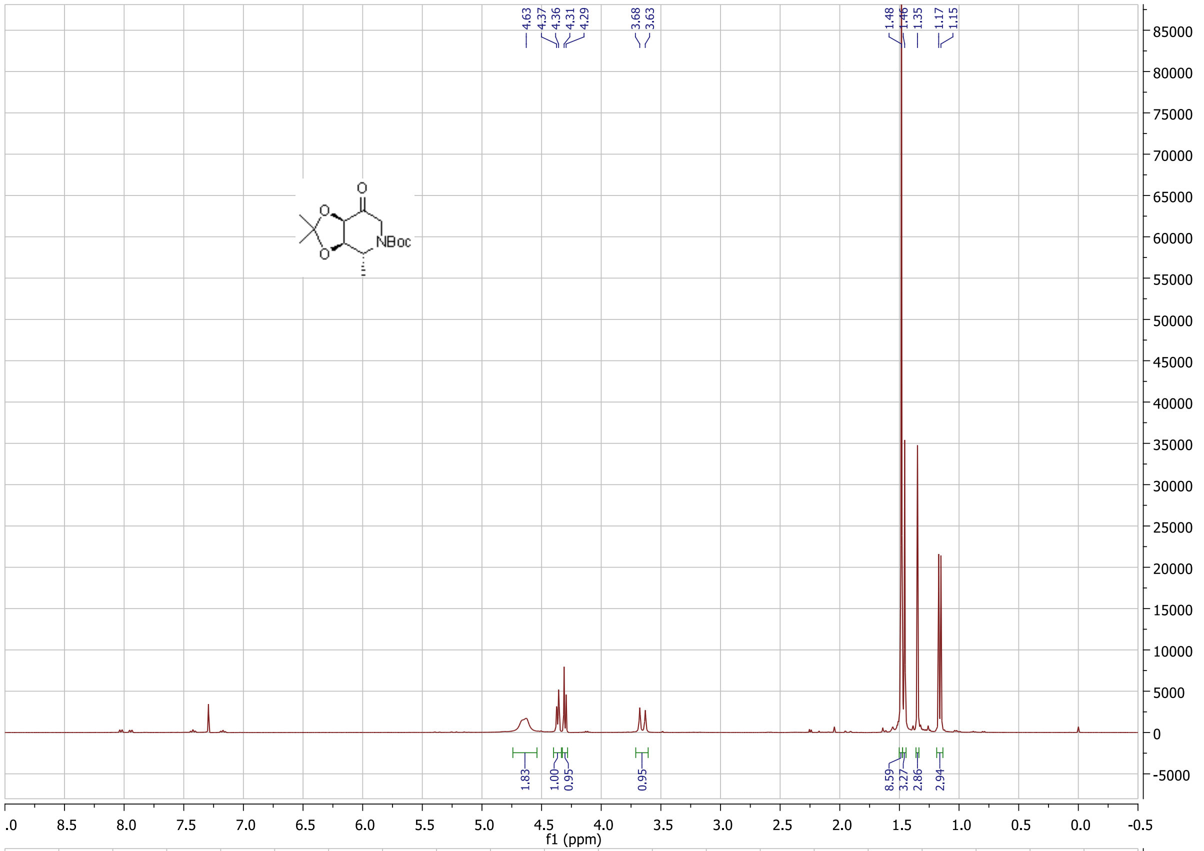

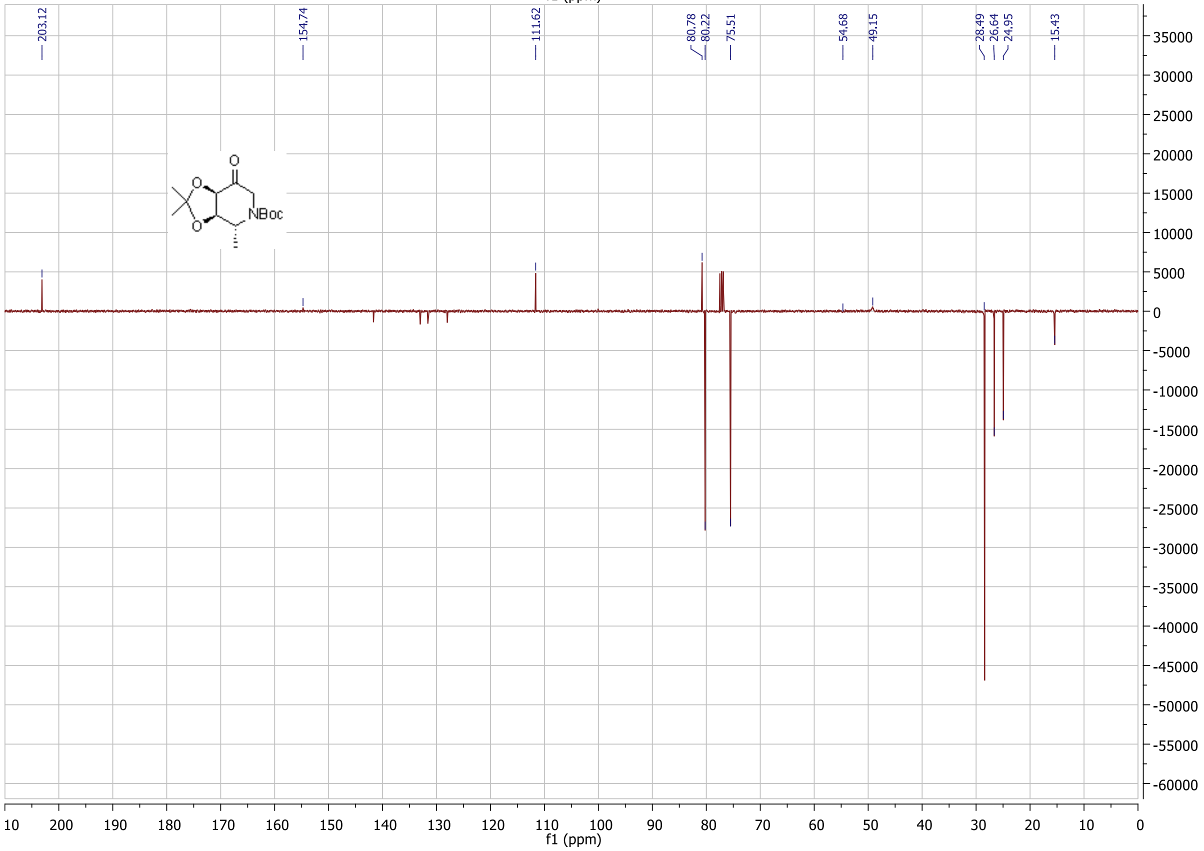

^{1}H and ^{13}C NMR ($CDCl_3$) compound **50**

tert-Butyl (3a*R*,4*R*,7*S*,7a*S*)-7-hydroxy-2,2,4-trimethyltetrahydro-[1,3]dioxolo[4,5-c]-pyridine-5(4*H*)-carboxylate

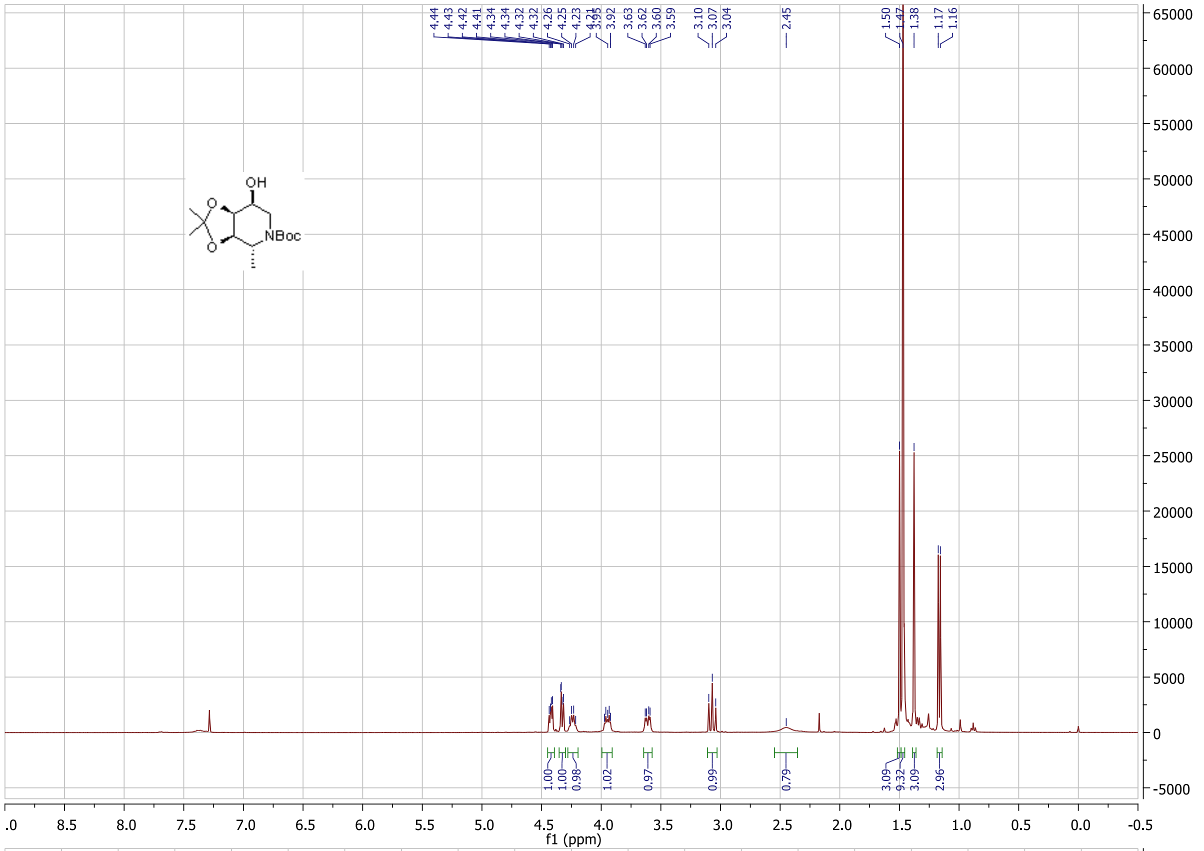

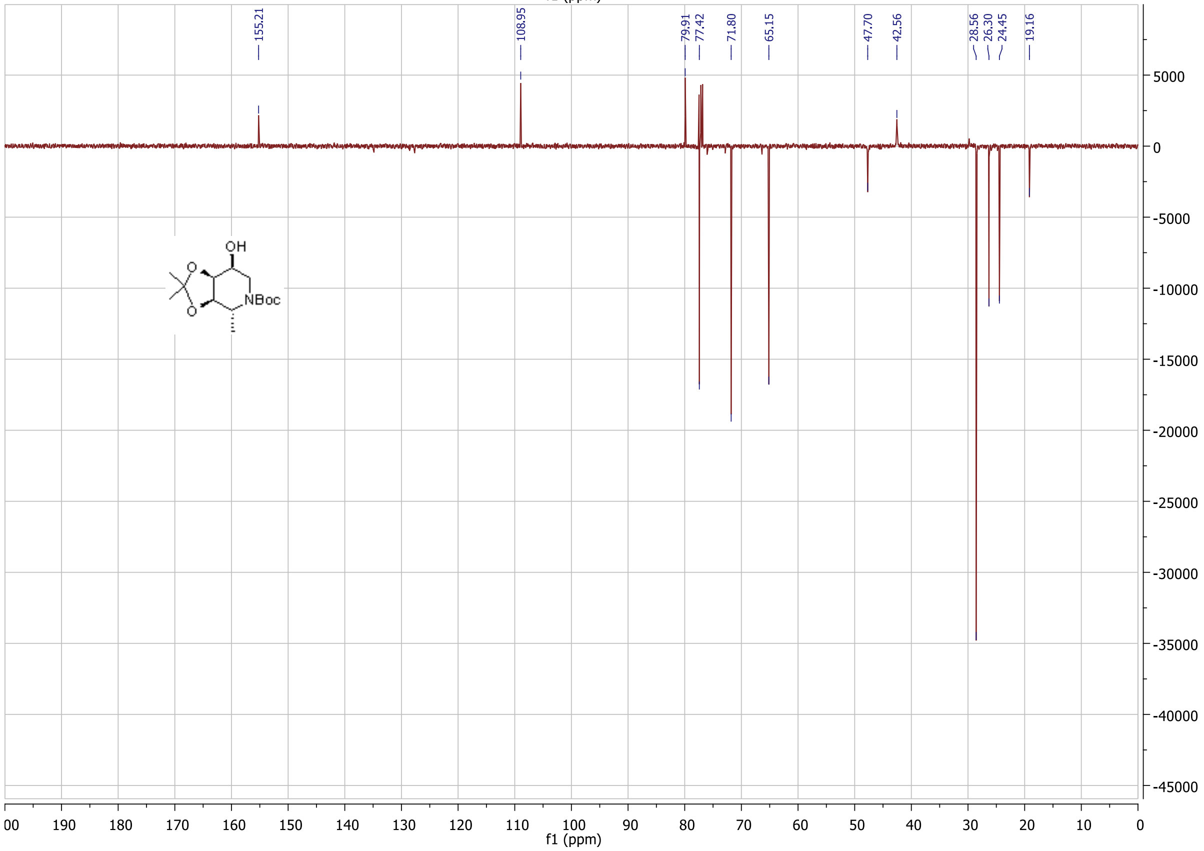

^{1}H and ^{13}C NMR (D_2O)

(2*R*,3*R*,4*S*,5*S*)-2-methylpiperidine-3,4,5-triol hydrochloride **(13)**

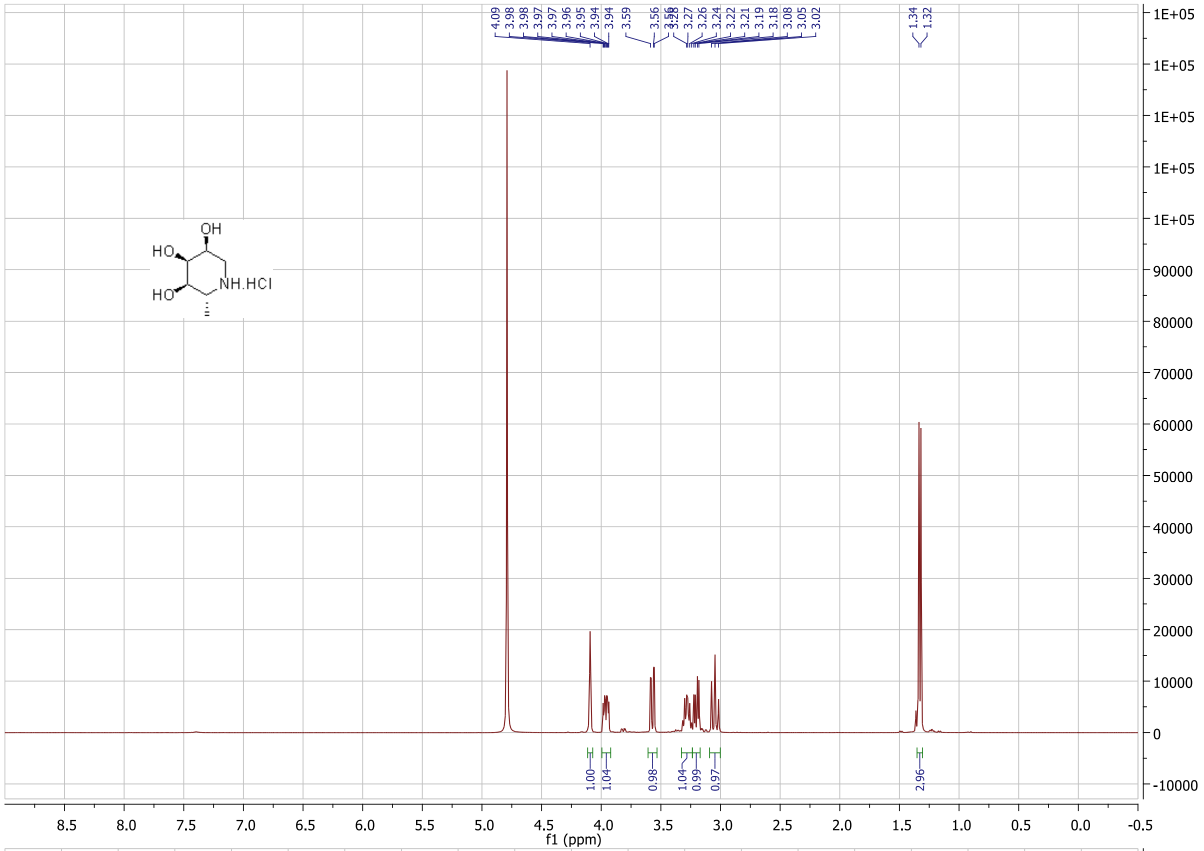

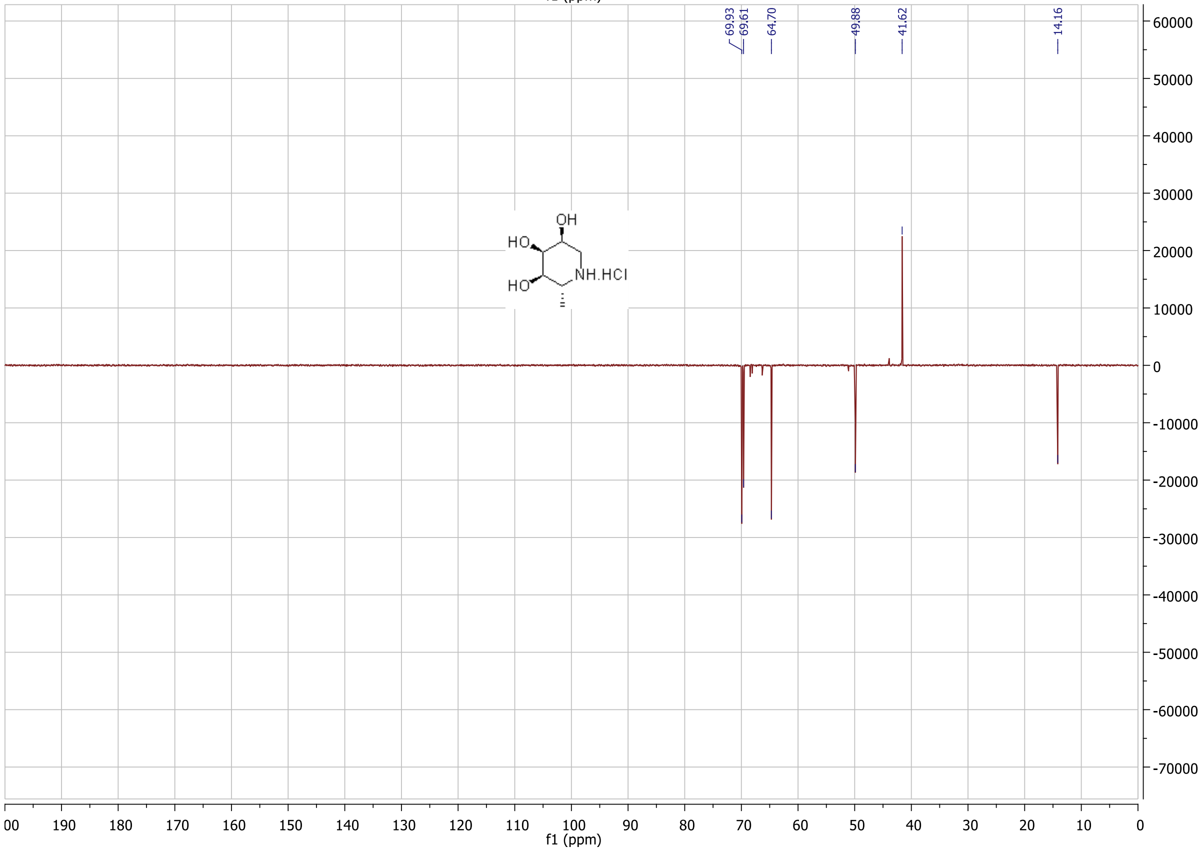

Supplement: Supplementary file 1 [file SC-006-C4SC03739A-s001.pdf]
